# *Leptospira borgpetersenii* Leucine-Rich Repeat Proteins Provide Strong Protective Efficacy as Novel Leptospiral Vaccine Candidates

**DOI:** 10.3390/tropicalmed8010006

**Published:** 2022-12-22

**Authors:** Siriwan Prapong, Yada Tansiri, Tepyuda Sritrakul, Sineenat Sripattanakul, Aukkrimapann Sopitthummakhun, Gerd Katzenmeier, Chin-Lin Hsieh, Sean P. McDonough, Teerasak Prapong, Yung-Fu Chang

**Affiliations:** 1 Faculty of Veterinary Medicine, Kasetsart University, Bangkok 10900, Thailand; 2 The Interdisciplinary Graduate Program in Genetic Engineering, The Graduate School, Kasetsart University, Bangkok 10900, Thailand; 3 Center for Advanced Studies for Agriculture and Food (CASAF), Kasetsart University, Bangkok 10900, Thailand; 4 Faculty of Medicine, King Mongkut’s Institute of Technology Ladkrabang, Bangkok 10520, Thailand; 5 Department of Veterinary Public Health, Faculty of Veterinary Medicine, Kasetsart University, Kamphaengsaen Campus, Nakorn Pathom 73140, Thailand; 6 Akkhraratchakumari Veterinary College, Walailak University, Nakhon Si Thammarat 80160, Thailand; 7 Department of Population Medicine and Diagnostic Sciences, College of Veterinary Medicine, Cornell University, Ithaca, NY 14853, USA; 8 Department of Biomedical Science, College of Veterinary Medicine, Cornell University, Ithaca, NY 14853, USA

**Keywords:** leptospirosis, leucine-rich repeat (LRR), tropical infectious diseases, emerging and re-emerging diseases, vaccine, disease prevention, one health

## Abstract

Leucine-rich repeat (LRR) proteins are advocated for being assessed in vaccine development. Leptospiral LRR proteins were identified recently in silico from the genome of *Leptospira borgpetersenii* serogroup Sejroe, the seroprevalence of leptospiral infections of cattle in Thailand. Two LRR recombinant proteins, rKU_Sej_LRR_2012M (2012) and rhKU_Sej_LRR_2271 (2271), containing predicted immunogenic epitopes, were investigated for their cross-protective efficacies in an acute leptospirosis model with heterologous *Leptospira* serovar Pomona, though, strains from serogroup Sejroe are host-adapted to bovine, leading to chronic disease. Since serovar Pomona is frequently reported as seropositive in cattle, buffaloes, pigs, and dogs in Thailand and causes acute and severe leptospirosis in cattle by incidental infection, the serogroup Sejroe LRR proteins were evaluated for their cross-protective immunity. The protective efficacies were 37.5%, 50.0%, and 75.0% based on the survival rate for the control, 2012, and 2271 groups, respectively. Sera from 2012-immunized hamsters showed weak bactericidal action compared to sera from 2271-immunized hamsters (*p* < 0.05). Therefore, bacterial tissue clearances, inflammatory responses, and humoral and cell-mediated immune (HMI and CMI) responses were evaluated only in 2271-immunized hamsters challenged with virulent *L. interrogans* serovar Pomona. The 2271 protein induced prompt humoral immune responses (*p* < 0.05) and leptospiral tissue clearance, reducing tissue inflammation in immunized hamsters. In addition, protein 2271 and its immunogenic peptides stimulated splenocyte lymphoproliferation and stimulated both HMI and CMI responses by activating Th1 and Th2 cytokine gene expression in vaccinated hamsters. Our data suggest that the immunogenic potential renders rhKU_Sej_LRR_2271 protein a promising candidate for the development of a novel cross-protective vaccine against animal leptospirosis.

## 1. Introduction

Leptospirosis is the most widespread zoonotic disease and is considered one of the neglected and emerging diseases in the world [1,2]. In humans and animals, the disease is caused by pathogenic bacteria of the genus *Leptospira* and affects livestock in all parts of the world. More than 200 serovars of these pathogenic spirochetes have been identified [1,3]. *L. borgpetersenii* serogroup Sejroe is an important serogroup causing infection in Thailand [4,5]. The distribution pattern of *Leptospira* serovars across regions in Thailand is similar in cattle but different in buffaloes. Since *Leptospira* serovar Tarassovi and serogroup Sejroe are predominant serogroups in cattle [4,6], researchers have focused on vaccine development for preventing leptospiral infections in tropical environments such as South-East Asia and Latin American countries [7,8]. It is important to emphasize that strains from the Sejroe serogroup are host-adapted to bovine, leading to a chronic and silent disease affecting the reproductive tract of cows, recognized as Bovine Genital Leptospirosis [9]. In addition, serogroup/serovars prevalence in MAT-positive in cattle, buffaloes, and pigs in Thailand were Sejroe, Ranarum, Mini, Pomona, and Bataviae in cattle; Mini, Sejroe, Bratislava, Pomona, and Ranarum in buffaloes; Ranarum, Pomona, Bratislava, and Bataviae in pigs [10]; and serogroups Icterohaemorrhagiae, Canicola, Pomona, Bataviae and Sejroe in dogs [10,11]. The serogroup Pomona serovar Pomona had been reported to cause acute and severe leptospirosis in cattle by incidental infection [12,13] even though protective immunity against the acute disease caused by *L. interrogans* serovar Pomona was observed, stimulated by LRR proteins cloned from *L. borgpetersenii* serogroup Sejroe, the most prevalent leptospiral serovar in bovine livestock in Thailand and South-East Asia.

Currently, leptospiral vaccine development is pursued by two major strategies: (1) whole-cell bacteria and (2) recombinant protein [7,14,15]. Commercial whole-cell leptospiral vaccines, which are widely used for veterinary purposes, induce antibodies against the leptospiral lipopolysaccharide (LPS). The humoral and cellular immunity stimulated by these bacterins is limited to serovars and/or serogroups included in the vaccine formulation [15,16,17]. The commercial leptospiral whole-cell-based vaccines contain four, six, or eight serovars for canine, swine, and bovine vaccines, respectively [12,18,19]. However, these multivalent whole-cell leptospiral vaccines usually do not achieve sufficient coverage against the spectrum of serovars important for animal health [15]. Although several research groups recently presented a novel approach for the preparation of whole-cell leptospiral vaccines with cross-protective immunity, the disadvantages of the bacterin vaccine are still a concern [20,21]. Adverse effects of the whole-cell vaccines are contaminated medium components resulting in serious side effects, the requirement for ongoing surveillance to identify new bacterin serovars for the preparation of multivalent whole-cell vaccines, and the maintenance of virulent strains in bacterin formulations [14,15,22].

Given the limitations of whole-cell vaccines currently in use, the identification of suitable protein candidates has emerged as a major task for vaccine development [15]. Several recombinant protein vaccines for leptospirosis, including LipL32, LipL41, LigA, LigB, FcpA, and Ompl1 proteins, have been studied and found a wide range of immune responses [15,23,24,25,26,27,28,29,30,31,32,33]. However, the application of these results is complicated by numerous modifications to the recombinant proteins. The greatest challenge for vaccine development against leptospirosis is the prediction of antigens that provide sterilizing immunity with long-lasting responses [15]. Recent advances in reverse vaccinology (RV) could represent a promising approach to identifying leptospiral vaccine candidates and for the urgently required development of improved recombinant leptospiral vaccines [14]. 

Proteins containing leucine-rich repeats (LRRs) have been predicted and reported to function in bacterial host-pathogen interactions, membrane anchoring, invasion, and stimulation of host defense mechanisms (Kobe and Kajava, 2001; McDonald et al., 2003; Seepersaud et al., 2005; Ye et al., 2009). It is interesting to concentrate on the pathogenic *Leptospira* LRR proteins as immunogens. Pathogenic *Leptospira* LRRs are can compete with the functions of the host to adhere and invade host cells such as LRR20 [34,35]. The role of rLRR20 in leptospirosis revealed that rLRR20 was observed to colocalize with E-cadherin on the cell surface and activate the downstream transcription factor, beta-catenin, which subsequently promoted the expression of MMP7, a kidney injury biomarker [34,35]. Recently, some bioinformatics studies revealed that the pathogenic *Leptospira* strains possess more leucine-rich repeat (LRR) genes than the saprophytic strains [36,37]. Moreover, Nitipan et al. reported the presence of seven pairs of the conserved LRR genes in the serovar Hardjo-bovis strain JB197 by the analysis of the *L. borgpetersenii* genome [37]. Identifying immunogenic epitopes in LRR proteins is interesting.

The immunogenic epitopes were identified by in silico studies from two leptospiral LRR proteins, the rKU_Sej_LRR_2012M and rKU_Sej_LRR_2271 proteins, from *L. borgpetersenii* serogroup Sejroe genome [37,38,39]. In addition, the two identified LRR proteins have cross-reactive immunity with rabbit hyperimmune sera against *Leptospira* serovars Icterohaemorrhagiae, Javanica, serogroup Sejroe, and serovars Bratislava, Icterohaemorrhagiae, and serogroup Sejroe, respectively [38,40].

Further evaluation of leptospiral LRR proteins, the rKU_Sej_LRR_2012M and the rKU_Sej_LRR_2271 proteins from *L. borgpetersenii* serogroup Sejroe, was performed as a first step to the vaccine development in cattle. The rKU_Sej_LRR_2012M and rKU_Sej_LRR_2271 proteins were chosen because they were cloned from *L. borgpetersenii* serogroup Sejroe, which is the most prevalent leptospiral serogroup in bovine livestock in Thailand and South-East Asia. It is important to emphasize that strains from the Sejroe serogroup are host-adapted to bovine, leading to a chronic and silent disease affecting the reproductive tract of cows, recognized as Bovine Genital Leptospirosis (BGL) [9]. Even though a cross-protective immunity against the acute disease caused by *L. interrogans* serovar Pomona was observed, stimulated by LRR proteins cloned from *L. borgpetersenii* serogroup Sejroe, the most prevalent leptospiral serovar in bovine livestock in Thailand and South-East Asia. This study investigates the LRR proteins as potent vaccine candidates for protecting cattle leptospirosis by evaluating cross-protective immunity in the hamster model challenged with *L. interrogans* serovar Pomona. 

## 2. Materials and Methods

### 2.1. Bacteria Strains and Culture

*L. interrogans* serovar Pomona (NVSL 11000-HL145A) was obtained from the National Veterinary Service Laboratories (NVSL), Ames, Iowa. Leptospires were maintained in the EMJH medium at 30 °C. Low passage cultures were isolated from infected hamsters with a sublethal dose of *L. interrogans* serovar Pomona, as previously described [23].

### 2.2. Expression and Purification of LRR Proteins

Two LRR proteins, the rKU_Sej_LRR_2012M and rhKU_Sej_LRR_2271 proteins, were expressed from pET161_hKU_R21M_2012 and pET160_hKU_R21_2271 plasmids in *Escherichia coli* BL21 Star^TM^ (DE3) expression systems, respectively [8,38,39] with a minor modification. Both proteins were expressed as 6xHis-Lumio-TEV fusion proteins. The proteins were purified by immobilized metal ion affinity chromatography (IMAC) with Protino^®^ Ni-TED Resin (Macherey-Nagel, Dueren, Germany) as previously described [38]. The 6xHis-Lumio-TEV was cleaved with TEV protease (NEB#P8112) after the purification step as per the manufacturer’s protocol. The protein concentration was determined using a Nanodrop spectrophotometer (Thermo Scientific, Waltham, MA, USA). The cleaved and purified protein was analyzed by 12% SDS-PAGE and Coomassie blue staining. The proteins were stored at −20 °C until used for immunization, stimulating splenocytes in vitro and ELISA assay.

### 2.3. Preparation of Antigen-Adjuvant Mixtures for Immunization 

Each purified protein was adjusted to a final concentration of 1 µg/µL with PBS. The aluminum hydroxide wet gel suspension (Alhydrogel^®^ adjuvant 2%, InvivoGen, San Diego, CA, USA) was used as an adjuvant at which the final volume ratio of Alhydrogel^®^ adjuvant 2% to the protein antigen was 1:1 for the antigen-adjuvant mixture preparation. The capped bottle of Alhydrogel^®^ adjuvant 2% was shaken well before use, and 50 µL of Alhydrogel^®^ adjuvant 2% was added to each tube containing 50 µL of PBS, 50 µg of rKU_Sej_LRR_2012M, and 50 µg of rhKU_Sej_LRR_2271 proteins for the preparation of control, 2012, and 2271 vaccines, respectively. The tube contents were mixed well by pipetting up and down for at least 5 min to allow Alhydrogel^®^ adjuvant 2% to effectively adsorb the antigen. The final volume of each antigen-adjuvant mixture was 100 µL. Every step for vaccine preparation was performed under aseptic techniques. The antigen-adjuvant mixtures or vaccines were immediately prepared before immunizations.

### 2.4. Animal Model for Acute Leptospirosis, Immunization Protocols, and Lethal Leptospiral Challenge

#### 2.4.1. The Animal Model for Acute Leptospirosis

The hamster was used for acute leptospirosis and the lethal leptospiral infection model. Female Golden Syrian hamsters (Harlan Sprague Dawley) 3–4 weeks old were used in this study. Male hamsters, raised in the same cage, tend to fight each other after 2 months of age. Male hamsters were also reported to present more severe symptoms in acute leptospirosis with no translation into a differential humoral response since no significant difference in IgG response was measured between male and female hamsters [41]. Therefore, the authors decided to use female hamsters in the experiments for animal welfare reasons. The animals had ad libitum access to commercial pelleted ration and drinking water. Animals in each experimental group were raised and allowed to run free in each cage, as previously described [42]. Experiments were conducted according to the protocols approved by the IACUC (Institutional Animal Care and Use Committee) at Cornell University and Kasetsart University Institutional Animal Care and Use Committee (Kasetsart University-IACUC).

#### 2.4.2. Immunization Protocols

Golden Syrian hamsters (3–4 weeks old) were divided into 3 and 2 groups of 8 and 13 animals for experiments 1 and 2, respectively. The immunization protocol was performed as previously described [30,42] with a minor modification. Before immunization, the animals were anesthetized by intraperitoneal injection of 100 µL of ketamine (10 mg/mL)/xylazine (1 mg/mL) per 130 g of body weight. Animals were immunized at 3-week intervals with a total of two injections for each animal.

Three experimental groups in experiment 1 consisted of the control (*n* = 8), 2012 (*n* = 8), and 2271 (*n* = 8) groups in which animals from each group were immunized subcutaneously with 100 µL of control, 2012, and 2271 antigen-adjuvant mixtures (vaccines), respectively. The antigen-adjuvant mixtures or vaccines were immediately prepared before immunizations by adjusting the 50 µg of purified protein to a final concentration of 1 µg/µL with PBS. Then, the aluminum hydroxide wet gel suspension (Alhydrogel^®^ adjuvant 2%, InvivoGen, San Diego, CA, USA) was used as an adjuvant at which the final volume ratio of Alhydrogel^®^ adjuvant 2% to the protein antigen was 1:1 for the antigen-adjuvant mixture preparation.

Animals in experiment 2 were comprised of control (*n* = 13) and 2271 (*n* = 13) groups, and animals were subcutaneously vaccinated with 100 µL of control and 2271 antigen-adjuvant mixtures (vaccines), respectively. Five hamsters from each treatment group were sacrificed 3 weeks after the second immunization, but before the challenge, to evaluate the lymphoproliferation and the cytokine production. The remaining animals, control (*n* = 8) and 2271 immunized (*n* = 8) hamsters, were challenged with highly virulent *L. interrogans* serovar Pomona on day 42 (3 weeks after the second immunization) and were monitored for 21 days post-challenge.

The hamsters were bled through the saphenous vein on day 0 (pre-immunization, PI), day 21 (3 weeks after the 1st immunization, AI), day 42 (3 weeks after the 2nd immunization or after 1st boost, AB), and day 49–63 (7–21 days after the challenge, AC). Sera were collected and kept at −80 °C until use.

#### 2.4.3. Challenge

Hamsters were challenged intraperitoneally on day 42 (3 weeks after the 2nd immunization) with *Leptospira* prepared as described in the previous work [30,42,43]. Briefly, 2.5 × 10^2^ (2.5× modified LD50 [MLD50]) of a single passage *L. interrogans* serovar Pomona (NVSL 11000-HL145A) in 1 mL sterile PBS was intraperitoneally injected into each animal (*n* = 8 for each experimental group). Clinical signs and mortality were monitored and recorded twice daily for 3 weeks. Hamsters with severe clinical signs of moribund [44], including loss of appetite, presenting gait or breathing difficulty, prostration, ruffled fur, dehydration and weight loss of ≥10% of the animal’s maximum weight, or signs of bleeding, or seizure, were euthanized after blood collection and counted as dead. The euthanized animals’ tissues, including lung, liver, kidney, and urinary bladder, were collected aseptically for histopathology examination and leptospiral culture and quantification. Hamsters that survived the challenge were sacrificed at the end of the observation period and bled by the cardiac puncture. Tissue samples, including lung, liver, kidney, and urinary bladder, were collected with an aseptic technique for histopathology examination and leptospiral culture and quantification.

### 2.5. Evaluation of Specific Humoral Immune Responses by ELISA

Antigen-specific IgG against rKU_Sej_LRR_2012M and rhKU_Sej_LRR_2271 proteins from the 2012 and 2271 immunized and control groups were evaluated by using an Enzyme-Linked Immuno Sorbent Assay (ELISA) as described previously [8,30,42] with some modifications. In brief, 5 µg/mL of the recombinant LRR protein in carbonate-bicarbonate buffer (CBB) was coated on polystyrene microplates (NuncMaxiSorp, Thermo Fisher Scientific) (100 µL/well) and incubated at room temperature for 2 h. After washing three times with 1× PBS supplemented with tween (PBST), plates were blocked with 200 µL blocking buffer (2% BSA in PBST) at 37 °C in a humid chamber for 1 h. Then, 100 µL of hamster serum (control and vaccinated) at dilution of 1:500 with PBST or PBST (as background or blank) was added and incubated at 37 °C for 1 h. Next, 100 µL of 2.7% hydrogen peroxide was added and incubated for 5 min. After the washing step, the 100 µL of HRP-conjugated goat anti-hamster IgG in dilution of 1:5000 (KPL, MD) was added to each well and incubated at 37 °C for 1 h. After washing, 100 µL of tetramethyl benzidine (TMB) substrate solution (KPL, MD) was added and incubated in the dark for 5 min. Absorbance was measured by an ELISA reader (Biotek Instruments Inc., Winooski, VT, USA) at 630 nm. The average absorbance of backgrounds or blanks (PBST) was subtracted from each sample absorbance. All experimental samples were tested in triplicate, and data are presented as mean ± S.E.M. (Standard Error of the Mean). The level of specific IgG was compared between the immunized and nonimmunized groups using the Kruskal-Wallis one-way ANOVA analysis.

### 2.6. Serum Bactericidal Assay

To determine whether serum collected from the animals immunized with LRR proteins had in vitro bactericidal activity, serum from each animal in experiment 1 (*n* = 8 per each treatment) at 3 weeks after the 2nd immunization but before the challenge was individually evaluated by serum bactericidal assay (SBA) as previously described [43]. Briefly, 10^8^ cells/mL of the low passage highly virulent *L. interrogans* serovar Pomona were prepared in PBS buffer containing 2 mM MgCl_2_ and 1 mM CaCl_2_ and then mixed with serum plus 25% of normal human serum (ImmunoReagents) as a complement source. The mixtures were incubated at 37 °C, and the viability of the bacteria was assessed using dark-field microscopy at different time points (0, 30, 60, 90, and 120 min) of incubation. The survival rate of *Leptospira* was calculated as the number of motile (alive) cells in every 100 counts performed by two researchers blinded to the treatment. The mean value was calculated from two independent measurements as a single replicate. Results are shown as mean ± S.E.M. from three trials of two replicates.

### 2.7. Lymphoproliferation Assay in Hamsters Splenocytes

Five animals from each treatment group in experiment 2 were sacrificed 3 weeks after the 2nd immunization but before the challenge. The spleens were removed aseptically for splenocyte preparation as per the protocol described previously, and the lymphoproliferation assay in response to an evoke antigen was performed as described previously [45] with some modifications. Briefly, splenocytes were seeded in flat-bottomed 96-well microtiter plates at a concentration of 5 × 10^5^ cells in 200 µL of cRPMI and stimulated with an individual antigen including the rhKU_Sej_LRR_2271 protein or the potent epitope peptides (either LL17 or SL19 peptides, [8] at a concentration of 10 µg/mL for 48 h at 37 °C, 5% CO_2_. Splenocytes stimulated with concanavalin A (ConA) (10 µg/mL; Sigma-Aldrich, Merck Ltd., Bangkok, Thailand) or medium were only considered positive and negative controls. Each experiment was performed in triplicate. 

The splenocyte DNA synthesis in each treatment was measured using the Click-iT EdU Proliferation Assay for Microplates (Invitrogen, Thermo Fisher Scientific) as the manufacturer’s protocol. Briefly, a nucleoside analog EdU (5-ethynyl-2′-deoxyuridine) was incorporated into cellular DNA during the active DNA synthesis. The incorporated EdU’s alkyne group is then joined covalently to an azide group present on HRP (horse-radish peroxidase) by using the click chemistry provided with the Click-iT EdU proliferation assay for microplates. The highly fluorescent Amplex Ultrared product produced from an Amplex Ultrared HRP substrate was measured on a microplate reader (Synergy HTX Multi-Mode Reader; Biotex Instruments, Winooski, VT, USA) using filter sets for excitation at 568 nM and emission at 585 nM. The results were expressed as stimulation indices (SI) and calculated as the ratios between cells cultured with either an antigen or ConA and the mean fluorescence intensity of cells cultured in medium only.

### 2.8. Evaluation of Splenocytes Cytokines Gene Expression by Quantitative Real-Time Reverse Transcription-Polymerase Chain Reaction (qRT-PCR)

The technique qRT-PCR was performed to assess the mRNA expression of the Th1 and Th2 cytokines, IFNg, IL-12, IL-4, and IL-10, in hamster spleens. Spleen samples were collected from control and immunized groups (*n* = 5 for each group in experiment 2) which were sacrificed at 3 weeks after the 2nd immunization but before a challenge. Total RNA samples were isolated from splenic tissues, and splenocytes were stimulated in vitro with or without antigen (rhKU_Sej_LRR_2271 protein, LL17, and SL19 at 10 µg/mL for each antigen). The total RNA was isolated using Tri-reagent (Sigma-Aldrich) as the manufacturer’s protocol. The total RNA concentration was quantified using a Nanodrop spectrophotometer (Thermo Scientific). The 1 µg of purified total RNA was reverse transcribed for cDNA synthesis using the SuperScript ™ III First-Strand Synthesis SuperMix for qRT-PCR (Invitrogen, Thermo Scientific, Waltham, MA, USA). The qRT-PCR was prepared using an iTaq Universal SYBR Green Supermix (Bio-Rad Laboratories, BKK, Thailand) and CFX96 Touch real-time PCR detection system (Bio-Rad Laboratories, BKK, Thailand) as per the manufacturer’s protocol, with primers as described previously [46]. Quantitation was performed using the comparative cycle threshold (CT) method and reported as relative transcription or the *n*-fold difference relative to the housekeeping gene hypoxanthine phosphoribosyl transferase (HPRT).

### 2.9. Quantification of Leptospira Load in Tissues by Quantitative Real-Time PCR

Liver, kidney, and urinary bladder were aseptically removed from the infected animal (*n* = 8 per each treatment group in experiment 2) that died after the challenge and from the animals that survived the challenge through the end of the observation period. The same organs from the uninfected hamsters were also collected and used as controls for standard curve preparation. All tissue samples were kept at −80 °C until total RNA was isolated and purified from each 200 mg tissue sample using an RNeasy mini kit (Qiagen, Hilden, Germany) following the manufacturer’s protocol with on-column DNase digestion. The total RNA concentration was quantified using a Nanodrop spectrophotometer (Thermo Scientific). The 1 µg of purified total RNA was reverse transcribed for cDNA synthesis using the SuperScript™ III First-Strand Synthesis SuperMix (Invitrogen, CA). The reverse transcribed cDNA was subsequently quantified the *Leptospira*-specific gene, LipL32, by qRT-PCR using TaqMan PCR protocol and primers set as follows: LipL32-45F (5′-AAG CAT TAC CGC TTG TGG TG-3′) and LipL32-286R (5′-GAA CTC CCA TTT CAG CGA TT-3′), and primer-probe LipL32-189P (FAM-5′-AA AGC CAG GAC AAG CGC CG-3′-BHQ1) as the protocol described previously [47]. Reactions of 20 µL consisted of 1 × TaqMan^®^ Gene Expression Master Mix (Applied Biosystems, Merck Ltd., Bangkok, Thailand), 400 nM of each primer, and 200 nM probe with 5 µL of cDNA sample. Amplification and fluorescence detection were performed using the 7500 Fast Real-Time PCR (Applied Biosystems) platform following standard conditions described elsewhere [47]. All reactions were duplicated with negative and positive controls for each run. The data were analyzed using the 7500 Fast Real-Time PCR software (Applied Biosystems).

The standard curve was prepared by spiking 1 × 10^1^–1 × 10^6^ leptospires into each 200 mg of uninfected hamsters’ tissue sample prior to RNA isolation, followed by cDNA synthesis and qRT-PCR to quantify the expression of the LipL32 gene in spiked samples. A standard curve was plotted with copy numbers of *Leptospira* versus threshold cycle (CT) for each tissue. The number of *Leptospira* in each sample was analyzed by comparing the CT value of the standard curve for each various tissue against the copy numbers in each standard spiked curve.

### 2.10. Culture

To conclusively determine the presence of *Leptospira*, the liver, kidney, and urinary bladder from infected animals were also submitted for culture in EMJH medium and maintained at 30 °C for 4 weeks. The growth of leptospires was monitored using dark-field microscopy. The culture samples that presented with visible leptospires were counted as positive, and those without visible leptospires were counted as negative.

### 2.11. Histopathology

Histopathological analysis was performed as previously described [30,42]. Briefly, tissues collected from animals in experiment 2 (*n* = 8 per group) were fixed by immersion in 10% neutral buffered formalin. Fixed tissues were sectioned at 5 µm, stained with hematoxylin and eosin, and examined by light microscopy. The severity of *Leptospira*-induced lesions in various organs was graded by a board-certificated veterinary pathologist who was blinded to the treatment group. Tubulointerstitial nephritis was categorized as follows: 0 = normal, 1 = mild, 2 = moderate, and 3 = severe, using criteria previously described [23,30]. The extent of pulmonary hemorrhage was graded as 0 = none, 1 = single focus, 2 = multiple foci, and 3 = extensive areas of hemorrhage. The liver lesion was graded based on the average number of inflammatory foci in 10 × 10 fields selected randomly as follows: 0 = normal, 1 = 1–3 foci, 2 = 4–7 foci, and 3 = >7 foci.

### 2.12. Statistical Analysis

The statistical analyses were performed using the Log-rank test to compare mortality and survival rate between control and immunized groups for the independent experiments (experiments 1 and 2). The significant difference was determined at a *p*-value < 0.05 for the comparison between each immunization group and the control group in both experiments. The Mann-Whitney U test was used for the leptospiral load in tissues, histopathology, and culture results. The Kruskal-Wallis one-way ANOVA followed by all pairwise comparisons was used to establish significant differences in groups with a non-normal distribution. A *p*-value of <0.05 was considered statistically significant. The statistical program PASW (SPSS) was used to perform analyses. Microsoft Excel 2019 was used to create graphs.

## 3. Results

### 3.1. Stimulation of Specific Humoral Immune Responses by Letospiral LRR Proteins in the Immunized Hamsters

**Figure 1:**
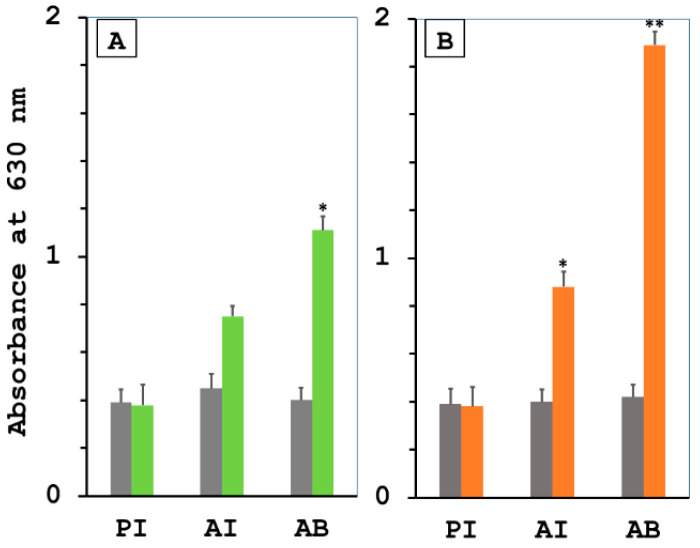
Induction of the specific humoral immune responses in hamsters immunized with LRR proteins, either the rKU_Sej_LRR_2012M or the rhKU_Sej_LRR_2271. (**A**) rKU_Sej_LRR_2012M-specific IgG and (**B**) rhKU_Sej_LRR_2271-specific IgG were evaluated in experiment 1 by ELISA. The level of specific IgG production shown on the Y-axis was compared between the control (gray bar) and the immunized hamsters with each protein (green bar: rKU_Sej_LRR_2012M; orange bar: rhKU_Sej_LRR_2271). Sera from the control and immunized hamsters were collected on day 0 (pre-immunization, PI), day 21 (3 weeks after the 1st immunization, AI), day 42 (3 weeks after the 2nd immunization or after 1st boost, AB) as shown on X-axis. The data represent the mean of eight hamsters per treatment group ± S.E.M. The * and ** indicate significant differences in *p*-values < 0.05 and <0.01, respectively.

Antigen-specific IgG against rKU_Sej_LRR_2012M and rhKU_Sej_LRR_2271 proteins in the 2012 or 2271 immunized and control groups in experiment 1 were assessed by using an ELISA. Before and after immunization with the LRR proteins at days 21 and 42, the levels of specific IgG from the immunized and control groups were determined. The animals immunized with either rKU_Sej_LRR_2012M or rhKU_Sej_LRR_2271 proteins had significantly greater levels of the LRR protein-specific IgG in sera when compared to the control group at day 21 (3 weeks after the first immunization, AI) for only the 2271 group, and day 42 (3 weeks after the second immunization, AB) for both 2012 and 2271 groups (Figure 1A,B). In addition, the rhKU_Sej_LRR_2271 specific antibody levels were increased sharply 3 weeks after the first and the second immunization (AI and AB) with the 2271 vaccine compared with the sera from the control group with *p*-values < 0.05 and <0.01, respectively (Figure 1B). The increased level of specific IgG from the hamsters immunized with both LRR proteins was saturated at day 42 (3 weeks after the second immunization, AB), and this level of specific IgG was detected until 21 days after the challenge (AC), which was the end of observation at day 63 as shown in Supplementary Figure S1A,B. The results revealed that the hamsters immunized with the 2271 protein produced more significant amounts of the specific IgG than in the control and 2012 groups.

### 3.2. Bactericidal Activity of Sera from LRR Proteins Immunized Hamsters

**Figure 2:**
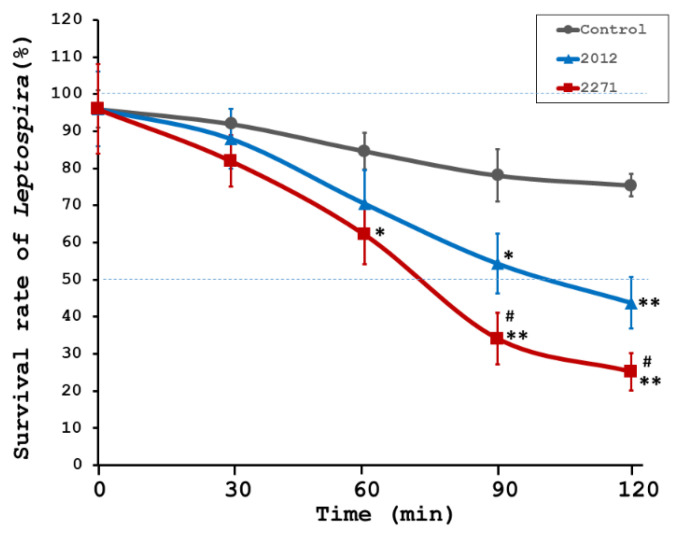
**Anti-LRR protein bactericidal proficiency.** The serum bactericidal assays to measure *Leptospira* survival rates were used to assess the in vitro bactericidal efficiency of sera bled from each animal in experiment 1 (*n* = 8 per each treatment) 3 weeks after the 2nd immunization before the challenge. Hamster sera from control (gray line), either rhKU_Sej_LRR_2012 (2012; blue line) or rhKU_Sej_LRR_2271 (2271; red line) immunized animals (*n* = 8 per group in experiment 1) were incubated with low passage, highly virulent *L. interrogans* serovar Pomona at 37 °C, and the viability of the bacteria was observed using dark-field microscopy at different time points (0, 30 60, 90 and 120 min) of incubation. Each value represents the mean ± S.E.M. Data were compared between animals immunized with 2012 protein, 2271 protein, and control groups with statistically significant differences at a *p*-value < 0.05 (*) and <0.01 (**), respectively, and were compared between 2012 and 2271 vaccinated groups as a *p*-value < 0.05 (#).

To assess the role of sera from the immunized hamsters showing high levels of specific IgG against either rKU_Sej_LRR_2012M or rhKU_Sej_LRR_2271 proteins in promoting the complement-mediated killing of *Leptospira* in vitro, the bactericidal assays were performed. Sera were collected from the control (*n* = 8) and immunized (*n* = 8 per each vaccine) animals at day 42 or 3 weeks after the second immunization but before the challenge. Sera from 2271 immunized hamsters exhibited significant killing activity starting from 60 min incubation until the end of the incubation period compared to sera from the control hamsters (Figure 2). Although sera from hamsters immunized with 2012 protein showed minor killing activity, the level of activity was not as intense as for sera from hamsters immunized with 2271 protein at 90–120 min of incubation at a *p*-value < 0.05 (# in Figure 2). Sera from the control group showed the poorest bactericidal activity, at a *p*-value < 0.05 (*) and <0.01 (**), as shown in Figure 2.

### 3.3. Protective Efficacy of LRR Proteins against Challenging Virulent L. interrogans Serovar Pomona in Hamsters

**Figure 3:**
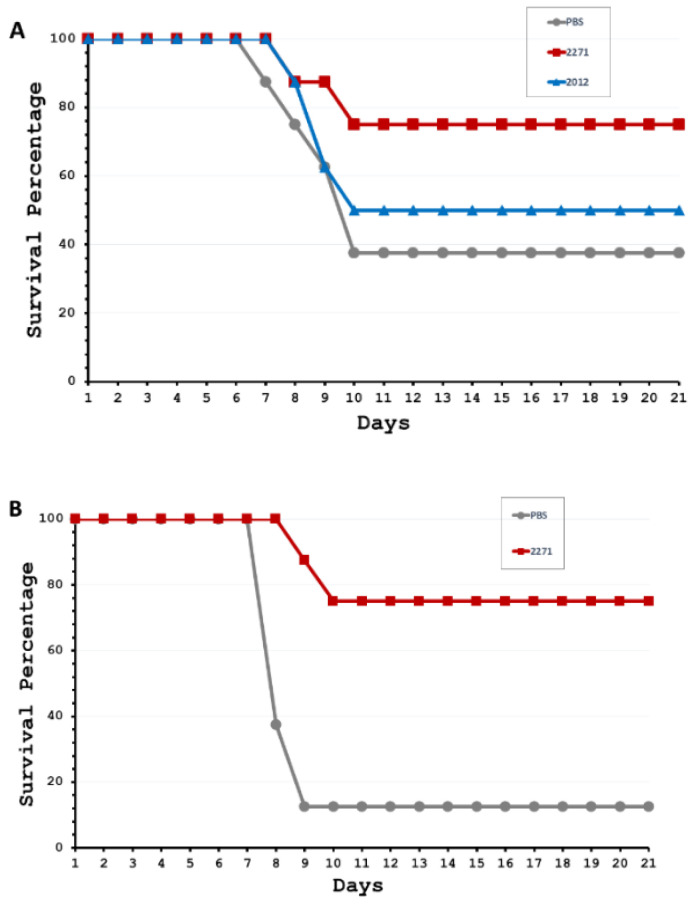
Survival of hamsters immunized with LRR proteins in two individual experiments ((**A**); three experimental groups in experiment 1) and ((**B**); two experimental groups in experiment 2) after challenge with the virulent *L. interrogans* serovar Pomona. The animals immunized with the rhKU_Sej_LRR_2012 (2012; blue line), the rhKU_Sej_LRR_2271 (2271; red line) proteins, and PBS as control (PBS; gray line) were challenged intraperitoneally with 2.5 × 10^2^ leptospires (2.5× modified LD50 [MLD50]) of a single passage of *L. interrogans* serovar Pomona (NVSL 11000-HL145A) in 1 mL of sterile PBS on 3 weeks after the 2nd immunization. The hamsters were monitored for mortality till day 21 post-challenge.

Since both LRR proteins can stimulate humoral immune responses and have bactericidal activity, albeit to different degrees, the protective efficacy of both proteins was evaluated. Experiment 1 was designed to evaluate whether each LRR protein could protect animals against lethal leptospiral infection. All hamsters (*n* = 8 per group) in three groups of experiment 1 were challenged with highly virulent *L. interrogans* serovar Pomona on day 42 (3 weeks after the second immunization). The survival data on the 21st day post-infection showed survival rates of 37.5%, 50.0%, and 75.0% for the control, 2012, and 2271 groups, respectively (Figure 3A). Overall, there was a significantly higher survival rate of animals that were only vaccinated with the rhKU_Sej_LRR_2271 (2271 group). Therefore, experiment 2 was designed to assess in greater depth the potential of the rhKU_Sej_LRR_2271 protein as a potential leptospiral vaccine candidate. Survival data in experiment 2 showed that challenged hamsters in the 2271 group (*n* = 8) had a 75% survival rate on the 21st day post-infection, while the survival rate of animals in the control group was only 12.5% (Figure 3B). Overall, the hamsters immunized with the rhKU_Sej_LRR_2271 protein (2271 group) showed a significant survival rate compared to the control group at a *p*-value < 0.05 for both experiments.

### 3.4. Role of the 2271 Protein as a Future Leptospiral Vaccine Candidate

Considering the rhKU_Sej_LRR_2271 protein (the 2271 immunogen) as a future leptospiral vaccine candidate, experiment 2 was conducted to validate 2271 protective actions by promoting protective immune systems on both humoral and cellular immunes responses, and by the tissue bacterial clearance and tissue inflammatory responses. Control (*n* = 13) and 2271 vaccinated (*n* = 13) hamsters were immunized as stated in the immunization protocol. The stimulating immune systems actions by the 2271 antigen were investigated on humoral and cellular immune responses in five hamsters from each treatment group. Hamsters were sacrificed 3 weeks after the second immunization but before the challenge. The lymphoproliferation and the cytokines production were evaluated. 

The remaining animals, control (*n* = 8) and 2271 vaccinated (*n* = 8) hamsters, were monitored for 21 days post-challenge with highly virulent *L. interrogans* serovar Pomona on day 42 (3 weeks after the second immunization). The tissue bacterial clearance and tissue inflammatory responses of challenged hamsters were evaluated.

#### 3.4.1. The 2271 Actions on Stimulating the Lymphoproliferation and the Cytokines Productions

The rhKU_Sej_LRR_2271 protein showed high humoral immune responses in 2271 vaccinated hamsters. The rhKU_Sej_LRR_2271 protein was previously reported to contain epitopes predicted to be recognized by both major histocompatibility complex (MHC) class I, class II, and T cell receptors and to contain promising target epitopes to stimulate humoral and cell-mediated immunity in rabbits [8]. A rising amount of specific IgG could indicate the activation of both humoral and cell cell-mediated immunity in sera, the enhancement of lymphoproliferation, and the upregulation of Th1 and Th2 type cytokines. Therefore, the 2271 immunity on stimulating the lymphoproliferation and the cytokines production was evaluated.

**Figure 4:**
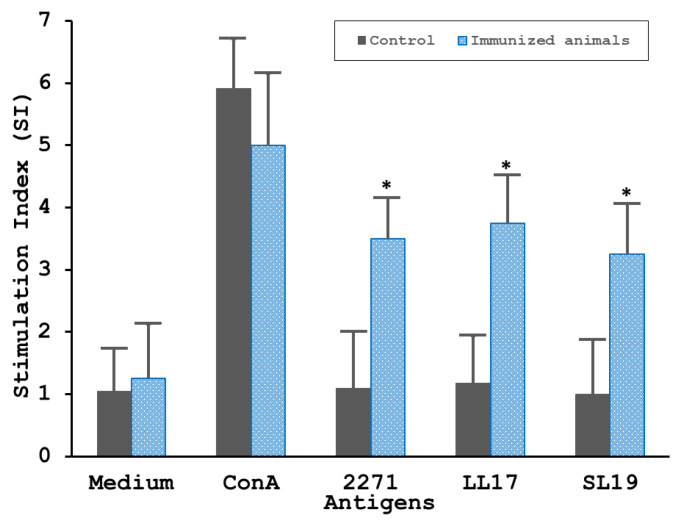
**The proliferation of splenocytes in response to the rhKU_Sej_LRR_2271 protein**. Splenocytes isolated from the spleens of control hamsters (*n* = 5) and hamsters immunized with 2271 protein (*n* = 5) 3 weeks after the 2nd immunization before a challenge were cultured in flat-bottomed 96-well microtiter plates. The cells were stimulated with 10 µg/mL of an individual antigen, including the rhKU_Sej_LRR_2271 protein, LL17, and SL19 peptides, as described in Section 2.7. Splenocytes stimulated with ConA or medium were considered positive and negative controls, respectively. The proliferative capacity was deliberated using the Click-iT EdU Proliferation Assay for Microplates (Invitrogen, Thermo Fisher Scientific) as per the manufacturer’s protocol. The data represents mean ± S.E.M., and the * represents the statistically significant differences (*p*-value < 0.05) determined using the Mann-Whitney U test.

As shown in Figure 4, splenocytes obtained 3 weeks after the 2nd immunization from 2271 immunized hamsters showed the proliferative activity considerably in response to the stimulation by either the 2271 immunogen or potent epitopes peptides, LL17 and SL19 (Figure 4). The splenocytes isolated from control animals that were immunized with PBS-alum failed to respond to any antigen except for ConA (positive control). Splenocyte proliferation was significant in response to ConA (positive control) but failed to respond to the medium alone (negative) in both negative control and immunized animals.

**Figure 5:**
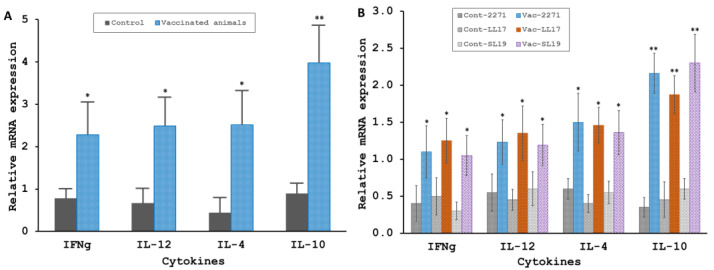
**Evaluation of cytokine gene expression by qRT-PCR.** Cytokine response to the 2271 protein was assessed by the determination of Th1 and Th2 cytokines mRNA expression relative to HPRT mRNA expression from the whole spleen (**A**) or splenocytes stimulated in vitro with antigen (**B**) 3 weeks after the 2nd immunization before a challenge from Control (*n* = 5) and immunized (*n* = 5) animals. The cells in (**B**) were stimulated with 10 µg/mL of an individual antigen, including the rhKU_Sej_LRR_2271 (2271) protein, LL17, and SL19 peptides as described in Section 2.8. The data represent mean ± S.E.M., and the * and ** represent statistically significant differences of *p*-values < 0.05 and <0.01, respectively, determined by using the Mann-Whitney U test for (**A**) and the Kruskal-Wallis one-way ANOVA analysis for (**B**).

The induction of both Th1 (IFNg, IL12) and Th2 (IL4, IL10) type cytokines gene expression in whole spleen and splenocytes stimulated by antigen was evaluated in vitro by qRT-PCR. The relative cytokines mRNA expression in the whole spleen from vaccinated (*n* = 5) hamsters exhibited a higher level of approximately 2, 2.5, 2.5, and 4 folds compared with the expression level from the control (*n* = 5) animals for investigated cytokines IFNg, IL-12, IL-4, and IL-10, respectively (Figure 5A). The most significant difference was seen for IL10 gene expression, with a four times increase in the immunized group and a *p*-value < 0.01. Further investigation of splenocyte’s cytokine response to in vitro antigen stimulation revealed a significant boost of 3–4 fold of the mRNA level for IL10 (Figure 5B). In addition, as shown in Figure 5B, all other observed cytokines, including IFNg, IL12, and IL4, from immunized animals displayed significantly enhanced levels of expression for the stimulation of each antigen, including the 2271 protein, LL17, and SL19 peptides (*p*-value < 0.05). No significant increase in mRNA levels was observed for the Th1 and Th2 cytokines for the control animals (Figure 5B).

#### 3.4.2. Tissues Bacterial Clearance and Tissues Inflammatory Responses of the 2271 Immunized Hamsters Challenged with *L. interrogans* Serovar Pomona

**Figure 6:**
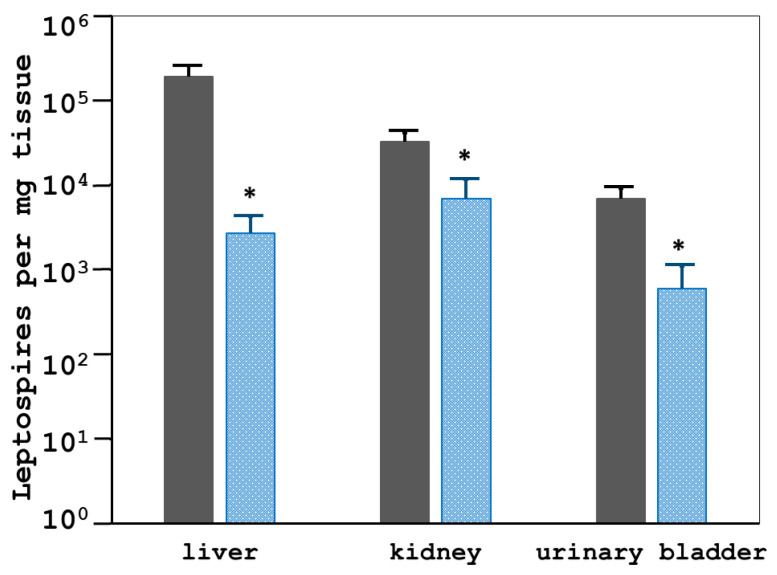
The effect of the 2271 immunization on the leptospiral load in tissues from the control (*n* = 8; dark gray bar) and immunized (*n* = 8; blue bar) animals after the challenge from experiment 2. The total RNA from each tissue was isolated and reverse-transcribed to cDNA. The cDNA of a specific *Leptospira* gene, LipL32, was quantified by qRT-PCR. The number of *Leptospira* in the sample was analyzed by comparing the CT value of the standard curve for each various tissue with the copy numbers in each standard spiked curve. The data represent the log scale of the mean bacterial loads in ±S.E.M. The * indicates the significant differences with a *p*-value < 0.05.

The 2271 antigen successfully reduced lethality in leptospiral-infected hamsters. The 2271 immunogen also promoted better clearance of *leptospires* from animal tissues, including the liver, kidney, and urinary bladder, as shown in Figure 6. The control and vaccinated animals demonstrated statistically significant differences in bacterial load (number of *leptospires* per mg tissue) in all evaluated tissues (*p*-value < 0.05) (Figure 6). The average number of *leptospires* in control tissues was 10 to 100-fold higher than that of vaccinated animals by qRT-PCR (Figure 6) and bacterial culture results (Table 1). Table 1 shows the conclusive determination of the presence of *Leptospira* cultured from tissues including the liver, kidney, and urinary bladder from control animals were significantly higher than that of the immunized hamsters. However, the kidneys from two of eight 2271 immunized hamsters that survived on day 21 post-challenge were found positive for *Leptospira* culture.

**Figure 7:**
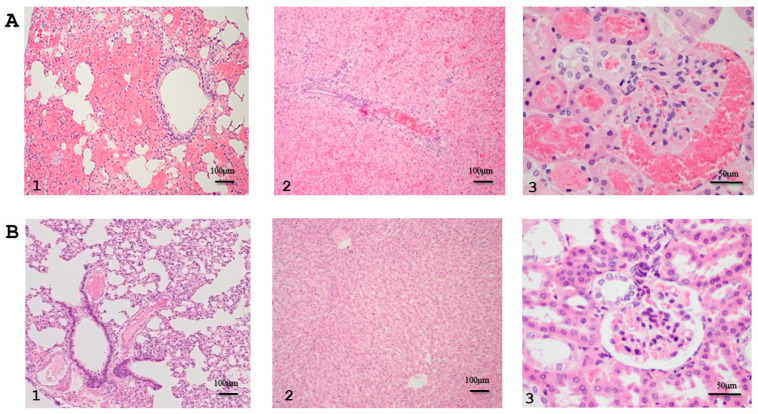
Histopathological analysis of lung (1), liver (2), and kidney (3) from the control (**A**) and animals immunized with 2271 protein (**B**) after the challenge from experiment 2.

Histopathological studies further assessed the efficacy of rhKU_Sej_LRR_2271 as a prophylactic immunogen. Data were evaluated in terms of inflammatory lesions in the lung, liver, and kidney of the challenged hamsters in experiment 2. Histopathologic examination of various organs demonstrated moderate to severe pulmonary lesions with multifocal hemorrhage, hepatitis with a high number of inflammatory foci, and hemorrhagic tubulointerstitial nephritis in control animals, Figure 7A (1–3). Tissues from the 2271-immunized animals had less severe lesions within the normal limits compared with control hamster tissues, Figure 7B (1–3). Table 2 shows the prophylactic efficacy of the rhKU_Sej_LRR_2271 protein evaluated based on histopathological lesion scores in various organs from challenged animals. Although there were no statistically significant differences with a *p*-value < 0.05 on the tissue histopathological lesion scores comparing the control and immunized animals, the severe pathological scores on inflammatory lesions in the lung, liver, and kidney from the immunized hamsters were lower than those from the control animals in Table 2.

## 4. Discussion

The greatest challenge for the development of a vaccine against leptospirosis is to identify antigens that provide long-lasting, cross-protective, and sterilizing immunity. To develop a fully protective, sterilizing immune response to a leptospiral vaccine, mixed strong humoral and cell-mediated immune responses are obligatory [14,15,16,20,29,30,45,48,49,50]. Since *Leptospira* is an extracellular bacterium, the predominant immunological effector response is humoral immunity, whereby IgG antibodies would inactivate the *Leptospira* because of complement-mediated lysis and/or via opsonization for phagocytosis. 

Although *Leptospira* strains from serogroup Sejroe are host-adapted to bovine, leading to a chronic and silent disease affecting the reproductive tract of cows, recognized as Bovine Genital Leptospirosis (BGL) [9], the incidence of BGL in Thailand is underestimated and no precise data have been reported in Thailand [51]. On the other hand, Pomona is incidental in ruminants and associated with an acute disease, whereas the serogroup Pomona serovar Pomona was frequently reported seropositive in cattle, buffaloes, pigs, and dogs in Thailand [10,11]. In addition, the serovar Pomona had been reported to cause acute and severe leptospirosis in cattle by incidental infection [12,13], especially in multi-host ecologically systems as in Thai rural agriculture areas [41]. Therefore, the hamster model for acute leptospirosis against heterologous *L. interrogans* serovar Pomona was set to evaluate vaccine candidate proficiencies of two LRR proteins cloned from *L. borgpetersenii* serogroup Sejroe.

Proteins containing leucine-rich repeats (LRRs) have been predicted and reported to function in bacterial host-pathogen interactions, membrane anchoring, and invasions, such as proteins Internalin A, B and J, YopM, and LRR20 [34,52,53,54,55,56,57,58,59]. LRR proteins containing the LPXTG motif, a cell wall anchoring motif [LPXTG cell wall anchor domain (IPR019931), https://www.ebi.ac.uk/interpro/entry/InterPro/IPR019931/ (accessed on 1 Febuary 2019)], have been reported as virulence factor such as internalin J (InlJ) [55]. Leucine-rich repeats of bacterial surface proteins also serve as common pattern recognition motifs of host cell receptors, as reported in human scavenger receptor gp340 by LrrG and E-cadherin by rLRR20 [34,60]. Both rKU_Sej_LRR_2012M and rhKU_Sej_LRR_2271 proteins contain the LPXAG motif, and the two leptospiral LRR proteins characterized in this study had been reported to exhibit rapid induction of specific humoral immune responses in immunized rabbits as well as in hamsters of this report [8,37,38,39,40]. Although this report did not investigate the virulence of rKU_Sej_LRR_2012M and rhKU_Sej_LRR_2271 proteins in pathogenic *Leptospira* infection, it has been reported that the LRR domain-containing protein family is vital for the virulence of pathogenic *Leptospira* species [34,35]. Therefore, two leptospiral LRR proteins investigated in this report are of interest as candidates for the development of a leptospirosis vaccine.

Although both LRR proteins characterized in this report exhibited rapid induction of specific humoral immune responses in immunized hamsters, only the rhKU_Sej_LRR_2271 protein induced antibody production 3 weeks after the first and the second immunization in hamsters. The high levels of IgG production against the rhKU_Sej_LRR_2271 protein from 2271 immunized hamsters also prominently promoted the complement-mediated killing of *Leptospires* per bactericidal assays. The strength of bactericidal activity exhibited by sera from 2012-vaccinated animals was less intense than the action exposed by sera from the 2271 immunized hamsters. In addition, the 2012 vaccines demonstrated only 50% protective efficacy against challenging virulent *L. interrogans* serovar Pomona in hamsters. The rKU_Sej_LRR_2012M protein showed poor proficiency as a leptospiral vaccine candidate under the challenging condition with virulent *L. interrogans* serovar Pomona. However, this report has not been performed and challenged with different pathogenic serovars and serogroups and awaits further studies.

The rKU_Sej_LRR_2012M (2012) protein was produced from two overlapping LRR genes of *L. borgpetersenii* serogroup Sejroe, the *KU_Sej_R21N_2012* (NCBI accession: JN627491.1) and *KU_Sej_R21C_2012* (NCBI accession: JN627492.1) genes to produce the *KU_Sej_R21_2012M gene* with a deletion at A346 of the gene “*KU_Sej_R21_2012* (NCBI accession: JN627495)” [39]. The gene “*KU_Sej_R21_2012*” from *L. borgpetersenii* serogroup Sejroe genome is an orthologous gene of the *LBJ_2012* gene of *L. borgpetersenii* serovar Hardjo-bovis str. JB197 [40]. Since the BLAST results showed no significant similarity found for the alignment between the “*KU_Sej_R21_2012* (NCBI accession: JN627495)” gene and *L. interrogans* serovar Pomona (taxid:44276), and only two genomes from *L. interrogans* serovar Bataviae strain 1489 and serovar Canicola strain 782 showed 74.53% identity with the *KU_Sej_R21_2012* gene (Supplementary Data S1); therefore, the 2012 protein provided poor cross-protective efficacy against challenging with virulent *L. interrogans* serovar Pomona in this report. In addition, Sripattanakul et al. reported recently that the rKU_Sej_LRR_2012M (2012) protein could be detected by rabbit hyperimmune sera against *L. borgpetersenii* serovar Canicola, Mini, and Tarassovi in both line-blot and ELISA, but the 2012 LRR protein could not be detected by rabbit hyperimmune sera against *L. interrogans* serovar Pomona by both techniques [11]. It implied a poor cross-immunity between serogroup Sejroe and serovar Pomona induced by the 2012 protein. Therefore, further investigation on the 2012 potential as a vaccine candidate against *L. borgpetersenii, L. mayottensis, L. weilii, L. santarosai, and L. interrogans* serovar Bataviae and serovar Canicola could present a commendable task.

Although the rhKU_Sej_LRR_2271 protein promoted intense humoral immune response and sera bactericidal action from 2271 immunized hamsters, only 75% protective efficacy in immunized hamsters against challenging with heterogeneous virulent strain *L. interrogans* serovar Pomona was attained. In addition, sterilizing immunity was not achieved. This could be explained by the result of a BLAST search of the *KU_R21_2271 gene* (NCBI accession: JX522460), which yielded no significant similarity for the alignment with the genome of *L. interrogans* serovar Pomona (taxid:44276), whereas the *KU_R21_2271* sequence similarities were 75.58% to 78.19% identity with 75 genes from other *L. interrogans strains* (Supplementary Data S2), and 98.04% to 99.84% identity to 36 sequences from *L. borgpetersenii* genomes (Supplementary Data S3). The BLAST data imply better prospects for the 2271 protein as a vaccine candidate against either *L. borgpetersenii* or *L. interrogans* serovars other than serovar Pomona, as the results of not fully cross-protection was achieved by the induction of the 2271 protein. The fully 100% cross-protective against the heterogeneous strain of serovar Pomona was not attended from the 2271 antigen; nevertheless, the sera from 2271 immunized hamsters provided great bactericidal action.

The intense humoral immune response and bactericidal ability of the 2271 antigen are in concordance with previous results from Tansiri et al., having demonstrated that the 2271 protein contains promiscuous T-cell epitopes, which were in silico computationally proposed to have potential binding both MHC class I and II alleles and successfully forming the pMHC/TCR complex [8]. The rhKU_Sej_LRR_2271 protein promiscuous T-cell epitopes, LL17:171-LLFLPLIKILYVDRNKL-187 and SL19:209SLNSGIKALPFNYEKLVNL-227, which can bind to over three of MHC alleles, significantly increased interferon-gamma (IFNγ)-producing specific T-cell responses in the rhKU_Sej_LRR_2271 immunized rabbits compared to nonimmunized rabbits. The LL17 peptide can induce interferon-gamma-producing specific CD4+ T-cell responses in immunized rabbits [8]. Tansiri et al. showed that the 2271 protein induced both humoral and cell-mediated immune responses in vaccinated rabbits. However, the mechanisms by which 2271 confers protective immunity against *leptospires* in both humoral and cell-mediated immune responses in acute leptospirosis have to be further investigated.

It is interesting to note that the rhKU_Sej_LRR_2271 protein can activate both Th1 and Th2 immune responses as a single protein. It has been previously reported that cell-mediated immunity is required for protection against bovine leptospirosis [16,48,50] and in a hamster model of acute leptospirosis [20,29,30,45,49,61]. The results in the present study are in agreement with the data reported by Tansiri et al. The 2271 antigen, including LL17 and SL19 peptides, stimulated splenocyte proliferative responses on cultured splenocytes isolated from the 2271 vaccinated hamster spleens. It was suggested that vaccines’ induction of lymphoproliferative responses indicates protective immunity through CMI responses [29,30,45]. The 2271 and its derived peptides in vitro stimulating Th1 cells were observed by IFNγ and IL-12 cytokine gene expressions in the whole spleen and the splenocytes of vaccinated hamsters. Although the IgG isotype levels against each antigen were not analyzed in this report, the Th1 immune response, which is thought to be responsible for protection against leptospirosis, has been observed in related studies [45,49,50,62,63]. These results strongly suggest that the 2271 vaccine elicited protective immunity through CMI actions.

Since the 2271-immunized hamsters demonstrated elevated levels of specific IgG against the rhKU_Sej_LRR_2271 protein, it is conceivable that the solid humoral immune responses by the vaccine result in increased mRNA profiles of IL-4 and IL-10. This would clearly suggest that the 2271 vaccine was capable of protecting hamsters against experimental leptospiral infection by activating strong humoral immunity and significant accomplishments of CMI. However, the 2271 antigen could not confer 100% protection from lethality as small numbers of *leptospires* were detected by qRT-PCR in tissues from some vaccinated hamsters. Nevertheless, the 2271 vaccine could reduce severe inflammatory lesions in immunized hamsters, although no statistically significant differences were observed at a *p*-value < 0.05. Immunized hamsters had 10 to 100-fold fewer *leptospires* in the liver, kidney, and urinary bladder when compared to unvaccinated hamsters. The lower number of *leptospires* in organs could be related to less severe tissue inflammation in immunized animals. The reduced number of *leptospires* in organs points to a possible control of the proliferation of *leptospires* after infection. Although the 2271 protein is supposed to function as bacterial host-pathogen interactions, membrane anchoring, and invasion, such as proteins Internalin A, B and J, YopM, and LRR20 [34,52,53,54,55,56,57,58,59], the 2271 vaccine could not provide a sterilizing immunity against challenging with virulent *L. interrogans* serovar Pomona in immunized hamsters. However, the protein alleviated severe inflammatory lesions in vital tissues; therefore, it is interesting to investigate further the 2271 vaccine activity against homologous *Leptospira* such as *L. borgpetersenii* and additional cross-immunity against heterologous strains from *L. interrogans* as previously studied [20,21,64].

Although the immunized hamsters had reduced leptospiral colonization in the liver, kidney, and urinary bladder, as seen in bacterial culture and qPCR leptospiral quantification results, renal colonization was observed in the 2271 immunized hamsters that survived on 21 days post-challenge. Therefore, the purified recombinant KU_Sej_LRR_2271 protein alone is insufficient as a subunit vaccine against *L. interrogans* serovar Pomona. Further studies to combine with other antigens such as LigA, LigB, LipL32, and/or with leptosome–entrapped, PC-liposome entrapped antigens, PLGA microsphere, DNA, chimeric BCG vaccine delivery system, [23,26,27,28,29,30,45,49,50,65]. As previously reported, a similar technology may be applicable to construct a 2271- mutant similar to the fcpA- mutant [20,21].

## 5. Conclusions

In summary, our study provides evidence that the 2271 protein provided humoral immune responses, inducing in vitro sera bactericidal actions, high protective efficacies, promoting *Leptospira* tissue clearances, reducing tissue inflammation, and stimulating CMI. Therefore, it seems reasonable to suggest that the recombinant KU_Sej_LRR_2271 protein could be a candidate for future modifications leading to an improved vaccine against leptospirosis in bovine, especially homologous protective immunity against BGL, heterologous cross-protective immunity against acute incidental bovine leptospirosis.

## 6. Patents

The patent announcement #1701001602 was advertised on 4 October 2018 regarding the expression of LRR proteins, the rKU_Sej_LRR_2012M and rhKU_Sej_LRR_2271 proteins, in *Escherichia coli* BL21 Star^TM^ (DE3) expression systems.

## Figures and Tables

**Table 1:** The conclusive determination of the presence of *Leptospira* cultured from the liver, kidney, and urinary bladder from the Control (*n* = 8) and immunized (*n* = 8) animals after the challenge. The liver, kidney, and urinary bladder from the infected animals were cultured in EMJH medium and maintained at 30 °C for 4 weeks. The growth of leptospires was monitored using dark-field microscopy. The visible f leptospires in culture samples were counted as positive, and those without visible leptospires were counted as negative. The * indicates the significant differences with a *p*-value < 0.05.

Culture Results (Score)	Liver		Kidney		Urinary Bladder	
	PBS	2271	PBS	2271	PBS	2271
Positive (+1)	7	2	8	2 (#)	7	1
Negative (0)	1	6	0	6	1	7
Average score	0.875	0.250	1.000	0.250	0.875	0.125
Statistical differences	*		*		*	

#: The 2271 immunized hamsters that survived on day 21 post-challenge.

**Table 2:** The prophylactic efficacy of the rhKU_Sej_LRR_2271 protein was evaluated based on histopathological lesion scores in various organs from challenged animals. Tissues from eight hamsters in each group were individually harvested and fixed. A board-certified veterinary pathologist graded the lesions in the lung, liver, and kidney of hamsters from control (PBS) and immunized (2271) groups on a scale of severity. Lung tissues were graded for severity of hemorrhage (0 = normal, 1 = focal, 2 = multifocal, 3 = extensive areas of hemorrhage). Liver tissues were graded for the number of inflammatory foci (0 = normal, 1 = 1–3, 2 = 4–7, 3 = >7). Kidney tissues were graded for severity of renal lesions (0 = normal, 1 = mild, 2 = moderate, 3 = severe tubulointerstitial nephritis). The difference between results from each tissue for the control and immunized hamsters is indicated by *p*-values.

Score	Lung		Liver		Kidney	
	PBS	2271	PBS	2271	PBS	2271
0	1	3	0	1	0	3
1	2	3	2	3	6	3
2	5	1	0	1	2	1
3	0	1	6	3	0	1
Average score	1.50	1.00	2.50	1.75	1.25	1.00
*p*-value	0.12		0.17		0.09	

## Data Availability

The datasets generated during and/or analyzed during the current study are available from the corresponding author upon request.

## Supplementary Materials

The following supporting information can be downloaded at: https://www.mdpi.com/article/10.3390/tropicalmed8010006/s1, Supplementary Figure S1: Induction of specific humoral immune responses in hamsters immunized with LRR proteins; Supplementary Data S1: The alignment between “*KU_Sej_R21_2012* (NCBI accession: JN627495)” gene and *L. interrogans* serovar Pomona (taxid:44276), and only two genomes from *L. interrogans* serovar Bataviae strain 1489 (orange color) and serovar Canicola strain 782 (green color) showed 74.53% identity with the *KU_Sej_R21_2012* gene; Supplementary Data S2: BLAST search of the *KU_R21_2271 gene* (NCBI accession: JX522460) yielded no significant similarity for the alignment with the genome of *L. interrogans* serovar Pomona (taxid:44276). Blast search of the *KU_R21_2271* sequence similarities were 75.58% to 78.19% identity with 75 genes from other *L. interrogans strains*; Supplementary Data S3: BLAST search of the *KU_R21_2271 gene* (NCBI accession: JX522460) yielded 98.04% to 99.84% identity to 36 sequences from *L. borgpetersenii* genomes.
