# Supplementary material for: Leptospira borgpetersenii Leucine-Rich Repeat Proteins Provide Strong Protective Efficacy as Novel Leptospiral Vaccine Candidates
Source: Trop Med Infect Dis. 2022 Dec 22;8(1):6. doi: 10.3390/tropicalmed8010006 (PMC9863753; doi:10.3390/tropicalmed8010006)
Supplement: Supplementary file 1 [file tropicalmed-08-00006-s001.zip › Supplementary data S1.pdf]

Supplementary data S1. The alignment between "*KU_Sej_R21_2012* (NCBI accession: JN627495)" gene and *L. interrogans* serovar Pomona (taxid:44276), and only two genomes from *L. interrogans* serovar Bataviae strain 1489 (orange color) and serovar Canicola strain 782 (green color) showed 74.53% identity with the *KU_Sej_R21_2012* gene.

Your search is limited to records that include: Leptospira interrogans (taxid:173), Leptospira interrogans serovar Pomona (taxid:44276), Leptospira interrogans serovar Pomona str. UT364 (taxid:1049925), Leptospira interrogans serovar Pomona str. Kennewicki LC82-25 (taxid:1049924), Leptospira interrogans serovar Pomona str. CSL4002 (taxid:1240691), Leptospira interrogans serovar Pomona str. Pomona (taxid:1001587), Leptospira interrogans serovar Pomona str. Fox 32256 (taxid:1218574), Leptospira interrogans serovar Pomona str. CSL10083 (taxid:1240692), Leptospira kirschneri serovar Pomona (taxid:561005), Leptospira borgpetersenii serovar Pomona (taxid:1192865), Leptospira borgpetersenii serovar Pomona str. 200901868 (taxid:1192866)

RID: DTK9YZ64013
Job Title:JN627495:Leptospira borgpetersenii serovar...
Program: BLASTN
Query: Leptospira borgpetersenii serovar Sejroe clone R21-2012 truncated leucine-rich repeat protein (LRR) gene, partial cds ID: JN627495.1(nucleic acid) Length: 643
Database: nt Nucleotide collection (nt)

Sequences producing significant alignments:

Description	Scientific Name	Common Name	Taxid	Max Score	Total Score	Query cover	E Value	Per. Ident	Acc. Len	Accession
Leptospira borgpetersenii serovar Sejroe clone R21-2012...	Leptospira b...	NA	338220	1118	1118	100%	0.0	100.00	643	JN627495.1
Leptospira borgpetersenii serovar Ceylonica strain Piyasena...	Leptospira b...	NA	508536	1083	1083	100%	0.0	98.60	3670231	CP026671.1
Leptospira borgpetersenii strain FMAS_AP2 chromosome 1, comple...	Leptospira b...	NA	174	1083	1083	100%	0.0	98.60	3595436	CP072630.1
Leptospira borgpetersenii strain FMAS_AP3 chromosome 1, comple...	Leptospira b...	NA	174	1083	1083	100%	0.0	98.60	3595898	CP072628.1
Leptospira borgpetersenii strain FMAS_AP4 chromosome 1, comple...	Leptospira b...	NA	174	1083	1083	100%	0.0	98.60	3595848	CP072626.1
Leptospira borgpetersenii strain FMAS_PN1 chromosome 1, comple...	Leptospira b...	NA	174	1083	1083	100%	0.0	98.60	3596207	CP072624.1
Leptospira borgpetersenii strain FMAS_PN4 chromosome 1, comple...	Leptospira b...	NA	174	1083	1083	100%	0.0	98.60	3595796	CP072622.1
Leptospira borgpetersenii strain FMAS_AP8 chromosome 1, comple...	Leptospira b...	NA	174	1083	1083	100%	0.0	98.60	3595870	CP072620.1
Leptospira borgpetersenii strain FMAS_AP9 chromosome 1, comple...	Leptospira b...	NA	174	1083	1083	100%	0.0	98.60	3595582	CP072618.1
Leptospira borgpetersenii strain R14-L chromosome 1	Leptospira b...	NA	174	1028	1028	100%	0.0	96.42	3581013	CP047516.1

Leptospira borgpetersenii strain R6L chromosome 1	Leptospira b...	NA	174	1028	1028	100%	0.0	96.42	3580531	CP047520.1
Leptospira borgpetersenii strain R14 chromosome 1	Leptospira b...	NA	174	1028	1028	100%	0.0	96.42	3601002	CP047504.1
Leptospira borgpetersenii strain R6 chromosome 1	Leptospira b...	NA	174	1028	1028	100%	0.0	96.42	3513910	CP047372.1
Leptospira borgpetersenii strain Mo4 chromosome 1	Leptospira b...	NA	174	1028	1028	100%	0.0	96.42	3581767	CP047334.1
Leptospira borgpetersenii strain R28 chromosome 1	Leptospira b...	NA	174	1028	1028	100%	0.0	96.42	3580481	CP047332.1
Leptospira borgpetersenii strain R23 chromosome 1	Leptospira b...	NA	174	1028	1028	100%	0.0	96.42	3579904	CP047370.1
Leptospira borgpetersenii strain R29 chromosome 1	Leptospira b...	NA	174	1028	1028	100%	0.0	96.42	3580756	CP047330.1
Leptospira borgpetersenii str. 4E chromosome 1	Leptospira b...	NA	1141102	1028	1028	100%	0.0	96.42	3550837	CP015814.2
Leptospira borgpetersenii serovar Ballum strain 56604 chromoso...	Leptospira b...	NA	280505	1028	1028	100%	0.0	96.42	3550837	CP012029.1
Leptospira borgpetersenii strain 34-PK chromosome 1, complete...	Leptospira b...	NA	174	1017	1017	100%	0.0	95.96	3603305	CP097464.1
Leptospira borgpetersenii serovar Hardjo-bovis strain L49...	Leptospira b...	NA	338217	892	977	100%	0.0	91.60	3620710	CP033440.1
Leptospira borgpetersenii serovar Hardjo-bovis strain 203...	Leptospira b...	NA	338217	892	977	100%	0.0	91.60	3589981	CP021412.1
Leptospira borgpetersenii serovar Hardjo strain NVSL S 818...	Leptospira b...	NA	328971	892	977	100%	0.0	91.60	3584464	CP015052.1
Leptospira borgpetersenii serovar Hardjo strain NVSL S 1343...	Leptospira b...	NA	328971	892	977	100%	0.0	91.60	3613654	CP015050.1
Leptospira borgpetersenii serovar Hardjo strain BK-30 chromoso...	Leptospira b...	NA	328971	892	977	100%	0.0	91.60	3629289	CP015048.1
Leptospira borgpetersenii serovar Hardjo strain BK-9 chromosome 1	Leptospira b...	NA	328971	892	977	100%	0.0	91.60	3631634	CP015046.1
Leptospira borgpetersenii serovar Hardjo strain BK-6 chromosome 1	Leptospira b...	NA	328971	892	977	100%	0.0	91.60	3649879	CP015044.1
Leptospira borgpetersenii strain KR39 chromosome 1, complete...	Leptospira b...	NA	174	892	977	100%	0.0	91.60	3603773	CP096127.1
Leptospira borgpetersenii serovar Hardjo strain TC129 chromoso...	Leptospira b...	NA	328971	892	977	100%	0.0	91.60	3585597	CP084038.1
Leptospira borgpetersenii strain TC147 chromosome I, complete...	Leptospira b...	NA	174	892	977	100%	0.0	91.60	3585599	CP084040.1
Leptospira borgpetersenii strain TC273 chromosome I, complete...	Leptospira b...	NA	174	892	977	100%	0.0	91.60	3585577	CP084042.1
Leptospira borgpetersenii strain TC112 chromosome I, complete...	Leptospira b...	NA	174	892	977	100%	0.0	91.60	3585599	CP084036.1
Leptospira borgpetersenii serovar Hardjo-bovis JB197 chromosom...	Leptospira b...	NA	355277	892	977	100%	0.0	91.60	3576473	CP000350.1

Leptospira borgpetersenii serovar Hardjo-bovis L550 chromosome... Leptospira b... NA 355276 892 977
100% 0.0 91.60 3614446 CP000348.1
Leptospira mayottensis strain MDI272 chromosome I, complete... Leptospira m... NA 1137606 757 757
100% 0.0 87.42 3861885 CP030147.1
Leptospira mayottensis 200901116 chromosome I, complete sequence Leptospira m... NA 1192864 757 757
100% 0.0 87.42 3815263 CP024871.1
Leptospira weilii strain CUD13 chromosome I, complete sequence Leptospira w... NA 28184 679
1121 100% 0.0 83.18 3978120 CP040843.1
Leptospira weilii strain CUD06 chromosome I, complete sequence Leptospira w... NA 28184 679
1121 100% 0.0 83.18 3965053 CP040840.1
Leptospira weilii strain FMAS_PD2 chromosome 1, complete sequence Leptospira w... NA 28184 679
1121 100% 0.0 83.18 4222777 CP092203.1
Leptospira mayottensis strain MDI222 chromosome I Leptospira m... NA 1137606 606 606
100% 1e-168 82.58 3934431 CP030144.1
Leptospira mayottensis strain VS2413 chromosome I, complete... Leptospira m... NA 1137606 602 602
100% 2e-167 82.43 3803942 CP030142.1
Leptospira borgpetersenii serovar Sejroe clone R21-N2012... Leptospira b... NA 338220 601 601
53% 6e-167 100.00 350 JN627491.1
Leptospira borgpetersenii serovar Ballum clone R3-N2012... Leptospira b... NA 280505 585 585
53% 3e-162 98.84 350 JN627493.1
Leptospira borgpetersenii serovar Javanica clone R12-N2012... Leptospira b... NA 280504 561 561
53% 3e-155 97.10 350 JN627494.1
Leptospira borgpetersenii serovar Sejroe clone R21-C2012... Leptospira b... NA 338220 517 517
46% 7e-142 100.00 297 JN627492.1
Leptospira interrogans serovar Bataviae strain 1489 chromosome... Leptospira i... NA 312175 454 454
99% 6e-123 74.53 4431091 CP043893.1
Leptospira interrogans serovar Canicola strain 782 chromosome ... Leptospira i... NA 211880 454 454
99% 6e-123 74.53 4556406 CP043884.1
Leptospira weilii strain FMAS_RT1 chromosome 1, complete sequence Leptospira w... NA 28184 442 442
100% 3e-119 73.99 4275568 CP092206.2
Leptospira borgpetersenii serovar Mini clone R15-N2012 truncat... Leptospira b... NA 280507 434 434
53% 9e-117 88.12 349 JN627496.1
Leptospira santarosai strain U233 chromosome I Leptospira s... NA 28183 171 171
21% 1e-37 87.50 3605957 CP028377.1
Leptospira santarosai strain U164 chromosome I Leptospira s... NA 28183 171 171
21% 1e-37 87.50 3730310 CP028370.1
Leptospira santarosai strain ah2 chromosome I sequence Leptospira s... NA 28183 171 171
21% 1e-37 87.50 3545388 CP022887.1
Leptospira santarosai strain U160 chromosome I Leptospira s... NA 28183 151 151
21% 9e-32 83.82 3768600 CP027843.1
Leptospira santarosai serovar Shermani str. LT 821 chromosome ... Leptospira s... NA 758847 151 151
21% 9e-32 83.82 3659905 CP006694.199% 2e-126 74.53 4556406 CP043884.1

```
Alignments:

>Leptospira interrogans serovar Bataviae strain 1489 chromosome 1, complete sequence
Sequence ID: CP043893.1 Length: 4431091
Range 1: 1058849 to 1059488

Score:454 bits(1043), Expect:2e-126,
Identities:480/644(75%),  Gaps:7/644(1%), Strand: Plus/Plus

Query  1        CAGGGAAAAAATGAATGGTGGAAATATCTATTCTGGATTTTGGACGAAAAAGATTCTGCA  60
                || | |||  | |||||||||||||||||| ||||| ||||| |||||||  ||||| |
Sbjct  1058849  CAAGAAAAGCAAGAATGGTGGAAATATCTAGTCTGGCTTTTGAACGAAAAGCATTCTTCG  1058908

Query  61       ACTGAATCCAACA---TAGATTCTCTTTCGGAAGACGGAGTTTTGTCCGCTCCGCCTTCC  117
                ||| || | |  |   || ||| |||| ||   |||||  || || | |||||||||||
Sbjct  1058909  ACTCAACCTAGGAATTTATATTATCTTCCGAGCGACGGGATTCTGGCGGCTCCGCCTTCG  1058968

Query  118      GATAAAAAGTTGAGCCAAGGTACCGAAGTAAGATTCGATTGGTTAACTTCCTTAATAGAG  177
                ||| || | |||||||||| |||||| |||||||||||| |||| ||||| ||    |||
Sbjct  1058969  GATGAACAATTGAGCCAAGCTACCGAGGTAAGATTCGATGGGTTGACTTCTTTGGCGGAG  1059028

Query  178      GTTCCGTTGGGTAAAATGCCGACTTTGGATACTTTGGATCTATATCCTCGAGAAGGAAAA  237
                ||| |||| || |||||| ||||| | ||||  |||    |    ||  |  ||| |||
Sbjct  1059029  GTTACGTTAGGCAAAATGTCGACTCTCGATAAATTGACGGTGCTCCCCGGCAAAGAAAAG  1059088

Query  238      AATGCTTCTAAACTTTCTTCTTTGGACGGAATTGAACGAGCGTCCGGATTAATTAAATTG  297
                || ||| ||||||||||||| ||||| ||||| ||||||||| |  | |||   | ||||
Sbjct  1059089  AAAGCTCCTAAACTTTCTTCCTTGGATGGAATCGAACGAGCGCCTTGGTTAGCCAGATTG  1059148

Query  298      AATGTGGAGAGAAATCAAGGCATATCGGATCTCGGTCTTTTGTCAAAAACTTCCTAATTT  357
                 |  ||   || |  |||| | | ||| || | |  ||||| || ||| ||||| |||||
Sbjct  1059149  GACATG---AGCATGCAAGACGTCTCGAATATAGACCTTTTATCGAAA-CTTCCGAATTT  1059204

Query  358      GAAAACTTTTAGCGGATCGAATAACTCGATTAAGGATTTGTCTCCACTTTCTCAGTGCAA  417
                 |||  | ||  ||||||| |||||||||| || ||| | ||||| ||||||||||||
Sbjct  1059205  AAAAGTTCTTCACGGATCGTATAACTCGATCAAAGATCTATCTCCGCTTTCTCAGTGCCG  1059264

Query  418      AAATTTAAATGCGCTTTATCTCAATAAAAATAAAATCTCCGATGTTTCCCCGCTTTCTTC  477
                ||| ||| |    ||||||||| | ||||||||||| ||||| || || || ||||||||
Sbjct  1059265  AAACTTAGAAATACTTTATCTCGACAAAAATAAAATTTCCGACGTCTCTCCCCTTTCTTC  1059324

Query  478      ACTTTCAAAAATAGAAACTCTTTGCTTAGCCGACAATCCGATCCAGGATATTCTTCCTTT  537
                 ||||   |||||||||| |||||  || |||||||||||||| ||||||||||||||||
Sbjct  1059325  GCTTTTGGAAATAGAAACGCTTTGGATATCCGACAATCCGATCAAGGATATTCTTCCTTT  1059384

Query  538      GGCCGGTTTaaaaaaaTTAAAAGAATTGAAAGTGCCGTCGAAACTCCCCGAAGAAAATTT  597
                  | ||||| || ||| | |||||| ||||| | |||  ||| ||  |  |||| |||||
Sbjct  1059385  AACGGGTTTGAAGAAACTGAAAGAACTGAAAATACCGGTGAAGCTTTCGAAAGAGAATTT  1059444

Query  598      AGCGAAGTTTAAGAAATTACGCCCCGATGTGAAAATCTCATTTT  641
                ||   |||| || ||| |||| ||||||||||| || || ||||
Sbjct  1059445  AGAACAGTTCAAAAAACTACGTCCCGATGTGAAGATTTCTTTTT  1059488
```

```
>Leptospira interrogans serovar Canicola strain 782 chromosome 1, complete sequence
Sequence ID: CP043884.1 Length: 4556406
Range 1: 3551479 to 3552118

Score:454 bits(1043), Expect:2e-126,
Identities:480/644(75%),  Gaps:7/644(1%), Strand: Plus/Minus

Query  1        CAGGGAAAAAATGAATGGTGGAAATATCTATTCTGGATTTTGGACGAAAAAGATTCTGCA  60
                || | |||  | |||||||||||||||||| ||||| ||||| |||||||  ||||| |
Sbjct  3552118  CAAGAAAAGCAAGAATGGTGGAAATATCTAGTCTGGCTTTTGAACGAAAAGCATTCTTCG  3552059

Query  61       ACTGAATCCAACA---TAGATTCTCTTTCGGAAGACGGAGTTTTGTCCGCTCCGCCTTCC  117
                ||| || | |  |   || ||| |||| ||   |||||  || || | ||||||||||||
Sbjct  3552058  ACTCAACCTAGGAATTTATATTATCTTCCGAGCGACGGGATTCTGGCGGCTCCGCCTTCG  3551999

Query  118      GATAAAAAGTTGAGCCAAGGTACCGAAGTAAGATTCGATTGGTTAACTTCCTTAATAGAG  177
                ||| || | |||||||||| |||||| |||||||||||| |||| ||||| ||    |||
Sbjct  3551998  GATGAACAATTGAGCCAAGCTACCGAGGTAAGATTCGATGGGTTGACTTCTTTGGCGGAG  3551939

Query  178      GTTCCGTTGGGTAAAATGCCGACTTTGGATACTTTGGATCTATATCCTCGAGAAGGAAAA  237
                ||| |||| || |||||| ||||| | ||||  |||    |    ||  |  ||| |||
Sbjct  3551938  GTTACGTTAGGCAAAATGTCGACTCTCGATAAATTGACGGTGCTCCCCGGCAAAGAAAAG  3551879

Query  238      AATGCTTCTAAACTTTCTTCTTTGGACGGAATTGAACGAGCGTCCGGATTAATTAAATTG  297
                || ||| ||||||||||||| ||||| ||||| ||||||||| |  | |||   | ||||
Sbjct  3551878  AAAGCTCCTAAACTTTCTTCCTTGGATGGAATCGAACGAGCGCCTTGGTTAGCCAGATTG  3551819

Query  298      AATGTGGAGAGAAATCAAGGCATATCGGATCTCGGTCTTTTGTCAAAAACTTCCTAATTT  357
                 |  ||   || |  |||| | | ||| || | |  ||||| || ||| ||||| |||||
Sbjct  3551818  GACATG---AGCATGCAAGACGTCTCGAATATAGACCTTTTATCGAAA-CTTCCGAATTT  3551763

Query  358      GAAAACTTTTAGCGGATCGAATAACTCGATTAAGGATTTGTCTCCACTTTCTCAGTGCAA  417
                 |||  | ||  ||||||| |||||||||| || ||| | ||||| ||||||||||||
Sbjct  3551762  AAAAGTTCTTCACGGATCGTATAACTCGATCAAAGATCTATCTCCGCTTTCTCAGTGCCG  3551703

Query  418      AAATTTAAATGCGCTTTATCTCAATAAAAATAAAATCTCCGATGTTTCCCCGCTTTCTTC  477
                ||| ||| |    ||||||||| | ||||||||||| ||||| || || || ||||||||
Sbjct  3551702  AAACTTAGAAATACTTTATCTCGACAAAAATAAAATTTCCGACGTCTCTCCCCTTTCTTC  3551643

Query  478      ACTTTCAAAAATAGAAACTCTTTGCTTAGCCGACAATCCGATCCAGGATATTCTTCCTTT  537
                 ||||   |||||||||| |||||  || |||||||||||||| ||||||||||||||||
Sbjct  3551642  GCTTTTGGAAATAGAAACGCTTTGGATATCCGACAATCCGATCAAGGATATTCTTCCTTT  3551583

Query  538      GGCCGGTTTaaaaaaaTTAAAAGAATTGAAAGTGCCGTCGAAACTCCCCGAAGAAAATTT  597
                  | ||||| || ||| | |||||| ||||| | |||  ||| ||  |  |||| |||||
Sbjct  3551582  AACGGGTTTGAAGAAACTGAAAGAACTGAAAATACCGGTGAAGCTTTCGAAAGAGAATTT  3551523

Query  598      AGCGAAGTTTAAGAAATTACGCCCCGATGTGAAAATCTCATTTT  641
                ||   |||| || ||| |||| ||||||||||| || || ||||
Sbjct  3551522  AGAACAGTTCAAAAAACTACGTCCCGATGTGAAGATTTCTTTTT  3551479
```
