# Supplementary material for: Leptospira borgpetersenii Leucine-Rich Repeat Proteins Provide Strong Protective Efficacy as Novel Leptospiral Vaccine Candidates
Source: Trop Med Infect Dis. 2022 Dec 22;8(1):6. doi: 10.3390/tropicalmed8010006 (PMC9863753; doi:10.3390/tropicalmed8010006)
Supplement: Supplementary file 1 [file tropicalmed-08-00006-s001.zip › Supplementary data S2.pdf]

Supplementary data S2. BLAST search of the *KU_R21_2271 gene* (NCBI accession: JX522460) yielded no significant similarity for the alignment with the genome of *L. interrogans* serovar Pomona (taxid:44276). Blast search of the *KU_R21_2271* sequence similarities were 75.58% to 78.19% identity with 75 genes from other *L. interrogans strains.*

Your search is limited to records that include: Leptospira interrogans (taxid:173), Leptospira interrogans serovar Pomona (taxid:44276), Leptospira interrogans serovar Pomona str. UT364 (taxid:1049925), Leptospira interrogans serovar Pomona str. Kennewicki LC82-25 (taxid:1049924), Leptospira interrogans serovar Pomona str. CSL4002 (taxid:1240691), Leptospira interrogans serovar Pomona str. Pomona (taxid:1001587), Leptospira interrogans serovar Pomona str. Fox 32256 (taxid:1218574), Leptospira interrogans serovar Pomona str. CSL10083 (taxid:1240692), Leptospira kirschneri serovar Pomona (taxid:561005), Leptospira borgpetersenii serovar Pomona (taxid:1192865), Leptospira borgpetersenii serovar Pomona str. 200901868 (taxid:1192866)

RID: DRCNYJJB01R
Job Title:JN627495:Leptospira borgpetersenii serovar...
Program: BLASTN
Query: Leptospira borgpetersenii serovar Sejroe leucine-rich repeat protein (LRR) gene, partial cds ID: JX522460.1(nucleic acid) Length: 612
Database: nt Nucleotide collection (nt)

Sequences producing significant alignments:

Description	Scientific Name	Common Name	Taxid	Max Score	Total Score	Query cover	E Value	Per. Ident	Acc. Len	Accession
Leptospira interrogans strain UI29382 chromosome 1, complete...	Leptospira i...	NA	173	482	482	95%	7e-135	78.19	4311919	CP072853.1
Leptospira interrogans serovar Canicola strain 611 chromosome ...	Leptospira i...	NA	211880	478	478	95%	3e-133	78.02	4255595	CP044513.1
Leptospira interrogans serovar Canicola strain LJ178 chromosom...	Leptospira i...	NA	211880	478	478	95%	3e-133	78.02	4259066	CP044509.1
Leptospira interrogans serovar Canicola strain 114 chromosome ...	Leptospira i...	NA	211880	478	478	95%	3e-133	78.02	4218946	CP022883.1
Leptospira interrogans strain FMAS_PN3 chromosome 1, complete...	Leptospira i...	NA	173	478	478	95%	3e-133	78.02	4263490	CP092663.1
Leptospira interrogans strain FMAS_AP1 chromosome 1, complete...	Leptospira i...	NA	173	478	478	95%	3e-133	78.02	4281936	CP092681.1
Leptospira interrogans strain FMAS_AP7 chromosome 1, complete...	Leptospira i...	NA	173	478	478	95%	3e-133	78.02	4298104	CP092152.1
Leptospira interrogans serovar Bataviae strain D64 chromosome ...	Leptospira i...	NA	312175	478	478	95%	3e-133	78.02	4355742	CP077942.1
Leptospira interrogans serovar Bataviae strain 1548 chromosome...	Leptospira i...	NA	312175	478	478	95%	3e-133	78.02	4354848	CP043880.1

Leptospira interrogans serovar Canicola strain RUFN chromosome I	Leptospira i...	NA	211880	474	474	95%	4e-132	78.02	4208129	CP022885.1
Leptospira interrogans strain FMAS_KW2 chromosome I, complete...	Leptospira i...	NA	173	473	473	95%	4e-132	77.85	4293951	CP039256.1
Leptospira interrogans serovar Manilae strain UP-MMC-NIID HP...	Leptospira i...	NA	214675	473	473	95%	4e-132	77.85	4238922	CP011934.1
Leptospira interrogans serovar Manilae strain UP-MMC-NIID LP...	Leptospira i...	NA	214675	473	473	95%	4e-132	77.85	4238972	CP011931.1
Leptospira interrogans strain FMAS_RT2 chromosome 1, complete...	Leptospira i...	NA	173	473	473	95%	4e-132	77.85	4342805	CP092166.1
Leptospira interrogans strain FMAS_PN2 chromosome 1, complete...	Leptospira i...	NA	173	473	473	95%	4e-132	77.85	4302184	CP092151.1
Leptospira interrogans serovar Canicola strain 782 chromosome ...	Leptospira i...	NA	211880	473	473	95%	4e-132	77.85	4556406	CP043884.1
Leptospira interrogans serovar Copenhageni strain SK1 chromoso...	Leptospira i...	NA	44275	469	469	95%	2e-130	77.68	4279936	CP048830.1
Leptospira interrogans strain R12 chromosome 1	Leptospira i...	NA	173	469	469	95%	2e-130	77.68	4223477	CP047508.1
Leptospira interrogans strain R19 chromosome 1	Leptospira i...	NA	173	469	469	95%	2e-130	77.68	4213431	CP047514.1
Leptospira interrogans strain R7 chromosome 1	Leptospira i...	NA	173	469	469	95%	2e-130	77.68	4212939	CP047512.1
Leptospira interrogans strain R11 chromosome 1	Leptospira i...	NA	173	469	469	95%	2e-130	77.68	4214328	CP047510.1
Leptospira interrogans strain R13-L chromosome 1	Leptospira i...	NA	173	469	469	95%	2e-130	77.68	4233607	CP047518.1
Leptospira interrogans strain R17 chromosome 1	Leptospira i...	NA	173	469	469	95%	2e-130	77.68	4213733	CP047500.1
Leptospira interrogans strain R13 chromosome 1	Leptospira i...	NA	173	469	469	95%	2e-130	77.68	4214175	CP047506.1
Leptospira interrogans strain R22 chromosome 1	Leptospira i...	NA	173	469	469	95%	2e-130	77.68	4206042	CP047496.1
Leptospira interrogans strain R21 chromosome 1	Leptospira i...	NA	173	469	469	95%	2e-130	77.68	4214555	CP047498.1
Leptospira interrogans strain R16 chromosome 1	Leptospira i...	NA	173	469	469	95%	2e-130	77.68	4213648	CP047502.1
Leptospira interrogans serovar Hardjo strain L53 chromosome 1,...	Leptospira i...	NA	176	469	469	95%	2e-130	77.68	4355568	CP043041.1
Leptospira interrogans strain FMAS_KW1 chromosome I, complete...	Leptospira i...	NA	173	469	469	95%	2e-130	77.68	4306144	CP039258.1
Leptospira interrogans strain FMAS_AW1 chromosome I, complete...	Leptospira i...	NA	173	469	469	95%	2e-130	77.68	4497709	CP039283.1
Leptospira interrogans serovar Copenhageni strain FDAARGOS_203...	Leptospira i...	NA	44275	469	469	95%	2e-130	77.68	4280582	CP020414.2
Leptospira interrogans strain RCA chromosome I	Leptospira i...	NA	173	469	469	95%	2e-130	77.68	4241665	CP022538.1

Leptospira interrogans serovar Hardjo-prajitno strain...	Leptospira i...	NA	38347	469	469	95%	2e-130	77.68	4339653	CP013147.1
Leptospira interrogans serovar Hardjo str. Norma chromosome I,...	Leptospira i...	NA	1279460	469	469	95%	2e-130	77.68	4406718	CP012603.1
Leptospira interrogans serovar Bratislava strain PigK151...	Leptospira i...	NA	338215	469	469	95%	2e-130	77.68	4370471	CP011410.1
Leptospira interrogans serovar Linhai str. 56609 chromosome 1,...	Leptospira i...	NA	1395589	469	469	95%	2e-130	77.68	4331770	CP006723.1
Leptospira interrogans strain KR40 chromosome 1, complete...	Leptospira i...	NA	173	469	469	95%	2e-130	77.68	4315670	CP096129.2
Leptospira interrogans strain N 116 chromosome 1, complete...	Leptospira i...	NA	173	469	469	95%	2e-130	77.68	4312952	CP097315.1
Leptospira interrogans strain NEG7 chromosome I	Leptospira i...	NA	173	469	469	95%	2e-130	77.68	4297584	CP093938.1
Leptospira interrogans strain FMAS_KG2 chromosome 1, complete...	Leptospira i...	NA	173	469	469	95%	2e-130	77.68	4721761	CP092985.1
Leptospira interrogans strain FMAS_PD1 chromosome 1, complete...	Leptospira i...	NA	173	469	469	95%	2e-130	77.68	4503505	CP092743.1
Leptospira interrogans strain FMAS_AW2 chromosome 1, complete...	Leptospira i...	NA	173	469	469	95%	2e-130	77.68	4634565	CP092739.1
Leptospira interrogans strain FMAS_AW3 chromosome 1, complete...	Leptospira i...	NA	173	469	469	95%	2e-130	77.68	4526110	CP092676.1
Leptospira interrogans strain FMAS_KG1 chromosome 1, complete...	Leptospira i...	NA	173	469	469	95%	2e-130	77.68	4458643	CP092672.1
Leptospira interrogans strain FMAS_AP6 chromosome 1, complete...	Leptospira i...	NA	173	469	469	95%	2e-130	77.68	4525852	CP092156.1
Leptospira interrogans strain FMAS_AP5 chromosome 1, complete...	Leptospira i...	NA	173	469	469	95%	2e-130	77.68	4478874	CP092161.1
Leptospira interrogans serovar Bataviae strain 1489 chromosome...	Leptospira i...	NA	312175	469	469	95%	2e-130	77.68	4431091	CP043893.1
Leptospira interrogans serovar Icterohaemorrhagiae strain 898...	Leptospira i...	NA	90062	469	469	95%	2e-130	77.68	4280412	CP043891.1
Leptospira interrogans serovar Icterohaemorrhagiae strain...	Leptospira i...	NA	90062	469	469	95%	2e-130	77.68	4367462	CP043876.1
Leptospira interrogans serovar Lai str. IPAV chromosome 1,...	Leptospira i...	NA	573825	469	469	95%	2e-130	77.68	4349158	CP001221.1
Leptospira interrogans serovar Lai str. 56601 chromosome I,...	Leptospira i...	NA	189518	469	469	95%	2e-130	77.68	4338762	AE010300.2
Leptospira interrogans serovar Copenhageni str. Fiocruz L1-130...	Leptospira i...	NA	267671	469	469	95%	2e-130	77.68	4277185	AE016823.1
Leptospira interrogans serovar Copenhageni/Icterohaemorrhagiae...	Leptospira i...	NA	766041	465	465	95%	2e-129	77.68	4221879	CP018146.1
Leptospira interrogans serovar Canicola leucine-rich repeat...	Leptospira i...	NA	211880	325	325	66%	1e-87	77.78	410	JX426070.1
Leptospira interrogans serovar Autumnalis leucine-rich repeat...	Leptospira i...	NA	174157	321	321	66%	5e-86	77.53	411	JX426069.1

Leptospira interrogans serovar Hebdomadis leucine-rich repeat...	Leptospira i...	NA	211881	259	259	47%	1e-67	79.86	303	JX426072.1
Leptospira interrogans serovar Pyrogenes leucine-rich repeat...	Leptospira i...	NA	280500	214	214	49%	5e-54	75.58	312	JX426071.1

```
Alignments:

>Leptospira interrogans strain UI29382 chromosome 1, complete sequence
Sequence ID: CP072853.1 Length: 4311919
Range 1: 1377193 to 1377779

Score:482 bits(534), Expect:7e-135,
Identities:459/587(78%),  Gaps:0/587(0%), Strand: Plus/Plus

Query  26       TCGACTGTAAAAAGAATGCGGAAGAGATTTTGGGAGAGGCAAAGGCAAAACCGGAATTGG  85
                |||| |||||||| || |||| ||||||||| | ||| ||||||  |||| |||||| | 
Sbjct  1377193  TCGATTGTAAAAAAAACGCGGTAGAGATTTTAGAAGAAGCAAAGAGAAAATCGGAATCGA  1377252

Query  86       TTCAAACTTTGGATTTTGGAATGCAGAAGTTGTCCACGGTTCCGGAAGAAGTTTGCGGTT  145
                ||||||  || ||||| |||||||| |||||  || || |||| |||| |||||||  ||
Sbjct  1377253  TTCAAATCTTAGATTTAGGAATGCAAAAGTTAACCTCGATTCCAGAAGGAGTTTGCTCTT  1377312

Query  146      TTCCGAATTTAACCAAGTTGGATCTTCGTTTAAACAGTTTGACTTTTCTTCCGGAATTTA  205
                |||||||||| ||  || ||||| | ||||| |||||||||| || |||||| |  |  |
Sbjct  1377313  TTCCGAATTTGACTCAGCTGGATTTACGTTTGAACAGTTTGAATTCTCTTCCTGGTTGGA  1377372

Query  206      TCGGAGAATGTAAACGCCTTGAACAACTTAATCTTTTCGGGAACGACCTTACAACATTTC  265
                | ||||  ||||||   || |||||| |||| |||||||| || || || |  ||  |||
Sbjct  1377373  TTGGAGCTTGTAAAAATCTGGAACAAATTAACCTTTTCGGAAATGATCTAAATACGGTTC  1377432

Query  266      CGTCTACGTTTTCTAAATTAAAAAATCTGAAAGTGTTACTGGCGGGAAATAACGATTTTA  325
                | ||| | |||||||||||||| ||| ||||||| || ||   ||| |||||||||||||
Sbjct  1377433  CATCTTCTTTTTCTAAATTAAAGAATTTGAAAGTATTGCTCTTGGGTAATAACGATTTTA  1377492

Query  326      CGATTCTACCCTCCGAACTTTTGTTTCTTCCGTTGATCAAAATTCTATACGTAGATCGGA  385
                |  ||||||| || |||||||||||||| || ||||| ||||  || ||| | ||||  |
Sbjct  1377493  CTTTTCTACCTTCGGAACTTTTGTTTCTGCCTTTGATTAAAACCCTGTACTTGGATCAAA  1377552

Query  386      ATAAATTAACTCTAACGGAAACGGATGTGGAAATTCTCGCTTCTCTTTCCTCCTTGGAGG  445
                | ||  | ||  | || ||||||||||| |||||||| ||||||||||||   ||||| |
Sbjct  1377553  ACAAGCTTACGTTGACCGAAACGGATGTTGAAATTCTAGCTTCTCTTTCCAGTTTGGAAG  1377612

Query  446      AATTGGATCTGAGTCTGAATTCGGGGATTAAGGCGCTCCCTTTTAATTACGAAAAACTTG  505
                |||||||||| | | |||| || || ||||| | ||| |||| ||| ||  | ||| |  
Sbjct  1377613  AATTGGATCTTAATTTGAACTCTGGAATTAAAGTGCTTCCTTCTAACTATAATAAATTGA  1377672

Query  506      TAAATCTCATTAATCTAAAAAGATTGAATATTaaaaaaaCTTCATTAAAGGGAGAGGATG  565
                 |||||| | | || ||||||||||||||||||||||||||||||||||||| || ||||
Sbjct  1377673  AAAATCTGACTCATTTAAAAAGATTGAATATTAAAAAAACTTCATTAAAGGGGGAAGATG  1377732

Query  566      CAGATAAATTGCAGGCGATTCTTCCAAATACCAAAATCGACTACTGA  612
                | |||||||| || ||  |||| |||||||| ||||| ||||| |||
Sbjct  1377733  CTGATAAATTACAAGCAGTTCTCCCAAATACAAAAATAGACTATTGA  1377779



>Leptospira interrogans serovar Canicola strain 611 chromosome 1, complete sequence
Sequence ID: CP044513.1 Length: 4255595
Range 1: 1351973 to 1352559

Score:478 bits(529), Expect:3e-133,
Identities:458/587(78%),  Gaps:0/587(0%), Strand: Plus/Plus

Query  26       TCGACTGTAAAAAGAATGCGGAAGAGATTTTGGGAGAGGCAAAGGCAAAACCGGAATTGG  85
                |||| |||||||| || |||| ||||||||| | ||| |||||   |||| |||||| | 
Sbjct  1351973  TCGATTGTAAAAAAAACGCGGTAGAGATTTTAGAAGAAGCAAAAAGAAAATCGGAATCGA  1352032
```

```
Query  86       TTCAAACTTTGGATTTTGGAATGCAGAAGTTGTCCACGGTTCCGGAAGAAGTTTGCGGTT  145
                ||||||  || ||||| |||||||| |||||  || || |||| |||| |||||||  ||
Sbjct  1352033  TTCAAATCTTAGATTTAGGAATGCAAAAGTTAACCTCGATTCCAGAAGGAGTTTGCTCTT  1352092

Query  146      TTCCGAATTTAACCAAGTTGGATCTTCGTTTAAACAGTTTGACTTTTCTTCCGGAATTTA  205
                |||||||||| ||  || ||||| | ||||| |||||||||| || |||||| |  |  |
Sbjct  1352093  TTCCGAATTTGACTCAGCTGGATTTACGTTTGAACAGTTTGAATTCTCTTCCTGGTTGGA  1352152

Query  206      TCGGAGAATGTAAACGCCTTGAACAACTTAATCTTTTCGGGAACGACCTTACAACATTTC  265
                | ||||  ||||||   || |||||| |||| |||||||| || || || |  ||  |||
Sbjct  1352153  TTGGAGCTTGTAAAAATCTGGAACAAATTAACCTTTTCGGAAATGATCTAAATACGGTTC  1352212

Query  266      CGTCTACGTTTTCTAAATTAAAAAATCTGAAAGTGTTACTGGCGGGAAATAACGATTTTA  325
                | ||| | |||||||||||||| ||| ||||||| || ||   ||| |||||||||||||
Sbjct  1352213  CATCTTCTTTTTCTAAATTAAAGAATTTGAAAGTATTGCTCTTGGGTAATAACGATTTTA  1352272

Query  326      CGATTCTACCCTCCGAACTTTTGTTTCTTCCGTTGATCAAAATTCTATACGTAGATCGGA  385
                |  ||||||| || |||||||||||||| || ||||| ||||  || ||| | ||||  |
Sbjct  1352273  CTTTTCTACCTTCGGAACTTTTGTTTCTGCCTTTGATTAAAACCCTGTACTTGGATCAAA  1352332

Query  386      ATAAATTAACTCTAACGGAAACGGATGTGGAAATTCTCGCTTCTCTTTCCTCCTTGGAGG  445
                | ||  | ||  | || ||||||||||| |||||||| ||||||||||||   ||||| |
Sbjct  1352333  ACAAGCTTACGTTGACCGAAACGGATGTTGAAATTCTAGCTTCTCTTTCCAGTTTGGAAG  1352392

Query  446      AATTGGATCTGAGTCTGAATTCGGGGATTAAGGCGCTCCCTTTTAATTACGAAAAACTTG  505
                |||||||||| | | |||| || || ||||| | ||| |||| ||| ||  | ||| |
Sbjct  1352393  AATTGGATCTTAATTTGAACTCTGGAATTAAAGTGCTTCCTTCTAACTATAATAAATTGA  1352452

Query  506      TAAATCTCATTAATCTAAAAAGATTGAATATTaaaaaaaCTTCATTAAAGGGAGAGGATG  565
                 |||||| | | || |||||||||||||||||||||||||||||||||||||||| ||||
Sbjct  1352453  AAAATCTGACTCATTTAAAAAGATTGAATATTAAAAAAACTTCATTAAAGGGAGAAGATG  1352512

Query  566      CAGATAAATTGCAGGCGATTCTTCCAAATACCAAAATCGACTACTGA  612
                | |||||||| || ||  |||| |||||||| | ||| ||||| |||
Sbjct  1352513  CTGATAAATTACAAGCAGTTCTCCCAAATACAAGAATAGACTATTGA  1352559



>Leptospira interrogans serovar Canicola strain LJ178 chromosome 1, complete sequence
Sequence ID: CP044509.1 Length: 4259066
Range 1: 1357390 to 1357976

Score:478 bits(529), Expect:3e-133,
Identities:458/587(78%),  Gaps:0/587(0%), Strand: Plus/Plus

Query  26       TCGACTGTAAAAAGAATGCGGAAGAGATTTTGGGAGAGGCAAAGGCAAAACCGGAATTGG  85
                |||| |||||||| || |||| ||||||||| | ||| |||||   |||| |||||| |
Sbjct  1357390  TCGATTGTAAAAAAAACGCGGTAGAGATTTTAGAAGAAGCAAAAAGAAAATCGGAATCGA  1357449

Query  86       TTCAAACTTTGGATTTTGGAATGCAGAAGTTGTCCACGGTTCCGGAAGAAGTTTGCGGTT  145
                ||||||  || ||||| |||||||| |||||  || || |||| |||| |||||||  ||
Sbjct  1357450  TTCAAATCTTAGATTTAGGAATGCAAAAGTTAACCTCGATTCCAGAAGGAGTTTGCTCTT  1357509

Query  146      TTCCGAATTTAACCAAGTTGGATCTTCGTTTAAACAGTTTGACTTTTCTTCCGGAATTTA  205
                |||||||||| ||  || ||||| | ||||| |||||||||| || |||||| |  |  |
Sbjct  1357510  TTCCGAATTTGACTCAGCTGGATTTACGTTTGAACAGTTTGAATTCTCTTCCTGGTTGGA  1357569

Query  206      TCGGAGAATGTAAACGCCTTGAACAACTTAATCTTTTCGGGAACGACCTTACAACATTTC  265
                | ||||  ||||||   || |||||| |||| |||||||| || || || |  ||  |||
Sbjct  1357570  TTGGAGCTTGTAAAAATCTGGAACAAATTAACCTTTTCGGAAATGATCTAAATACGGTTC  1357629

Query  266      CGTCTACGTTTTCTAAATTAAAAAATCTGAAAGTGTTACTGGCGGGAAATAACGATTTTA  325
```

```
                | ||| | |||||||||||||| ||| ||||||| || ||   ||| |||||||||||||
Sbjct  1357630  CATCTTCTTTTTCTAAATTAAAGAATTTGAAAGTATTGCTCTTGGGTAATAACGATTTTA  1357689

Query  326      CGATTCTACCCTCCGAACTTTTGTTTCTTCCGTTGATCAAAATTCTATACGTAGATCGGA  385
                |  ||||||| || |||||||||||||| || ||||| ||||  || ||| | ||||  |
Sbjct  1357690  CTTTTCTACCTTCGGAACTTTTGTTTCTGCCTTTGATTAAAACCCTGTACTTGGATCAAA  1357749

Query  386      ATAAATTAACTCTAACGGAAACGGATGTGGAAATTCTCGCTTCTCTTTCCTCCTTGGAGG  445
                | ||  | ||  | || ||||||||||| |||||||| ||||||||||||   ||||| |
Sbjct  1357750  ACAAGCTTACGTTGACCGAAACGGATGTTGAAATTCTAGCTTCTCTTTCCAGTTTGGAAG  1357809

Query  446      AATTGGATCTGAGTCTGAATTCGGGGATTAAGGCGCTCCCTTTTAATTACGAAAAACTTG  505
                |||||||||| | | |||| || || ||||| | ||| |||| ||| ||  | ||| |
Sbjct  1357810  AATTGGATCTTAATTTGAACTCTGGAATTAAAGTGCTTCCTTCTAACTATAATAAATTGA  1357869

Query  506      TAAATCTCATTAATCTAAAAAGATTGAATATTaaaaaaaCTTCATTAAAGGGAGAGGATG  565
                 |||||| | | || |||||||||||||||||||||||||||||||||||||||| ||||
Sbjct  1357870  AAAATCTGACTCATTTAAAAAGATTGAATATTAAAAAAACTTCATTAAAGGGAGAAGATG  1357929

Query  566      CAGATAAATTGCAGGCGATTCTTCCAAATACCAAAATCGACTACTGA  612
                | |||||||| || ||  |||| |||||||| | ||| ||||| |||
Sbjct  1357930  CTGATAAATTACAAGCAGTTCTCCCAAATACAAGAATAGACTATTGA  1357976
```

>Leptospira interrogans serovar Canicola strain 114 chromosome I sequence
Sequence ID: CP022883.1 Length: 4218946
Range 1: 3467024 to 3467610

Score:478 bits(529), Expect:3e-133,
Identities:458/587(78%), Gaps:0/587(0%), Strand: Plus/Plus

```
Query  26       TCGACTGTAAAAAGAATGCGGAAGAGATTTTGGGAGAGGCAAAGGCAAAACCGGAATTGG  85
                |||| |||||||| || |||| ||||||||| | ||| |||||   |||| |||||| |
Sbjct  3467024  TCGATTGTAAAAAAAACGCGGTAGAGATTTTAGAAGAAGCAAAAAGAAAATCGGAATCGA  3467083

Query  86       TTCAAACTTTGGATTTTGGAATGCAGAAGTTGTCCACGGTTCCGGAAGAAGTTTGCGGTT  145
                ||||||  || ||||| |||||||| |||||  || || |||| |||| |||||||  ||
Sbjct  3467084  TTCAAATCTTAGATTTAGGAATGCAAAAGTTAACCTCGATTCCAGAAGGAGTTTGCTCTT  3467143

Query  146      TTCCGAATTTAACCAAGTTGGATCTTCGTTTAAACAGTTTGACTTTTCTTCCGGAATTTA  205
                |||||||||| ||  || ||||| | ||||| |||||||||| || |||||| |  |  |
Sbjct  3467144  TTCCGAATTTGACTCAGCTGGATTTACGTTTGAACAGTTTGAATTCTCTTCCTGGTTGGA  3467203

Query  206      TCGGAGAATGTAAACGCCTTGAACAACTTAATCTTTTCGGGAACGACCTTACAACATTTC  265
                | ||||  ||||||   || |||||| |||| |||||||| || || || |  ||  |||
Sbjct  3467204  TTGGAGCTTGTAAAAATCTGGAACAAATTAACCTTTTCGGAAATGATCTAAATACGGTTC  3467263

Query  266      CGTCTACGTTTTCTAAATTAAAAAATCTGAAAGTGTTACTGGCGGGAAATAACGATTTTA  325
                | ||| | |||||||||||||| ||| ||||||| || ||   ||| |||||||||||||
Sbjct  3467264  CATCTTCTTTTTCTAAATTAAAGAATTTGAAAGTATTGCTCTTGGGTAATAACGATTTTA  3467323

Query  326      CGATTCTACCCTCCGAACTTTTGTTTCTTCCGTTGATCAAAATTCTATACGTAGATCGGA  385
                |  ||||||| || |||||||||||||| || ||||| ||||  || ||| | ||||  |
Sbjct  3467324  CTTTTCTACCTTCGGAACTTTTGTTTCTGCCTTTGATTAAAACCCTGTACTTGGATCAAA  3467383

Query  386      ATAAATTAACTCTAACGGAAACGGATGTGGAAATTCTCGCTTCTCTTTCCTCCTTGGAGG  445
                | ||  | ||  | || ||||||||||| |||||||| ||||||||||||   ||||| |
Sbjct  3467384  ACAAGCTTACGTTGACCGAAACGGATGTTGAAATTCTAGCTTCTCTTTCCAGTTTGGAAG  3467443

Query  446      AATTGGATCTGAGTCTGAATTCGGGGATTAAGGCGCTCCCTTTTAATTACGAAAAACTTG  505
                |||||||||| | | |||| || || ||||| | ||| |||| ||| ||  | ||| |
```

```
Sbjct  3467444  AATTGGATCTTAATTTGAACTCTGGAATTAAAGTGCTTCCTTCTAACTATAATAAATTGA  3467503

Query  506      TAAATCTCATTAATCTAAAAAGATTGAATATTaaaaaaaCTTCATTAAAGGGAGAGGATG  565
                 |||||| | | || ||||||||||||||||||||||||||||||||||||||| ||||
Sbjct  3467504  AAAATCTGACTCATTTAAAAAGATTGAATATTAAAAAAACTTCATTAAAGGGAGAAGATG  3467563

Query  566      CAGATAAATTGCAGGCGATTCTTCCAAATACCAAAATCGACTACTGA  612
                | |||||||| || ||  |||| |||||||| | ||| ||||| |||
Sbjct  3467564  CTGATAAATTACAAGCAGTTCTCCCAAATACAAGAATAGACTATTGA  3467610
```

>Leptospira interrogans strain FMAS_PN3 chromosome 1, complete sequence
Sequence ID: CP092663.1 Length: 4263490
Range 1: 2862050 to 2862636

Score:478 bits(529), Expect:3e-133,
Identities:458/587(78%), Gaps:0/587(0%), Strand: Plus/Minus

```
Query  26       TCGACTGTAAAAAGAATGCGGAAGAGATTTTGGGAGAGGCAAAGGCAAAACCGGAATTGG  85
                |||| |||||||| || |||| ||||||||| | ||| |||||   |||| |||||| |
Sbjct  2862636  TCGATTGTAAAAAAAACGCGGTAGAGATTTTAGAAGAAGCAAAAAGAAAATCGGAATCGA  2862577

Query  86       TTCAAACTTTGGATTTTGGAATGCAGAAGTTGTCCACGGTTCCGGAAGAAGTTTGCGGTT  145
                ||||||  || ||||| |||||||| |||||  || || |||| |||| |||||||  ||
Sbjct  2862576  TTCAAATCTTAGATTTAGGAATGCAAAAGTTAACCTCGATTCCAGAAGGAGTTTGCTCTT  2862517

Query  146      TTCCGAATTTAACCAAGTTGGATCTTCGTTTAAACAGTTTGACTTTTCTTCCGGAATTTA  205
                |||||||||| ||  || ||||| | ||||| |||||||||| || |||||| |  |  |
Sbjct  2862516  TTCCGAATTTGACTCAGCTGGATTTACGTTTGAACAGTTTGAATTCTCTTCCTGGTTGGA  2862457

Query  206      TCGGAGAATGTAAACGCCTTGAACAACTTAATCTTTTCGGGAACGACCTTACAACATTTC  265
                | ||||  ||||||   || |||||| |||| |||||||| || || || |  ||  |||
Sbjct  2862456  TTGGAGCTTGTAAAAATCTGGAACAAATTAACCTTTTCGGAAATGATCTAAATACGGTTC  2862397

Query  266      CGTCTACGTTTTCTAAATTAAAAAATCTGAAAGTGTTACTGGCGGGAAATAACGATTTTA  325
                | ||| | |||||||||||||| ||| ||||||| || ||   ||| |||||||||||||
Sbjct  2862396  CATCTTCTTTTTCTAAATTAAAGAATTTGAAAGTATTGCTCTTGGGTAATAACGATTTTA  2862337

Query  326      CGATTCTACCCTCCGAACTTTTGTTTCTTCCGTTGATCAAAATTCTATACGTAGATCGGA  385
                |  ||||||| || |||||||||||||| || ||||| ||||  || ||| | ||||  |
Sbjct  2862336  CTTTTCTACCTTCGGAACTTTTGTTTCTGCCTTTGATTAAAACCCTGTACTTGGATCAAA  2862277

Query  386      ATAAATTAACTCTAACGGAAACGGATGTGGAAATTCTCGCTTCTCTTTCCTCCTTGGAGG  445
                | ||  | ||  | || ||||||||||| |||||||| ||||||||||||   ||||| |
Sbjct  2862276  ACAAGCTTACGTTGACCGAAACGGATGTTGAAATTCTAGCTTCTCTTTCCAGTTTGGAAG  2862217

Query  446      AATTGGATCTGAGTCTGAATTCGGGGATTAAGGCGCTCCCTTTTAATTACGAAAAACTTG  505
                |||||||||| | | |||| || || || || | ||| |||| ||||||  | ||| |
Sbjct  2862216  AATTGGATCTTAATTTGAACTCTGGAATCAAAGTGCTTCCTTCTAATTATAATAAATTGA  2862157

Query  506      TAAATCTCATTAATCTAAAAAGATTGAATATTaaaaaaaCTTCATTAAAGGGAGAGGATG  565
                 |||||| | | || ||||||||||||||||||||||||||||||||||||| |||||||
Sbjct  2862156  AAAATCTGACTCATTTAAAAAGATTGAATATTAAAAAAACTTCATTAAAGGGGGAGGATG  2862097

Query  566      CAGATAAATTGCAGGCGATTCTTCCAAATACCAAAATCGACTACTGA  612
                | |||||||| || ||  |||| |||||||| | ||| ||||| |||
Sbjct  2862096  CTGATAAATTACAAGCAGTTCTCCCAAATACAAGAATAGACTATTGA  2862050
```

```
>Leptospira interrogans strain FMAS_AP1 chromosome 1, complete sequence
Sequence ID: CP092681.1 Length: 4281936
Range 1: 2877199 to 2877785

Score:478 bits(529), Expect:3e-133,
Identities:458/587(78%),  Gaps:0/587(0%), Strand: Plus/Minus

Query  26       TCGACTGTAAAAAGAATGCGGAAGAGATTTTGGGAGAGGCAAAGGCAAAACCGGAATTGG  85
                |||| |||||||| || |||| ||||||||| | ||| |||||   |||| |||||| |
Sbjct  2877785  TCGATTGTAAAAAAAACGCGGTAGAGATTTTAGAAGAAGCAAAAAGAAAATCGGAATCGA  2877726

Query  86       TTCAAACTTTGGATTTTGGAATGCAGAAGTTGTCCACGGTTCCGGAAGAAGTTTGCGGTT  145
                ||||||  || ||||| |||||||| |||||  || || |||| |||| |||||||  ||
Sbjct  2877725  TTCAAATCTTAGATTTAGGAATGCAAAAGTTAACCTCGATTCCAGAAGGAGTTTGCTCTT  2877666

Query  146      TTCCGAATTTAACCAAGTTGGATCTTCGTTTAAACAGTTTGACTTTTCTTCCGGAATTTA  205
                |||||||||| ||  || ||||| | ||||| |||||||||| || |||||| |  |  |
Sbjct  2877665  TTCCGAATTTGACTCAGCTGGATTTACGTTTGAACAGTTTGAATTCTCTTCCTGGTTGGA  2877606

Query  206      TCGGAGAATGTAAACGCCTTGAACAACTTAATCTTTTCGGGAACGACCTTACAACATTTC  265
                | ||||  ||||||   || |||||| |||| |||||||| || || || |  ||  |||
Sbjct  2877605  TTGGAGCTTGTAAAAATCTGGAACAAATTAACCTTTTCGGAAATGATCTAAATACGGTTC  2877546

Query  266      CGTCTACGTTTTCTAAATTAAAAAATCTGAAAGTGTTACTGGCGGGAAATAACGATTTTA  325
                | ||| | |||||||||||||| ||| ||||||| || ||   ||| |||||||||||||
Sbjct  2877545  CATCTTCTTTTTCTAAATTAAAGAATTTGAAAGTATTGCTCTTGGGTAATAACGATTTTA  2877486

Query  326      CGATTCTACCCTCCGAACTTTTGTTTCTTCCGTTGATCAAAATTCTATACGTAGATCGGA  385
                |  ||||||| || |||||||||||||| || ||||| ||||  || ||| | ||||  |
Sbjct  2877485  CTTTTCTACCTTCGGAACTTTTGTTTCTGCCTTTGATTAAAACCCTGTACTTGGATCAAA  2877426

Query  386      ATAAATTAACTCTAACGGAAACGGATGTGGAAATTCTCGCTTCTCTTTCCTCCTTGGAGG  445
                | ||  | ||  | || ||||||||||| |||||||| ||||||||||||   ||||| |
Sbjct  2877425  ACAAGCTTACGTTGACCGAAACGGATGTTGAAATTCTAGCTTCTCTTTCCAGTTTGGAAG  2877366

Query  446      AATTGGATCTGAGTCTGAATTCGGGGATTAAGGCGCTCCCTTTTAATTACGAAAAACTTG  505
                |||||||||| | | |||| || || || || | ||| |||| ||||||  | ||| |
Sbjct  2877365  AATTGGATCTTAATTTGAACTCTGGAATCAAAGTGCTTCCTTCTAATTATAATAAATTGA  2877306

Query  506      TAAATCTCATTAATCTAAAAAGATTGAATATTaaaaaaaCTTCATTAAAGGGAGAGGATG  565
                 |||||| | | || ||||||||||||||||||||||||||||||||||||| |||||||
Sbjct  2877305  AAAATCTGACTCATTTAAAAAGATTGAATATTAAAAAAACTTCATTAAAGGGGGAGGATG  2877246

Query  566      CAGATAAATTGCAGGCGATTCTTCCAAATACCAAAATCGACTACTGA  612
                | |||||||| || ||  |||| |||||||| | ||| ||||| |||
Sbjct  2877245  CTGATAAATTACAAGCAGTTCTCCCAAATACAAGAATAGACTATTGA  2877199
```

```
>Leptospira interrogans strain FMAS_AP7 chromosome 1, complete sequence
Sequence ID: CP092152.1 Length: 4298104
Range 1: 4193201 to 4193787

Score:478 bits(529), Expect:3e-133,
Identities:458/587(78%),  Gaps:0/587(0%), Strand: Plus/Minus

Query  26       TCGACTGTAAAAAGAATGCGGAAGAGATTTTGGGAGAGGCAAAGGCAAAACCGGAATTGG  85
                |||| |||||||| || |||| ||||||||| | ||| |||||   |||| |||||| |
Sbjct  4193787  TCGATTGTAAAAAAAACGCGGTAGAGATTTTAGAAGAAGCAAAAAGAAAATCGGAATCGA  4193728

Query  86       TTCAAACTTTGGATTTTGGAATGCAGAAGTTGTCCACGGTTCCGGAAGAAGTTTGCGGTT  145
                ||||||  || ||||| |||||||| |||||  || || |||| |||| |||||||  ||
```

```
Sbjct  4193727  TTCAAATCTTAGATTTAGGAATGCAAAAGTTAACCTCGATTCCAGAAGGAGTTTGCTCTT  4193668

Query  146      TTCCGAATTTAACCAAGTTGGATCTTCGTTTAAACAGTTTGACTTTTCTTCCGGAATTTA  205
                |||||||||| ||  || ||||| | ||||| |||||||||| || |||||| |  |  |
Sbjct  4193667  TTCCGAATTTGACTCAGCTGGATTTACGTTTGAACAGTTTGAATTCTCTTCCTGGTTGGA  4193608

Query  206      TCGGAGAATGTAAACGCCTTGAACAACTTAATCTTTTCGGGAACGACCTTACAACATTTC  265
                | ||||  ||||||   || |||||| |||| |||||||| || || || |  ||  |||
Sbjct  4193607  TTGGAGCTTGTAAAAATCTGGAACAAATTAACCTTTTCGGAAATGATCTAAATACGGTTC  4193548

Query  266      CGTCTACGTTTTCTAAATTAAAAAATCTGAAAGTGTTACTGGCGGGAAATAACGATTTTA  325
                | ||| | |||||||||||||| ||| ||||||| || ||   ||| |||||||||||||
Sbjct  4193547  CATCTTCTTTTTCTAAATTAAAGAATTTGAAAGTATTGCTCTTGGGTAATAACGATTTTA  4193488

Query  326      CGATTCTACCCTCCGAACTTTTGTTTCTTCCGTTGATCAAAATTCTATACGTAGATCGGA  385
                |  ||||||| || |||||||||||||| || ||||| ||||  || ||| | ||||  |
Sbjct  4193487  CTTTTCTACCTTCGGAACTTTTGTTTCTGCCTTTGATTAAAACCCTGTACTTGGATCAAA  4193428

Query  386      ATAAATTAACTCTAACGGAAACGGATGTGGAAATTCTCGCTTCTCTTTCCTCCTTGGAGG  445
                | ||  | ||  | || ||||||||||| |||||||| ||||||||||||   ||||| |
Sbjct  4193427  ACAAGCTTACGTTGACCGAAACGGATGTTGAAATTCTAGCTTCTCTTTCCAGTTTGGAAG  4193368

Query  446      AATTGGATCTGAGTCTGAATTCGGGGATTAAGGCGCTCCCTTTTAATTACGAAAAACTTG  505
                |||||||||| | | |||| || || || || | ||| |||| ||||||  | ||| |
Sbjct  4193367  AATTGGATCTTAATTTGAACTCTGGAATCAAAGTGCTTCCTTCTAATTATAATAAATTGA  4193308

Query  506      TAAATCTCATTAATCTAAAAAGATTGAATATTaaaaaaaCTTCATTAAAGGGAGAGGATG  565
                 |||||| | | || ||||||||||||||||||||||||||||||||||||| |||||||
Sbjct  4193307  AAAATCTGACTCATTTAAAAAGATTGAATATTAAAAAAACTTCATTAAAGGGGGAGGATG  4193248

Query  566      CAGATAAATTGCAGGCGATTCTTCCAAATACCAAAATCGACTACTGA  612
                | |||||||| || ||  |||| |||||||| | ||| ||||| |||
Sbjct  4193247  CTGATAAATTACAAGCAGTTCTCCCAAATACAAGAATAGACTATTGA  4193201



>Leptospira interrogans serovar Bataviae strain D64 chromosome I, complete sequence
Sequence ID: CP077942.1 Length: 4355742
Range 1: 1430617 to 1431203

Score:478 bits(529), Expect:3e-133,
Identities:458/587(78%),  Gaps:0/587(0%), Strand: Plus/Plus

Query  26       TCGACTGTAAAAAGAATGCGGAAGAGATTTTGGGAGAGGCAAAGGCAAAACCGGAATTGG  85
                |||| |||||||| || |||| ||||||||| | ||| |||||   |||| |||||| |
Sbjct  1430617  TCGATTGTAAAAAAAACGCGGTAGAGATTTTAGAAGAAGCAAAAAGAAAATCGGAATCGA  1430676

Query  86       TTCAAACTTTGGATTTTGGAATGCAGAAGTTGTCCACGGTTCCGGAAGAAGTTTGCGGTT  145
                ||||||  || ||||| |||||||| |||||  || || |||| |||| |||||||  ||
Sbjct  1430677  TTCAAATCTTAGATTTAGGAATGCAAAAGTTAACCTCGATTCCAGAAGGAGTTTGCTCTT  1430736

Query  146      TTCCGAATTTAACCAAGTTGGATCTTCGTTTAAACAGTTTGACTTTTCTTCCGGAATTTA  205
                |||||||||| ||  || ||||| | ||||| |||||||||| || |||||| |  |  |
Sbjct  1430737  TTCCGAATTTGACTCAGCTGGATTTACGTTTGAACAGTTTGAATTCTCTTCCTGGTTGGA  1430796

Query  206      TCGGAGAATGTAAACGCCTTGAACAACTTAATCTTTTCGGGAACGACCTTACAACATTTC  265
                | ||||  ||||||   || |||||| |||| |||||||| || || || |  ||  |||
Sbjct  1430797  TTGGAGCTTGTAAAAATCTGGAACAAATTAACCTTTTCGGAAATGATCTAAATACGGTTC  1430856

Query  266      CGTCTACGTTTTCTAAATTAAAAAATCTGAAAGTGTTACTGGCGGGAAATAACGATTTTA  325
                | ||| | |||||||||||||| ||| ||||||| || ||   ||| |||||||||||||
Sbjct  1430857  CATCTTCTTTTTCTAAATTAAAGAATTTGAAAGTATTGCTCTTGGGTAATAACGATTTTA  1430916
```

```
Query  326      CGATTCTACCCTCCGAACTTTTGTTTCTTCCGTTGATCAAAATTCTATACGTAGATCGGA  385
                |  |||||||||| |||||||||||||| || ||||| ||||  || ||| | ||||  |
Sbjct  1430917  CTTTTCTACCCTCGGAACTTTTGTTTCTGCCTTTGATTAAAACCCTGTACTTGGATCAAA  1430976

Query  386      ATAAATTAACTCTAACGGAAACGGATGTGGAAATTCTCGCTTCTCTTTCCTCCTTGGAGG  445
                | ||  | ||  | || ||||||||||| |||||||| ||||||||||||   ||||| |
Sbjct  1430977  ACAAGCTTACGTTGACCGAAACGGATGTTGAAATTCTAGCTTCTCTTTCCAGTTTGGAAG  1431036

Query  446      AATTGGATCTGAGTCTGAATTCGGGGATTAAGGCGCTCCCTTTTAATTACGAAAAACTTG  505
                |||||||||| | | |||| || || || || | ||| |||| ||| ||  | ||| |
Sbjct  1431037  AATTGGATCTTAATTTGAACTCTGGAATCAAAGTGCTTCCTTCTAACTATAATAAATTGA  1431096

Query  506      TAAATCTCATTAATCTAAAAAGATTGAATATTaaaaaaaCTTCATTAAAGGGAGAGGATG  565
                 |||||| | | || ||||||||||||||||||||||||||||||||||||| |||||||
Sbjct  1431097  AAAATCTGACTCATTTAAAAAGATTGAATATTAAAAAAACTTCATTAAAGGGGGAGGATG  1431156

Query  566      CAGATAAATTGCAGGCGATTCTTCCAAATACCAAAATCGACTACTGA  612
                | |||||||| || ||  |||| ||||||||| | ||| ||||| |||
Sbjct  1431157  CTGATAAATTACAAGCAGTTCTCCCAAATACAAGAATAGACTATTGA  1431203
```

>Leptospira interrogans serovar Bataviae strain 1548 chromosome, complete genome
Sequence ID: CP043880.1 Length: 4354848
Range 1: 1430028 to 1430614

Score:478 bits(529), Expect:3e-133,
Identities:458/587(78%), Gaps:0/587(0%), Strand: Plus/Plus

```
Query  26       TCGACTGTAAAAAGAATGCGGAAGAGATTTTGGGAGAGGCAAAGGCAAAACCGGAATTGG  85
                |||| |||||||| || |||| ||||||||| | ||| |||||   |||| |||||| |
Sbjct  1430028  TCGATTGTAAAAAAAAACGCGGTAGAGATTTTAGAAGAAGCAAAAAGAAAATCGGAATCGA  1430087

Query  86       TTCAAACTTTGGATTTTGGAATGCAGAAGTTGTCCACGGTTCCGGAAGAAGTTTGCGGTT  145
                ||||||  || ||||| |||||||| |||||  || || |||| |||| |||||||  ||
Sbjct  1430088  TTCAAATCTTAGATTTAGGAATGCAAAAGTTAACCTCGATTCCAGAAGGAGTTTGCTCTT  1430147

Query  146      TTCCGAATTTAACCAAGTTGGATCTTCGTTTAAACAGTTTGACTTTTCTTCCGGAATTTA  205
                |||||||||| ||  || ||||| | ||||| |||||||||| || |||||| |  |  |
Sbjct  1430148  TTCCGAATTTGACTCAGCTGGATTTACGTTTGAACAGTTTGAATTCTCTTCCTGGTTGGA  1430207

Query  206      TCGGAGAATGTAAACGCCTTGAACAACTTAATCTTTTCGGGAACGACCTTACAACATTTC  265
                | ||||  ||||||   || |||||| |||| |||||||| || || || |  ||  |||
Sbjct  1430208  TTGGAGCTTGTAAAAATCTGGAACAAATTAACCTTTTCGGAAATGATCTAAATACGGTTC  1430267

Query  266      CGTCTACGTTTTCTAAATTAAAAAATCTGAAAGTGTTACTGGCGGGAAATAACGATTTTA  325
                | ||| | |||||||||||||| ||| ||||||| || ||   ||| |||||||||||||
Sbjct  1430268  CATCTTCTTTTTCTAAATTAAAGAATTTGAAAGTATTGCTCTTGGGTAATAACGATTTTA  1430327

Query  326      CGATTCTACCCTCCGAACTTTTGTTTCTTCCGTTGATCAAAATTCTATACGTAGATCGGA  385
                |  |||||||||| |||||||||||||| || ||||| ||||  || ||| | ||||  |
Sbjct  1430328  CTTTTCTACCCTCGGAACTTTTGTTTCTGCCTTTGATTAAAACCCTGTACTTGGATCAAA  1430387

Query  386      ATAAATTAACTCTAACGGAAACGGATGTGGAAATTCTCGCTTCTCTTTCCTCCTTGGAGG  445
                | ||  | ||  | || ||||||||||| |||||||| ||||||||||||   ||||| |
Sbjct  1430388  ACAAGCTTACGTTGACCGAAACGGATGTTGAAATTCTAGCTTCTCTTTCCAGTTTGGAAG  1430447

Query  446      AATTGGATCTGAGTCTGAATTCGGGGATTAAGGCGCTCCCTTTTAATTACGAAAAACTTG  505
                |||||||||| | | |||| || || || || | ||| |||| ||| ||  | ||| |
Sbjct  1430448  AATTGGATCTTAATTTGAACTCTGGAATCAAAGTGCTTCCTTCTAACTATAATAAATTGA  1430507
```

```
Query  506      TAAATCTCATTAATCTAAAAAGATTGAATATTaaaaaaaCTTCATTAAAGGGAGAGGATG  565
                 |||||| | | || |||||||||||||||||||||||||||||||||||||| |||||||
Sbjct  1430508  AAAATCTGACTCATTTAAAAAGATTGAATATTAAAAAAACTTCATTAAAGGGGGAGGATG  1430567

Query  566      CAGATAAATTGCAGGCGATTCTTCCAAATACCAAAATCGACTACTGA  612
                | |||||||| || ||  |||| |||||||| | ||| ||||| |||
Sbjct  1430568  CTGATAAATTACAAGCAGTTCTCCCAAATACAAGAATAGACTATTGA  1430614
```

>Leptospira interrogans serovar Canicola strain RUFN chromosome I
Sequence ID: CP022885.1 Length: 4208129
Range 1: 600677 to 601262

Score:474 bits(525), Expect:4e-132,
Identities:458/587(78%), Gaps:1/587(0%), Strand: Plus/Plus

```
Query  26      TCGACTGTAAAAAGAATGCGGAAGAGATTTTGGGAGAGGCAAAGGCAAAACCGGAATTGG  85
               |||| |||||||| || |||| ||||||||| | ||| |||||   |||| |||||| |
Sbjct  600677  TCGATTGTAAAAA-AACGCGGTAGAGATTTTAGAAGAAGCAAAAAGAAAATCGGAATCGA  600735

Query  86      TTCAAACTTTGGATTTTGGAATGCAGAAGTTGTCCACGGTTCCGGAAGAAGTTTGCGGTT  145
               ||||||  || ||||| |||||||| |||||  || || |||| |||| |||||||  ||
Sbjct  600736  TTCAAATCTTAGATTTAGGAATGCAAAAGTTAACCTCGATTCCAGAAGGAGTTTGCTCTT  600795

Query  146     TTCCGAATTTAACCAAGTTGGATCTTCGTTTAAACAGTTTGACTTTTCTTCCGGAATTTA  205
               |||||||||| ||  || ||||| | ||||| |||||||||| || |||||| |  |  |
Sbjct  600796  TTCCGAATTTGACTCAGCTGGATTTACGTTTGAACAGTTTGAATTCTCTTCCTGGTTGGA  600855

Query  206     TCGGAGAATGTAAACGCCTTGAACAACTTAATCTTTTCGGGAACGACCTTACAACATTTC  265
               | ||||  ||||||   || |||||| |||| |||||||| || || || |  ||  |||
Sbjct  600856  TTGGAGCTTGTAAAAATCTGGAACAAATTAACCTTTTCGGAAATGATCTAAATACGGTTC  600915

Query  266     CGTCTACGTTTTCTAAATTAAAAAATCTGAAAGTGTTACTGGCGGGAAATAACGATTTTA  325
               | ||| | |||||||||||||| ||| ||||||| || ||   ||| |||||||||||||
Sbjct  600916  CATCTTCTTTTTCTAAATTAAAGAATTTGAAAGTATTGCTCTTGGGTAATAACGATTTTA  600975

Query  326     CGATTCTACCCTCCGAACTTTTGTTTCTTCCGTTGATCAAAATTCTATACGTAGATCGGA  385
               |  ||||||| || ||||||||||||| || ||||| ||||  || ||| | ||||  |
Sbjct  600976  CTTTTCTACCTTCGGAACTTTTGTTTCTGCCTTTGATTAAAACCCTGTACTTGGATCAAA  601035

Query  386     ATAAATTAACTCTAACGGAAACGGATGTGGAAATTCTCGCTTCTCTTTCCTCCTTGGAGG  445
               | ||  | ||  | || ||||||||||| |||||||| ||||||||||||   ||||| |
Sbjct  601036  ACAAGCTTACGTTGACCGAAACGGATGTTGAAATTCTAGCTTCTCTTTCCAGTTTGGAAG  601095

Query  446     AATTGGATCTGAGTCTGAATTCGGGGATTAAGGCGCTCCCTTTTAATTACGAAAAACTTG  505
               |||||||||| | | |||| || || ||||| | ||| |||| ||| ||  | ||| |
Sbjct  601096  AATTGGATCTTAATTTGAACTCTGGAATTAAAGTGCTTCCTTCTAACTATAATAAATTGA  601155

Query  506     TAAATCTCATTAATCTAAAAAGATTGAATATTaaaaaaaCTTCATTAAAGGGAGAGGATG  565
                |||||| | | || ||||||||||||||||||||||||||||||||||||||||| ||||
Sbjct  601156  AAAATCTGACTCATTTAAAAAGATTGAATATTAAAAAAACTTCATTAAAGGGAGAAGATG  601215

Query  566     CAGATAAATTGCAGGCGATTCTTCCAAATACCAAAATCGACTACTGA  612
               | |||||||| || ||  |||| |||||||| | ||| ||||| |||
Sbjct  601216  CTGATAAATTACAAGCAGTTCTCCCAAATACAAGAATAGACTATTGA  601262
```

>Leptospira interrogans strain FMAS_KW2 chromosome I, complete sequence
Sequence ID: CP039256.1 Length: 4293951

```
Range 1: 2874786 to 2875372

Score:473 bits(524), Expect:4e-132,
Identities:457/587(78%),  Gaps:0/587(0%), Strand: Plus/Minus

Query  26       TCGACTGTAAAAAGAATGCGGAAGAGATTTTGGGAGAGGCAAAGGCAAAACCGGAATTGG  85
                |||| |||||||| || |||| ||||||||| | ||| |||||   |||| |||||| |
Sbjct  2875372  TCGATTGTAAAAAAAAACGCGGTAGAGATTTTAGAAGAAGCAAAAAGAAAATCGGAATCGA  2875313

Query  86       TTCAAACTTTGGATTTTGGAATGCAGAAGTTGTCCACGGTTCCGGAAGAAGTTTGCGGTT  145
                ||||||  || ||||| |||||||| |||||  || || |||| |||| |||||||  ||
Sbjct  2875312  TTCAAATCTTAGATTTAGGAATGCAAAAGTTAACCTCGATTCCAGAAGGAGTTTGCTCTT  2875253

Query  146      TTCCGAATTTAACCAAGTTGGATCTTCGTTTAAACAGTTTGACTTTTCTTCCGGAATTTA  205
                |||||||||| ||  || ||||| | ||||| |||||||||| || |||||| |  |  |
Sbjct  2875252  TTCCGAATTTGACTCAGCTGGATTTACGTTTGAACAGTTTGAATTCTCTTCCTGGTTGGA  2875193

Query  206      TCGGAGAATGTAAACGCCTTGAACAACTTAATCTTTTCGGGAACGACCTTACAACATTTC  265
                | ||||  ||||||   || |||||| |||| |||||||| || || || |  ||  |||
Sbjct  2875192  TTGGAGCTTGTAAAAATCTGGAACAAATTAACCTTTTCGGAAATGATCTAAATACGGTTC  2875133

Query  266      CGTCTACGTTTTCTAAATTAAAAAATCTGAAAGTGTTACTGGCGGGAAATAACGATTTTA  325
                | ||| | |||||||||||||| ||| ||||||| || ||   ||| |||||||||||||
Sbjct  2875132  CATCTTCTTTTTCTAAATTAAAGAATTTGAAAGTATTGCTCTTGGGTAATAACGATTTTA  2875073

Query  326      CGATTCTACCCTCCGAACTTTTGTTTCTTCCGTTGATCAAAATTCTATACGTAGATCGGA  385
                |  ||||||| || |||||||||||||| || ||||| ||||  || ||| | ||||  |
Sbjct  2875072  CTTTTCTACCTTCGGAACTTTTGTTTCTGCCTTTGATTAAAACCCTGTACTTGGATCAAA  2875013

Query  386      ATAAATTAACTCTAACGGAAACGGATGTGGAAATTCTCGCTTCTCTTTCCTCCTTGGAGG  445
                | ||  | ||  | || ||||||||||| |||||||| ||||||||||||   ||||| |
Sbjct  2875012  ACAAGCTTACGTTGACTGAAACGGATGTTGAAATTCTAGCTTCTCTTTCCAGTTTGGAAG  2874953

Query  446      AATTGGATCTGAGTCTGAATTCGGGGATTAAGGCGCTCCCTTTTAATTACGAAAAACTTG  505
                |||||||||| | | |||| || || || || | ||| |||| ||||||  | ||| |
Sbjct  2874952  AATTGGATCTTAATTTGAACTCTGGAATCAAAGTGCTTCCTTCTAATTATAATAAATTGA  2874893

Query  506      TAAATCTCATTAATCTAAAAAGATTGAATATTaaaaaaaCTTCATTAAAGGGAGAGGATG  565
                 |||||| | | || ||||||||||||||||||||||||||||||||||||| ||||| |
Sbjct  2874892  AAAATCTGACTCATTTAAAAAGATTGAATATTAAAAAAACTTCATTAAAGGGGGAGGACG  2874833

Query  566      CAGATAAATTGCAGGCGATTCTTCCAAATACCAAAATCGACTACTGA  612
                | |||||||| || ||  |||| |||||||| | ||| ||||| |||
Sbjct  2874832  CTGATAAATTACAAGCAGTTCTCCCAAATACAAGAATAGACTATTGA  2874786



>Leptospira interrogans serovar Manilae strain UP-MMC-NIID HP chromosome 1, complete
sequence
Sequence ID: CP011934.1 Length: 4238922
Range 1: 2850353 to 2850939

Score:473 bits(524), Expect:4e-132,
Identities:457/587(78%),  Gaps:0/587(0%), Strand: Plus/Minus

Query  26       TCGACTGTAAAAAGAATGCGGAAGAGATTTTGGGAGAGGCAAAGGCAAAACCGGAATTGG  85
                |||| |||||||| || |||| ||||||||| | ||| |||||   |||| |||||| |
Sbjct  2850939  TCGATTGTAAAAAAAAACGCGGTAGAGATTTTAGAAGAAGCAAAAAGAAAATCGGAATCGA  2850880

Query  86       TTCAAACTTTGGATTTTGGAATGCAGAAGTTGTCCACGGTTCCGGAAGAAGTTTGCGGTT  145
                ||||||  || ||||| |||||||| |||||  || || |||| |||| |||||||  ||
Sbjct  2850879  TTCAAATCTTAGATTTAGGAATGCAAAAGTTAACCTCGATTCCAGAAGGAGTTTGCTCTT  2850820
```

```
Query  146      TTCCGAATTTAACCAAGTTGGATCTTCGTTTAAACAGTTTGACTTTTCTTCCGGAATTTA  205
                |||||||||| ||  || ||||| | ||||| |||||||||| || |||||| |  |  |
Sbjct  2850819  TTCCGAATTTGACTCAGCTGGATTTACGTTTGAACAGTTTGAATTCTCTTCCTGGTTGGA  2850760

Query  206      TCGGAGAATGTAAACGCCTTGAACAACTTAATCTTTTCGGGAACGACCTTACAACATTTC  265
                | ||||  ||||||   || |||||| |||| |||||||| || || || |  ||  |||
Sbjct  2850759  TTGGAGCTTGTAAAAATCTGGAACAAATTAACCTTTTCGGAAATGATCTAAATACTGTTC  2850700

Query  266      CGTCTACGTTTTCTAAATTAAAAAATCTGAAAGTGTTACTGGCGGGAAATAACGATTTTA  325
                | ||| | |||||||||||||| ||| ||||||| || ||   ||| |||||||||||||
Sbjct  2850699  CATCTTCTTTTTCTAAATTAAAGAATTTGAAAGTATTGCTCTTGGGTAATAACGATTTTA  2850640

Query  326      CGATTCTACCCTCCGAACTTTTGTTTCTTCCGTTGATCAAAATTCTATACGTAGATCGGA  385
                |  |||||||||| |||||||||||||| || ||| | ||||  || ||| | ||||  |
Sbjct  2850639  CTTTTCTACCCTCGGAACTTTTGTTTCTGCCTTTGCTTAAAACCCTGTACTTGGATCAAA  2850580

Query  386      ATAAATTAACTCTAACGGAAACGGATGTGGAAATTCTCGCTTCTCTTTCCTCCTTGGAGG  445
                | ||  | ||  | || ||||||||||| |||||||| |||||||||| |   ||||| |
Sbjct  2850579  ACAAGCTTACGTTGACTGAAACGGATGTTGAAATTCTAGCTTCTCTTTTCAGTTTGGAAG  2850520

Query  446      AATTGGATCTGAGTCTGAATTCGGGGATTAAGGCGCTCCCTTTTAATTACGAAAAACTTG  505
                |||||||||| | | |||| || || || || | ||| |||| ||| ||  | ||| |
Sbjct  2850519  AATTGGATCTTAATTTGAACTCTGGAATCAAAGTGCTTCCTTCTAACTATAATAAATTGA  2850460

Query  506      TAAATCTCATTAATCTAAAAAGATTGAATATTaaaaaaaCTTCATTAAAGGGAGAGGATG  565
                 |||||| | | || ||||||||||||||||||||||||||||||||||||| |||||||
Sbjct  2850459  AAAATCTGACTCATTTAAAAAGATTGAATATTAAAAAAACTTCATTAAAGGGGGAGGATG  2850400

Query  566      CAGATAAATTGCAGGCGATTCTTCCAAATACCAAAATCGACTACTGA  612
                | |||||||| || ||  |||| |||||||| ||||| ||||| |||
Sbjct  2850399  CTGATAAATTACAAGCAGTTCTCCCAAATACAAAAATAGACTATTGA  2850353
```

>Leptospira interrogans serovar Manilae strain UP-MMC-NIID LP chromosome 1, complete sequence
Sequence ID: CP011931.1 Length: 4238972
Range 1: 2850352 to 2850938

Score:473 bits(524), Expect:4e-132,
Identities:457/587(78%), Gaps:0/587(0%), Strand: Plus/Minus

```
Query  26       TCGACTGTAAAAAGAATGCGGAAGAGATTTTGGGAGAGGCAAAGGCAAAACCGGAATTGG  85
                |||| |||||||| || |||| ||||||||| | ||| |||||   |||| |||||| |
Sbjct  2850938  TCGATTGTAAAAAAAACGCGGTAGAGATTTTAGAAGAAGCAAAAAGAAAATCGGAATCGA  2850879

Query  86       TTCAAACTTTGGATTTTGGAATGCAGAAGTTGTCCACGGTTCCGGAAGAAGTTTGCGGTT  145
                ||||||  || ||||| |||||||| |||||  || || |||| |||| |||||||  ||
Sbjct  2850878  TTCAAATCTTAGATTTAGGAATGCAAAAGTTAACCTCGATTCCAGAAGGAGTTTGCTCTT  2850819

Query  146      TTCCGAATTTAACCAAGTTGGATCTTCGTTTAAACAGTTTGACTTTTCTTCCGGAATTTA  205
                |||||||||| ||  || ||||| | ||||| |||||||||| || |||||| |  |  |
Sbjct  2850818  TTCCGAATTTGACTCAGCTGGATTTACGTTTGAACAGTTTGAATTCTCTTCCTGGTTGGA  2850759

Query  206      TCGGAGAATGTAAACGCCTTGAACAACTTAATCTTTTCGGGAACGACCTTACAACATTTC  265
                | ||||  ||||||   || |||||| |||| |||||||| || || || |  ||  |||
Sbjct  2850758  TTGGAGCTTGTAAAAATCTGGAACAAATTAACCTTTTCGGAAATGATCTAAATACTGTTC  2850699

Query  266      CGTCTACGTTTTCTAAATTAAAAAATCTGAAAGTGTTACTGGCGGGAAATAACGATTTTA  325
                | ||| | |||||||||||||| ||| ||||||| || ||   ||| |||||||||||||
Sbjct  2850698  CATCTTCTTTTTCTAAATTAAAGAATTTGAAAGTATTGCTCTTGGGTAATAACGATTTTA  2850639
```

```
Query  326       CGATTCTACCCTCCGAACTTTTGTTTCTTCCGTTGATCAAAATTCTATACGTAGATCGGA  385
                 |  |||||||||| |||||||||||||| || ||| | ||||  || ||| | ||||  |
Sbjct  2850638   CTTTTCTACCCTCGGAACTTTTGTTTCTGCCTTTGCTTAAAACCCTGTACTTGGATCAAA  2850579

Query  386       ATAAATTAACTCTAACGGAAACGGATGTGGAAATTCTCGCTTCTCTTTCCTCCTTGGAGG  445
                 | ||  | ||  | || ||||||||||| |||||||| |||||||||| |   ||||| |
Sbjct  2850578   ACAAGCTTACGTTGACTGAAACGGATGTTGAAATTCTAGCTTCTCTTTTCAGTTTGGAAG  2850519

Query  446       AATTGGATCTGAGTCTGAATTCGGGGATTAAGGCGCTCCCTTTTAATTACGAAAAACTTG  505
                 |||||||||| | | |||| || || || || | ||| |||| ||| ||  | ||| |
Sbjct  2850518   AATTGGATCTTAATTTGAACTCTGGAATCAAAGTGCTTCCTTCTAACTATAATAAATTGA  2850459

Query  506       TAAATCTCATTAATCTAAAAAGATTGAATATTaaaaaaaCTTCATTAAAGGGAGAGGATG  565
                  |||||| | | || ||||||||||||||||||||||||||||||||||||| |||||||
Sbjct  2850458   AAAATCTGACTCATTTAAAAAGATTGAATATTAAAAAAACTTCATTAAAGGGGGAGGATG  2850399

Query  566       CAGATAAATTGCAGGCGATTCTTCCAAATACCAAAATCGACTACTGA  612
                 | |||||||| || ||  |||| |||||||| ||||| ||||| |||
Sbjct  2850398   CTGATAAATTACAAGCAGTTCTCCCAAATACAAAAATAGACTATTGA  2850352
```

>Leptospira interrogans strain FMAS_RT2 chromosome 1, complete sequence
Sequence ID: CP092166.1 Length: 4342805
Range 1: 2179558 to 2180144

Score:473 bits(524), Expect:4e-132,
Identities:457/587(78%), Gaps:0/587(0%), Strand: Plus/Minus

```
Query  26        TCGACTGTAAAAAGAATGCGGAAGAGATTTTGGGAGAGGCAAAGGCAAAACCGGAATTGG  85
                 |||| |||||||| || |||| ||||||||| | ||| |||||   |||| |||||| |
Sbjct  2180144   TCGATTGTAAAAAAAACGCGGTAGAGATTTTAGAAGAAGCAAAAAGAAAATCGGAATCGA  2180085

Query  86        TTCAAACTTTGGATTTTGGAATGCAGAAGTTGTCCACGGTTCCGGAAGAAGTTTGCGGTT  145
                 ||||||  || ||||| |||||||| |||||  || || |||| |||| |||||||  ||
Sbjct  2180084   TTCAAATCTTAGATTTAGGAATGCAAAAGTTAACCTCGATTCCAGAAGGAGTTTGCTCTT  2180025

Query  146       TTCCGAATTTAACCAAGTTGGATCTTCGTTTAAACAGTTTGACTTTTCTTCCGGAATTTA  205
                 |||||||||| ||  || ||||| | ||||| |||||||||| || |||||| |  |  |
Sbjct  2180024   TTCCGAATTTGACTCAGCTGGATTTACGTTTGAACAGTTTGAATTCTCTTCCTGGTTGGA  2179965

Query  206       TCGGAGAATGTAAACGCCTTGAACAACTTAATCTTTTCGGGAACGACCTTACAACATTTC  265
                 | ||||  ||||||   || |||||| |||| |||||||| || || || |  ||  |||
Sbjct  2179964   TTGGAGCTTGTAAAAATCTGGAACAAATTAACCTTTTCGGAAATGATCTAAATACGGTTC  2179905

Query  266       CGTCTACGTTTTCTAAATTAAAAAATCTGAAAGTGTTACTGGCGGGAAATAACGATTTTA  325
                 | ||| | |||||||||||||| ||| ||||||| || ||   ||| |||||||||||||
Sbjct  2179904   CATCTTCTTTTTCTAAATTAAAGAATTTGAAAGTATTGCTCTTGGGTAATAACGATTTTA  2179845

Query  326       CGATTCTACCCTCCGAACTTTTGTTTCTTCCGTTGATCAAAATTCTATACGTAGATCGGA  385
                 |  ||||||| || |||||||||||||| || ||||| ||||  || ||| | ||||  |
Sbjct  2179844   CTTTTCTACCTTCGGAACTTTTGTTTCTGCCTTTGATTAAAACCCTGTACTTGGATCAAA  2179785

Query  386       ATAAATTAACTCTAACGGAAACGGATGTGGAAATTCTCGCTTCTCTTTCCTCCTTGGAGG  445
                 | ||  | ||  | || ||||||||||| |||||||| ||||||||||||   ||||| |
Sbjct  2179784   ACAAGCTTACGTTGACTGAAACGGATGTTGAAATTCTAGCTTCTCTTTCCAGTTTGGAAG  2179725

Query  446       AATTGGATCTGAGTCTGAATTCGGGGATTAAGGCGCTCCCTTTTAATTACGAAAAACTTG  505
                 |||||||||| | | |||| || || || || | ||| |||| ||||||  | ||| |
Sbjct  2179724   AATTGGATCTTAATTTGAACTCTGGAATCAAAGTGCTTCCTTCTAATTATAATAAATTGA  2179665
```

```
Query  506      TAAATCTCATTAATCTAAAAAGATTGAATATTaaaaaaaCTTCATTAAAGGGAGAGGATG  565
                 |||||| | | || ||||||||||||||||||||||||||||||||||||| ||||| |
Sbjct  2179664  AAAATCTGACTCATTTAAAAAGATTGAATATTAAAAAAACTTCATTAAAGGGGGAGGACG  2179605

Query  566      CAGATAAATTGCAGGCGATTCTTCCAAATACCAAAATCGACTACTGA  612
                | |||||||| || ||  |||| |||||||| | ||| ||||| |||
Sbjct  2179604  CTGATAAATTACAAGCAGTTCTCCCAAATACAAGAATAGACTATTGA  2179558
```

>Leptospira interrogans strain FMAS_PN2 chromosome 1, complete sequence
Sequence ID: CP092151.1 Length: 4302184
Range 1: 3441048 to 3441634

Score:473 bits(524), Expect:4e-132,
Identities:457/587(78%), Gaps:0/587(0%), Strand: Plus/Minus

```
Query  26       TCGACTGTAAAAAGAATGCGGAAGAGATTTTGGGAGAGGCAAAGGCAAAACCGGAATTGG  85
                |||| |||||||| || |||| ||||||||| | ||| |||||   |||| |||||| |
Sbjct  3441634  TCGATTGTAAAAAAAACGCGGTAGAGATTTTAGAAGAAGCAAAAAGAAAATCGGAATCGA  3441575

Query  86       TTCAAACTTTGGATTTTGGAATGCAGAAGTTGTCCACGGTTCCGGAAGAAGTTTGCGGTT  145
                ||||||  || ||||| |||||||| |||||  || || |||| |||| |||||||  ||
Sbjct  3441574  TTCAAATCTTAGATTTAGGAATGCAAAAGTTAACCTCGATTCCAGAAGGAGTTTGCTCTT  3441515

Query  146      TTCCGAATTTAACCAAGTTGGATCTTCGTTTAAACAGTTTGACTTTTCTTCCGGAATTTA  205
                |||||||||| ||  || ||||| | ||||| |||||||||| || |||||| |  |  |
Sbjct  3441514  TTCCGAATTTGACTCAGCTGGATTTACGTTTGAACAGTTTGAATTCTCTTCCTGGTTGGA  3441455

Query  206      TCGGAGAATGTAAACGCCTTGAACAACTTAATCTTTTCGGGAACGACCTTACAACATTTC  265
                | ||||  ||||||   || |||||| |||| |||||||| || || || |  ||  |||
Sbjct  3441454  TTGGAGCTTGTAAAAATCTGGAACAAATTAACCTTTTCGGAAATGATCTAAATACGGTTC  3441395

Query  266      CGTCTACGTTTTCTAAATTAAAAAATCTGAAAGTGTTACTGGCGGGAAATAACGATTTTA  325
                | ||| | |||||||||||||| ||| ||||||| || ||   ||| |||||||||||||
Sbjct  3441394  CATCTTCTTTTTCTAAATTAAAGAATTTGAAAGTATTGCTCTTGGGTAATAACGATTTTA  3441335

Query  326      CGATTCTACCCTCCGAACTTTTGTTTCTTCCGTTGATCAAAATTCTATACGTAGATCGGA  385
                |  ||||||| || |||||||||||||| || ||||| ||||  || ||| | ||||  |
Sbjct  3441334  CTTTTCTACCTTCGGAACTTTTGTTTCTGCCTTTGATTAAAACCCTGTACTTGGATCAAA  3441275

Query  386      ATAAATTAACTCTAACGGAAACGGATGTGGAAATTCTCGCTTCTCTTTCCTCCTTGGAGG  445
                | ||  | ||  | || ||||||||||| |||||||| |||||||| |||   ||||| |
Sbjct  3441274  ACAAGCTTACGTTGACCGAAACGGATGTTGAAATTCTAGCTTCTCTCTCCAGTTTGGAAG  3441215

Query  446      AATTGGATCTGAGTCTGAATTCGGGGATTAAGGCGCTCCCTTTTAATTACGAAAAACTTG  505
                |||||||||| | | |||| || || ||||| | ||| |||| ||| ||  | ||| |
Sbjct  3441214  AATTGGATCTTAATTTGAACTCTGGAATTAAAGTGCTTCCTTCTAACTATAATAAATTGA  3441155

Query  506      TAAATCTCATTAATCTAAAAAGATTGAATATTaaaaaaaCTTCATTAAAGGGAGAGGATG  565
                 |||||| | | |||||||||||||||||||||||||||||||||||||||||| ||||
Sbjct  3441154  AAAATCTGACTCATTTAAAAAGATTGAATATTAAAAAAACTTCATTAAAGGGAGAAGATG  3441095

Query  566      CAGATAAATTGCAGGCGATTCTTCCAAATACCAAAATCGACTACTGA  612
                | |||||||| || ||  |||| |||||||| | ||| ||||| |||
Sbjct  3441094  CTGATAAATTACAAGCAGTTCTCCCAAATACAAGAATAGACTATTGA  3441048
```

>Leptospira interrogans serovar Canicola strain 782 chromosome 1, complete sequence
Sequence ID: CP043884.1 Length: 4556406

```
Range 1: 1482000 to 1482586

Score:473 bits(524), Expect:4e-132,
Identities:457/587(78%),  Gaps:0/587(0%), Strand: Plus/Plus

Query  26       TCGACTGTAAAAAGAATGCGGAAGAGATTTTGGGAGAGGCAAAGGCAAAACCGGAATTGG  85
                |||| |||||||| || |||| ||||||||| | ||| |||||   |||| |||||| |
Sbjct  1482000  TCGATTGTAAAAAAAACGCGGTAGAGATTTTAGAAGAAGCAAAAAGAAAATCGGAATCGA  1482059

Query  86       TTCAAACTTTGGATTTTGGAATGCAGAAGTTGTCCACGGTTCCGGAAGAAGTTTGCGGTT  145
                ||||||  || ||||| |||||||| |||||  || || |||| |||| |||||||  ||
Sbjct  1482060  TTCAAATCTTAGATTTAGGAATGCAAAAGTTAACCTCGATTCCAGAAGGAGTTTGCTCTT  1482119

Query  146      TTCCGAATTTAACCAAGTTGGATCTTCGTTTAAACAGTTTGACTTTTCTTCCGGAATTTA  205
                |||||||||| ||  || ||||| | ||||| |||||||||| || |||||| |  |  |
Sbjct  1482120  TTCCGAATTTGACTCAGCTGGATTTACGTTTGAACAGTTTGAATTCTCTTCCTGGTTGGA  1482179

Query  206      TCGGAGAATGTAAACGCCTTGAACAACTTAATCTTTTCGGGAACGACCTTACAACATTTC  265
                | ||||  ||||||   || |||||| |||| |||||||| || || || |  ||  |||
Sbjct  1482180  TTGGAGCTTGTAAAAATCTGGAACAAATTAACCTTTTCGGAAATGATCTAAATACGGTTC  1482239

Query  266      CGTCTACGTTTTCTAAATTAAAAAATCTGAAAGTGTTACTGGCGGGAAATAACGATTTTA  325
                | ||| | |||||||||||||| ||| ||||||| |||||   ||| |||||||||||||
Sbjct  1482240  CATCTTCTTTTTCTAAATTAAAGAATTTGAAAGTATTACTCTTGGGTAATAACGATTTTA  1482299

Query  326      CGATTCTACCCTCCGAACTTTTGTTTCTTCCGTTGATCAAAATTCTATACGTAGATCGGA  385
                |  ||||||| || |||||||||||||| || ||| | ||||  || ||  | ||||  |
Sbjct  1482300  CTTTTCTACCTTCGGAACTTTTGTTTCTGCCTTTGCTTAAAACCCTGTATTTGGATCAAA  1482359

Query  386      ATAAATTAACTCTAACGGAAACGGATGTGGAAATTCTCGCTTCTCTTTCCTCCTTGGAGG  445
                | || || ||  | || ||||||||||| |||||||| ||||||||||||   ||||| |
Sbjct  1482360  ACAAGTTTACGTTGACTGAAACGGATGTTGAAATTCTAGCTTCTCTTTCCAGTTTGGAAG  1482419

Query  446      AATTGGATCTGAGTCTGAATTCGGGGATTAAGGCGCTCCCTTTTAATTACGAAAAACTTG  505
                |||||||||| | | |||| || || || || | ||| |||| ||| ||  | ||| |
Sbjct  1482420  AATTGGATCTTAATTTGAACTCTGGAATCAAAGTGCTTCCTTCTAACTATAATAAATTGA  1482479

Query  506      TAAATCTCATTAATCTAAAAAGATTGAATATTaaaaaaaCTTCATTAAAGGGAGAGGATG  565
                 |||||| | | || ||||||||||||||||||||||||||||||||||||| |||||||
Sbjct  1482480  AAAATCTGACTCATTTAAAAAGATTGAATATTAAAAAAACTTCATTAAAGGGGGAGGATG  1482539

Query  566      CAGATAAATTGCAGGCGATTCTTCCAAATACCAAAATCGACTACTGA  612
                | |||||||| || ||  |||| |||||||| | ||| ||||| |||
Sbjct  1482540  CTGATAAATTACAAGCAGTTCTCCCAAATACAAGAATAGACTATTGA  1482586



>Leptospira interrogans serovar Copenhageni strain SK1 chromosome I, complete sequence
Sequence ID: CP048830.1 Length: 4279936
Range 1: 2909396 to 2909982

Score:469 bits(519), Expect:2e-130,
Identities:456/587(78%),  Gaps:0/587(0%), Strand: Plus/Minus

Query  26       TCGACTGTAAAAAGAATGCGGAAGAGATTTTGGGAGAGGCAAAGGCAAAACCGGAATTGG  85
                |||| |||||||| || |||| ||||||||| | ||| |||||   |||| |||||| |
Sbjct  2909982  TCGATTGTAAAAAAAACGCGGTAGAGATTTTAGAAGAAGCAAAAAGAAAATCGGAATCGA  2909923

Query  86       TTCAAACTTTGGATTTTGGAATGCAGAAGTTGTCCACGGTTCCGGAAGAAGTTTGCGGTT  145
                ||||||  || ||||| |||||||| |||||  || || |||| |||| |||||||  ||
Sbjct  2909922  TTCAAATCTTAGATTTAGGAATGCAAAAGTTAACCTCGATTCCAGAAGGAGTTTGCTCTT  2909863
```

```
Query  146      TTCCGAATTTAACCAAGTTGGATCTTCGTTTAAACAGTTTGACTTTTCTTCCGGAATTTA  205
                |||||||||| ||  || ||||| | ||||| |||||||||| || |||||| |  |  |
Sbjct  2909862  TTCCGAATTTGACTCAGCTGGATTTACGTTTGAACAGTTTGAATTCTCTTCCTGGTTGGA  2909803

Query  206      TCGGAGAATGTAAACGCCTTGAACAACTTAATCTTTTCGGGAACGACCTTACAACATTTC  265
                | ||||  ||||||   || |||||| |||| |||||||| || || || |  ||  |||
Sbjct  2909802  TTGGAGCTTGTAAAAATCTGGAACAAATTAACCTTTTCGGAAATGATCTAAATACGGTTC  2909743

Query  266      CGTCTACGTTTTCTAAATTAAAAAATCTGAAAGTGTTACTGGCGGGAAATAACGATTTTA  325
                | ||| | |||||||||||||| ||| ||||||| |||||   ||| |||||||||||||
Sbjct  2909742  CATCTTCTTTTTCTAAATTAAAGAATTTGAAAGTATTACTCTTGGGTAATAACGATTTTA  2909683

Query  326      CGATTCTACCCTCCGAACTTTTGTTTCTTCCGTTGATCAAAATTCTATACGTAGATCGGA  385
                |  ||||||| || |||||||||||||| || ||| | ||||  || ||  | ||||  |
Sbjct  2909682  CTTTTCTACCTTCGGAACTTTTGTTTCTGCCTTTGCTTAAAACCCTGTATTTGGATCAAA  2909623

Query  386      ATAAATTAACTCTAACGGAAACGGATGTGGAAATTCTCGCTTCTCTTTCCTCCTTGGAGG  445
                | ||  | ||  | || ||||||||||| |||||||| ||||||||||||   ||||| |
Sbjct  2909622  ACAAGCTTACGTTGACTGAAACGGATGTTGAAATTCTAGCTTCTCTTTCCAGTTTGGAAG  2909563

Query  446      AATTGGATCTGAGTCTGAATTCGGGGATTAAGGCGCTCCCTTTTAATTACGAAAAACTTG  505
                |||||||||| | | |||| || || || || | ||| |||| ||| ||  | ||| |
Sbjct  2909562  AATTGGATCTTAATTTGAACTCTGGAATCAAAGTGCTTCCTTCTAACTATAATAAATTGA  2909503

Query  506      TAAATCTCATTAATCTAAAAAGATTGAATATTaaaaaaaCTTCATTAAAGGGAGAGGATG  565
                 |||||| | | || ||||||||||||||||||||||||||||||||||||| |||||||
Sbjct  2909502  AAAATCTGACTCATTTAAAAAGATTGAATATTAAAAAAACTTCATTAAAGGGGGAGGATG  2909443

Query  566      CAGATAAATTGCAGGCGATTCTTCCAAATACCAAAATCGACTACTGA  612
                | |||||||| || ||  |||| |||||||| | ||| ||||| |||
Sbjct  2909442  CTGATAAATTACAAGCAGTTCTCCCAAATACAAGAATAGACTATTGA  2909396
```

>Leptospira interrogans strain R12 chromosome 1
Sequence ID: CP047508.1 Length: 4223477
Range 1: 2872427 to 2873013

Score:469 bits(519), Expect:2e-130,
Identities:456/587(78%), Gaps:0/587(0%), Strand: Plus/Minus

```
Query  26       TCGACTGTAAAAAGAATGCGGAAGAGATTTTGGGAGAGGCAAAGGCAAAACCGGAATTGG  85
                |||| |||||||| || |||| ||||||||| | ||| |||||   |||| |||||| |
Sbjct  2873013  TCGATTGTAAAAAAAACGCGGTAGAGATTTTAGAAGAAGCAAAAAGAAAATCGGAATCGA  2872954

Query  86       TTCAAACTTTGGATTTTGGAATGCAGAAGTTGTCCACGGTTCCGGAAGAAGTTTGCGGTT  145
                ||||||  || ||||| |||||||| |||||  || || |||| |||| |||||||  ||
Sbjct  2872953  TTCAAATCTTAGATTTAGGAATGCAAAAGTTAACCTCGATTCCAGAAGGAGTTTGCTCTT  2872894

Query  146      TTCCGAATTTAACCAAGTTGGATCTTCGTTTAAACAGTTTGACTTTTCTTCCGGAATTTA  205
                |||||||||| ||  || ||||| | ||||| |||||||||| || |||||| |  |  |
Sbjct  2872893  TTCCGAATTTGACTCAGCTGGATTTACGTTTGAACAGTTTGAATTCTCTTCCTGGTTGGA  2872834

Query  206      TCGGAGAATGTAAACGCCTTGAACAACTTAATCTTTTCGGGAACGACCTTACAACATTTC  265
                | ||||  ||||||   || |||||| |||| |||||||| || || || |  ||  |||
Sbjct  2872833  TTGGAGCTTGTAAAAATCTGGAACAAATTAACCTTTTCGGAAATGATCTAAATACGGTTC  2872774

Query  266      CGTCTACGTTTTCTAAATTAAAAAATCTGAAAGTGTTACTGGCGGGAAATAACGATTTTA  325
                | ||| | |||||||||||||| ||| ||||||| |||||   ||| |||||||||||||
Sbjct  2872773  CATCTTCTTTTTCTAAATTAAAGAATTTGAAAGTATTACTCTTGGGTAATAACGATTTTA  2872714

Query  326      CGATTCTACCCTCCGAACTTTTGTTTCTTCCGTTGATCAAAATTCTATACGTAGATCGGA  385
```

```
                |  ||||||| || |||||||||||||| || ||| | ||||  || ||  | ||||  |
Sbjct  2872713  CTTTTCTACCTTCGGAACTTTTGTTTCTGCCTTTGCTTAAAACCCTGTATTTGGATCAAA  2872654

Query  386      ATAAATTAACTCTAACGGAAACGGATGTGGAAATTCTCGCTTCTCTTTCCTCCTTGGAGG  445
                | ||  | ||  | || ||||||||||| |||||||| ||||||||||||   ||||| |
Sbjct  2872653  ACAAGCTTACGTTGACTGAAACGGATGTTGAAATTCTAGCTTCTCTTTCCAGTTTGGAAG  2872594

Query  446      AATTGGATCTGAGTCTGAATTCGGGGATTAAGGCGCTCCCTTTTAATTACGAAAAACTTG  505
                |||||||||| | | |||| || || || || | ||| |||| ||| ||  | ||| |
Sbjct  2872593  AATTGGATCTTAATTTGAACTCTGGAATCAAAGTGCTTCCTTCTAACTATAATAAATTGA  2872534

Query  506      TAAATCTCATTAATCTAAAAAGATTGAATATTaaaaaaaCTTCATTAAAGGGAGAGGATG  565
                 |||||| | | || ||||||||||||||||||||||||||||||||||||| |||||||
Sbjct  2872533  AAAATCTGACTCATTTAAAAAGATTGAATATTAAAAAAACTTCATTAAAGGGGGAGGATG  2872474

Query  566      CAGATAAATTGCAGGCGATTCTTCCAAATACCAAAATCGACTACTGA  612
                | |||||||| || ||  |||| |||||||| | ||| ||||| |||
Sbjct  2872473  CTGATAAATTACAAGCAGTTCTCCCAAATACAAGAATAGACTATTGA  2872427
```

```
>Leptospira interrogans strain R19 chromosome 1
Sequence ID: CP047514.1 Length: 4213431
Range 1: 2873903 to 2874489

Score:469 bits(519), Expect:2e-130,
Identities:456/587(78%),  Gaps:0/587(0%), Strand: Plus/Minus

Query  26       TCGACTGTAAAAAGAATGCGGAAGAGATTTTGGGAGAGGCAAAGGCAAAACCGGAATTGG  85
                |||| |||||||| || |||| ||||||||| | ||| |||||   |||| |||||| |
Sbjct  2874489  TCGATTGTAAAAAAAACGCGGTAGAGATTTTAGAAGAAGCAAAAAGAAAATCGGAATCGA  2874430

Query  86       TTCAAACTTTGGATTTTGGAATGCAGAAGTTGTCCACGGTTCCGGAAGAAGTTTGCGGTT  145
                ||||||  || ||||| |||||||| |||||  || || |||| |||| |||||||  ||
Sbjct  2874429  TTCAAATCTTAGATTTAGGAATGCAAAAGTTAACCTCGATTCCAGAAGGAGTTTGCTCTT  2874370

Query  146      TTCCGAATTTAACCAAGTTGGATCTTCGTTTAAACAGTTTGACTTTTCTTCCGGAATTTA  205
                |||||||||| ||  || ||||| | ||||| |||||||||| || |||||| |  |  |
Sbjct  2874369  TTCCGAATTTGACTCAGCTGGATTTACGTTTGAACAGTTTGAATTCTCTTCCTGGTTGGA  2874310

Query  206      TCGGAGAATGTAAACGCCTTGAACAACTTAATCTTTTCGGGAACGACCTTACAACATTTC  265
                | ||||  ||||||   || |||||| |||| |||||||| || || || |  ||  |||
Sbjct  2874309  TTGGAGCTTGTAAAAATCTGGAACAAATTAACCTTTTCGGAAATGATCTAAATACGGTTC  2874250

Query  266      CGTCTACGTTTTCTAAATTAAAAAATCTGAAAGTGTTACTGGCGGGAAATAACGATTTTA  325
                | ||| | |||||||||||||| ||| ||||||| |||||   ||| |||||||||||||
Sbjct  2874249  CATCTTCTTTTTCTAAATTAAAGAATTTGAAAGTATTACTCTTGGGTAATAACGATTTTA  2874190

Query  326      CGATTCTACCCTCCGAACTTTTGTTTCTTCCGTTGATCAAAATTCTATACGTAGATCGGA  385
                |  ||||||| || |||||||||||||| || ||| | ||||  || ||  | ||||  |
Sbjct  2874189  CTTTTCTACCTTCGGAACTTTTGTTTCTGCCTTTGCTTAAAACCCTGTATTTGGATCAAA  2874130

Query  386      ATAAATTAACTCTAACGGAAACGGATGTGGAAATTCTCGCTTCTCTTTCCTCCTTGGAGG  445
                | ||  | ||  | || ||||||||||| |||||||| ||||||||||||   ||||| |
Sbjct  2874129  ACAAGCTTACGTTGACTGAAACGGATGTTGAAATTCTAGCTTCTCTTTCCAGTTTGGAAG  2874070

Query  446      AATTGGATCTGAGTCTGAATTCGGGGATTAAGGCGCTCCCTTTTAATTACGAAAAACTTG  505
                |||||||||| | | |||| || || || || | ||| |||| ||| ||  | ||| |
Sbjct  2874069  AATTGGATCTTAATTTGAACTCTGGAATCAAAGTGCTTCCTTCTAACTATAATAAATTGA  2874010

Query  506      TAAATCTCATTAATCTAAAAAGATTGAATATTaaaaaaaCTTCATTAAAGGGAGAGGATG  565
                 |||||| | | || ||||||||||||||||||||||||||||||||||||| |||||||
```

```
Sbjct  2874009  AAAATCTGACTCATTTAAAAAGATTGAATATTAAAAAAACTTCATTAAAGGGGGAGGATG  2873950

Query  566      CAGATAAATTGCAGGCGATTCTTCCAAATACCAAAATCGACTACTGA  612
                | |||||||| || ||  |||| |||||||| | ||| ||||| |||
Sbjct  2873949  CTGATAAATTACAAGCAGTTCTCCCAAATACAAGAATAGACTATTGA  2873903
```

>Leptospira interrogans strain R7 chromosome 1
Sequence ID: CP047512.1 Length: 4212939
Range 1: 2864077 to 2864663

Score:469 bits(519), Expect:2e-130,
Identities:456/587(78%), Gaps:0/587(0%), Strand: Plus/Minus

```
Query  26       TCGACTGTAAAAAGAATGCGGAAGAGATTTTGGGAGAGGCAAAGGCAAAACCGGAATTGG  85
                |||| |||||||| || |||| ||||||||| | ||| |||||   |||| |||||| |
Sbjct  2864663  TCGATTGTAAAAAAAACGCGGTAGAGATTTTAGAAGAAGCAAAAAGAAAATCGGAATCGA  2864604

Query  86       TTCAAACTTTGGATTTTGGAATGCAGAAGTTGTCCACGGTTCCGGAAGAAGTTTGCGGTT  145
                ||||||  || ||||| |||||||| |||||  || || |||| |||| |||||||  ||
Sbjct  2864603  TTCAAATCTTAGATTTAGGAATGCAAAAGTTAACCTCGATTCCAGAAGGAGTTTGCTCTT  2864544

Query  146      TTCCGAATTTAACCAAGTTGGATCTTCGTTTAAACAGTTTGACTTTTCTTCCGGAATTTA  205
                |||||||||| ||  || ||||| | ||||| |||||||||| || |||||| |  |  |
Sbjct  2864543  TTCCGAATTTGACTCAGCTGGATTTACGTTTGAACAGTTTGAATTCTCTTCCTGGTTGGA  2864484

Query  206      TCGGAGAATGTAAACGCCTTGAACAACTTAATCTTTTCGGGAACGACCTTACAACATTTC  265
                | ||||  ||||||   || |||||| |||| |||||||| || || || |  ||  |||
Sbjct  2864483  TTGGAGCTTGTAAAAATCTGGAACAAATTAACCTTTTCGGAAATGATCTAAATACGGTTC  2864424

Query  266      CGTCTACGTTTTCTAAATTAAAAAATCTGAAAGTGTTACTGGCGGGAAATAACGATTTTA  325
                | ||| | ||||||||||||| ||| ||||||| |||||   ||| |||||||||||||
Sbjct  2864423  CATCTTCTTTTTCTAAATTAAAGAATTTGAAAGTATTACTCTTGGGTAATAACGATTTTA  2864364

Query  326      CGATTCTACCCTCCGAACTTTTGTTTCTTCCGTTGATCAAAATTCTATACGTAGATCGGA  385
                |  ||||||| || |||||||||||||| || ||| | ||||  || ||  | ||||  |
Sbjct  2864363  CTTTTCTACCTTCGGAACTTTTGTTTCTGCCTTTGCTTAAAACCCTGTATTTGGATCAAA  2864304

Query  386      ATAAATTAACTCTAACGGAAACGGATGTGGAAATTCTCGCTTCTCTTTCCTCCTTGGAGG  445
                | ||  | ||  | || ||||||||||| |||||||| |||||||||||   ||||| |
Sbjct  2864303  ACAAGCTTACGTTGACTGAAACGGATGTTGAAATTCTAGCTTCTCTTTCCAGTTTGGAAG  2864244

Query  446      AATTGGATCTGAGTCTGAATTCGGGGATTAAGGCGCTCCCTTTTAATTACGAAAAACTTG  505
                |||||||||| | | |||| || || || || | ||| |||| ||| ||  | ||| |
Sbjct  2864243  AATTGGATCTTAATTTGAACTCTGGAATCAAAGTGCTTCCTTCTAACTATAATAAATTGA  2864184

Query  506      TAAATCTCATTAATCTAAAAAGATTGAATATTaaaaaaaCTTCATTAAAGGGAGAGGATG  565
                 |||||| | | || |||||||||||||||||||||||||||||||||||||| |||||||
Sbjct  2864183  AAAATCTGACTCATTTAAAAAGATTGAATATTAAAAAAACTTCATTAAAGGGGGAGGATG  2864124

Query  566      CAGATAAATTGCAGGCGATTCTTCCAAATACCAAAATCGACTACTGA  612
                | |||||||| || ||  |||| |||||||| | ||| ||||| |||
Sbjct  2864123  CTGATAAATTACAAGCAGTTCTCCCAAATACAAGAATAGACTATTGA  2864077
```

>Leptospira interrogans strain R11 chromosome 1
Sequence ID: CP047510.1 Length: 4214328
Range 1: 2865468 to 2866054

```
Score:469 bits(519), Expect:2e-130,
Identities:456/587(78%),  Gaps:0/587(0%), Strand: Plus/Minus

Query  26       TCGACTGTAAAAAGAATGCGGAAGAGATTTTGGGAGAGGCAAAGGCAAAACCGGAATTGG  85
                |||| |||||||| || |||| ||||||||| | ||| |||||   |||| |||||| |
Sbjct  2866054  TCGATTGTAAAAAAAACGCGGTAGAGATTTTAGAAGAAGCAAAAAGAAAATCGGAATCGA  2865995

Query  86       TTCAAACTTTGGATTTTGGAATGCAGAAGTTGTCCACGGTTCCGGAAGAAGTTTGCGGTT  145
                ||||||  || ||||| |||||||| |||||  || || |||| |||| |||||||  ||
Sbjct  2865994  TTCAAATCTTAGATTTAGGAATGCAAAAGTTAACCTCGATTCCAGAAGGAGTTTGCTCTT  2865935

Query  146      TTCCGAATTTAACCAAGTTGGATCTTCGTTTAAACAGTTTGACTTTTCTTCCGGAATTTA  205
                |||||||||| ||  || ||||| | ||||| |||||||||| || |||||| |  |  |
Sbjct  2865934  TTCCGAATTTGACTCAGCTGGATTTACGTTTGAACAGTTTGAATTCTCTTCCTGGTTGGA  2865875

Query  206      TCGGAGAATGTAAACGCCTTGAACAACTTAATCTTTTCGGGAACGACCTTACAACATTTC  265
                | ||||  ||||||   || |||||| |||| |||||||| || || || |  ||  |||
Sbjct  2865874  TTGGAGCTTGTAAAAATCTGGAACAAATTAACCTTTTCGGAAATGATCTAAATACGGTTC  2865815

Query  266      CGTCTACGTTTTCTAAATTAAAAAATCTGAAAGTGTTACTGGCGGGAAATAACGATTTTA  325
                | ||| | |||||||||||||| ||| ||||||| |||||   ||| |||||||||||||
Sbjct  2865814  CATCTTCTTTTTCTAAATTAAAGAATTTGAAAGTATTACTCTTGGGTAATAACGATTTTA  2865755

Query  326      CGATTCTACCCTCCGAACTTTTGTTTCTTCCGTTGATCAAAATTCTATACGTAGATCGGA  385
                |  ||||||| || |||||||||||||| || ||| | ||||  || ||  | ||||  |
Sbjct  2865754  CTTTTCTACCTTCGGAACTTTTGTTTCTGCCTTTGCTTAAAACCCTGTATTTGGATCAAA  2865695

Query  386      ATAAATTAACTCTAACGGAAACGGATGTGGAAATTCTCGCTTCTCTTTCCTCCTTGGAGG  445
                | ||  | ||  | || ||||||||||| |||||||| ||||||||||||   ||||| |
Sbjct  2865694  ACAAGCTTACGTTGACTGAAACGGATGTTGAAATTCTAGCTTCTCTTTCCAGTTTGGAAG  2865635

Query  446      AATTGGATCTGAGTCTGAATTCGGGGATTAAGGCGCTCCCTTTTAATTACGAAAAACTTG  505
                |||||||||| | | |||| || || || || | ||| |||| ||| ||  | ||| |
Sbjct  2865634  AATTGGATCTTAATTTGAACTCTGGAATCAAAGTGCTTCCTTCTAACTATAATAAATTGA  2865575

Query  506      TAAATCTCATTAATCTAAAAAGATTGAATATTaaaaaaaCTTCATTAAAGGGAGAGGATG  565
                 |||||| | | || ||||||||||||||||||||||||||||||||||||| |||||||
Sbjct  2865574  AAAATCTGACTCATTTAAAAAGATTGAATATTAAAAAAACTTCATTAAAGGGGGAGGATG  2865515

Query  566      CAGATAAATTGCAGGCGATTCTTCCAAATACCAAAATCGACTACTGA  612
                | |||||||| || ||  |||| |||||||| | ||| ||||| |||
Sbjct  2865514  CTGATAAATTACAAGCAGTTCTCCCAAATACAAGAATAGACTATTGA  2865468



>Leptospira interrogans strain R13-L chromosome 1
Sequence ID: CP047518.1 Length: 4233607
Range 1: 2882682 to 2883268

Score:469 bits(519), Expect:2e-130,
Identities:456/587(78%),  Gaps:0/587(0%), Strand: Plus/Minus

Query  26       TCGACTGTAAAAAGAATGCGGAAGAGATTTTGGGAGAGGCAAAGGCAAAACCGGAATTGG  85
                |||| |||||||| || |||| ||||||||| | ||| |||||   |||| |||||| |
Sbjct  2883268  TCGATTGTAAAAAAAACGCGGTAGAGATTTTAGAAGAAGCAAAAAGAAAATCGGAATCGA  2883209

Query  86       TTCAAACTTTGGATTTTGGAATGCAGAAGTTGTCCACGGTTCCGGAAGAAGTTTGCGGTT  145
                ||||||  || ||||| |||||||| |||||  || || |||| |||| |||||||  ||
Sbjct  2883208  TTCAAATCTTAGATTTAGGAATGCAAAAGTTAACCTCGATTCCAGAAGGAGTTTGCTCTT  2883149

Query  146      TTCCGAATTTAACCAAGTTGGATCTTCGTTTAAACAGTTTGACTTTTCTTCCGGAATTTA  205
                |||||||||| ||  || ||||| | ||||| |||||||||| || |||||| |  |  |
```

```
Sbjct  2883148  TTCCGAATTTGACTCAGCTGGATTTACGTTTGAACAGTTTGAATTCTCTTCCTGGTTGGA  2883089

Query  206      TCGGAGAATGTAAACGCCTTGAACAACTTAATCTTTTCGGGAACGACCTTACAACATTTC  265
                | ||||  ||||||   || |||||| |||| |||||||| || || || |  ||  |||
Sbjct  2883088  TTGGAGCTTGTAAAAATCTGGAACAAATTAACCTTTTCGGAAATGATCTAAATACGGTTC  2883029

Query  266      CGTCTACGTTTTCTAAATTAAAAAATCTGAAAGTGTTACTGGCGGGAAATAACGATTTTA  325
                | ||| | |||||||||||||| ||| ||||||| |||||   ||| |||||||||||||
Sbjct  2883028  CATCTTCTTTTTCTAAATTAAAGAATTTGAAAGTATTACTCTTGGGTAATAACGATTTTA  2882969

Query  326      CGATTCTACCCTCCGAACTTTTGTTTCTTCCGTTGATCAAAATTCTATACGTAGATCGGA  385
                |  ||||||| || |||||||||||||| || ||| | ||||  || ||  | ||||  |
Sbjct  2882968  CTTTTCTACCTTCGGAACTTTTGTTTCTGCCTTTGCTTAAAACCCTGTATTTGGATCAAA  2882909

Query  386      ATAAATTAACTCTAACGGAAACGGATGTGGAAATTCTCGCTTCTCTTTCCTCCTTGGAGG  445
                | ||  | ||  | || ||||||||||| |||||||| ||||||||||||   ||||| |
Sbjct  2882908  ACAAGCTTACGTTGACTGAAACGGATGTTGAAATTCTAGCTTCTCTTTCCAGTTTGGAAG  2882849

Query  446      AATTGGATCTGAGTCTGAATTCGGGGATTAAGGCGCTCCCTTTTAATTACGAAAAACTTG  505
                |||||||||| | | |||| || || || || | ||| |||| ||| ||  | ||| |
Sbjct  2882848  AATTGGATCTTAATTTGAACTCTGGAATCAAAGTGCTTCCTTCTAACTATAATAAATTGA  2882789

Query  506      TAAATCTCATTAATCTAAAAAGATTGAATATTaaaaaaaCTTCATTAAAGGGAGAGGATG  565
                 |||||| | | || ||||||||||||||||||||||||||||||||||||| |||||||
Sbjct  2882788  AAAATCTGACTCATTTAAAAAGATTGAATATTAAAAAAACTTCATTAAAGGGGGAGGATG  2882729

Query  566      CAGATAAATTGCAGGCGATTCTTCCAAATACCAAAATCGACTACTGA  612
                | |||||||| || ||  |||| |||||||| | ||| ||||| |||
Sbjct  2882728  CTGATAAATTACAAGCAGTTCTCCCAAATACAAGAATAGACTATTGA  2882682
```

>Leptospira interrogans strain R17 chromosome 1
Sequence ID: CP047500.1 Length: 4213733
Range 1: 2874834 to 2875420

Score:469 bits(519), Expect:2e-130,
Identities:456/587(78%), Gaps:0/587(0%), Strand: Plus/Minus

```
Query  26       TCGACTGTAAAAAGAATGCGGAAGAGATTTTGGGAGAGGCAAAGGCAAAACCGGAATTGG  85
                |||| |||||||| || |||| ||||||||| | ||| |||||   |||| |||||| |
Sbjct  2875420  TCGATTGTAAAAAAAACGCGGTAGAGATTTTAGAAGAAGCAAAAAGAAAATCGGAATCGA  2875361

Query  86       TTCAAACTTTGGATTTTGGAATGCAGAAGTTGTCCACGGTTCCGGAAGAAGTTTGCGGTT  145
                ||||||  || ||||| |||||||| |||||  || || |||| |||| |||||||  ||
Sbjct  2875360  TTCAAATCTTAGATTTAGGAATGCAAAAGTTAACCTCGATTCCAGAAGGAGTTTGCTCTT  2875301

Query  146      TTCCGAATTTAACCAAGTTGGATCTTCGTTTAAACAGTTTGACTTTTCTTCCGGAATTTA  205
                |||||||||| ||  || ||||| | ||||| |||||||||| || |||||| |  |  |
Sbjct  2875300  TTCCGAATTTGACTCAGCTGGATTTACGTTTGAACAGTTTGAATTCTCTTCCTGGTTGGA  2875241

Query  206      TCGGAGAATGTAAACGCCTTGAACAACTTAATCTTTTCGGGAACGACCTTACAACATTTC  265
                | ||||  ||||||   || |||||| |||| |||||||| || || || |  ||  |||
Sbjct  2875240  TTGGAGCTTGTAAAAATCTGGAACAAATTAACCTTTTCGGAAATGATCTAAATACGGTTC  2875181

Query  266      CGTCTACGTTTTCTAAATTAAAAAATCTGAAAGTGTTACTGGCGGGAAATAACGATTTTA  325
                | ||| | |||||||||||||| ||| ||||||| |||||   ||| |||||||||||||
Sbjct  2875180  CATCTTCTTTTTCTAAATTAAAGAATTTGAAAGTATTACTCTTGGGTAATAACGATTTTA  2875121

Query  326      CGATTCTACCCTCCGAACTTTTGTTTCTTCCGTTGATCAAAATTCTATACGTAGATCGGA  385
                |  ||||||| || |||||||||||||| || ||| | ||||  || ||  | ||||  |
Sbjct  2875120  CTTTTCTACCTTCGGAACTTTTGTTTCTGCCTTTGCTTAAAACCCTGTATTTGGATCAAA  2875061
```

```
Query  386      ATAAATTAACTCTAACGGAAACGGATGTGGAAATTCTCGCTTCTCTTTCCTCCTTGGAGG  445
                | ||  | ||  | || ||||||||||| |||||||| ||||||||||||   ||||| |
Sbjct  2875060  ACAAGCTTACGTTGACTGAAACGGATGTTGAAATTCTAGCTTCTCTTTCCAGTTTGGAAG  2875001

Query  446      AATTGGATCTGAGTCTGAATTCGGGGATTAAGGCGCTCCCTTTTAATTACGAAAAACTTG  505
                |||||||||| | | |||| || || || || | ||| |||| ||| ||  | ||| |
Sbjct  2875000  AATTGGATCTTAATTTGAACTCTGGAATCAAAGTGCTTCCTTCTAACTATAATAAATTGA  2874941

Query  506      TAAATCTCATTAATCTAAAAAGATTGAATATTaaaaaaaCTTCATTAAAGGGAGAGGATG  565
                 |||||| | | || ||||||||||||||||||||||||||||||||||||| |||||||
Sbjct  2874940  AAAATCTGACTCATTTAAAAAGATTGAATATTAAAAAAACTTCATTAAAGGGGGAGGATG  2874881

Query  566      CAGATAAATTGCAGGCGATTCTTCCAAATACCAAAATCGACTACTGA  612
                | |||||||| || ||  |||| |||||||| | ||| ||||| |||
Sbjct  2874880  CTGATAAATTACAAGCAGTTCTCCCAAATACAAGAATAGACTATTGA  2874834
```

```
>Leptospira interrogans strain R13 chromosome 1
Sequence ID: CP047506.1 Length: 4214175
Range 1: 2865293 to 2865879

Score:469 bits(519), Expect:2e-130,
Identities:456/587(78%),  Gaps:0/587(0%), Strand: Plus/Minus

Query  26       TCGACTGTAAAAAGAATGCGGAAGAGATTTTGGGAGAGGCAAAGGCAAAACCGGAATTGG  85
                |||| |||||||| || |||| ||||||||| | ||| |||||   |||| |||||| |
Sbjct  2865879  TCGATTGTAAAAAAAACGCGGTAGAGATTTTAGAAGAAGCAAAAAGAAAATCGGAATCGA  2865820

Query  86       TTCAAACTTTGGATTTTGGAATGCAGAAGTTGTCCACGGTTCCGGAAGAAGTTTGCGGTT  145
                ||||||  || ||||| |||||||| |||||  || || |||| |||| |||||||  ||
Sbjct  2865819  TTCAAATCTTAGATTTAGGAATGCAAAAGTTAACCTCGATTCCAGAAGGAGTTTGCTCTT  2865760

Query  146      TTCCGAATTTAACCAAGTTGGATCTTCGTTTAAACAGTTTGACTTTTCTTCCGGAATTTA  205
                |||||||||| ||  || ||||| | ||||| |||||||||| || |||||| |  |  |
Sbjct  2865759  TTCCGAATTTGACTCAGCTGGATTTACGTTTGAACAGTTTGAATTCTCTTCCTGGTTGGA  2865700

Query  206      TCGGAGAATGTAAACGCCTTGAACAACTTAATCTTTTCGGGAACGACCTTACAACATTTC  265
                | ||||  ||||||   || |||||| |||| |||||||| || || || |  ||  |||
Sbjct  2865699  TTGGAGCTTGTAAAAATCTGGAACAAATTAACCTTTTCGGAAATGATCTAAATACGGTTC  2865640

Query  266      CGTCTACGTTTTCTAAATTAAAAAATCTGAAAGTGTTACTGGCGGGAAATAACGATTTTA  325
                | ||| | |||||||||||||| ||| ||||||| |||||   ||| |||||||||||||
Sbjct  2865639  CATCTTCTTTTTCTAAATTAAAGAATTTGAAAGTATTACTCTTGGGTAATAACGATTTTA  2865580

Query  326      CGATTCTACCCTCCGAACTTTTGTTTCTTCCGTTGATCAAAATTCTATACGTAGATCGGA  385
                |  ||||||| || |||||||||||||| || ||| | ||||  || ||  | ||||  |
Sbjct  2865579  CTTTTCTACCTTCGGAACTTTTGTTTCTGCCTTTGCTTAAAACCCTGTATTTGGATCAAA  2865520

Query  386      ATAAATTAACTCTAACGGAAACGGATGTGGAAATTCTCGCTTCTCTTTCCTCCTTGGAGG  445
                | ||  | ||  | || ||||||||||| |||||||| ||||||||||||   ||||| |
Sbjct  2865519  ACAAGCTTACGTTGACTGAAACGGATGTTGAAATTCTAGCTTCTCTTTCCAGTTTGGAAG  2865460

Query  446      AATTGGATCTGAGTCTGAATTCGGGGATTAAGGCGCTCCCTTTTAATTACGAAAAACTTG  505
                |||||||||| | | |||| || || || || | ||| |||| ||| ||  | ||| |
Sbjct  2865459  AATTGGATCTTAATTTGAACTCTGGAATCAAAGTGCTTCCTTCTAACTATAATAAATTGA  2865400

Query  506      TAAATCTCATTAATCTAAAAAGATTGAATATTaaaaaaaCTTCATTAAAGGGAGAGGATG  565
                 |||||| | | || ||||||||||||||||||||||||||||||||||||| |||||||
Sbjct  2865399  AAAATCTGACTCATTTAAAAAGATTGAATATTAAAAAAACTTCATTAAAGGGGGAGGATG  2865340
```

```
Query  566      CAGATAAATTGCAGGCGATTCTTCCAAATACCAAAATCGACTACTGA  612
                | |||||||| || ||  |||| |||||||| | ||| ||||| |||
Sbjct  2865339  CTGATAAATTACAAGCAGTTCTCCCAAATACAAGAATAGACTATTGA  2865293
```

>Leptospira interrogans strain R22 chromosome 1
Sequence ID: CP047496.1 Length: 4206042
Range 1: 2873918 to 2874504

Score:469 bits(519), Expect:2e-130,
Identities:456/587(78%), Gaps:0/587(0%), Strand: Plus/Minus

```
Query  26       TCGACTGTAAAAAGAATGCGGAAGAGATTTTGGGAGAGGCAAAGGCAAAACCGGAATTGG  85
                |||| |||||||| || |||| ||||||||| | ||| |||||   |||| |||||| |
Sbjct  2874504  TCGATTGTAAAAAAAACGCGGTAGAGATTTTAGAAGAAGCAAAAAGAAAATCGGAATCGA  2874445

Query  86       TTCAAACTTTGGATTTTGGAATGCAGAAGTTGTCCACGGTTCCGGAAGAAGTTTGCGGTT  145
                ||||||  || ||||| |||||||| |||||  || || |||| |||| |||||||  ||
Sbjct  2874444  TTCAAATCTTAGATTTAGGAATGCAAAAGTTAACCTCGATTCCAGAAGGAGTTTGCTCTT  2874385

Query  146      TTCCGAATTTAACCAAGTTGGATCTTCGTTTAAACAGTTTGACTTTTCTTCCGGAATTTA  205
                |||||||||| ||  || ||||| | ||||| |||||||||| || |||||| |  |  |
Sbjct  2874384  TTCCGAATTTGACTCAGCTGGATTTACGTTTGAACAGTTTGAATTCTCTTCCTGGTTGGA  2874325

Query  206      TCGGAGAATGTAAACGCCTTGAACAACTTAATCTTTTCGGGAACGACCTTACAACATTTC  265
                | ||||  ||||||   || |||||| |||| |||||||| || || || |  ||  |||
Sbjct  2874324  TTGGAGCTTGTAAAAATCTGGAACAAATTAACCTTTTCGGAAATGATCTAAATACGGTTC  2874265

Query  266      CGTCTACGTTTTCTAAATTAAAAAATCTGAAAGTGTTACTGGCGGGAAATAACGATTTTA  325
                | ||| | |||||||||||||| ||| ||||||| |||||   ||| |||||||||||||
Sbjct  2874264  CATCTTCTTTTTCTAAATTAAAGAATTTGAAAGTATTACTCTTGGGTAATAACGATTTTA  2874205

Query  326      CGATTCTACCCTCCGAACTTTTGTTTCTTCCGTTGATCAAAATTCTATACGTAGATCGGA  385
                |  ||||||| || |||||||||||||| || ||| | ||||  || ||  | ||||  |
Sbjct  2874204  CTTTTCTACCTTCGGAACTTTTGTTTCTGCCTTTGCTTAAAACCCTGTATTTGGATCAAA  2874145

Query  386      ATAAATTAACTCTAACGGAAACGGATGTGGAAATTCTCGCTTCTCTTTCCTCCTTGGAGG  445
                | ||  | ||  | || ||||||||||| |||||||| ||||||||||||   ||||| |
Sbjct  2874144  ACAAGCTTACGTTGACTGAAACGGATGTTGAAATTCTAGCTTCTCTTTCCAGTTTGGAAG  2874085

Query  446      AATTGGATCTGAGTCTGAATTCGGGGATTAAGGCGCTCCCTTTTAATTACGAAAAACTTG  505
                |||||||||| | | |||| || || || || | ||| |||| ||| ||  | ||| |
Sbjct  2874084  AATTGGATCTTAATTTGAACTCTGGAATCAAAGTGCTTCCTTCTAACTATAATAAATTGA  2874025

Query  506      TAAATCTCATTAATCTAAAAAGATTGAATATTaaaaaaaCTTCATTAAAGGGAGAGGATG  565
                 |||||| | | || ||||||||||||||||||||||||||||||||||||| |||||||
Sbjct  2874024  AAAATCTGACTCATTTAAAAAGATTGAATATTAAAAAAACTTCATTAAAGGGGGAGGATG  2873965

Query  566      CAGATAAATTGCAGGCGATTCTTCCAAATACCAAAATCGACTACTGA  612
                | |||||||| || ||  |||| |||||||| | ||| ||||| |||
Sbjct  2873964  CTGATAAATTACAAGCAGTTCTCCCAAATACAAGAATAGACTATTGA  2873918
```

>Leptospira interrogans strain R21 chromosome 1
Sequence ID: CP047498.1 Length: 4214555
Range 1: 2874543 to 2875129

Score:469 bits(519), Expect:2e-130,
Identities:456/587(78%), Gaps:0/587(0%), Strand: Plus/Minus

```
Query  26       TCGACTGTAAAAAGAATGCGGAAGAGATTTTGGGAGAGGCAAAGGCAAAACCGGAATTGG  85
                |||| |||||||| || |||| ||||||||| | ||| |||||   |||| |||||| |
Sbjct  2875129  TCGATTGTAAAAAAAACGCGGTAGAGATTTTAGAAGAAGCAAAAAGAAAATCGGAATCGA  2875070

Query  86       TTCAAACTTTGGATTTTGGAATGCAGAAGTTGTCCACGGTTCCGGAAGAAGTTTGCGGTT  145
                ||||||  || ||||| |||||||| |||||  || || |||| |||| |||||||  ||
Sbjct  2875069  TTCAAATCTTAGATTTAGGAATGCAAAAGTTAACCTCGATTCCAGAAGGAGTTTGCTCTT  2875010

Query  146      TTCCGAATTTAACCAAGTTGGATCTTCGTTTAAACAGTTTGACTTTTCTTCCGGAATTTA  205
                |||||||||| ||  || ||||| | ||||| |||||||||| || |||||| |  |  |
Sbjct  2875009  TTCCGAATTTGACTCAGCTGGATTTACGTTTGAACAGTTTGAATTCTCTTCCTGGTTGGA  2874950

Query  206      TCGGAGAATGTAAACGCCTTGAACAACTTAATCTTTTCGGGAACGACCTTACAACATTTC  265
                | ||||  ||||||   || |||||| |||| |||||||| || || || |  ||  |||
Sbjct  2874949  TTGGAGCTTGTAAAAATCTGGAACAAATTAACCTTTTCGGAAATGATCTAAATACGGTTC  2874890

Query  266      CGTCTACGTTTTCTAAATTAAAAAATCTGAAAGTGTTACTGGCGGGAAATAACGATTTTA  325
                | ||| | |||||||||||||| ||| ||||||| |||||   ||| |||||||||||||
Sbjct  2874889  CATCTTCTTTTTCTAAATTAAAGAATTTGAAAGTATTACTCTTGGGTAATAACGATTTTA  2874830

Query  326      CGATTCTACCCTCCGAACTTTTGTTTCTTCCGTTGATCAAAATTCTATACGTAGATCGGA  385
                |  ||||||| || |||||||||||||| || ||| | ||||  || ||  | ||||  |
Sbjct  2874829  CTTTTCTACCTTCGGAACTTTTGTTTCTGCCTTTGCTTAAAACCCTGTATTTGGATCAAA  2874770

Query  386      ATAAATTAACTCTAACGGAAACGGATGTGGAAATTCTCGCTTCTCTTTCCTCCTTGGAGG  445
                | ||  | ||  | || ||||||||||| |||||||| ||||||||||||   ||||| |
Sbjct  2874769  ACAAGCTTACGTTGACTGAAACGGATGTTGAAATTCTAGCTTCTCTTTCCAGTTTGGAAG  2874710

Query  446      AATTGGATCTGAGTCTGAATTCGGGGATTAAGGCGCTCCCTTTTAATTACGAAAAACTTG  505
                |||||||||| | | |||| || || || || | ||| |||| ||| ||  | ||| |
Sbjct  2874709  AATTGGATCTTAATTTGAACTCTGGAATCAAAGTGCTTCCTTCTAACTATAATAAATTGA  2874650

Query  506      TAAATCTCATTAATCTAAAAAGATTGAATATTaaaaaaaCTTCATTAAAGGGAGAGGATG  565
                 |||||| | | || ||||||||||||||||||||||||||||||||||||| |||||||
Sbjct  2874649  AAAATCTGACTCATTTAAAAAGATTGAATATTAAAAAAACTTCATTAAAGGGGGAGGATG  2874590

Query  566      CAGATAAATTGCAGGCGATTCTTCCAAATACCAAAATCGACTACTGA  612
                | |||||||| || ||  |||| |||||||| | ||| ||||| |||
Sbjct  2874589  CTGATAAATTACAAGCAGTTCTCCCAAATACAAGAATAGACTATTGA  2874543
```

>Leptospira interrogans strain R16 chromosome 1
Sequence ID: CP047502.1 Length: 4213648
Range 1: 2865029 to 2865615

Score:469 bits(519), Expect:2e-130,
Identities:456/587(78%), Gaps:0/587(0%), Strand: Plus/Minus

```
Query  26       TCGACTGTAAAAAGAATGCGGAAGAGATTTTGGGAGAGGCAAAGGCAAAACCGGAATTGG  85
                |||| |||||||| || |||| ||||||||| | ||| |||||   |||| |||||| |
Sbjct  2865615  TCGATTGTAAAAAAAACGCGGTAGAGATTTTAGAAGAAGCAAAAAGAAAATCGGAATCGA  2865556

Query  86       TTCAAACTTTGGATTTTGGAATGCAGAAGTTGTCCACGGTTCCGGAAGAAGTTTGCGGTT  145
                ||||||  || ||||| |||||||| |||||  || || |||| |||| |||||||  ||
Sbjct  2865555  TTCAAATCTTAGATTTAGGAATGCAAAAGTTAACCTCGATTCCAGAAGGAGTTTGCTCTT  2865496

Query  146      TTCCGAATTTAACCAAGTTGGATCTTCGTTTAAACAGTTTGACTTTTCTTCCGGAATTTA  205
                |||||||||| ||  || ||||| | ||||| |||||||||| || |||||| |  |  |
Sbjct  2865495  TTCCGAATTTGACTCAGCTGGATTTACGTTTGAACAGTTTGAATTCTCTTCCTGGTTGGA  2865436
```

```
Query  206      TCGGAGAATGTAAACGCCTTGAACAACTTAATCTTTTCGGGAACGACCTTACAACATTTC  265
                | ||||  ||||||   || |||||| |||| |||||||| || || || |  ||  |||
Sbjct  2865435  TTGGAGCTTGTAAAAATCTGGAACAAATTAACCTTTTCGGAAATGATCTAAATACGGTTC  2865376

Query  266      CGTCTACGTTTTCTAAATTAAAAAATCTGAAAGTGTTACTGGCGGGAAATAACGATTTTA  325
                | ||| | |||||||||||||| ||| ||||||| |||||   ||| |||||||||||||
Sbjct  2865375  CATCTTCTTTTTCTAAATTAAAGAATTTGAAAGTATTACTCTTGGGTAATAACGATTTTA  2865316

Query  326      CGATTCTACCCTCCGAACTTTTGTTTCTTCCGTTGATCAAAATTCTATACGTAGATCGGA  385
                |  ||||||| || |||||||||||||| || ||| | ||||  || ||  | ||||  |
Sbjct  2865315  CTTTTCTACCTTCGGAACTTTTGTTTCTGCCTTTGCTTAAAACCCTGTATTTGGATCAAA  2865256

Query  386      ATAAATTAACTCTAACGGAAACGGATGTGGAAATTCTCGCTTCTCTTTCCTCCTTGGAGG  445
                | ||  | ||  | || ||||||||||| |||||||| ||||||||||||   ||||| |
Sbjct  2865255  ACAAGCTTACGTTGACTGAAACGGATGTTGAAATTCTAGCTTCTCTTTCCAGTTTGGAAG  2865196

Query  446      AATTGGATCTGAGTCTGAATTCGGGGATTAAGGCGCTCCCTTTTAATTACGAAAAACTTG  505
                |||||||||| | | |||| || || || || | ||| |||| ||| ||  | ||| |
Sbjct  2865195  AATTGGATCTTAATTTGAACTCTGGAATCAAAGTGCTTCCTTCTAACTATAATAAATTGA  2865136

Query  506      TAAATCTCATTAATCTAAAAAGATTGAATATTaaaaaaaCTTCATTAAAGGGAGAGGATG  565
                 |||||| | | || ||||||||||||||||||||||||||||||||||||| |||||||
Sbjct  2865135  AAAATCTGACTCATTTAAAAAGATTGAATATTAAAAAAACTTCATTAAAGGGGGAGGATG  2865076

Query  566      CAGATAAATTGCAGGCGATTCTTCCAAATACCAAAATCGACTACTGA  612
                | |||||||| || ||  |||| |||||||| | ||| ||||| |||
Sbjct  2865075  CTGATAAATTACAAGCAGTTCTCCCAAATACAAGAATAGACTATTGA  2865029
```

>Leptospira interrogans serovar Hardjo strain L53 chromosome 1, complete sequence
Sequence ID: CP043041.1 Length: 4355568
Range 1: 1437409 to 1437995

Score:469 bits(519), Expect:2e-130,
Identities:456/587(78%), Gaps:0/587(0%), Strand: Plus/Plus

```
Query  26       TCGACTGTAAAAAGAATGCGGAAGAGATTTTGGGAGAGGCAAAGGCAAAACCGGAATTGG  85
                |||| |||||||| || |||| ||||||||| | ||| |||||   |||| |||||| |
Sbjct  1437409  TCGATTGTAAAAAAAACGCGGTAGAGATTTTAGAAGAAGCAAAAAGAAAATCGGAATCGA  1437468

Query  86       TTCAAACTTTGGATTTTGGAATGCAGAAGTTGTCCACGGTTCCGGAAGAAGTTTGCGGTT  145
                ||||||  || ||||| |||||||| |||||  || || |||| |||| |||||||  ||
Sbjct  1437469  TTCAAATCTTAGATTTAGGAATGCAAAAGTTAACCTCGATTCCAGAAGGAGTTTGCTCTT  1437528

Query  146      TTCCGAATTTAACCAAGTTGGATCTTCGTTTAAACAGTTTGACTTTTCTTCCGGAATTTA  205
                |||||||||| ||  || ||||| | ||||| |||||||||| || |||||| |  |  |
Sbjct  1437529  TTCCGAATTTGACTCAGCTGGATTTACGTTTGAACAGTTTGAATTCTCTTCCTGGTTGGA  1437588

Query  206      TCGGAGAATGTAAACGCCTTGAACAACTTAATCTTTTCGGGAACGACCTTACAACATTTC  265
                | ||||  ||||||   || |||||| |||| |||||||| || || || |  |   |||
Sbjct  1437589  TTGGAGCTTGTAAAAATCTGGAACAAATTAACCTTTTCGGAAATGATCTAAATATGGTTC  1437648

Query  266      CGTCTACGTTTTCTAAATTAAAAAATCTGAAAGTGTTACTGGCGGGAAATAACGATTTTA  325
                | ||| | |||||||||||||| ||| ||||||| || ||   ||| |||||||||||||
Sbjct  1437649  CATCTTCTTTTTCTAAATTAAAGAATTTGAAAGTATTGCTCTTGGGTAATAACGATTTTA  1437708

Query  326      CGATTCTACCCTCCGAACTTTTGTTTCTTCCGTTGATCAAAATTCTATACGTAGATCGGA  385
                |  ||||||| || |||||||||||||| || ||||| ||||  || ||| | ||||  |
Sbjct  1437709  CTTTTCTACCTTCGGAACTTTTGTTTCTGCCTTTGATTAAAACCCTGTACTTGGATCAAA  1437768

Query  386      ATAAATTAACTCTAACGGAAACGGATGTGGAAATTCTCGCTTCTCTTTCCTCCTTGGAGG  445
```

```
                  | ||  | ||  | || ||||||||||| |||||||| ||||||||||||   ||||| |
Sbjct  1437769  ACAAGCTTACGTTGACCGAAACGGATGTTGAAATTCTAGCTTCTCTTTCCAGTTTGGAAG  1437828

Query  446      AATTGGATCTGAGTCTGAATTCGGGGATTAAGGCGCTCCCTTTTAATTACGAAAAACTTG  505
                |||||||||| | | |||| || || || || | ||| |||| ||| ||  | ||| |
Sbjct  1437829  AATTGGATCTTAATTTGAACTCTGGAATCAAAGTGCTTCCTTCTAACTATAATAAATTGA  1437888

Query  506      TAAATCTCATTAATCTAAAAAGATTGAATATTaaaaaaaCTTCATTAAAGGGAGAGGATG  565
                 |||||| | | || ||||||||||||||||||||||||||||||||||||| |||||||
Sbjct  1437889  AAAATCTGACTCATTTAAAAAGATTGAATATTAAAAAAACTTCATTAAAGGGGGAGGATG  1437948

Query  566      CAGATAAATTGCAGGCGATTCTTCCAAATACCAAAATCGACTACTGA  612
                | |||||||| || ||  |||| |||||||| | ||| ||||| |||
Sbjct  1437949  CTGATAAATTACAAGCAGTTCTCCCAAATACAAGAATAGACTATTGA  1437995
```

>Leptospira interrogans strain FMAS_KW1 chromosome I, complete sequence
Sequence ID: CP039258.1 Length: 4306144
Range 1: 1377410 to 1377996

Score:469 bits(519), Expect:2e-130,
Identities:456/587(78%), Gaps:0/587(0%), Strand: Plus/Plus

```
Query  26       TCGACTGTAAAAAGAATGCGGAAGAGATTTTGGGAGAGGCAAAGGCAAAACCGGAATTGG  85
                |||| |||||||| || |||| ||||||||| | ||| |||||   |||| |||||| |
Sbjct  1377410  TCGATTGTAAAAAAAACGCGGTAGAGATTTTAGAAGAAGCAAAAAGAAAATCGGAATCGA  1377469

Query  86       TTCAAACTTTGGATTTTGGAATGCAGAAGTTGTCCACGGTTCCGGAAGAAGTTTGCGGTT  145
                ||||||  || ||||| |||||||| |||||  || || |||| |||| |||||||  ||
Sbjct  1377470  TTCAAATCTTAGATTTAGGAATGCAAAAGTTAACCTCGATTCCAGAAGGAGTTTGCTCTT  1377529

Query  146      TTCCGAATTTAACCAAGTTGGATCTTCGTTTAAACAGTTTGACTTTTCTTCCGGAATTTA  205
                |||||||||| ||  || ||||| | ||||| |||||||||| || |||||| |  |  |
Sbjct  1377530  TTCCGAATTTGACTCAGCTGGATTTACGTTTGAACAGTTTGAATTCTCTTCCTGGTTGGA  1377589

Query  206      TCGGAGAATGTAAACGCCTTGAACAACTTAATCTTTTCGGGAACGACCTTACAACATTTC  265
                | || |  ||||||   || |||||| |||| |||||||| || || || |  ||  |||
Sbjct  1377590  TTGGGGCTTGTAAAAATCTGGAACAAATTAACCTTTTCGGAAATGATCTAAATACGGTTC  1377649

Query  266      CGTCTACGTTTTCTAAATTAAAAAATCTGAAAGTGTTACTGGCGGGAAATAACGATTTTA  325
                | ||| | ||||||||||||| ||| ||||||| || ||   ||| |||||||||||||
Sbjct  1377650  CATCTTCTTTTTCTAAATTAAAGAATTTGAAAGTATTGCTCTTGGGTAATAACGATTTTA  1377709

Query  326      CGATTCTACCCTCCGAACTTTTGTTTCTTCCGTTGATCAAAATTCTATACGTAGATCGGA  385
                |  ||||||| || |||||||||||||| || ||||| ||||  || ||| | ||||  |
Sbjct  1377710  CTTTTCTACCTTCGGAACTTTTGTTTCTGCCTTTGATTAAAACCCTGTACTTGGATCAAA  1377769

Query  386      ATAAATTAACTCTAACGGAAACGGATGTGGAAATTCTCGCTTCTCTTTCCTCCTTGGAGG  445
                | ||  | ||  | || ||||||||||| |||||||| ||||||||||||   ||||| |
Sbjct  1377770  ACAAGCTTACGTTGACTGAAACGGATGTTGAAATTCTAGCTTCTCTTTCCAGTTTGGAAG  1377829

Query  446      AATTGGATCTGAGTCTGAATTCGGGGATTAAGGCGCTCCCTTTTAATTACGAAAAACTTG  505
                |||||||||| | | |||| || || || || | ||| |||| ||||||  | ||| |
Sbjct  1377830  AATTGGATCTTAATTTGAACTCTGGAATCAAAGTGCTTCCTTCTAATTATAATAAATTGA  1377889

Query  506      TAAATCTCATTAATCTAAAAAGATTGAATATTaaaaaaaCTTCATTAAAGGGAGAGGATG  565
                 |||||| | | || ||||||||||||||||||||||||||||||||||||| ||||| |
Sbjct  1377890  AAAATCTGACTCATTTAAAAAGATTGAATATTAAAAAAACTTCATTAAAGGGGGAGGACG  1377949

Query  566      CAGATAAATTGCAGGCGATTCTTCCAAATACCAAAATCGACTACTGA  612
                | |||||||| || ||  |||| |||||||| | ||| ||||| |||
```

```
Sbjct  1377950  CTGATAAATTACAAGCAGTTCTCCCAAATACAAGAATAGACTATTGA  1377996




>Leptospira interrogans strain FMAS_AW1 chromosome I, complete sequence
Sequence ID: CP039283.1 Length: 4497709
Range 1: 2831793 to 2832379

Score:469 bits(519), Expect:2e-130,
Identities:456/587(78%),  Gaps:0/587(0%), Strand: Plus/Minus

Query  26       TCGACTGTAAAAAGAATGCGGAAGAGATTTTGGGAGAGGCAAAGGCAAAACCGGAATTGG  85
                |||| |||||||| || |||| ||||||||| | ||| |||||   |||| |||||| |
Sbjct  2832379  TCGATTGTAAAAAAAACGCGGTAGAGATTTTAGAAGAAGCAAAAAGAAAATCGGAATCGA  2832320

Query  86       TTCAAACTTTGGATTTTGGAATGCAGAAGTTGTCCACGGTTCCGGAAGAAGTTTGCGGTT  145
                ||||||  || ||||| |||||||| |||||  || || |||| |||| |||||||  ||
Sbjct  2832319  TTCAAATCTTAGATTTAGGAATGCAAAAGTTAACCTCGATTCCAGAAGGAGTTTGCTCTT  2832260

Query  146      TTCCGAATTTAACCAAGTTGGATCTTCGTTTAAACAGTTTGACTTTTCTTCCGGAATTTA  205
                |||||||||| ||  || ||||| | ||||| |||||||||| || |||||| |  |  |
Sbjct  2832259  TTCCGAATTTGACTCAGCTGGATTTACGTTTGAACAGTTTGAATTCTCTTCCTGGTTGGA  2832200

Query  206      TCGGAGAATGTAAACGCCTTGAACAACTTAATCTTTTCGGGAACGACCTTACAACATTTC  265
                | ||||  ||||||   || |||||| |||| |||||||| || || || |  ||  |||
Sbjct  2832199  TTGGAGCTTGTAAAAATCTGGAACAAATTAACCTTTTCGGAAATGATCTAAATACGGTTC  2832140

Query  266      CGTCTACGTTTTCTAAATTAAAAAATCTGAAAGTGTTACTGGCGGGAAATAACGATTTTA  325
                | ||| | |||||||||||||| ||| ||||||| |||||   ||| |||||||||||||
Sbjct  2832139  CATCTTCTTTTTCTAAATTAAAGAATTTGAAAGTATTACTCTTGGGTAATAACGATTTTA  2832080

Query  326      CGATTCTACCCTCCGAACTTTTGTTTCTTCCGTTGATCAAAATTCTATACGTAGATCGGA  385
                |  ||||||| || |||||||||||||| || ||| | ||||  || ||  | ||||  |
Sbjct  2832079  CTTTTCTACCTTCGGAACTTTTGTTTCTGCCTTTGCTTAAAACCCTGTATTTGGATCAAA  2832020

Query  386      ATAAATTAACTCTAACGGAAACGGATGTGGAAATTCTCGCTTCTCTTTCCTCCTTGGAGG  445
                | ||  | ||  | || ||||||||||| ||||||||| ||||||||||||   ||||| |
Sbjct  2832019  ACAAGCTTACGTTGACTGAAACGGATGTTGAAATTCTAGCTTCTCTTTCCAGTTTGGAAG  2831960

Query  446      AATTGGATCTGAGTCTGAATTCGGGGATTAAGGCGCTCCCTTTTAATTACGAAAAACTTG  505
                |||||||||| | | |||| || || || || | ||| |||| ||| ||  | ||| |
Sbjct  2831959  AATTGGATCTTAATTTGAACTCTGGAATCAAAGTGCTTCCTTCTAACTATAATAAATTGA  2831900

Query  506      TAAATCTCATTAATCTAAAAAGATTGAATATTaaaaaaaCTTCATTAAAGGGAGAGGATG  565
                 |||||| | | || ||||||||||||||||||||||||||||||||||||| |||||||
Sbjct  2831899  AAAATCTGACTCATTTAAAAAGATTGAATATTAAAAAAACTTCATTAAAGGGGGAGGATG  2831840

Query  566      CAGATAAATTGCAGGCGATTCTTCCAAATACCAAAATCGACTACTGA  612
                | |||||||| || ||  |||| |||||||| | ||| ||||| |||
Sbjct  2831839  CTGATAAATTACAAGCAGTTCTCCCAAATACAAGAATAGACTATTGA  2831793




>Leptospira interrogans serovar Copenhageni strain FDAARGOS_203 chromosome, complete
genome
Sequence ID: CP020414.2 Length: 4280582
Range 1: 918642 to 919228

Score:469 bits(519), Expect:2e-130,
Identities:456/587(78%),  Gaps:0/587(0%), Strand: Plus/Minus
```

```
Query  26      TCGACTGTAAAAAGAATGCGGAAGAGATTTTGGGAGAGGCAAAGGCAAAACCGGAATTGG  85
               |||| |||||||| || |||| ||||||||| | ||| |||||   |||| |||||| |
Sbjct  919228  TCGATTGTAAAAAAAACGCGGTAGAGATTTTAGAAGAAGCAAAAAGAAAATCGGAATCGA  919169

Query  86      TTCAAACTTTGGATTTTGGAATGCAGAAGTTGTCCACGGTTCCGGAAGAAGTTTGCGGTT  145
               ||||||  || ||||| |||||||| |||||  || || |||| |||| |||||||  ||
Sbjct  919168  TTCAAATCTTAGATTTAGGAATGCAAAAGTTAACCTCGATTCCAGAAGGAGTTTGCTCTT  919109

Query  146     TTCCGAATTTAACCAAGTTGGATCTTCGTTTAAACAGTTTGACTTTTCTTCCGGAATTTA  205
               |||||||||| ||  || ||||| | ||||| |||||||||| || |||||| |  |  |
Sbjct  919108  TTCCGAATTTGACTCAGCTGGATTTACGTTTGAACAGTTTGAATTCTCTTCCTGGTTGGA  919049

Query  206     TCGGAGAATGTAAACGCCTTGAACAACTTAATCTTTTCGGGAACGACCTTACAACATTTC  265
               | ||||  ||||||   || |||||| |||| |||||||| || || || |  ||  |||
Sbjct  919048  TTGGAGCTTGTAAAAATCTGGAACAAATTAACCTTTTCGGAAATGATCTAAATACGGTTC  918989

Query  266     CGTCTACGTTTTCTAAATTAAAAAATCTGAAAGTGTTACTGGCGGGAAATAACGATTTTA  325
               | ||| | |||||||||||||| ||| ||||||| |||||   ||| |||||||||||||
Sbjct  918988  CATCTTCTTTTTCTAAATTAAAGAATTTGAAAGTATTACTCTTGGGTAATAACGATTTTA  918929

Query  326     CGATTCTACCCTCCGAACTTTTGTTTCTTCCGTTGATCAAAATTCTATACGTAGATCGGA  385
               |  ||||||| || |||||||||||||| || ||| | ||||  || ||  | ||||  |
Sbjct  918928  CTTTTCTACCTTCGGAACTTTTGTTTCTGCCTTTGCTTAAAACCCTGTATTTGGATCAAA  918869

Query  386     ATAAATTAACTCTAACGGAAACGGATGTGGAAATTCTCGCTTCTCTTTCCTCCTTGGAGG  445
               | ||  | ||  | || ||||||||||| |||||||| ||||||||||||   ||||| |
Sbjct  918868  ACAAGCTTACGTTGACTGAAACGGATGTTGAAATTCTAGCTTCTCTTTCCAGTTTGGAAG  918809

Query  446     AATTGGATCTGAGTCTGAATTCGGGGATTAAGGCGCTCCCTTTTAATTACGAAAAACTTG  505
               |||||||||| | | |||| || || || || | ||| |||| ||| ||  | ||| |
Sbjct  918808  AATTGGATCTTAATTTGAACTCTGGAATCAAAGTGCTTCCTTCTAACTATAATAAATTGA  918749

Query  506     TAAATCTCATTAATCTAAAAAGATTGAATATTaaaaaaaCTTCATTAAAGGGAGAGGATG  565
                |||||| | | || ||||||||||||||||||||||||||||||||||||| |||||||
Sbjct  918748  AAAATCTGACTCATTTAAAAAGATTGAATATTAAAAAAACTTCATTAAAGGGGGAGGATG  918689

Query  566     CAGATAAATTGCAGGCGATTCTTCCAAATACCAAAATCGACTACTGA  612
               | |||||||| || ||  |||| |||||||| | ||| ||||| |||
Sbjct  918688  CTGATAAATTACAAGCAGTTCTCCCAAATACAAGAATAGACTATTGA  918642
```

>Leptospira interrogans strain RCA chromosome I
Sequence ID: CP022538.1 Length: 4241665
Range 1: 3328085 to 3328671

Score:469 bits(519), Expect:2e-130,
Identities:456/587(78%), Gaps:0/587(0%), Strand: Plus/Minus

```
Query  26       TCGACTGTAAAAAGAATGCGGAAGAGATTTTGGGAGAGGCAAAGGCAAAACCGGAATTGG  85
                |||| |||||||| || |||| ||||||||| | ||| |||||   |||| |||||| |
Sbjct  3328671  TCGATTGTAAAAAAAACGCGGTAGAGATTTTAGAAGAAGCAAAAAGAAAATCGGAATCGA  3328612

Query  86       TTCAAACTTTGGATTTTGGAATGCAGAAGTTGTCCACGGTTCCGGAAGAAGTTTGCGGTT  145
                ||||||  || ||||| |||||||| |||||  || || |||| |||| |||||||  ||
Sbjct  3328611  TTCAAATCTTAGATTTAGGAATGCAAAAGTTAACCTCGATTCCAGAAGGAGTTTGCTCTT  3328552

Query  146      TTCCGAATTTAACCAAGTTGGATCTTCGTTTAAACAGTTTGACTTTTCTTCCGGAATTTA  205
                |||||||||| ||  || ||||| | ||||| |||||||||| || |||||| |  |  |
Sbjct  3328551  TTCCGAATTTGACTCAGCTGGATTTACGTTTGAACAGTTTGAATTCTCTTCCTGGTTGGA  3328492

Query  206      TCGGAGAATGTAAACGCCTTGAACAACTTAATCTTTTCGGGAACGACCTTACAACATTTC  265
```

```
                 | ||||  ||||||   || |||||| |||| |||||||| || || || |  ||  |||
Sbjct  3328491   TTGGAGCTTGTAAAAATCTGGAACAAATTAACCTTTTCGGAAATGATCTAAATACGGTTC  3328432

Query  266       CGTCTACGTTTTCTAAATTAAAAAATCTGAAAGTGTTACTGGCGGGAAATAACGATTTTA  325
                 | ||| | |||||||||||||| ||| ||||||| |||||   ||| |||||||||||||
Sbjct  3328431   CATCTTCTTTTTCTAAATTAAAGAATTTGAAAGTATTACTCTTGGGTAATAACGATTTTA  3328372

Query  326       CGATTCTACCCTCCGAACTTTTGTTTCTTCCGTTGATCAAAATTCTATACGTAGATCGGA  385
                 |  ||||||| || |||||||||||||| || ||| | ||||  || ||  | ||||  |
Sbjct  3328371   CTTTTCTACCTTCGGAACTTTTGTTTCTGCCTTTGCTTAAAACCCTGTATTTGGATCAAA  3328312

Query  386       ATAAATTAACTCTAACGGAAACGGATGTGGAAATTCTCGCTTCTCTTTCCTCCTTGGAGG  445
                 | ||  | ||  | || ||||||||||| |||||||| ||||||||||||   ||||| |
Sbjct  3328311   ACAAGCTTACGTTGACTGAAACGGATGTTGAAATTCTAGCTTCTCTTTCCAGTTTGGAAG  3328252

Query  446       AATTGGATCTGAGTCTGAATTCGGGGATTAAGGCGCTCCCTTTTAATTACGAAAAACTTG  505
                 |||||||||| | | |||| || || || || | ||| |||| ||| ||  | ||| |
Sbjct  3328251   AATTGGATCTTAATTTGAACTCTGGAATCAAAGTGCTTCCTTCTAACTATAATAAATTGA  3328192

Query  506       TAAATCTCATTAATCTAAAAAGATTGAATATTaaaaaaaCTTCATTAAAGGGAGAGGATG  565
                  |||||| | | || ||||||||||||||||||||||||||||||||||||| |||||||
Sbjct  3328191   AAAATCTGACTCATTTAAAAAGATTGAATATTAAAAAAACTTCATTAAAGGGGGAGGATG  3328132

Query  566       CAGATAAATTGCAGGCGATTCTTCCAAATACCAAAATCGACTACTGA  612
                 | |||||||| || ||  |||| |||||||| | ||| ||||| |||
Sbjct  3328131   CTGATAAATTACAAGCAGTTCTCCCAAATACAAGAATAGACTATTGA  3328085
```

```
>Leptospira interrogans serovar Hardjo-prajitno strain Hardjoprajitno chromosome 1
sequence
Sequence ID: CP013147.1 Length: 4339653
Range 1: 1431712 to 1432298

Score:469 bits(519), Expect:2e-130,
Identities:456/587(78%),  Gaps:0/587(0%), Strand: Plus/Plus

Query  26        TCGACTGTAAAAAGAATGCGGAAGAGATTTTGGGAGAGGCAAAGGCAAAACCGGAATTGG  85
                 |||| |||||||| || |||| ||||||||| | ||| |||||   |||| |||||| |
Sbjct  1431712   TCGATTGTAAAAAAAACGCGGTAGAGATTTTAGAAGAAGCAAAAAGAAAATCGGAATCGA  1431771

Query  86        TTCAAACTTTGGATTTTGGAATGCAGAAGTTGTCCACGGTTCCGGAAGAAGTTTGCGGTT  145
                 ||||||  || ||||| |||||||| |||||  || || |||| |||| |||||||  ||
Sbjct  1431772   TTCAAATCTTAGATTTAGGAATGCAAAAGTTAACCTCGATTCCAGAAGGAGTTTGCTCTT  1431831

Query  146       TTCCGAATTTAACCAAGTTGGATCTTCGTTTAAACAGTTTGACTTTTCTTCCGGAATTTA  205
                 |||||||||| ||  || ||||| | ||||| |||||||||| || |||||| |  |  |
Sbjct  1431832   TTCCGAATTTGACTCAGCTGGATTTACGTTTGAACAGTTTGAATTCTCTTCCTGGTTGGA  1431891

Query  206       TCGGAGAATGTAAACGCCTTGAACAACTTAATCTTTTCGGGAACGACCTTACAACATTTC  265
                 | ||||  ||||||   || |||||| |||| |||||||| || || || |  |   |||
Sbjct  1431892   TTGGAGCTTGTAAAAATCTGGAACAAATTAACCTTTTCGGAAATGATCTAAATATGGTTC  1431951

Query  266       CGTCTACGTTTTCTAAATTAAAAAATCTGAAAGTGTTACTGGCGGGAAATAACGATTTTA  325
                 | ||| | |||||||||||||| ||| ||||||| || ||   ||| |||||||||||||
Sbjct  1431952   CATCTTCTTTTTCTAAATTAAAGAATTTGAAAGTATTGCTCTTGGGTAATAACGATTTTA  1432011

Query  326       CGATTCTACCCTCCGAACTTTTGTTTCTTCCGTTGATCAAAATTCTATACGTAGATCGGA  385
                 |  ||||||| || |||||||||||||| || ||||| ||||  || ||| | ||||  |
Sbjct  1432012   CTTTTCTACCTTCGGAACTTTTGTTTCTGCCTTTGATTAAAACCCTGTACTTGGATCAAA  1432071

Query  386       ATAAATTAACTCTAACGGAAACGGATGTGGAAATTCTCGCTTCTCTTTCCTCCTTGGAGG  445
```

```
                 | ||  | ||  | || ||||||||||| |||||||| ||||||||||||   ||||| |
Sbjct  1432072  ACAAGCTTACGTTGACCGAAACGGATGTTGAAATTCTAGCTTCTCTTTCCAGTTTGGAAG  1432131

Query  446      AATTGGATCTGAGTCTGAATTCGGGGATTAAGGCGCTCCCTTTTAATTACGAAAAACTTG  505
                |||||||||| | | |||| || || || || | ||| |||| ||| ||  | ||| |
Sbjct  1432132  AATTGGATCTTAATTTGAACTCTGGAATCAAAGTGCTTCCTTCTAACTATAATAAATTGA  1432191

Query  506      TAAATCTCATTAATCTAAAAAGATTGAATATTaaaaaaaCTTCATTAAAGGGAGAGGATG  565
                 |||||| | | || ||||||||||||||||||||||||||||||||||||| |||||||
Sbjct  1432192  AAAATCTGACTCATTTAAAAAGATTGAATATTAAAAAAACTTCATTAAAGGGGGAGGATG  1432251

Query  566      CAGATAAATTGCAGGCGATTCTTCCAAATACCAAAATCGACTACTGA  612
                | |||||||| || ||  |||| |||||||| | ||| ||||| |||
Sbjct  1432252  CTGATAAATTACAAGCAGTTCTCCCAAATACAAGAATAGACTATTGA  1432298
```

>Leptospira interrogans serovar Hardjo str. Norma chromosome I, complete sequence
Sequence ID: CP012603.1 Length: 4406718
Range 1: 1409557 to 1410143

Score:469 bits(519), Expect:2e-130,
Identities:456/587(78%), Gaps:0/587(0%), Strand: Plus/Plus

```
Query  26       TCGACTGTAAAAAGAATGCGGAAGAGATTTTGGGAGAGGCAAAGGCAAAACCGGAATTGG  85
                |||| |||||||| || |||| ||||||||| | ||| |||||   |||| |||||| |
Sbjct  1409557  TCGATTGTAAAAAAAACGCGGTAGAGATTTTAGAAGAAGCAAAAAGAAAATCGGAATCGA  1409616

Query  86       TTCAAACTTTGGATTTTGGAATGCAGAAGTTGTCCACGGTTCCGGAAGAAGTTTGCGGTT  145
                |||||  || ||||| |||||||| |||||  || || |||| |||| |||||||  ||
Sbjct  1409617  TTCAAATCTTAGATTTAGGAATGCAAAAGTTAACCTCGATTCCAGAAGGAGTTTGCTCTT  1409676

Query  146      TTCCGAATTTAACCAAGTTGGATCTTCGTTTAAACAGTTTGACTTTTCTTCCGGAATTTA  205
                |||||||||| ||  || ||||| | ||||| |||||||||| || |||||| |  |  |
Sbjct  1409677  TTCCGAATTTGACTCAGCTGGATTTACGTTTGAACAGTTTGAATTCTCTTCCTGGTTGGA  1409736

Query  206      TCGGAGAATGTAAACGCCTTGAACAACTTAATCTTTTCGGGAACGACCTTACAACATTTC  265
                | ||||  ||||||   || |||||| |||| |||||||| || || || |  |   |||
Sbjct  1409737  TTGGAGCTTGTAAAAATCTGGAACAAATTAACCTTTTCGGAAATGATCTAAATATGGTTC  1409796

Query  266      CGTCTACGTTTTCTAAATTAAAAAATCTGAAAGTGTTACTGGCGGGAAATAACGATTTTA  325
                | ||| | ||||||||||||| ||| ||||||| || ||   ||| |||||||||||||
Sbjct  1409797  CATCTTCTTTTTCTAAATTAAAGAATTTGAAAGTATTGCTCTTGGGTAATAACGATTTTA  1409856

Query  326      CGATTCTACCCTCCGAACTTTTGTTTCTTCCGTTGATCAAAATTCTATACGTAGATCGGA  385
                |  ||||||| || ||||||||||||| || ||||| ||||  || ||| | ||||  |
Sbjct  1409857  CTTTTCTACCTTCGGAACTTTTGTTTCTGCCTTTGATTAAAACCCTGTACTTGGATCAAA  1409916

Query  386      ATAAATTAACTCTAACGGAAACGGATGTGGAAATTCTCGCTTCTCTTTCCTCCTTGGAGG  445
                | ||  | ||  | || ||||||||||| |||||||| ||||||||||||   ||||| |
Sbjct  1409917  ACAAGCTTACGTTGACCGAAACGGATGTTGAAATTCTAGCTTCTCTTTCCAGTTTGGAAG  1409976

Query  446      AATTGGATCTGAGTCTGAATTCGGGGATTAAGGCGCTCCCTTTTAATTACGAAAAACTTG  505
                |||||||||| | | |||| || || || || | ||| |||| ||| ||  | ||| |
Sbjct  1409977  AATTGGATCTTAATTTGAACTCTGGAATCAAAGTGCTTCCTTCTAACTATAATAAATTGA  1410036

Query  506      TAAATCTCATTAATCTAAAAAGATTGAATATTaaaaaaaCTTCATTAAAGGGAGAGGATG  565
                 |||||| | | || ||||||||||||||||||||||||||||||||||||| |||||||
Sbjct  1410037  AAAATCTGACTCATTTAAAAAGATTGAATATTAAAAAAACTTCATTAAAGGGGGAGGATG  1410096

Query  566      CAGATAAATTGCAGGCGATTCTTCCAAATACCAAAATCGACTACTGA  612
                | |||||||| || ||  |||| |||||||| | ||| ||||| |||
```

```
Sbjct  1410097  CTGATAAATTACAAGCAGTTCTCCCAAATACAAGAATAGACTATTGA  1410143
```

>Leptospira interrogans serovar Bratislava strain PigK151 chromosome I, complete sequence
Sequence ID: CP011410.1 Length: 4370471
Range 1: 1464085 to 1464671

Score:469 bits(519), Expect:2e-130,
Identities:456/587(78%), Gaps:0/587(0%), Strand: Plus/Plus

```
Query  26       TCGACTGTAAAAAGAATGCGGAAGAGATTTTGGGAGAGGCAAAGGCAAAACCGGAATTGG  85
                |||| |||||||| || |||| ||||||||| | ||| |||||   |||| |||||| |
Sbjct  1464085  TCGATTGTAAAAAAAACGCGGTAGAGATTTTAGAAGAAGCAAAAAGAAAATCGGAATCGA  1464144

Query  86       TTCAAACTTTGGATTTTGGAATGCAGAAGTTGTCCACGGTTCCGGAAGAAGTTTGCGGTT  145
                ||||||  || ||||| |||||||| |||||  || || |||| |||| |||||||  ||
Sbjct  1464145  TTCAAATCTTAGATTTAGGAATGCAAAAGTTAACCTCGATTCCAGAAGGAGTTTGCTCTT  1464204

Query  146      TTCCGAATTTAACCAAGTTGGATCTTCGTTTAAACAGTTTGACTTTTCTTCCGGAATTTA  205
                |||||||||| ||  || ||||| | ||||| |||||||||| || |||||| |  |  |
Sbjct  1464205  TTCCGAATTTGACTCAGCTGGATTTACGTTTGAACAGTTTGAATTCTCTTCCTGGTTGGA  1464264

Query  206      TCGGAGAATGTAAACGCCTTGAACAACTTAATCTTTTCGGGAACGACCTTACAACATTTC  265
                | ||||  ||||||   || |||||| |||| |||||||| || || || |  ||  |||
Sbjct  1464265  TTGGAGCTTGTAAAAATCTGGAACAAATTAACCTTTTCGGAAATGATCTAAATACGGTTC  1464324

Query  266      CGTCTACGTTTTCTAAATTAAAAAATCTGAAAGTGTTACTGGCGGGAAATAACGATTTTA  325
                | ||| | |||||||||||||| ||| ||||||| || ||   ||| |||||||||||||
Sbjct  1464325  CATCTTCTTTTTCTAAATTAAAGAATTTGAAAGTATTGCTCTTGGGTAATAACGATTTTA  1464384

Query  326      CGATTCTACCCTCCGAACTTTTGTTTCTTCCGTTGATCAAAATTCTATACGTAGATCGGA  385
                |  ||||||| || |||||||||||||| || ||| | ||||  || ||| | ||||  |
Sbjct  1464385  CTTTTCTACCTTCGGAACTTTTGTTTCTGCCTTTGCTTAAAACCCTGTACTTGGATCAAA  1464444

Query  386      ATAAATTAACTCTAACGGAAACGGATGTGGAAATTCTCGCTTCTCTTTCCTCCTTGGAGG  445
                | ||  | ||  | || ||||||||||| |||||||| ||||||||||||   ||||| |
Sbjct  1464445  ACAAGCTTACGTTGACTGAAACGGATGTTGAAATTCTAGCTTCTCTTTCCAGTTTGGAAG  1464504

Query  446      AATTGGATCTGAGTCTGAATTCGGGGATTAAGGCGCTCCCTTTTAATTACGAAAAACTTG  505
                |||||||||| | | |||| || || || || | ||| |||| ||| ||  | ||| |
Sbjct  1464505  AATTGGATCTTAATTTGAACTCTGGAATCAAAGTGCTTCCTTCTAACTATAATAAATTGA  1464564

Query  506      TAAATCTCATTAATCTAAAAAGATTGAATATTaaaaaaaCTTCATTAAAGGGAGAGGATG  565
                 |||||| | | || ||||||||||||||||||||||||||||||||||||| |||||||
Sbjct  1464565  AAAATCTGACTCATTTAAAAAGATTGAATATTAAAAAAACTTCATTAAAGGGGGAGGATG  1464624

Query  566      CAGATAAATTGCAGGCGATTCTTCCAAATACCAAAATCGACTACTGA  612
                | |||||||| || ||  |||| |||||||| | ||| ||||| |||
Sbjct  1464625  CTGATAAATTACAAGCAGTTCTCCCAAATACAAGAATAGACTATTGA  1464671
```

>Leptospira interrogans serovar Linhai str. 56609 chromosome 1, complete sequence
Sequence ID: CP006723.1 Length: 4331770
Range 1: 1409365 to 1409951

Score:469 bits(519), Expect:2e-130,
Identities:456/587(78%), Gaps:0/587(0%), Strand: Plus/Plus

```
Query  26       TCGACTGTAAAAAGAATGCGGAAGAGATTTTGGGAGAGGCAAAGGCAAAACCGGAATTGG  85
```

```
                  |||| |||||||| || |||| ||||||||| | ||| |||||   |||| |||||| |
Sbjct  1409365    TCGATTGTAAAAAAAACGCGGTAGAGATTTTAGAAGAAGCAAAAAGAAAATCGGAATCGA  1409424

Query  86         TTCAAACTTTGGATTTTGGAATGCAGAAGTTGTCCACGGTTCCGGAAGAAGTTTGCGGTT  145
                  ||||||  || ||||| |||||||| |||||  || || |||| |||| |||||||  ||
Sbjct  1409425    TTCAAATCTTAGATTTAGGAATGCAAAAGTTAACCTCGATTCCAGAAGGAGTTTGCTCTT  1409484

Query  146        TTCCGAATTTAACCAAGTTGGATCTTCGTTTAAACAGTTTGACTTTTCTTCCGGAATTTA  205
                  |||||||||| ||  || ||||| | ||||| |||||||||| || |||||| |  |  |
Sbjct  1409485    TTCCGAATTTGACTCAGCTGGATTTACGTTTGAACAGTTTGAATTCTCTTCCTGGTTGGA  1409544

Query  206        TCGGAGAATGTAAACGCCTTGAACAACTTAATCTTTTCGGGAACGACCTTACAACATTTC  265
                  | ||||  ||||||   || |||||| |||| |||||||| || || || |  ||  |||
Sbjct  1409545    TTGGAGCTTGTAAAAATCTGGAACAAATTAACCTTTTCGGAAATGATCTGAATACGGTTC  1409604

Query  266        CGTCTACGTTTTCTAAATTAAAAAATCTGAAAGTGTTACTGGCGGGAAATAACGATTTTA  325
                  | ||| | ||||||||||||||| ||| |||||||| || ||   ||| |||||||||||||
Sbjct  1409605    CATCTTCTTTTTCTAAATTAAAGAATTTGAAAGTATTGCTCTTGGGTAATAACGATTTTA  1409664

Query  326        CGATTCTACCCTCCGAACTTTTGTTTCTTCCGTTGATCAAAATTCTATACGTAGATCGGA  385
                  |  ||||||| || |||||||||||||| || ||| | ||||  || ||| | ||||  |
Sbjct  1409665    CTTTTCTACCTTCGGAACTTTTGTTTCTGCCTTTGCTTAAAACCCTGTACTTGGATCAAA  1409724

Query  386        ATAAATTAACTCTAACGGAAACGGATGTGGAAATTCTCGCTTCTCTTTCCTCCTTGGAGG  445
                  | ||  | ||  | || ||||||||||| |||||||| ||||||||||||   ||||| |
Sbjct  1409725    ACAAGCTTACGTTGACTGAAACGGATGTTGAAATTCTAGCTTCTCTTTCCAGTTTGGAAG  1409784

Query  446        AATTGGATCTGAGTCTGAATTCGGGGATTAAGGCGCTCCCTTTTAATTACGAAAAACTTG  505
                  |||||||||| | | |||| || || || || | ||| |||| ||| ||  | ||| |
Sbjct  1409785    AATTGGATCTTAATTTGAACTCTGGAATCAAAGTGCTTCCTTCTAACTATAATAAATTGA  1409844

Query  506        TAAATCTCATTAATCTAAAAAGATTGAATATTaaaaaaaCTTCATTAAAGGGAGAGGATG  565
                   |||||| | | || ||||||||||||||||||||||||||||||||||||| |||||||
Sbjct  1409845    AAAATCTGACTCATTTAAAAAGATTGAATATTAAAAAAACTTCATTAAAGGGGAGGATG  1409904

Query  566        CAGATAAATTGCAGGCGATTCTTCCAAATACCAAAATCGACTACTGA  612
                  | |||||||| || ||  |||| |||||||| | ||| ||||| |||
Sbjct  1409905    CTGATAAATTACAAGCAGTTCTCCCAAATACAAGAATAGACTATTGA  1409951
```

>Leptospira interrogans strain KR40 chromosome 1, complete sequence
Sequence ID: CP096129.2 Length: 4315670
Range 1: 1412082 to 1412668

Score:469 bits(519), Expect:2e-130,
Identities:456/587(78%), Gaps:0/587(0%), Strand: Plus/Plus

```
Query  26         TCGACTGTAAAAAGAATGCGGAAGAGATTTTGGGAGAGGCAAAGGCAAAACCGGAATTGG  85
                  |||| |||||||| || |||| ||||||||| | ||| |||||   |||| |||||| |
Sbjct  1412082    TCGATTGTAAAAAAAACGCGGTAGAGATTTTAGAAGAAGCAAAAAGAAAATCGGAATCGA  1412141

Query  86         TTCAAACTTTGGATTTTGGAATGCAGAAGTTGTCCACGGTTCCGGAAGAAGTTTGCGGTT  145
                  ||||||  || ||||| |||||||| |||||  || || |||| |||| |||||||  ||
Sbjct  1412142    TTCAAATCTTAGATTTAGGAATGCAAAAGTTAACCTCGATTCCAGAAGGAGTTTGCTCTT  1412201

Query  146        TTCCGAATTTAACCAAGTTGGATCTTCGTTTAAACAGTTTGACTTTTCTTCCGGAATTTA  205
                  |||||||||| ||  || ||||| | ||||| |||||||||| || |||||| |  |  |
Sbjct  1412202    TTCCGAATTTGACTCAGCTGGATTTACGTTTGAACAGTTTGAATTCTCTTCCTGGTTGGA  1412261

Query  206        TCGGAGAATGTAAACGCCTTGAACAACTTAATCTTTTCGGGAACGACCTTACAACATTTC  265
                  | ||||  ||||||   || |||||| |||| |||||||| || || || |  |   |||
```

```
Sbjct  1412262  TTGGAGCTTGTAAAAATCTGGAACAAATTAACCTTTTCGGAAATGATCTAAATATGGTTC  1412321

Query  266      CGTCTACGTTTTCTAAATTAAAAAATCTGAAAGTGTTACTGGCGGGAAATAACGATTTTA  325
                | ||| | |||||||||||||| ||| ||||||| || ||   ||| |||||||||||||
Sbjct  1412322  CATCTTCTTTTTCTAAATTAAAGAATTTGAAAGTATTGCTCTTGGGTAATAACGATTTTA  1412381

Query  326      CGATTCTACCCTCCGAACTTTTGTTTCTTCCGTTGATCAAAATTCTATACGTAGATCGGA  385
                |  ||||||| || |||||||||||||| || ||||| ||||  || ||| | ||||  |
Sbjct  1412382  CTTTTCTACCTTCGGAACTTTTGTTTCTGCCTTTGATTAAAACCCTGTACTTGGATCAAA  1412441

Query  386      ATAAATTAACTCTAACGGAAACGGATGTGGAAATTCTCGCTTCTCTTTCCTCCTTGGAGG  445
                | ||  | ||  | || |||||||||| |||||||| ||||||||||||   ||||| |
Sbjct  1412442  ACAAGCTTACGTTGACCGAAACGGATGTTGAAATTCTAGCTTCTCTTTCCAGTTTGGAAG  1412501

Query  446      AATTGGATCTGAGTCTGAATTCGGGGATTAAGGCGCTCCCTTTTAATTACGAAAAACTTG  505
                |||||||||| | | |||| || || || || | ||| |||| ||| ||  | ||| |
Sbjct  1412502  AATTGGATCTTAATTTGAACTCTGGAATCAAAGTGCTTCCTTCTAACTATAATAAATTGA  1412561

Query  506      TAAATCTCATTAATCTAAAAAGATTGAATATTaaaaaaaCTTCATTAAAGGGAGAGGATG  565
                 |||||| | | || ||||||||||||||||||||||||||||||||||||| |||||||
Sbjct  1412562  AAAATCTGACTCATTTAAAAAGATTGAATATTAAAAAAACTTCATTAAAGGGGGAGGATG  1412621

Query  566      CAGATAAATTGCAGGCGATTCTTCCAAATACCAAAATCGACTACTGA  612
                | |||||||| || ||  |||| |||||||| | ||| ||||| |||
Sbjct  1412622  CTGATAAATTACAAGCAGTTCTCCCAAATACAAGAATAGACTATTGA  1412668



>Leptospira interrogans strain N 116 chromosome 1, complete sequence
Sequence ID: CP097315.1 Length: 4312952
Range 1: 1411824 to 1412410

Score:469 bits(519), Expect:2e-130,
Identities:456/587(78%),  Gaps:0/587(0%), Strand: Plus/Plus

Query  26       TCGACTGTAAAAAGAATGCGGAAGAGATTTTGGGAGAGGCAAAGGCAAAACCGGAATTGG  85
                |||| |||||||| || |||| ||||||||| | ||| |||||   |||| |||||| |
Sbjct  1411824  TCGATTGTAAAAAAAACGCGGTAGAGATTTTAGAAGAAGCAAAAAGAAAATCGGAATCGA  1411883

Query  86       TTCAAACTTTGGATTTTGGAATGCAGAAGTTGTCCACGGTTCCGGAAGAAGTTTGCGGTT  145
                ||||||  || ||||| |||||||| |||||  || || |||| |||| |||||||  ||
Sbjct  1411884  TTCAAATCTTAGATTTAGGAATGCAAAAGTTAACCTCGATTCCAGAAGGAGTTTGCTCTT  1411943

Query  146      TTCCGAATTTAACCAAGTTGGATCTTCGTTTAAACAGTTTGACTTTTCTTCCGGAATTTA  205
                |||||||||| ||  || ||||| | ||||| |||||||||| || |||||| |  |  |
Sbjct  1411944  TTCCGAATTTGACTCAGCTGGATTTACGTTTGAACAGTTTGAATTCTCTTCCTGGTTGGA  1412003

Query  206      TCGGAGAATGTAAACGCCTTGAACAACTTAATCTTTTCGGGAACGACCTTACAACATTTC  265
                | ||||  ||||||   || |||||| |||| |||||||| || || || |  |   |||
Sbjct  1412004  TTGGAGCTTGTAAAAATCTGGAACAAATTAACCTTTTCGGAAATGATCTAAATATGGTTC  1412063

Query  266      CGTCTACGTTTTCTAAATTAAAAAATCTGAAAGTGTTACTGGCGGGAAATAACGATTTTA  325
                | ||| | |||||||||||||| ||| ||||||| || ||   ||| |||||||||||||
Sbjct  1412064  CATCTTCTTTTTCTAAATTAAAGAATTTGAAAGTATTGCTCTTGGGTAATAACGATTTTA  1412123

Query  326      CGATTCTACCCTCCGAACTTTTGTTTCTTCCGTTGATCAAAATTCTATACGTAGATCGGA  385
                |  ||||||| || |||||||||||||| || ||||| ||||  || ||| | ||||  |
Sbjct  1412124  CTTTTCTACCTTCGGAACTTTTGTTTCTGCCTTTGATTAAAACCCTGTACTTGGATCAAA  1412183

Query  386      ATAAATTAACTCTAACGGAAACGGATGTGGAAATTCTCGCTTCTCTTTCCTCCTTGGAGG  445
                | ||  | ||  | || |||||||||| |||||||| ||||||||||||   ||||| |
Sbjct  1412184  ACAAGCTTACGTTGACCGAAACGGATGTTGAAATTCTAGCTTCTCTTTCCAGTTTGGAAG  1412243
```

```
Query  446      AATTGGATCTGAGTCTGAATTCGGGGATTAAGGCGCTCCCTTTTAATTACGAAAAACTTG  505
                |||||||||| | | |||| || || || || | ||| |||| ||| ||  | ||| |
Sbjct  1412244  AATTGGATCTTAATTTGAACTCTGGAATCAAAGTGCTTCCTTCTAACTATAATAAATTGA  1412303

Query  506      TAAATCTCATTAATCTAAAAAGATTGAATATTaaaaaaaCTTCATTAAAGGGAGAGGATG  565
                 |||||| | | || ||||||||||||||||||||||||||||||||||||| |||||||
Sbjct  1412304  AAAATCTGACTCATTTAAAAAGATTGAATATTAAAAAAACTTCATTAAAGGGGGAGGATG  1412363

Query  566      CAGATAAATTGCAGGCGATTCTTCCAAATACCAAAATCGACTACTGA  612
                | |||||||| || ||  |||| |||||||| | ||| ||||| |||
Sbjct  1412364  CTGATAAATTACAAGCAGTTCTCCCAAATACAAGAATAGACTATTGA  1412410
```

>Leptospira interrogans strain NEG7 chromosome I
Sequence ID: CP093938.1 Length: 4297584
Range 1: 2911278 to 2911864

Score:469 bits(519), Expect:2e-130,
Identities:456/587(78%), Gaps:0/587(0%), Strand: Plus/Minus

```
Query  26       TCGACTGTAAAAAGAATGCGGAAGAGATTTTGGGAGAGGCAAAGGCAAAACCGGAATTGG  85
                |||| |||||||| || |||| ||||||||| | ||| |||||   |||| |||||| |
Sbjct  2911864  TCGATTGTAAAAAAAACGCGGTAGAGATTTTAGAAGAAGCAAAAAGAAAATCGGAATCGA  2911805

Query  86       TTCAAACTTTGGATTTTGGAATGCAGAAGTTGTCCACGGTTCCGGAAGAAGTTTGCGGTT  145
                ||||||  || ||||| |||||||| |||||  || || |||| |||| |||||||  ||
Sbjct  2911804  TTCAAATCTTAGATTTAGGAATGCAAAAGTTAACCTCGATTCCAGAAGGAGTTTGCTCTT  2911745

Query  146      TTCCGAATTTAACCAAGTTGGATCTTCGTTTAAACAGTTTGACTTTTCTTCCGGAATTTA  205
                |||||||||| ||  || ||||| | ||||| |||||||||| || |||||| |  |  |
Sbjct  2911744  TTCCGAATTTGACTCAGCTGGATTTACGTTTGAACAGTTTGAATTCTCTTCCTGGTTGGA  2911685

Query  206      TCGGAGAATGTAAACGCCTTGAACAACTTAATCTTTTCGGGAACGACCTTACAACATTTC  265
                | ||||  ||||||   || |||||| |||| |||||||| || || || |  ||  |||
Sbjct  2911684  TTGGAGCTTGTAAAAATCTGGAACAAATTAACCTTTTCGGAAATGATCTAAATACGGTTC  2911625

Query  266      CGTCTACGTTTTCTAAATTAAAAAATCTGAAAGTGTTACTGGCGGGAAATAACGATTTTA  325
                | ||| | |||||||||||||| ||| ||||||| |||||   ||| |||||||||||||
Sbjct  2911624  CATCTTCTTTTTCTAAATTAAAGAATTTGAAAGTATTACTCTTGGGTAATAACGATTTTA  2911565

Query  326      CGATTCTACCCTCCGAACTTTTGTTTCTTCCGTTGATCAAAATTCTATACGTAGATCGGA  385
                |  ||||||| || |||||||||||||| || ||| | ||||  || ||  | ||||  |
Sbjct  2911564  CTTTTCTACCTTCGGAACTTTTGTTTCTGCCTTTGCTTAAAACCCTGTATTTGGATCAAA  2911505

Query  386      ATAAATTAACTCTAACGGAAACGGATGTGGAAATTCTCGCTTCTCTTTCCTCCTTGGAGG  445
                | ||  | ||  | || ||||||||||| |||||||| ||||||||||||   ||||| |
Sbjct  2911504  ACAAGCTTACGTTGACTGAAACGGATGTTGAAATTCTAGCTTCTCTTTCCAGTTTGGAAG  2911445

Query  446      AATTGGATCTGAGTCTGAATTCGGGGATTAAGGCGCTCCCTTTTAATTACGAAAAACTTG  505
                |||||||||| | | |||| || || || || | ||| |||| ||| ||  | ||| |
Sbjct  2911444  AATTGGATCTTAATTTGAACTCTGGAATCAAAGTGCTTCCTTCTAACTATAATAAATTGA  2911385

Query  506      TAAATCTCATTAATCTAAAAAGATTGAATATTaaaaaaaCTTCATTAAAGGGAGAGGATG  565
                 |||||| | | || ||||||||||||||||||||||||||||||||||||| |||||||
Sbjct  2911384  AAAATCTGACTCATTTAAAAAGATTGAATATTAAAAAAACTTCATTAAAGGGGGAGGATG  2911325

Query  566      CAGATAAATTGCAGGCGATTCTTCCAAATACCAAAATCGACTACTGA  612
                | |||||||| || ||  |||| |||||||| | ||| ||||| |||
Sbjct  2911324  CTGATAAATTACAAGCAGTTCTCCCAAATACAAGAATAGACTATTGA  2911278
```

```
>Leptospira interrogans strain FMAS_KG2 chromosome 1, complete sequence
Sequence ID: CP092985.1 Length: 4721761
Range 1: 3364108 to 3364694

Score:469 bits(519), Expect:2e-130,
Identities:456/587(78%),  Gaps:0/587(0%), Strand: Plus/Minus

Query  26       TCGACTGTAAAAAGAATGCGGAAGAGATTTTGGGAGAGGCAAAGGCAAAACCGGAATTGG  85
                |||| |||||||| || |||| ||||||||| | ||| |||||   |||| |||||| |
Sbjct  3364694  TCGATTGTAAAAAAAACGCGGTAGAGATTTTAGAAGAAGCAAAAAGAAAATCGGAATCGA  3364635

Query  86       TTCAAACTTTGGATTTTGGAATGCAGAAGTTGTCCACGGTTCCGGAAGAAGTTTGCGGTT  145
                ||||||  || ||||| |||||||| |||||  || || |||| |||| |||||||  ||
Sbjct  3364634  TTCAAATCTTAGATTTAGGAATGCAAAAGTTAACCTCGATTCCAGAAGGAGTTTGCTCTT  3364575

Query  146      TTCCGAATTTAACCAAGTTGGATCTTCGTTTAAACAGTTTGACTTTTCTTCCGGAATTTA  205
                |||||||||| ||  || ||||| | ||||| |||||||||| || |||||| |  |  |
Sbjct  3364574  TTCCGAATTTGACTCAGCTGGATTTACGTTTGAACAGTTTGAATTCTCTTCCTGGTTGGA  3364515

Query  206      TCGGAGAATGTAAACGCCTTGAACAACTTAATCTTTTCGGGAACGACCTTACAACATTTC  265
                | ||||  ||||||   || |||||| |||| |||||||| || || || |  ||  |||
Sbjct  3364514  TTGGAGCTTGTAAAAATCTGGAACAAATTAACCTTTTCGGAAATGATCTAAATACGGTTC  3364455

Query  266      CGTCTACGTTTTCTAAATTAAAAAATCTGAAAGTGTTACTGGCGGGAAATAACGATTTTA  325
                | ||| | |||||||||||||| ||| ||||||| || ||   ||| |||||||||||||
Sbjct  3364454  CATCTTCTTTTTCTAAATTAAAGAATTTGAAAGTATTGCTCTTGGGTAATAACGATTTTA  3364395

Query  326      CGATTCTACCCTCCGAACTTTTGTTTCTTCCGTTGATCAAAATTCTATACGTAGATCGGA  385
                |  ||||||| || |||||||||||||| || ||| | ||||  || ||| | ||||  |
Sbjct  3364394  CTTTTCTACCTTCGGAACTTTTGTTTCTGCCTTTGCTTAAAACCCTGTACTTGGATCAAA  3364335

Query  386      ATAAATTAACTCTAACGGAAACGGATGTGGAAATTCTCGCTTCTCTTTCCTCCTTGGAGG  445
                | ||  | ||  | || ||||||||||| |||||||| ||||||||||||   ||||| |
Sbjct  3364334  ACAAGCTTACGTTGACTGAAACGGATGTTGAAATTCTAGCTTCTCTTTCCAGTTTGGAAG  3364275

Query  446      AATTGGATCTGAGTCTGAATTCGGGGATTAAGGCGCTCCCTTTTAATTACGAAAAACTTG  505
                |||||||||| | | |||| || || || || | ||| |||| ||| ||  | ||| |
Sbjct  3364274  AATTGGATCTTAATTTGAACTCTGGAATCAAAGTGCTTCCTTCTAACTATAATAAATTGA  3364215

Query  506      TAAATCTCATTAATCTAAAAAGATTGAATATTaaaaaaaCTTCATTAAAGGGAGAGGATG  565
                 |||||| | | || ||||||||||||||||||||||||||||||||||||| |||||||
Sbjct  3364214  AAAATCTGACTCATTTAAAAAGATTGAATATTAAAAAAACTTCATTAAAGGGGGAGGATG  3364155

Query  566      CAGATAAATTGCAGGCGATTCTTCCAAATACCAAAATCGACTACTGA  612
                | |||||||| || ||  |||| |||||||| | ||| ||||| |||
Sbjct  3364154  CTGATAAATTACAAGCAGTTCTCCCAAATACAAGAATAGACTATTGA  3364108
```

```
>Leptospira interrogans strain FMAS_PD1 chromosome 1, complete sequence
Sequence ID: CP092743.1 Length: 4503505
Range 1: 2921059 to 2921645

Score:469 bits(519), Expect:2e-130,
Identities:456/587(78%),  Gaps:0/587(0%), Strand: Plus/Minus

Query  26       TCGACTGTAAAAAGAATGCGGAAGAGATTTTGGGAGAGGCAAAGGCAAAACCGGAATTGG  85
                |||| |||||||| || |||| ||||||||| | ||| |||||   |||| |||||| |
Sbjct  2921645  TCGATTGTAAAAAAAACGCGGTAGAGATTTTAGAAGAAGCAAAAAGAAAATCGGAATCGA  2921586
```

```
Query  86       TTCAAACTTTGGATTTTGGAATGCAGAAGTTGTCCACGGTTCCGGAAGAAGTTTGCGGTT  145
                ||||||  || ||||| |||||||| |||||  || || |||| |||| |||||||  ||
Sbjct  2921585  TTCAAATCTTAGATTTAGGAATGCAAAAGTTAACCTCGATTCCAGAAGGAGTTTGCTCTT  2921526

Query  146      TTCCGAATTTAACCAAGTTGGATCTTCGTTTAAACAGTTTGACTTTTCTTCCGGAATTTA  205
                |||||||||| ||  || ||||| | ||||| |||||||||| || |||||| |  |  |
Sbjct  2921525  TTCCGAATTTGACTCAGCTGGATTTACGTTTGAACAGTTTGAATTCTCTTCCTGGTTGGA  2921466

Query  206      TCGGAGAATGTAAACGCCTTGAACAACTTAATCTTTTCGGGAACGACCTTACAACATTTC  265
                | ||||  ||||||   || |||||| |||| |||||||| || || || |  ||  |||
Sbjct  2921465  TTGGAGCTTGTAAAAATCTGGAACAAATTAACCTTTTCGGAAATGATCTAAATACGGTTC  2921406

Query  266      CGTCTACGTTTTCTAAATTAAAAAATCTGAAAGTGTTACTGGCGGGAAATAACGATTTTA  325
                | ||| | |||||||||||||| ||| ||||||| || ||   ||| |||||||||||||
Sbjct  2921405  CATCTTCTTTTTCTAAATTAAAGAATTTGAAAGTATTGCTCTTGGGTAATAACGATTTTA  2921346

Query  326      CGATTCTACCCTCCGAACTTTTGTTTCTTCCGTTGATCAAAATTCTATACGTAGATCGGA  385
                |  ||||||| || |||||||||||||| || ||| | ||||  || ||| | ||||  |
Sbjct  2921345  CTTTTCTACCTTCGGAACTTTTGTTTCTGCCTTTGCTTAAAACCCTGTACTTGGATCAAA  2921286

Query  386      ATAAATTAACTCTAACGGAAACGGATGTGGAAATTCTCGCTTCTCTTTCCTCCTTGGAGG  445
                | ||  | ||  | || ||||||||||| |||||||| ||||||||||||   ||||| |
Sbjct  2921285  ACAAGCTTACGTTGACTGAAACGGATGTTGAAATTCTAGCTTCTCTTTCCAGTTTGGAAG  2921226

Query  446      AATTGGATCTGAGTCTGAATTCGGGGATTAAGGCGCTCCCTTTTAATTACGAAAAACTTG  505
                |||||||||| | | |||| || || || || | ||| |||| ||| ||  | ||| |
Sbjct  2921225  AATTGGATCTTAATTTGAACTCTGGAATCAAAGTGCTTCCTTCTAACTATAATAAATTGA  2921166

Query  506      TAAATCTCATTAATCTAAAAAGATTGAATATTaaaaaaaCTTCATTAAAGGGAGAGGATG  565
                 |||||| | | || ||||||||||||||||||||||||||||||||||||| |||||||
Sbjct  2921165  AAAATCTGACTCATTTAAAAAGATTGAATATTAAAAAAACTTCATTAAAGGGGGAGGATG  2921106

Query  566      CAGATAAATTGCAGGCGATTCTTCCAAATACCAAAATCGACTACTGA  612
                | |||||||| || ||  |||| |||||||| | ||| ||||| |||
Sbjct  2921105  CTGATAAATTACAAGCAGTTCTCCCAAATACAAGAATAGACTATTGA  2921059
```

>Leptospira interrogans strain FMAS_AW2 chromosome 1, complete sequence
Sequence ID: CP092739.1 Length: 4634565
Range 1: 1387600 to 1388186

Score:469 bits(519), Expect:2e-130,
Identities:456/587(78%), Gaps:0/587(0%), Strand: Plus/Plus

```
Query  26       TCGACTGTAAAAAGAATGCGGAAGAGATTTTGGGAGAGGCAAAGGCAAAACCGGAATTGG  85
                |||| |||||||| || |||| ||||||||| | ||| |||||   |||| |||||| |
Sbjct  1387600  TCGATTGTAAAAAAAAACGCGGTAGAGATTTTAGAAGAAGCAAAAAGAAAATCGGAATCGA  1387659

Query  86       TTCAAACTTTGGATTTTGGAATGCAGAAGTTGTCCACGGTTCCGGAAGAAGTTTGCGGTT  145
                ||||||  || ||||| |||||||| |||||  || || |||| |||| |||||||  ||
Sbjct  1387660  TTCAAATCTTAGATTTAGGAATGCAAAAGTTAACCTCGATTCCAGAAGGAGTTTGCTCTT  1387719

Query  146      TTCCGAATTTAACCAAGTTGGATCTTCGTTTAAACAGTTTGACTTTTCTTCCGGAATTTA  205
                |||||||||| ||  || ||||| | ||||| |||||||||| || |||||| |  |  |
Sbjct  1387720  TTCCGAATTTGACTCAGCTGGATTTACGTTTGAACAGTTTGAATTCTCTTCCTGGTTGGA  1387779

Query  206      TCGGAGAATGTAAACGCCTTGAACAACTTAATCTTTTCGGGAACGACCTTACAACATTTC  265
                | ||||  ||||||   || |||||| |||| |||||||| || || || |  ||  |||
Sbjct  1387780  TTGGAGCTTGTAAAAATCTGGAACAAATTAACCTTTTCGGAAATGATCTAAATACGGTTC  1387839
```

```
Query  266      CGTCTACGTTTTCTAAATTAAAAAATCTGAAAGTGTTACTGGCGGGAAATAACGATTTTA  325
                | ||| | |||||||||||||| ||| ||||||| |||||   ||| |||||||||||||
Sbjct  1387840  CATCTTCTTTTTCTAAATTAAAGAATTTGAAAGTATTACTCTTGGGTAATAACGATTTTA  1387899

Query  326      CGATTCTACCCTCCGAACTTTTGTTTCTTCCGTTGATCAAAATTCTATACGTAGATCGGA  385
                |  ||||||| || |||||||||||||| || ||| | ||||  || ||  | ||||  |
Sbjct  1387900  CTTTTCTACCTTCGGAACTTTTGTTTCTGCCTTTGCTTAAAACCCTGTATTTGGATCAAA  1387959

Query  386      ATAAATTAACTCTAACGGAAACGGATGTGGAAATTCTCGCTTCTCTTTCCTCCTTGGAGG  445
                | ||  | ||  | || ||||||||||| |||||||| ||||||||||||   ||||| |
Sbjct  1387960  ACAAGCTTACGTTGACTGAAACGGATGTTGAAATTCTAGCTTCTCTTTCCAGTTTGGAAG  1388019

Query  446      AATTGGATCTGAGTCTGAATTCGGGGATTAAGGCGCTCCCTTTTAATTACGAAAAACTTG  505
                |||||||||| | | |||| || || || || | ||| |||| ||| ||  | ||| |  
Sbjct  1388020  AATTGGATCTTAATTTGAACTCTGGAATCAAAGTGCTTCCTTCTAACTATAATAAATTGA  1388079

Query  506      TAAATCTCATTAATCTAAAAAGATTGAATATTaaaaaaaCTTCATTAAAGGGAGAGGATG  565
                 |||||| | | || ||||||||||||||||||||||||||||||||||||| |||||||
Sbjct  1388080  AAAATCTGACTCATTTAAAAAGATTGAATATTAAAAAAACTTCATTAAAGGGGGAGGATG  1388139

Query  566      CAGATAAATTGCAGGCGATTCTTCCAAATACCAAAATCGACTACTGA  612
                | |||||||| || ||  |||| |||||||| | ||| ||||| |||
Sbjct  1388140  CTGATAAATTACAAGCAGTTCTCCCAAATACAAGAATAGACTATTGA  1388186
```

>Leptospira interrogans strain FMAS_AW3 chromosome 1, complete sequence
Sequence ID: CP092676.1 Length: 4526110
Range 1: 1241524 to 1242110

Score:469 bits(519), Expect:2e-130,
Identities:456/587(78%), Gaps:0/587(0%), Strand: Plus/Plus

```
Query  26       TCGACTGTAAAAAGAATGCGGAAGAGATTTTGGGAGAGGCAAAGGCAAAACCGGAATTGG  85
                |||| |||||||| || |||| ||||||||| | ||| |||||   |||| |||||| | 
Sbjct  1241524  TCGATTGTAAAAAAAACGCGGTAGAGATTTTAGAAGAAGCAAAAAGAAAATCGGAATCGA  1241583

Query  86       TTCAAACTTTGGATTTTGGAATGCAGAAGTTGTCCACGGTTCCGGAAGAAGTTTGCGGTT  145
                ||||||  || ||||| |||||||| |||||  || || |||| |||| |||||||  ||
Sbjct  1241584  TTCAAATCTTAGATTTAGGAATGCAAAAGTTAACCTCGATTCCAGAAGGAGTTTGCTCTT  1241643

Query  146      TTCCGAATTTAACCAAGTTGGATCTTCGTTTAAACAGTTTGACTTTTCTTCCGGAATTTA  205
                |||||||||| ||  || ||||| | ||||| |||||||||| || |||||| |  |  |
Sbjct  1241644  TTCCGAATTTGACTCAGCTGGATTTACGTTTGAACAGTTTGAATTCTCTTCCTGGTTGGA  1241703

Query  206      TCGGAGAATGTAAACGCCTTGAACAACTTAATCTTTTCGGGAACGACCTTACAACATTTC  265
                | ||||  ||||||   || |||||| |||| |||||||| || || || |  ||  |||
Sbjct  1241704  TTGGAGCTTGTAAAAATCTGGAACAAATTAACCTTTTCGGAAATGATCTAAATACGGTTC  1241763

Query  266      CGTCTACGTTTTCTAAATTAAAAAATCTGAAAGTGTTACTGGCGGGAAATAACGATTTTA  325
                | ||| | |||||||||||||| ||| ||||||| |||||   ||| |||||||||||||
Sbjct  1241764  CATCTTCTTTTTCTAAATTAAAGAATTTGAAAGTATTACTCTTGGGTAATAACGATTTTA  1241823

Query  326      CGATTCTACCCTCCGAACTTTTGTTTCTTCCGTTGATCAAAATTCTATACGTAGATCGGA  385
                |  ||||||| || |||||||||||||| || ||| | ||||  || ||  | ||||  |
Sbjct  1241824  CTTTTCTACCTTCGGAACTTTTGTTTCTGCCTTTGCTTAAAACCCTGTATTTGGATCAAA  1241883

Query  386      ATAAATTAACTCTAACGGAAACGGATGTGGAAATTCTCGCTTCTCTTTCCTCCTTGGAGG  445
                | ||  | ||  | || ||||||||||| |||||||| ||||||||||||   ||||| |
Sbjct  1241884  ACAAGCTTACGTTGACTGAAACGGATGTTGAAATTCTAGCTTCTCTTTCCAGTTTGGAAG  1241943

Query  446      AATTGGATCTGAGTCTGAATTCGGGGATTAAGGCGCTCCCTTTTAATTACGAAAAACTTG  505
```

```
                ||||||||||| | | |||| || || || || | ||| |||| ||| ||  | ||| |
Sbjct  1241944  AATTGGATCTTAATTTGAACTCTGGAATCAAAGTGCTTCCTTCTAACTATAATAAATTGA  1242003

Query  506      TAAATCTCATTAATCTAAAAAGATTGAATATTaaaaaaaCTTCATTAAAGGGAGAGGATG  565
                 |||||| | | || ||||||||||||||||||||||||||||||||||||| |||||||
Sbjct  1242004  AAAATCTGACTCATTTAAAAAGATTGAATATTAAAAAAACTTCATTAAAGGGGGAGGATG  1242063

Query  566      CAGATAAATTGCAGGCGATTCTTCCAAATACCAAAATCGACTACTGA  612
                | |||||||| || ||  |||| |||||||| | ||| ||||| |||
Sbjct  1242064  CTGATAAATTACAAGCAGTTCTCCCAAATACAAGAATAGACTATTGA  1242110
```

>Leptospira interrogans strain FMAS_KG1 chromosome 1, complete sequence
Sequence ID: CP092672.1 Length: 4458643
Range 1: 3022201 to 3022787

Score:469 bits(519), Expect:2e-130,
Identities:456/587(78%), Gaps:0/587(0%), Strand: Plus/Minus

```
Query  26       TCGACTGTAAAAAGAATGCGGAAGAGATTTTGGGAGAGGCAAAGGCAAAACCGGAATTGG  85
                |||| |||||||| || |||| ||||||||| | ||| |||||   |||| |||||| |
Sbjct  3022787  TCGATTGTAAAAAAAACGCGGTAGAGATTTTAGAAGAAGCAAAAAGAAAATCGGAATCGA  3022728

Query  86       TTCAAACTTTGGATTTTGGAATGCAGAAGTTGTCCACGGTTCCGGAAGAAGTTTGCGGTT  145
                ||||||  || ||||| |||||||| |||||  || || |||| |||| |||||||  ||
Sbjct  3022727  TTCAAATCTTAGATTTAGGAATGCAAAAGTTAACCTCGATTCCAGAAGGAGTTTGCTCTT  3022668

Query  146      TTCCGAATTTAACCAAGTTGGATCTTCGTTTAAACAGTTTGACTTTTCTTCCGGAATTTA  205
                |||||||||| ||  || ||||| | ||||| |||||||||| || |||||| |  |  |
Sbjct  3022667  TTCCGAATTTGACTCAGCTGGATTTACGTTTGAACAGTTTGAATTCTCTTCCTGGTTGGA  3022608

Query  206      TCGGAGAATGTAAACGCCTTGAACAACTTAATCTTTTCGGGAACGACCTTACAACATTTC  265
                | ||||  ||||||   || |||||| |||| |||||||| || || || |  ||  |||
Sbjct  3022607  TTGGAGCTTGTAAAAATCTGGAACAAATTAACCTTTTCGGAAATGATCTAAATACGGTTC  3022548

Query  266      CGTCTACGTTTTCTAAATTAAAAAATCTGAAAGTGTTACTGGCGGGAAATAACGATTTTA  325
                | ||| | |||||||||||||| ||| ||||||| |||||   ||| |||||||||||||
Sbjct  3022547  CATCTTCTTTTTCTAAATTAAAGAATTTGAAAGTATTACTCTTGGGTAATAACGATTTTA  3022488

Query  326      CGATTCTACCCTCCGAACTTTTGTTTCTTCCGTTGATCAAAATTCTATACGTAGATCGGA  385
                |  ||||||| || |||||||||||||| || ||| | ||||  || ||  | ||||  |
Sbjct  3022487  CTTTTCTACCTTCGGAACTTTTGTTTCTGCCTTTGCTTAAAACCCTGTATTTGGATCAAA  3022428

Query  386      ATAAATTAACTCTAACGGAAACGGATGTGGAAATTCTCGCTTCTCTTTCCTCCTTGGAGG  445
                | ||  | ||  | || ||||||||||| |||||||| ||||||||||||   ||||| |
Sbjct  3022427  ACAAGCTTACGTTGACTGAAACGGATGTTGAAATTCTAGCTTCTCTTTCCAGTTTGGAAG  3022368

Query  446      AATTGGATCTGAGTCTGAATTCGGGGATTAAGGCGCTCCCTTTTAATTACGAAAAACTTG  505
                |||||||||| | | |||| || || || || | ||| |||| ||| ||  | ||| |
Sbjct  3022367  AATTGGATCTTAATTTGAACTCTGGAATCAAAGTGCTTCCTTCTAACTATAATAAATTGA  3022308

Query  506      TAAATCTCATTAATCTAAAAAGATTGAATATTaaaaaaaCTTCATTAAAGGGAGAGGATG  565
                 |||||| | | || ||||||||||||||||||||||||||||||||||||| |||||||
Sbjct  3022307  AAAATCTGACTCATTTAAAAAGATTGAATATTAAAAAAACTTCATTAAAGGGGGAGGATG  3022248

Query  566      CAGATAAATTGCAGGCGATTCTTCCAAATACCAAAATCGACTACTGA  612
                | |||||||| || ||  |||| |||||||| | ||| ||||| |||
Sbjct  3022247  CTGATAAATTACAAGCAGTTCTCCCAAATACAAGAATAGACTATTGA  3022201
```

```
>Leptospira interrogans strain FMAS_AP6 chromosome 1, complete sequence
Sequence ID: CP092156.1 Length: 4525852
Range 1: 2149342 to 2149928

Score:469 bits(519), Expect:2e-130,
Identities:456/587(78%),  Gaps:0/587(0%), Strand: Plus/Minus

Query  26       TCGACTGTAAAAAGAATGCGGAAGAGATTTTGGGAGAGGCAAAGGCAAAACCGGAATTGG  85
                |||| |||||||| || |||| ||||||||| | ||| |||||   |||| |||||| |
Sbjct  2149928  TCGATTGTAAAAAAAACGCGGTAGAGATTTTAGAAGAAGCAAAAAGAAAATCGGAATCGA  2149869

Query  86       TTCAAACTTTGGATTTTGGAATGCAGAAGTTGTCCACGGTTCCGGAAGAAGTTTGCGGTT  145
                ||||||  || ||||| |||||||| |||||  || || |||| |||| |||||||  ||
Sbjct  2149868  TTCAAATCTTAGATTTAGGAATGCAAAAGTTAACCTCGATTCCAGAAGGAGTTTGCTCTT  2149809

Query  146      TTCCGAATTTAACCAAGTTGGATCTTCGTTTAAACAGTTTGACTTTTCTTCCGGAATTTA  205
                |||||||||| ||  || ||||| | ||||| |||||||||| || |||||| |  |  |
Sbjct  2149808  TTCCGAATTTGACTCAGCTGGATTTACGTTTGAACAGTTTGAATTCTCTTCCTGGTTGGA  2149749

Query  206      TCGGAGAATGTAAACGCCTTGAACAACTTAATCTTTTCGGGAACGACCTTACAACATTTC  265
                | ||||  ||||||   || |||||| |||| |||||||| || || || |  ||  |||
Sbjct  2149748  TTGGAGCTTGTAAAAATCTGGAACAAATTAACCTTTTCGGAAATGATCTAAATACGGTTC  2149689

Query  266      CGTCTACGTTTTCTAAATTAAAAAATCTGAAAGTGTTACTGGCGGGAAATAACGATTTTA  325
                | ||| | |||||||||||||| ||| ||||||| |||||   ||| |||||||||||||
Sbjct  2149688  CATCTTCTTTTTCTAAATTAAAGAATTTGAAAGTATTACTCTTGGGTAATAACGATTTTA  2149629

Query  326      CGATTCTACCCTCCGAACTTTTGTTTCTTCCGTTGATCAAAATTCTATACGTAGATCGGA  385
                |  ||||||| || |||||||||||||| || ||| | ||||  || ||  | ||||  |
Sbjct  2149628  CTTTTCTACCTTCGGAACTTTTGTTTCTGCCTTTGCTTAAAACCCTGTATTTGGATCAAA  2149569

Query  386      ATAAATTAACTCTAACGGAAACGGATGTGGAAATTCTCGCTTCTCTTTCCTCCTTGGAGG  445
                | ||  | ||  | || ||||||||||| |||||||| ||||||||||||   ||||| |
Sbjct  2149568  ACAAGCTTACGTTGACTGAAACGGATGTTGAAATTCTAGCTTCTCTTTCCAGTTTGGAAG  2149509

Query  446      AATTGGATCTGAGTCTGAATTCGGGGATTAAGGCGCTCCCTTTTAATTACGAAAAACTTG  505
                |||||||||| | | |||| || || || || | ||| |||| ||| ||  | ||| |
Sbjct  2149508  AATTGGATCTTAATTTGAACTCTGGAATCAAAGTGCTTCCTTCTAACTATAATAAATTGA  2149449

Query  506      TAAATCTCATTAATCTAAAAAGATTGAATATTaaaaaaaCTTCATTAAAGGGAGAGGATG  565
                 |||||| | | || ||||||||||||||||||||||||||||||||||||| |||||||
Sbjct  2149448  AAAATCTGACTCATTTAAAAAGATTGAATATTAAAAAAACTTCATTAAAGGGGGAGGATG  2149389

Query  566      CAGATAAATTGCAGGCGATTCTTCCAAATACCAAAATCGACTACTGA  612
                | |||||||| || ||  |||| |||||||| | ||| ||||| |||
Sbjct  2149388  CTGATAAATTACAAGCAGTTCTCCCAAATACAAGAATAGACTATTGA  2149342



>Leptospira interrogans strain FMAS_AP5 chromosome 1, complete sequence
Sequence ID: CP092161.1 Length: 4478874
Range 1: 2832820 to 2833406

Score:469 bits(519), Expect:2e-130,
Identities:456/587(78%),  Gaps:0/587(0%), Strand: Plus/Plus

Query  26       TCGACTGTAAAAAGAATGCGGAAGAGATTTTGGGAGAGGCAAAGGCAAAACCGGAATTGG  85
                |||| |||||||| || |||| ||||||||| | ||| |||||   |||| |||||| |
Sbjct  2832820  TCGATTGTAAAAAAAACGCGGTAGAGATTTTAGAAGAAGCAAAAAGAAAATCGGAATCGA  2832879

Query  86       TTCAAACTTTGGATTTTGGAATGCAGAAGTTGTCCACGGTTCCGGAAGAAGTTTGCGGTT  145
```

```
                 ||||||  || ||||| |||||||| |||||  || || |||| |||| |||||||  ||
Sbjct  2832880   TTCAAATCTTAGATTTAGGAATGCAAAAGTTAACCTCGATTCCAGAAGGAGTTTGCTCTT  2832939

Query  146       TTCCGAATTTAACCAAGTTGGATCTTCGTTTAAACAGTTTGACTTTTCTTCCGGAATTTA  205
                 |||||||||| ||  || ||||| | ||||| |||||||||| || |||||| |  |  |
Sbjct  2832940   TTCCGAATTTGACTCAGCTGGATTTACGTTTGAACAGTTTGAATTCTCTTCCTGGTTGGA  2832999

Query  206       TCGGAGAATGTAAACGCCTTGAACAACTTAATCTTTTCGGGAACGACCTTACAACATTTC  265
                 | ||||  ||||||   || |||||| |||| |||||||| || || || |  ||  |||
Sbjct  2833000   TTGGAGCTTGTAAAAATCTGGAACAAATTAACCTTTTCGGAAATGATCTAAATACGGTTC  2833059

Query  266       CGTCTACGTTTTCTAAATTAAAAAATCTGAAAGTGTTACTGGCGGGAAATAACGATTTTA  325
                 | ||| | |||||||||||||| ||| ||||||| |||||   ||| |||||||||||||
Sbjct  2833060   CATCTTCTTTTTCTAAATTAAAGAATTTGAAAGTATTACTCTTGGGTAATAACGATTTTA  2833119

Query  326       CGATTCTACCCTCCGAACTTTTGTTTCTTCCGTTGATCAAAATTCTATACGTAGATCGGA  385
                 |  ||||||| || |||||||||||||| || ||| | ||||  || ||  | ||||  |
Sbjct  2833120   CTTTTCTACCTTCGGAACTTTTGTTTCTGCCTTTGCTTAAAACCCTGTATTTGGATCAAA  2833179

Query  386       ATAAATTAACTCTAACGGAAACGGATGTGGAAATTCTCGCTTCTCTTTCCTCCTTGGAGG  445
                 | ||  | ||  | || ||||||||||| ||||||||| ||||||||||||   ||||| |
Sbjct  2833180   ACAAGCTTACGTTGACTGAAACGGATGTTGAAATTCTAGCTTCTCTTTCCAGTTTGGAAG  2833239

Query  446       AATTGGATCTGAGTCTGAATTCGGGGATTAAGGCGCTCCCTTTTAATTACGAAAAACTTG  505
                 |||||||||| | | |||| || || || || | ||| |||| ||| ||  | ||| |
Sbjct  2833240   AATTGGATCTTAATTTGAACTCTGGAATCAAAGTGCTTCCTTCTAACTATAATAAATTGA  2833299

Query  506       TAAATCTCATTAATCTAAAAAGATTGAATATTaaaaaaaCTTCATTAAAGGGAGAGGATG  565
                  |||||| | | || ||||||||||||||||||||||||||||||||||||| |||||||
Sbjct  2833300   AAAATCTGACTCATTTAAAAAGATTGAATATTAAAAAAACTTCATTAAAGGGGGAGGATG  2833359

Query  566       CAGATAAATTGCAGGCGATTCTTCCAAATACCAAAATCGACTACTGA  612
                 | |||||||| || ||  |||| |||||||| | ||| ||||| |||
Sbjct  2833360   CTGATAAATTACAAGCAGTTCTCCCAAATACAAGAATAGACTATTGA  2833406



>Leptospira interrogans serovar Bataviae strain 1489 chromosome 1, complete sequence
Sequence ID: CP043893.1 Length: 4431091
Range 1: 3000720 to 3001306

Score:469 bits(519), Expect:2e-130,
Identities:456/587(78%),  Gaps:0/587(0%), Strand: Plus/Minus

Query  26        TCGACTGTAAAAAGAATGCGGAAGAGATTTTGGGAGAGGCAAAGGCAAAACCGGAATTGG  85
                 |||| |||||||| || |||| ||||||||| | ||| ||||||  |||| |||||| |
Sbjct  3001306   TCGATTGTAAAAAAAACGCGGTAGAGATTTTAGAAGAAGCAAAGAGAAAATCGGAATCGA  3001247

Query  86        TTCAAACTTTGGATTTTGGAATGCAGAAGTTGTCCACGGTTCCGGAAGAAGTTTGCGGTT  145
                 ||||||  || ||||| |||||||| |||||  || || |||| |||| |||||||  ||
Sbjct  3001246   TTCAAATCTTAGATTTAGGAATGCAAAAGTTAACCTCGATTCCAGAAGGAGTTTGCTCTT  3001187

Query  146       TTCCGAATTTAACCAAGTTGGATCTTCGTTTAAACAGTTTGACTTTTCTTCCGGAATTTA  205
                 |||||||||| ||  || ||||| | ||||| |||||||||| || |||||| |  |  |
Sbjct  3001186   TTCCGAATTTGACTCAGCTGGATTTACGTTTGAACAGTTTGAATTCTCTTCCTGGTTGGA  3001127

Query  206       TCGGAGAATGTAAACGCCTTGAACAACTTAATCTTTTCGGGAACGACCTTACAACATTTC  265
                 | ||||  ||||||   || |||||| |||| |||||||| || || || |  ||  |||
Sbjct  3001126   TTGGAGCTTGTAAAAATCTGGAACAAATTAACCTTTTCGGAAATGATCTAAATACGGTTC  3001067

Query  266       CGTCTACGTTTTCTAAATTAAAAAATCTGAAAGTGTTACTGGCGGGAAATAACGATTTTA  325
                 | ||| | |||||||||||||| ||| ||||||| |||||   ||| |||||||||||||
```

```
Sbjct  3001066  CATCTTCTTTTTCTAAATTAAAGAATTTGAAAGTATTACTCTTGGGTAATAACGATTTTA  3001007

Query  326      CGATTCTACCCTCCGAACTTTTGTTTCTTCCGTTGATCAAAATTCTATACGTAGATCGGA  385
                |  ||||||| || |||||||||||||| || ||| | ||||  || ||  | ||||  |
Sbjct  3001006  CTTTTCTACCTTCGGAACTTTTGTTTCTGCCTTTGCTTAAAACCCTGTATTTGGATCAAA  3000947

Query  386      ATAAATTAACTCTAACGGAAACGGATGTGGAAATTCTCGCTTCTCTTTCCTCCTTGGAGG  445
                | ||  | ||  | || ||||||||||| |||||| | ||||||||||||   ||||| |
Sbjct  3000946  ACAAGCTTACGTTGACTGAAACGGATGTTGAAATTTTAGCTTCTCTTTCCAGTTTGGAAG  3000887

Query  446      AATTGGATCTGAGTCTGAATTCGGGGATTAAGGCGCTCCCTTTTAATTACGAAAAACTTG  505
                |||||||||| | | |||| || || || || | ||| |||| ||| ||  | ||| |
Sbjct  3000886  AATTGGATCTTAATTTGAACTCTGGAATCAAAGTGCTTCCTTCTAACTATAATAAATTGA  3000827

Query  506      TAAATCTCATTAATCTAAAAAGATTGAATATTaaaaaaaCTTCATTAAAGGGAGAGGATG  565
                 |||||| | | || ||||||||||||||||||||||||||||||||||||| |||||||
Sbjct  3000826  AAAATCTGACTCATTTAAAAAGATTGAATATTAAAAAAACTTCATTAAAGGGGGAGGATG  3000767

Query  566      CAGATAAATTGCAGGCGATTCTTCCAAATACCAAAATCGACTACTGA  612
                | |||||||| || ||  |||| |||||||| | ||| ||||| |||
Sbjct  3000766  CTGATAAATTACAAGCAGTTCTCCCAAATACAAGAATAGACTATTGA  3000720
```

>Leptospira interrogans serovar Icterohaemorrhagiae strain 898 chromosome 1, complete sequence
Sequence ID: CP043891.1 Length: 4280412
Range 1: 2909276 to 2909862

Score:469 bits(519), Expect:2e-130,
Identities:456/587(78%), Gaps:0/587(0%), Strand: Plus/Minus

```
Query  26       TCGACTGTAAAAAGAATGCGGAAGAGATTTTGGGAGAGGCAAAGGCAAAACCGGAATTGG  85
                |||| |||||||| || |||| ||||||||| | ||| |||||   |||| |||||| |
Sbjct  2909862  TCGATTGTAAAAAAAACGCGGTAGAGATTTTAGAAGAAGCAAAAAGAAAATCGGAATCGA  2909803

Query  86       TTCAAACTTTGGATTTTGGAATGCAGAAGTTGTCCACGGTTCCGGAAGAAGTTTGCGGTT  145
                ||||||  || ||||| |||||||| |||||  || || |||| |||| |||||||  ||
Sbjct  2909802  TTCAAATCTTAGATTTAGGAATGCAAAAGTTAACCTCGATTCCAGAAGGAGTTTGCTCTT  2909743

Query  146      TTCCGAATTTAACCAAGTTGGATCTTCGTTTAAACAGTTTGACTTTTCTTCCGGAATTTA  205
                |||||||||| ||  || ||||| | ||||| |||||||||| || |||||| |  |  |
Sbjct  2909742  TTCCGAATTTGACTCAGCTGGATTTACGTTTGAACAGTTTGAATTCTCTTCCTGGTTGGA  2909683

Query  206      TCGGAGAATGTAAACGCCTTGAACAACTTAATCTTTTCGGGAACGACCTTACAACATTTC  265
                | ||||  ||||||   || |||||| |||| |||||||| || || || |  ||  |||
Sbjct  2909682  TTGGAGCTTGTAAAAATCTGGAACAAATTAACCTTTTCGGAAATGATCTAAATACGGTTC  2909623

Query  266      CGTCTACGTTTTCTAAATTAAAAAATCTGAAAGTGTTACTGGCGGGAAATAACGATTTTA  325
                | ||| | |||||||||||||| ||| ||||||| |||||   ||| |||||||||||||
Sbjct  2909622  CATCTTCTTTTTCTAAATTAAAGAATTTGAAAGTATTACTCTTGGGTAATAACGATTTTA  2909563

Query  326      CGATTCTACCCTCCGAACTTTTGTTTCTTCCGTTGATCAAAATTCTATACGTAGATCGGA  385
                |  ||||||| || |||||||||||||| || ||| | ||||  || ||  | ||||  |
Sbjct  2909562  CTTTTCTACCTTCGGAACTTTTGTTTCTGCCTTTGCTTAAAACCCTGTATTTGGATCAAA  2909503

Query  386      ATAAATTAACTCTAACGGAAACGGATGTGGAAATTCTCGCTTCTCTTTCCTCCTTGGAGG  445
                | ||  | ||  | || ||||||||||| |||||||| ||||||||||||   ||||| |
Sbjct  2909502  ACAAGCTTACGTTGACTGAAACGGATGTTGAAATTCTAGCTTCTCTTTCCAGTTTGGAAG  2909443

Query  446      AATTGGATCTGAGTCTGAATTCGGGGATTAAGGCGCTCCCTTTTAATTACGAAAAACTTG  505
                |||||||||| | | |||| || || || || | ||| |||| ||| ||  | ||| |
```

```
Sbjct  2909442  AATTGGATCTTAATTTGAACTCTGGAATCAAAGTGCTTCCTTCTAACTATAATAAATTGA  2909383

Query  506      TAAATCTCATTAATCTAAAAAGATTGAATATTaaaaaaCTTCATTAAAGGGAGAGGATG  565
                 |||||| | | || ||||||||||||||||||||||||||||||||||||| |||||||
Sbjct  2909382  AAAATCTGACTCATTTAAAAAGATTGAATATTAAAAAACTTCATTAAAGGGGGAGGATG  2909323

Query  566      CAGATAAATTGCAGGCGATTCTTCCAAATACCAAAATCGACTACTGA  612
                | |||||||| || ||  |||| |||||||| | ||| ||||| |||
Sbjct  2909322  CTGATAAATTACAAGCAGTTCTCCCAAATACAAGAATAGACTATTGA  2909276
```

>Leptospira interrogans serovar Icterohaemorrhagiae strain Langkawi chromosome 1, complete sequence
Sequence ID: CP043876.1 Length: 4367462
Range 1: 2966377 to 2966963

Score:469 bits(519), Expect:2e-130,
Identities:456/587(78%), Gaps:0/587(0%), Strand: Plus/Minus

```
Query  26       TCGACTGTAAAAAGAATGCGGAAGAGATTTTGGGAGAGGCAAAGGCAAAACCGGAATTGG  85
                |||| |||||||| || |||| ||||||||| | ||| |||||   |||| |||||| |
Sbjct  2966963  TCGATTGTAAAAAAAACGCGGTAGAGATTTTAGAAGAAGCAAAAAGAAAATCGGAATCGA  2966904

Query  86       TTCAAACTTTGGATTTTGGAATGCAGAAGTTGTCCACGGTTCCGGAAGAAGTTTGCGGTT  145
                ||||||  || ||||| |||||||| |||||  || || |||| |||| |||||||  ||
Sbjct  2966903  TTCAAATCTTAGATTTAGGAATGCAAAAGTTAACCTCGATTCCAGAAGGAGTTTGCTCTT  2966844

Query  146      TTCCGAATTTAACCAAGTTGGATCTTCGTTTAAACAGTTTGACTTTTCTTCCGGAATTTA  205
                |||||||||| ||  || ||||| | ||||| |||||||||| || |||||| |  |  |
Sbjct  2966843  TTCCGAATTTGACTCAGCTGGATTTACGTTTGAACAGTTTGAATTCTCTTCCTGGTTGGA  2966784

Query  206      TCGGAGAATGTAAACGCCTTGAACAACTTAATCTTTTCGGGAACGACCTTACAACATTTC  265
                | ||||  ||||||   || |||||| |||| |||||||| || || || |  ||  |||
Sbjct  2966783  TTGGAGCTTGTAAAAATCTGGAACAAATTAACCTTTTCGGAAATGATCTAAATACGGTTC  2966724

Query  266      CGTCTACGTTTTCTAAATTAAAAAATCTGAAAGTGTTACTGGCGGGAAATAACGATTTTA  325
                | ||| | |||||||||||||| ||| ||||||| |||||   ||| |||||||||||||
Sbjct  2966723  CATCTTCTTTTTCTAAATTAAAGAATTTGAAAGTATTACTCTTGGGTAATAACGATTTTA  2966664

Query  326      CGATTCTACCCTCCGAACTTTTGTTTCTTCCGTTGATCAAAATTCTATACGTAGATCGGA  385
                |  ||||||| || |||||||||||||| || ||| | ||||  || ||  | ||||  |
Sbjct  2966663  CTTTTCTACCTTCGGAACTTTTGTTTCTGCCTTTGCTTAAAACCCTGTATTTGGATCAAA  2966604

Query  386      ATAAATTAACTCTAACGGAAACGGATGTGGAAATTCTCGCTTCTCTTTCCTCCTTGGAGG  445
                | ||  | ||  | || ||||||||||| |||||||| ||||||||||||   ||||| |
Sbjct  2966603  ACAAGCTTACGTTGACTGAAACGGATGTTGAAATTCTAGCTTCTCTTTCCAGTTTGGAAG  2966544

Query  446      AATTGGATCTGAGTCTGAATTCGGGGATTAAGGCGCTCCCTTTTAATTACGAAAAACTTG  505
                |||||||||| | | |||| || || || || | ||| |||| ||| ||  | ||| |
Sbjct  2966543  AATTGGATCTTAATTTGAACTCTGGAATCAAAGTGCTTCCTTCTAACTATAATAAATTGA  2966484

Query  506      TAAATCTCATTAATCTAAAAAGATTGAATATTaaaaaaCTTCATTAAAGGGAGAGGATG  565
                 |||||| | | || ||||||||||||||||||||||||||||||||||||| |||||||
Sbjct  2966483  AAAATCTGACTCATTTAAAAAGATTGAATATTAAAAAACTTCATTAAAGGGGGAGGATG  2966424

Query  566      CAGATAAATTGCAGGCGATTCTTCCAAATACCAAAATCGACTACTGA  612
                | |||||||| || ||  |||| |||||||| | ||| ||||| |||
Sbjct  2966423  CTGATAAATTACAAGCAGTTCTCCCAAATACAAGAATAGACTATTGA  2966377
```

```
>Leptospira interrogans serovar Lai str. IPAV chromosome 1, complete sequence
Sequence ID: CP001221.1 Length: 4349158
Range 1: 1315702 to 1316288

Score:469 bits(519), Expect:2e-130,
Identities:456/587(78%),  Gaps:0/587(0%), Strand: Plus/Plus

Query  26       TCGACTGTAAAAAGAATGCGGAAGAGATTTTGGGAGAGGCAAAGGCAAAACCGGAATTGG  85
                |||| |||||||| || |||| ||||||||| | ||| |||||   |||| |||||| |
Sbjct  1315702  TCGATTGTAAAAAAAACGCGGTAGAGATTTTAGAAGAAGCAAAAAGAAAATCGGAATCGA  1315761

Query  86       TTCAAACTTTGGATTTTGGAATGCAGAAGTTGTCCACGGTTCCGGAAGAAGTTTGCGGTT  145
                ||||||  || ||||| |||||||| |||||  || || |||| |||| |||||||  ||
Sbjct  1315762  TTCAAATCTTAGATTTAGGAATGCAAAAGTTAACCTCGATTCCAGAAGGAGTTTGCTCTT  1315821

Query  146      TTCCGAATTTAACCAAGTTGGATCTTCGTTTAAACAGTTTGACTTTTCTTCCGGAATTTA  205
                |||||||||| ||  || ||||| | ||||| |||||||||| || |||||| |  |  |
Sbjct  1315822  TTCCGAATTTGACTCAGCTGGATTTACGTTTGAACAGTTTGAATTCTCTTCCTGGTTGGA  1315881

Query  206      TCGGAGAATGTAAACGCCTTGAACAACTTAATCTTTTCGGGAACGACCTTACAACATTTC  265
                | ||||  ||||||   || |||||| |||| |||||||| || || || |  ||  |||
Sbjct  1315882  TTGGAGCTTGTAAAAATCTGGAACAAATTAACCTTTTCGGAAATGATCTAAATACGGTTC  1315941

Query  266      CGTCTACGTTTTCTAAATTAAAAAATCTGAAAGTGTTACTGGCGGGAAATAACGATTTTA  325
                | ||| | |||||||||||||| ||| ||||||| |||||   ||| |||||||||||||
Sbjct  1315942  CATCTTCTTTTTCTAAATTAAAGAATTTGAAAGTATTACTCTTGGGTAATAACGATTTTA  1316001

Query  326      CGATTCTACCCTCCGAACTTTTGTTTCTTCCGTTGATCAAAATTCTATACGTAGATCGGA  385
                |  ||||||| || |||||||||||||| || ||| | ||||  || ||  | ||||  |
Sbjct  1316002  CTTTTCTACCTTCGGAACTTTTGTTTCTGCCTTTGCTTAAAACCCTGTATTTGGATCAAA  1316061

Query  386      ATAAATTAACTCTAACGGAAACGGATGTGGAAATTCTCGCTTCTCTTTCCTCCTTGGAGG  445
                | ||  | ||  | || ||||||||||| |||||||| ||||||||||||   ||||| |
Sbjct  1316062  ACAAGCTTACGTTGACTGAAACGGATGTTGAAATTCTAGCTTCTCTTTCCAGTTTGGAAG  1316121

Query  446      AATTGGATCTGAGTCTGAATTCGGGGATTAAGGCGCTCCCTTTTAATTACGAAAAACTTG  505
                |||||||||| | | |||| || || || || | ||| |||| ||| ||  | ||| |
Sbjct  1316122  AATTGGATCTTAATTTGAACTCTGGAATCAAAGTGCTTCCTTCTAACTATAATAAATTGA  1316181

Query  506      TAAATCTCATTAATCTAAAAAGATTGAATATTaaaaaaaCTTCATTAAAGGGAGAGGATG  565
                 |||||| | | || ||||||||||||||||||||||||||||||||||||| |||||||
Sbjct  1316182  AAAATCTGACTCATTTAAAAAGATTGAATATTAAAAAAACTTCATTAAAGGGGGAGGATG  1316241

Query  566      CAGATAAATTGCAGGCGATTCTTCCAAATACCAAAATCGACTACTGA  612
                | |||||||| || ||  |||| |||||||| | ||| ||||| |||
Sbjct  1316242  CTGATAAATTACAAGCAGTTCTCCCAAATACAAGAATAGACTATTGA  1316288



>Leptospira interrogans serovar Lai str. 56601 chromosome I, complete sequence
Sequence ID: AE010300.2 Length: 4338762
Range 1: 1316631 to 1317217

Score:469 bits(519), Expect:2e-130,
Identities:456/587(78%),  Gaps:0/587(0%), Strand: Plus/Plus

Query  26       TCGACTGTAAAAAGAATGCGGAAGAGATTTTGGGAGAGGCAAAGGCAAAACCGGAATTGG  85
                |||| |||||||| || |||| ||||||||| | ||| |||||   |||| |||||| |
Sbjct  1316631  TCGATTGTAAAAAAAACGCGGTAGAGATTTTAGAAGAAGCAAAAAGAAAATCGGAATCGA  1316690

Query  86       TTCAAACTTTGGATTTTGGAATGCAGAAGTTGTCCACGGTTCCGGAAGAAGTTTGCGGTT  145
```

```
                 |||||||  || ||||| |||||||| |||||  || || |||| |||| |||||||  ||
Sbjct  1316691  TTCAAATCTTAGATTTAGGAATGCAAAAGTTAACCTCGATTCCAGAAGGAGTTTGCTCTT  1316750

Query  146      TTCCGAATTTAACCAAGTTGGATCTTCGTTTAAACAGTTTGACTTTTCTTCCGGAATTTA  205
                |||||||||| ||  || ||||| | ||||| |||||||||| || |||||| |  |  |
Sbjct  1316751  TTCCGAATTTGACTCAGCTGGATTTACGTTTGAACAGTTTGAATTCTCTTCCTGGTTGGA  1316810

Query  206      TCGGAGAATGTAAACGCCTTGAACAACTTAATCTTTTCGGGAACGACCTTACAACATTTC  265
                | ||||  ||||||   || |||||| |||| |||||||| || || || |  ||  |||
Sbjct  1316811  TTGGAGCTTGTAAAAATCTGGAACAAATTAACCTTTTCGGAAATGATCTAAATACGGTTC  1316870

Query  266      CGTCTACGTTTTCTAAATTAAAAAATCTGAAAGTGTTACTGGCGGGAAATAACGATTTTA  325
                | ||| | |||||||||||||| ||| ||||||| |||||   ||| |||||||||||||
Sbjct  1316871  CATCTTCTTTTTCTAAATTAAAGAATTTGAAAGTATTACTCTTGGGTAATAACGATTTTA  1316930

Query  326      CGATTCTACCCTCCGAACTTTTGTTTCTTCCGTTGATCAAAATTCTATACGTAGATCGGA  385
                |  ||||||| || |||||||||||||| || ||| | ||||  || ||  | ||||  |
Sbjct  1316931  CTTTTCTACCTTCGGAACTTTTGTTTCTGCCTTTGCTTAAAACCCTGTATTTGGATCAAA  1316990

Query  386      ATAAATTAACTCTAACGGAAACGGATGTGGAAATTCTCGCTTCTCTTTCCTCCTTGGAGG  445
                | ||  | ||  | || ||||||||||| |||||||| ||||||||||||   ||||| |
Sbjct  1316991  ACAAGCTTACGTTGACTGAAACGGATGTTGAAATTCTAGCTTCTCTTTCCAGTTTGGAAG  1317050

Query  446      AATTGGATCTGAGTCTGAATTCGGGGATTAAGGCGCTCCCTTTTAATTACGAAAAACTTG  505
                |||||||||| | | |||| || || || || | ||| |||| ||| ||  | ||| |
Sbjct  1317051  AATTGGATCTTAATTTGAACTCTGGAATCAAAGTGCTTCCTTCTAACTATAATAAATTGA  1317110

Query  506      TAAATCTCATTAATCTAAAAAGATTGAATATTaaaaaaaCTTCATTAAAGGGAGAGGATG  565
                 |||||| | | || ||||||||||||||||||||||||||||||||||||| |||||||
Sbjct  1317111  AAAATCTGACTCATTTAAAAAGATTGAATATTAAAAAAACTTCATTAAAGGGGGAGGATG  1317170

Query  566      CAGATAAATTGCAGGCGATTCTTCCAAATACCAAAATCGACTACTGA  612
                | |||||||| || ||  |||| |||||||| | ||| ||||| |||
Sbjct  1317171  CTGATAAATTACAAGCAGTTCTCCCAAATACAAGAATAGACTATTGA  1317217
```

>Leptospira interrogans serovar Copenhageni str. Fiocruz L1-130, chromosome I, complete sequence
Sequence ID: AE016823.1 Length: 4277185
Range 1: 2909058 to 2909644

Score:469 bits(519), Expect:2e-130,
Identities:456/587(78%), Gaps:0/587(0%), Strand: Plus/Minus

```
Query  26       TCGACTGTAAAAAGAATGCGGAAGAGATTTTGGGAGAGGCAAAGGCAAAACCGGAATTGG  85
                |||| |||||||| || |||| ||||||||| | ||| |||||   |||| |||||| |
Sbjct  2909644  TCGATTGTAAAAAAAACGCGGTAGAGATTTTAGAAGAAGCAAAAAGAAAATCGGAATCGA  2909585

Query  86       TTCAAACTTTGGATTTTGGAATGCAGAAGTTGTCCACGGTTCCGGAAGAAGTTTGCGGTT  145
                ||||||  || ||||| |||||||| |||||  || || |||| |||| |||||||  ||
Sbjct  2909584  TTCAAATCTTAGATTTAGGAATGCAAAAGTTAACCTCGATTCCAGAAGGAGTTTGCTCTT  2909525

Query  146      TTCCGAATTTAACCAAGTTGGATCTTCGTTTAAACAGTTTGACTTTTCTTCCGGAATTTA  205
                |||||||||| ||  || ||||| | ||||| |||||||||| || |||||| |  |  |
Sbjct  2909524  TTCCGAATTTGACTCAGCTGGATTTACGTTTGAACAGTTTGAATTCTCTTCCTGGTTGGA  2909465

Query  206      TCGGAGAATGTAAACGCCTTGAACAACTTAATCTTTTCGGGAACGACCTTACAACATTTC  265
                | ||||  ||||||   || |||||| |||| |||||||| || || || |  ||  |||
Sbjct  2909464  TTGGAGCTTGTAAAAATCTGGAACAAATTAACCTTTTCGGAAATGATCTAAATACGGTTC  2909405

Query  266      CGTCTACGTTTTCTAAATTAAAAAATCTGAAAGTGTTACTGGCGGGAAATAACGATTTTA  325
```

```
                   | ||| | |||||||||||||| ||| ||||||| |||||   ||| |||||||||||||
Sbjct  2909404  CATCTTCTTTTTCTAAATTAAAGAATTTGAAAGTATTACTCTTGGGTAATAACGATTTA  2909345

Query  326      CGATTCTACCCTCCGAACTTTTGTTTCTTCCGTTGATCAAAATTCTATACGTAGATCGGA  385
                |  ||||||| || |||||||||||||| || ||| | ||||  || ||  | ||||  |
Sbjct  2909344  CTTTTCTACCTTCGGAACTTTTGTTTCTGCCTTTGCTTAAAACCCTGTATTTGGATCAAA  2909285

Query  386      ATAAATTAACTCTAACGGAAACGGATGTGGAAATTCTCGCTTCTCTTTCCTCCTTGGAGG  445
                | ||  | ||  | || ||||||||||| |||||||| ||||||||||||   ||||| |
Sbjct  2909284  ACAAGCTTACGTTGACTGAAACGGATGTTGAAATTCTAGCTTCTCTTTCCAGTTTGGAAG  2909225

Query  446      AATTGGATCTGAGTCTGAATTCGGGGATTAAGGCGCTCCCTTTTAATTACGAAAAACTTG  505
                |||||||||| | | |||| || || || || | ||| |||| ||| ||  | ||| |
Sbjct  2909224  AATTGGATCTTAATTTGAACTCTGGAATCAAAGTGCTTCCTTCTAACTATAATAAATTGA  2909165

Query  506      TAAATCTCATTAATCTAAAAAGATTGAATATTaaaaaaaCTTCATTAAAGGGAGAGGATG  565
                 |||||| | | || |||||||||||||||||||||||||||||||||||||| |||||||
Sbjct  2909164  AAAATCTGACTCATTTAAAAAGATTGAATATTAAAAAAACTTCATTAAAGGGGGAGGATG  2909105

Query  566      CAGATAAATTGCAGGCGATTCTTCCAAATACCAAAATCGACTACTGA  612
                | |||||||| || ||  |||| |||||||| | ||| ||||| |||
Sbjct  2909104  CTGATAAATTACAAGCAGTTCTCCCAAATACAAGAATAGACTATTGA  2909058
```

>Leptospira interrogans serovar Copenhageni/Icterohaemorrhagiae strain Piscina chromosome 1
Sequence ID: CP018146.1 Length: 4221879
Range 1: 2867106 to 2867691

Score:465 bits(515), Expect:2e-129,
Identities:456/587(78%), Gaps:1/587(0%), Strand: Plus/Minus

```
Query  26       TCGACTGTAAAAAGAATGCGGAAGAGATTTTGGGAGAGGCAAAGGCAAAACCGGAATTGG  85
                |||| |||||||| || |||| ||||||||| | ||| |||||   |||| |||||| |
Sbjct  2867691  TCGATTGTAAAAA-AACGCGGTAGAGATTTTAGAAGAAGCAAAAAGAAAATCGGAATCGA  2867633

Query  86       TTCAAACTTTGGATTTTGGAATGCAGAAGTTGTCCACGGTTCCGGAAGAAGTTTGCGGTT  145
                ||||||  || ||||| |||||||| |||||  || || |||| |||| |||||||  ||
Sbjct  2867632  TTCAAATCTTAGATTTAGGAATGCAAAAGTTAACCTCGATTCCAGAAGGAGTTTGCTCTT  2867573

Query  146      TTCCGAATTTAACCAAGTTGGATCTTCGTTTAAACAGTTTGACTTTTCTTCCGGAATTTA  205
                |||||||||| ||  || ||||| | ||||| |||||||||| || |||||| |  |  |
Sbjct  2867572  TTCCGAATTTGACTCAGCTGGATTTACGTTTGAACAGTTTGAATTCTCTTCCTGGTTGGA  2867513

Query  206      TCGGAGAATGTAAACGCCTTGAACAACTTAATCTTTTCGGGAACGACCTTACAACATTTC  265
                | ||||  ||||||   || |||||| |||| |||||||| || || || |  ||  |||
Sbjct  2867512  TTGGAGCTTGTAAAAATCTGGAACAAATTAACCTTTTCGGAAATGATCTAAATACGGTTC  2867453

Query  266      CGTCTACGTTTTCTAAATTAAAAAATCTGAAAGTGTTACTGGCGGGAAATAACGATTTA  325
                | ||| | |||||||||||||| ||| ||||||| |||||   ||| |||||||||||||
Sbjct  2867452  CATCTTCTTTTTCTAAATTAAAGAATTTGAAAGTATTACTCTTGGGTAATAACGATTTA  2867393

Query  326      CGATTCTACCCTCCGAACTTTTGTTTCTTCCGTTGATCAAAATTCTATACGTAGATCGGA  385
                |  ||||||| || |||||||||||||| || ||| | ||||  || ||  | ||||  |
Sbjct  2867392  CTTTTCTACCTTCGGAACTTTTGTTTCTGCCTTTGCTTAAAACCCTGTATTTGGATCAAA  2867333

Query  386      ATAAATTAACTCTAACGGAAACGGATGTGGAAATTCTCGCTTCTCTTTCCTCCTTGGAGG  445
                | ||  | ||  | || ||||||||||| |||||||| ||||||||||||   ||||| |
Sbjct  2867332  ACAAGCTTACGTTGACTGAAACGGATGTTGAAATTCTAGCTTCTCTTTCCAGTTTGGAAG  2867273

Query  446      AATTGGATCTGAGTCTGAATTCGGGGATTAAGGCGCTCCCTTTTAATTACGAAAAACTTG  505
```

```
                |||||||||| | | |||| || || || || | ||| |||| ||| ||  | ||| |
Sbjct  2867272  AATTGGATCTTAATTTGAACTCTGGAATCAAAGTGCTTCCTTCTAACTATAATAAATTGA  2867213

Query  506      TAAATCTCATTAATCTAAAAAGATTGAATATTaaaaaaaCTTCATTAAAGGGAGAGGATG  565
                 |||||| | | || ||||||||||||||||||||||||||||||||||||| |||||||
Sbjct  2867212  AAAATCTGACTCATTTAAAAAGATTGAATATTAAAAAAACTTCATTAAAGGGGGAGGATG  2867153

Query  566      CAGATAAATTGCAGGCGATTCTTCCAAATACCAAAATCGACTACTGA  612
                | |||||||| || ||  |||| |||||||| | ||| ||||| |||
Sbjct  2867152  CTGATAAATTACAAGCAGTTCTCCCAAATACAAGAATAGACTATTGA  2867106
```

>Leptospira interrogans serovar Canicola leucine-rich repeat protein gene, partial cds
Sequence ID: JX426070.1 Length: 410
Range 1: 3 to 407

Score:325 bits(360), Expect:1e-87,
Identities:315/405(78%), Gaps:0/405(0%), Strand: Plus/Plus

```
Query  192  TCTTCCGGAATTTATCGGAGAATGTAAACGCCTTGAACAACTTAATCTTTTCGGGAACGA  251
            |||||| |  |  || ||||  ||||||   || |||||| |||| |||||||| || ||
Sbjct  3    TCTTCCTGGTTGGATTGGAGCTTGTAAAAATCTGGAACAAATTAACCTTTTCGGAAATGA  62

Query  252  CCTTACAACATTTCCGTCTACGTTTTCTAAATTAAAAAATCTGAAAGTGTTACTGGCGGG  311
             || |  ||  |||| ||| | |||||||||||||| ||| ||||||| || ||   |||
Sbjct  63   TCTAAATACGGTTCCATCTTCTTTTTCTAAATTAAAGAATTTGAAAGTATTGCTCTTGGG  122

Query  312  AAATAACGATTTTACGATTCTACCCTCCGAACTTTTGTTTCTTCCGTTGATCAAAATTCT  371
             ||||||||||||||  ||||||| || |||||||||||||| || ||||| ||||  ||
Sbjct  123  TAATAACGATTTTACTTTTCTACCTTCGGAACTTTTGTTTCTGCCTTTGATTAAAACCCT  182

Query  372  ATACGTAGATCGGAATAAATTAACTCTAACGGAAACGGATGTGGAAATTCTCGCTTCTCT  431
             ||| | ||||  || ||  | ||  | || ||||||||||| |||||||| ||||||||
Sbjct  183  GTACTTGGATCAAAACAAGCTTACGTTGACCGAAACGGATGTTGAAATTCTAGCTTCTCT  242

Query  432  TTCCTCCTTGGAGGAATTGGATCTGAGTCTGAATTCGGGGATTAAGGCGCTCCCTTTTAA  491
            ||||   ||||| ||||||||||| | | |||| || || ||||| | ||| |||| |||
Sbjct  243  TTCCAGTTTGGAAGAATTGGATCTTAATTTGAACTCTGGAATTAAAGTGCTTCCTTCTAA  302

Query  492  TTACGAAAAACTTGTAAATCTCATTAATCTAAAAAGATTGAATATTaaaaaaaCTTCATT  551
             ||  | ||| |   |||||| | | || |||||||||||||||||||||||||||||||
Sbjct  303  CTATAATAAATTGAAAAATCTGACTCATTTAAAAAGATTGAATATTAAAAAAACTTCATT  362

Query  552  AAAGGGAGAGGATGCAGATAAATTGCAGGCGATTCTTCCAAATAC  596
            ||||||||| ||||| |||||||| || ||  |||| ||||||||
Sbjct  363  AAAGGGAGAAGATGCTGATAAATTACAAGCAGTTCTCCCAAATAC  407
```

>Leptospira interrogans serovar Autumnalis leucine-rich repeat protein gene, partial cds
Sequence ID: JX426069.1 Length: 411
Range 1: 3 to 407

Score:321 bits(355), Expect:5e-86,
Identities:314/405(78%), Gaps:0/405(0%), Strand: Plus/Plus

```
Query  192  TCTTCCGGAATTTATCGGAGAATGTAAACGCCTTGAACAACTTAATCTTTTCGGGAACGA  251
            |||||| |  |  || ||||  ||||||   || |||||| |||| |||||||| || ||
Sbjct  3    TCTTCCTGGTTGGATTGGAGCTTGTAAAAATCTGGAACAAATTAACCTTTTCGGAAATGA  62
```

```
Query  252  CCTTACAACATTTCCGTCTACGTTTTCTAAATTAAAAAATCTGAAAGTGTTACTGGCGGG  311
             || |  ||  |||| ||| | |||||||||||||| ||| ||||||| || ||   |||
Sbjct  63   TCTAAATACGGTTCCATCTTCTTTTTCTAAATTAAAGAATTTGAAAGTATTGCTCTTGGG  122

Query  312  AAATAACGATTTTACGATTCTACCCTCCGAACTTTTGTTTCTTCCGTTGATCAAAATTCT  371
             ||||||||||||||  ||||||| || |||||||||||||| || ||||| ||||  ||
Sbjct  123  TAATAACGATTTTACTTTTCTACCTTCGGAACTTTTGTTTCTGCCTTTGATTAAAACCCT  182

Query  372  ATACGTAGATCGGAATAAATTAACTCTAACGGAAACGGATGTGGAAATTCTCGCTTCTCT  431
             ||| | ||||  || ||  | ||  | || ||||||||||| |||||||| ||||||||
Sbjct  183  GTACTTGGATCAAAACAAGCTTACGTTGACCGAAACGGATGTTGAAATTCTAGCTTCTCT  242

Query  432  TTCCTCCTTGGAGGAATTGGATCTGAGTCTGAATTCGGGGATTAAGGCGCTCCCTTTTAA  491
            ||||   ||||| ||||||||||| | | |||| || || ||||| | ||| |||| |||
Sbjct  243  TTCCAGTTTGGAAGAATTGGATCTTAATTTGAACTCTGGAATTAAAGTGCTTCCTTCTAA  302

Query  492  TTACGAAAAACTTGTAAATCTCATTAATCTAAAAAGATTGAATATTaaaaaaaCTTCATT  551
             ||  | ||| |   |||||| | | || |||||||||||||||||||||||||||||||
Sbjct  303  CTATAATAAATTGAAAAATCTGACTCATTTAAAAAGATTGAATATTAAAAAAACTTCATT  362

Query  552  AAAGGGAGAGGATGCAGATAAATTGCAGGCGATTCTTCCAAATAC  596
            |||||| || ||||| |||||||| || ||  |||| ||||||||
Sbjct  363  AAAGGGGGAAGATGCTGATAAATTACAAGCAGTTCTCCCAAATAC  407
```

>Leptospira interrogans serovar Hebdomadis leucine-rich repeat protein gene, partial cds
Sequence ID: JX426072.1 Length: 303
Range 1: 12 to 299

Score:259 bits(286), Expect:1e-67,
Identities:230/288(80%), Gaps:0/288(0%), Strand: Plus/Plus

```
Query  309  GGGAAATAACGATTTTACGATTCTACCCTCCGAACTTTTGTTTCTTCCGTTGATCAAAAT  368
            ||| ||||||||||||||  ||||||| || |||||||||||||| || ||||| ||||
Sbjct  12   GGGTAATAACGATTTTACTTTTCTACCTTCGGAACTTTTGTTTCTGCCTTTGATTAAAAC  71

Query  369  TCTATACGTAGATCGGAATAAATTAACTCTAACGGAAACGGATGTGGAAATTCTCGCTTC  428
             || ||| | ||||  || ||  | ||  | || ||||||||||| |||||||| |||||
Sbjct  72   CCTGTACTTGGATCAAAACAAGCTTACGTTGACCGAAACGGATGTTGAAATTCTAGCTTC  131

Query  429  TCTTTCCTCCTTGGAGGAATTGGATCTGAGTCTGAATTCGGGGATTAAGGCGCTCCCTTT  488
            |||||||   ||||| ||||||||||| | | |||| || || ||||| | ||| ||||
Sbjct  132  TCTTTCCAGTTTGGAAGAATTGGATCTTAATTTGAACTCTGGAATTAAAGTGCTTCCTTC  191

Query  489  TAATTACGAAAAACTTGTAAATCTCATTAATCTAAAAAGATTGAATATTaaaaaaaCTTC  548
            ||| ||  | ||| |   |||||| | | || ||||||||||||||||||||||||||||
Sbjct  192  TAACTATAATAAATTGAAAAATCTGACTCATTTAAAAAGATTGAATATTAAAAAAACTTC  251

Query  549  ATTAAAGGGAGAGGATGCAGATAAATTGCAGGCGATTCTTCCAAATAC  596
            |||||||||||| ||||| |||||||| || ||  |||| ||||||||
Sbjct  252  ATTAAAGGGAGAAGATGCTGATAAATTACAAGCAGTTCTCCCAAATAC  299
```

>Leptospira interrogans serovar Pyrogenes leucine-rich repeat protein gene, partial cds
Sequence ID: JX426071.1 Length: 312
Range 1: 3 to 305

Score:214 bits(236), Expect:5e-54,
Identities:229/303(76%), Gaps:0/303(0%), Strand: Plus/Plus

```
Query  192  TCTTCCGGAATTTATCGGAGAATGTAAACGCCTTGAACAACTTAATCTTTTCGGGAACGA  251
            |||||| |  |  || ||||  ||||||   || |||||| |||| |||||||| || ||
Sbjct  3    TCTTCCTGGTTGGATTGGAGCTTGTAAAAATCTGGAACAAATTAACCTTTTCGGAAATGA  62

Query  252  CCTTACAACATTTCCGTCTACGTTTTCTAAATTAAAAAATCTGAAAGTGTTACTGGCGGG  311
             || |  ||  |||| ||| | |||||||||||||| ||| ||||||| |||||   |||
Sbjct  63   TCTAAATACGGTTCCATCTTCTTTTTCTAAATTAAAGAATTTGAAAGTATTACTCTTGGG  122

Query  312  AAATAACGATTTTACGATTCTACCCTCCGAACTTTTGTTTCTTCCGTTGATCAAAATTCT  371
             ||||||||||||||  ||||||| || |||||||||||||| || ||| | ||||  ||
Sbjct  123  TAATAACGATTTTACTTTTCTACCTTCGGAACTTTTGTTTCTGCCTTTGCTTAAAACCCT  182

Query  372  ATACGTAGATCGGAATAAATTAACTCTAACGGAAACGGATGTGGAAATTCTCGCTTCTCT  431
             ||  | ||||  || ||  | ||  | || ||||||||||| |||||||| ||||||||
Sbjct  183  GTATTTGGATCAAAACAAGCTTACGTTGACTGAAACGGATGTTGAAATTCTAGCTTCTCT  242

Query  432  TTCCTCCTTGGAGGAATTGGATCTGAGTCTGAATTCGGGGATTAAGGCGCTCCCTTTTAA  491
            ||||   ||||| ||||||||||| | | |||| || || || || | ||| |||| |||
Sbjct  243  TTCCAGTTTGGAAGAATTGGATCTTAATTTGAACTCTGGAATCAAAGTGCTTCCTTCTAA  302

Query  492  TTA  494
             ||
Sbjct  303  CTA  305
```
