# Supplementary material for: Leptospira borgpetersenii Leucine-Rich Repeat Proteins Provide Strong Protective Efficacy as Novel Leptospiral Vaccine Candidates
Source: Trop Med Infect Dis. 2022 Dec 22;8(1):6. doi: 10.3390/tropicalmed8010006 (PMC9863753; doi:10.3390/tropicalmed8010006)
Supplement: Supplementary file 1 [file tropicalmed-08-00006-s001.zip › Supplementary data S3.pdf]

Supplementary data S3. BLAST search of the *KU_R21_2271 gene* (NCBI accession: JX522460) yielded 98.04% to 99.84% identity to 36 sequences from *L. borgpetersenii* genomes.

```
RID: DW3W9HX8013
Job Title:JX522460:Leptospira borgpetersenii serovar...
Program: BLASTN
Query: Leptospira borgpetersenii serovar Sejroe leucine-rich repeat protein (LRR) gene, partial cds ID:
JX522460.1(nucleic acid) Length: 612
Database: nt Nucleotide collection (nt)
```

Sequences producing significant alignments:

Description	Scientific Name	Common Name	Taxid	Max Score	Total Score	Query cover	E Value	Per. Ident	Acc. Len	Accession
Leptospira borgpetersenii serovar Sejroe leucine-rich repeat...	Leptospira b...	NA	338220	1131	1131	100%	0.0	100.00	612	JX522460.1
Leptospira borgpetersenii strain R14-L chromosome 1	Leptospira b...	NA	174	1125	1125	100%	0.0	99.84	3581013	CP047516.1
Leptospira borgpetersenii strain R6L chromosome 1	Leptospira b...	NA	174	1125	1125	100%	0.0	99.84	3580531	CP047520.1
Leptospira borgpetersenii strain R14 chromosome 1	Leptospira b...	NA	174	1125	1125	100%	0.0	99.84	3601002	CP047504.1
Leptospira borgpetersenii strain R6 chromosome 1	Leptospira b...	NA	174	1125	1125	100%	0.0	99.84	3513910	CP047372.1
Leptospira borgpetersenii strain Mo4 chromosome 1	Leptospira b...	NA	174	1125	1125	100%	0.0	99.84	3581767	CP047334.1
Leptospira borgpetersenii strain R28 chromosome 1	Leptospira b...	NA	174	1125	1125	100%	0.0	99.84	3580481	CP047332.1
Leptospira borgpetersenii strain R23 chromosome 1	Leptospira b...	NA	174	1125	1125	100%	0.0	99.84	3579904	CP047370.1
Leptospira borgpetersenii strain R29 chromosome 1	Leptospira b...	NA	174	1125	1125	100%	0.0	99.84	3580756	CP047330.1
Leptospira borgpetersenii str. 4E chromosome 1	Leptospira b...	NA	1141102	1125	1125	100%	0.0	99.84	3550837	CP015814.2
Leptospira borgpetersenii serovar Ballum strain 56604 chromoso...	Leptospira b...	NA	280505	1125	1125	100%	0.0	99.84	3550837	CP012029.1
Leptospira borgpetersenii serovar Mini leucine-rich repeat...	Leptospira b...	NA	280507	1125	1125	100%	0.0	99.84	612	JX522459.1
Leptospira borgpetersenii serovar Ballum leucine-rich repeat...	Leptospira b...	NA	280505	1125	1125	100%	0.0	99.84	612	JX522458.1
Leptospira borgpetersenii strain 34-PK chromosome 1, complete...	Leptospira b...	NA	174	1120	1120	100%	0.0	99.67	3603305	CP097464.1
Leptospira borgpetersenii serovar Ceylonica strain Piyasena...	Leptospira b...	NA	508536	1114	1114	100%	0.0	99.51	3670231	CP026671.1

Leptospira borgpetersenii strain FMAS_AP2 chromosome 1, comple...	Leptospira b...	NA	174	1114	1114	100%	0.0	99.51	3595436	CP072630.1
Leptospira borgpetersenii strain FMAS_AP3 chromosome 1, comple...	Leptospira b...	NA	174	1114	1114	100%	0.0	99.51	3595898	CP072628.1
Leptospira borgpetersenii strain FMAS_AP4 chromosome 1, comple...	Leptospira b...	NA	174	1114	1114	100%	0.0	99.51	3595848	CP072626.1
Leptospira borgpetersenii strain FMAS_PN1 chromosome 1, comple...	Leptospira b...	NA	174	1114	1114	100%	0.0	99.51	3596207	CP072624.1
Leptospira borgpetersenii strain FMAS_PN4 chromosome 1, comple...	Leptospira b...	NA	174	1114	1114	100%	0.0	99.51	3595796	CP072622.1
Leptospira borgpetersenii strain FMAS_AP8 chromosome 1, comple...	Leptospira b...	NA	174	1114	1114	100%	0.0	99.51	3595870	CP072620.1
Leptospira borgpetersenii strain FMAS_AP9 chromosome 1, comple...	Leptospira b...	NA	174	1114	1114	100%	0.0	99.51	3595582	CP072618.1
Leptospira borgpetersenii serovar Javanica leucine-rich repeat...	Leptospira b...	NA	280504	1114	1114	100%	0.0	99.51	612	JX522457.1
Leptospira borgpetersenii serovar Hardjo strain NVSL S 818...	Leptospira b...	NA	328971	1070	1070	100%	0.0	98.20	3584464	CP015052.1
Leptospira borgpetersenii serovar Hardjo-bovis JB197 chromosom...	Leptospira b...	NA	355277	1070	1070	100%	0.0	98.20	3576473	CP000350.1
Leptospira borgpetersenii serovar Hardjo-bovis strain L49...	Leptospira b...	NA	338217	1064	1064	100%	0.0	98.04	3620710	CP033440.1
Leptospira borgpetersenii serovar Hardjo-bovis strain 203...	Leptospira b...	NA	338217	1064	1064	100%	0.0	98.04	3589981	CP021412.1
Leptospira borgpetersenii serovar Hardjo strain NVSL S 1343...	Leptospira b...	NA	328971	1064	1064	100%	0.0	98.04	3613654	CP015050.1
Leptospira borgpetersenii serovar Hardjo strain BK-30 chromoso...	Leptospira b...	NA	328971	1064	1064	100%	0.0	98.04	3629289	CP015048.1
Leptospira borgpetersenii serovar Hardjo strain BK-9 chromosome 1	Leptospira b...	NA	328971	1064	1064	100%	0.0	98.04	3631634	CP015046.1
Leptospira borgpetersenii serovar Hardjo strain BK-6 chromosome 1	Leptospira b...	NA	328971	1064	1064	100%	0.0	98.04	3649879	CP015044.1
Leptospira borgpetersenii strain KR39 chromosome 1, complete...	Leptospira b...	NA	174	1064	1064	100%	0.0	98.04	3603773	CP096127.1
Leptospira borgpetersenii serovar Hardjo strain TC129 chromoso...	Leptospira b...	NA	328971	1064	1064	100%	0.0	98.04	3585597	CP084038.1
Leptospira borgpetersenii strain TC147 chromosome I, complete...	Leptospira b...	NA	174	1064	1064	100%	0.0	98.04	3585599	CP084040.1
Leptospira borgpetersenii strain TC273 chromosome I, complete...	Leptospira b...	NA	174	1064	1064	100%	0.0	98.04	3585577	CP084042.1
Leptospira borgpetersenii strain TC112 chromosome I, complete...	Leptospira b...	NA	174	1064	1064	100%	0.0	98.04	3585599	CP084036.1
Leptospira borgpetersenii serovar Hardjo-bovis L550 chromosome...	Leptospira b...	NA	355276	1064	1064	100%	0.0	98.04	3614446	CP000348.1

Alignments:

>Leptospira borgpetersenii serovar Sejroe leucine-rich repeat protein (LRR) gene, partial cds
Sequence ID: JX522460.1 Length: 612
Range 1: 1 to 612

Score:1131 bits(612), Expect:0.0,
Identities:612/612(100%), Gaps:0/612(0%), Strand: Plus/Plus

```
Query  1    GGCTTCGTTTGTTTTACTGCGAGTTTCGACTGTAAAAAGAATGCGGAAGAGATTTTGGGA  60
            ||||||||||||||||||||||||||||||||||||||||||||||||||||||||||||
Sbjct  1    GGCTTCGTTTGTTTTACTGCGAGTTTCGACTGTAAAAAGAATGCGGAAGAGATTTTGGGA  60

Query  61   GAGGCAAAGGCAAAACCGGAATTGGTTCAAACTTTGGATTTTGGAATGCAGAAGTTGTCC  120
            ||||||||||||||||||||||||||||||||||||||||||||||||||||||||||||
Sbjct  61   GAGGCAAAGGCAAAACCGGAATTGGTTCAAACTTTGGATTTTGGAATGCAGAAGTTGTCC  120

Query  121  ACGGTTCCGGAAGAAGTTTGCGGTTTTCCGAATTTAACCAAGTTGGATCTTCGTTTAAAC  180
            ||||||||||||||||||||||||||||||||||||||||||||||||||||||||||||
Sbjct  121  ACGGTTCCGGAAGAAGTTTGCGGTTTTCCGAATTTAACCAAGTTGGATCTTCGTTTAAAC  180

Query  181  AGTTTGACTTTTCTTCCGGAATTTATCGGAGAATGTAAACGCCTTGAACAACTTAATCTT  240
            ||||||||||||||||||||||||||||||||||||||||||||||||||||||||||||
Sbjct  181  AGTTTGACTTTTCTTCCGGAATTTATCGGAGAATGTAAACGCCTTGAACAACTTAATCTT  240

Query  241  TTCGGGAACGACCTTACAACATTTCCGTCTACGTTTTCTAAATTAAAAAATCTGAAAGTG  300
            ||||||||||||||||||||||||||||||||||||||||||||||||||||||||||||
Sbjct  241  TTCGGGAACGACCTTACAACATTTCCGTCTACGTTTTCTAAATTAAAAAATCTGAAAGTG  300

Query  301  TTACTGGCGGGAAATAACGATTTTACGATTCTACCCTCCGAACTTTTGTTTCTTCCGTTG  360
            ||||||||||||||||||||||||||||||||||||||||||||||||||||||||||||
Sbjct  301  TTACTGGCGGGAAATAACGATTTTACGATTCTACCCTCCGAACTTTTGTTTCTTCCGTTG  360

Query  361  ATCAAAATTCTATACGTAGATCGGAATAAATTAACTCTAACGGAAACGGATGTGGAAATT  420
            ||||||||||||||||||||||||||||||||||||||||||||||||||||||||||||
Sbjct  361  ATCAAAATTCTATACGTAGATCGGAATAAATTAACTCTAACGGAAACGGATGTGGAAATT  420

Query  421  CTCGCTTCTCTTTCCTCCTTGGAGGAATTGGATCTGAGTCTGAATTCGGGGATTAAGGCG  480
            ||||||||||||||||||||||||||||||||||||||||||||||||||||||||||||
Sbjct  421  CTCGCTTCTCTTTCCTCCTTGGAGGAATTGGATCTGAGTCTGAATTCGGGGATTAAGGCG  480

Query  481  CTCCCTTTTAATTACGAAAAACTTGTAAATCTCATTAATCTAAAAAGATTGAATATTaaa  540
            ||||||||||||||||||||||||||||||||||||||||||||||||||||||||||||
Sbjct  481  CTCCCTTTTAATTACGAAAAACTTGTAAATCTCATTAATCTAAAAAGATTGAATATTAAA  540

Query  541  aaaaCTTCATTAAAGGGAGAGGATGCAGATAAATTGCAGGCGATTCTTCCAAATACCAAA  600
            ||||||||||||||||||||||||||||||||||||||||||||||||||||||||||||
Sbjct  541  AAAACTTCATTAAAGGGAGAGGATGCAGATAAATTGCAGGCGATTCTTCCAAATACCAAA  600

Query  601  ATCGACTACTGA  612
            ||||||||||||
Sbjct  601  ATCGACTACTGA  612
```

>Leptospira borgpetersenii strain R14-L chromosome 1
Sequence ID: CP047516.1 Length: 3581013
Range 1: 2585893 to 2586504

Score:1125 bits(609), Expect:0.0,
Identities:611/612(99%), Gaps:0/612(0%), Strand: Plus/Plus

```
Query  1        GGCTTCGTTTGTTTTACTGCGAGTTTCGACTGTAAAAAGAATGCGGAAGAGATTTTGGGA  60
                ||||||||||||||||||||||||||||||||||||||||||||||||||||||||||||
Sbjct  2585893  GGCTTCGTTTGTTTTACTGCGAGTTTCGACTGTAAAAAGAATGCGGAAGAGATTTTGGGA  2585952

Query  61       GAGGCAAAGGCAAAACCGGAATTGGTTCAAACTTTGGATTTTGGAATGCAGAAGTTGTCC  120
                ||||||||||||||||||||||||||||||||||||||||||||||||||||||||||||
Sbjct  2585953  GAGGCAAAGGCAAAACCGGAATTGGTTCAAACTTTGGATTTTGGAATGCAGAAGTTGTCC  2586012

Query  121      ACGGTTCCGGAAGAAGTTTGCGGTTTTCCGAATTTAACCAAGTTGGATCTTCGTTTAAAC  180
                ||||||||||||||||||||||||||||||||||||||||||||||||||||||||||||
Sbjct  2586013  ACGGTTCCGGAAGAAGTTTGCGGTTTTCCGAATTTAACCAAGTTGGATCTTCGTTTAAAC  2586072

Query  181      AGTTTGACTTTTCTTCCGGAATTTATCGGAGAATGTAAACGCCTTGAACAACTTAATCTT  240
                ||||||||||||||||||||||||||||||||||||||||||||||||||||||||||||
Sbjct  2586073  AGTTTGACTTTTCTTCCGGAATTTATCGGAGAATGTAAACGCCTTGAACAACTTAATCTT  2586132

Query  241      TTCGGGAACGACCTTACAACATTTCCGTCTACGTTTTCTAAATTAAAAAATCTGAAAGTG  300
                ||||||||||||||||||||||||||||||||||||||||||||||||||||||||||||
Sbjct  2586133  TTCGGGAACGACCTTACAACATTTCCGTCTACGTTTTCTAAATTAAAAAATCTGAAAGTG  2586192

Query  301      TTACTGGCGGGAAATAACGATTTTACGATTCTACCCTCCGAACTTTTGTTTCTTCCGTTG  360
                ||||||||||||||||||||||||||||||||||||||||||||||||||||||||||||
Sbjct  2586193  TTACTGGCGGGAAATAACGATTTTACGATTCTACCCTCCGAACTTTTGTTTCTTCCGTTG  2586252

Query  361      ATCAAAATTCTATACGTAGATCGGAATAAATTAACTCTAACGGAAACGGATGTGGAAATT  420
                ||||||||||||||||||||||||||||||||||||||||||||||||||||||||||||
Sbjct  2586253  ATCAAAATTCTATACGTAGATCGGAATAAATTAACTCTAACGGAAACGGATGTGGAAATT  2586312

Query  421      CTCGCTTCTCTTTCCTCCTTGGAGGAATTGGATCTGAGTCTGAATTCGGGGATTAAGGCG  480
                ||||||||||||||||||||||||||||||||||||||||||||||||||||||||||||
Sbjct  2586313  CTCGCTTCTCTTTCCTCCTTGGAGGAATTGGATCTGAGTCTGAATTCGGGGATTAAGGCG  2586372

Query  481      CTCCCTTTTAATTACGAAAAACTTGTAAATCTCATTAATCTAAAAAGATTGAATATTaaa  540
                ||||||||||||||||||||||||||||||||||||||||||||||||||||||||||||
Sbjct  2586373  CTCCCTTTTAATTACGAAAAACTTGTAAATCTCATTAATCTAAAAAGATTGAATATTAAA  2586432

Query  541      aaaaCTTCATTAAAGGGAGAGGATGCAGATAAATTGCAGGCGATTCTTCCAAATACCAAA  600
                ||||||||||||||||||||||||||||||||||||||||||||||||||||| ||||||
Sbjct  2586433  AAAACTTCATTAAAGGGAGAGGATGCAGATAAATTGCAGGCGATTCTTCCAAACACCAAA  2586492

Query  601      ATCGACTACTGA  612
                ||||||||||||
Sbjct  2586493  ATCGACTACTGA  2586504
```

>Leptospira borgpetersenii strain R6L chromosome 1
Sequence ID: CP047520.1 Length: 3580531
Range 1: 2527014 to 2527625

Score:1125 bits(609), Expect:0.0,
Identities:611/612(99%), Gaps:0/612(0%), Strand: Plus/Plus

```
Query  1        GGCTTCGTTTGTTTTACTGCGAGTTTCGACTGTAAAAAGAATGCGGAAGAGATTTTGGGA  60
                ||||||||||||||||||||||||||||||||||||||||||||||||||||||||||||
Sbjct  2527014  GGCTTCGTTTGTTTTACTGCGAGTTTCGACTGTAAAAAGAATGCGGAAGAGATTTTGGGA  2527073

Query  61       GAGGCAAAGGCAAAACCGGAATTGGTTCAAACTTTGGATTTTGGAATGCAGAAGTTGTCC  120
                ||||||||||||||||||||||||||||||||||||||||||||||||||||||||||||
Sbjct  2527074  GAGGCAAAGGCAAAACCGGAATTGGTTCAAACTTTGGATTTTGGAATGCAGAAGTTGTCC  2527133
```

```
Query  121      ACGGTTCCGGAAGAAGTTTGCGGTTTTCCGAATTTAACCAAGTTGGATCTTCGTTTAAAC  180
                ||||||||||||||||||||||||||||||||||||||||||||||||||||||||||||
Sbjct  2527134  ACGGTTCCGGAAGAAGTTTGCGGTTTTCCGAATTTAACCAAGTTGGATCTTCGTTTAAAC  2527193

Query  181      AGTTTGACTTTTCTTCCGGAATTTATCGGAGAATGTAAACGCCTTGAACAACTTAATCTT  240
                ||||||||||||||||||||||||||||||||||||||||||||||||||||||||||||
Sbjct  2527194  AGTTTGACTTTTCTTCCGGAATTTATCGGAGAATGTAAACGCCTTGAACAACTTAATCTT  2527253

Query  241      TTCGGGAACGACCTTACAACATTTCCGTCTACGTTTTCTAAATTAAAAAATCTGAAAGTG  300
                ||||||||||||||||||||||||||||||||||||||||||||||||||||||||||||
Sbjct  2527254  TTCGGGAACGACCTTACAACATTTCCGTCTACGTTTTCTAAATTAAAAAATCTGAAAGTG  2527313

Query  301      TTACTGGCGGGAAATAACGATTTTACGATTCTACCCTCCGAACTTTTGTTTCTTCCGTTG  360
                ||||||||||||||||||||||||||||||||||||||||||||||||||||||||||||
Sbjct  2527314  TTACTGGCGGGAAATAACGATTTTACGATTCTACCCTCCGAACTTTTGTTTCTTCCGTTG  2527373

Query  361      ATCAAAATTCTATACGTAGATCGGAATAAATTAACTCTAACGGAAACGGATGTGGAAATT  420
                ||||||||||||||||||||||||||||||||||||||||||||||||||||||||||||
Sbjct  2527374  ATCAAAATTCTATACGTAGATCGGAATAAATTAACTCTAACGGAAACGGATGTGGAAATT  2527433

Query  421      CTCGCTTCTCTTTCCTCCTTGGAGGAATTGGATCTGAGTCTGAATTCGGGGATTAAGGCG  480
                ||||||||||||||||||||||||||||||||||||||||||||||||||||||||||||
Sbjct  2527434  CTCGCTTCTCTTTCCTCCTTGGAGGAATTGGATCTGAGTCTGAATTCGGGGATTAAGGCG  2527493

Query  481      CTCCCTTTTAATTACGAAAAACTTGTAAATCTCATTAATCTAAAAAGATTGAATATTaaa  540
                ||||||||||||||||||||||||||||||||||||||||||||||||||||||||||||
Sbjct  2527494  CTCCCTTTTAATTACGAAAAACTTGTAAATCTCATTAATCTAAAAAGATTGAATATTAAA  2527553

Query  541      aaaaCTTCATTAAAGGGAGAGGATGCAGATAAATTGCAGGCGATTCTTCCAAATACCAAA  600
                ||||||||||||||||||||||||||||||||||||||||||||||||||||| ||||||
Sbjct  2527554  AAAACTTCATTAAAGGGAGAGGATGCAGATAAATTGCAGGCGATTCTTCCAAACACCAAA  2527613

Query  601      ATCGACTACTGA  612
                ||||||||||||
Sbjct  2527614  ATCGACTACTGA  2527625
```

>Leptospira borgpetersenii strain R14 chromosome 1
Sequence ID: CP047504.1 Length: 3601002
Range 1: 2545970 to 2546581

Score:1125 bits(609), Expect:0.0,
Identities:611/612(99%), Gaps:0/612(0%), Strand: Plus/Plus

```
Query  1        GGCTTCGTTTGTTTTACTGCGAGTTTCGACTGTAAAAAGAATGCGGAAGAGATTTTGGGA  60
                ||||||||||||||||||||||||||||||||||||||||||||||||||||||||||||
Sbjct  2545970  GGCTTCGTTTGTTTTACTGCGAGTTTCGACTGTAAAAAGAATGCGGAAGAGATTTTGGGA  2546029

Query  61       GAGGCAAAGGCAAAACCGGAATTGGTTCAAACTTTGGATTTTGGAATGCAGAAGTTGTCC  120
                ||||||||||||||||||||||||||||||||||||||||||||||||||||||||||||
Sbjct  2546030  GAGGCAAAGGCAAAACCGGAATTGGTTCAAACTTTGGATTTTGGAATGCAGAAGTTGTCC  2546089

Query  121      ACGGTTCCGGAAGAAGTTTGCGGTTTTCCGAATTTAACCAAGTTGGATCTTCGTTTAAAC  180
                ||||||||||||||||||||||||||||||||||||||||||||||||||||||||||||
Sbjct  2546090  ACGGTTCCGGAAGAAGTTTGCGGTTTTCCGAATTTAACCAAGTTGGATCTTCGTTTAAAC  2546149

Query  181      AGTTTGACTTTTCTTCCGGAATTTATCGGAGAATGTAAACGCCTTGAACAACTTAATCTT  240
                ||||||||||||||||||||||||||||||||||||||||||||||||||||||||||||
Sbjct  2546150  AGTTTGACTTTTCTTCCGGAATTTATCGGAGAATGTAAACGCCTTGAACAACTTAATCTT  2546209

Query  241      TTCGGGAACGACCTTACAACATTTCCGTCTACGTTTTCTAAATTAAAAAATCTGAAAGTG  300
```

```
                  ||||||||||||||||||||||||||||||||||||||||||||||||||||||||||||
Sbjct  2546210  TTCGGGAACGACCTTACAACATTTCCGTCTACGTTTTCTAAATTAAAAAATCTGAAAGTG  2546269

Query  301      TTACTGGCGGGAAATAACGATTTTACGATTCTACCCTCCGAACTTTTGTTTCTTCCGTTG  360
                ||||||||||||||||||||||||||||||||||||||||||||||||||||||||||||
Sbjct  2546270  TTACTGGCGGGAAATAACGATTTTACGATTCTACCCTCCGAACTTTTGTTTCTTCCGTTG  2546329

Query  361      ATCAAAATTCTATACGTAGATCGGAATAAATTAACTCTAACGGAAACGGATGTGGAAATT  420
                ||||||||||||||||||||||||||||||||||||||||||||||||||||||||||||
Sbjct  2546330  ATCAAAATTCTATACGTAGATCGGAATAAATTAACTCTAACGGAAACGGATGTGGAAATT  2546389

Query  421      CTCGCTTCTCTTTCCTCCTTGGAGGAATTGGATCTGAGTCTGAATTCGGGGATTAAGGCG  480
                ||||||||||||||||||||||||||||||||||||||||||||||||||||||||||||
Sbjct  2546390  CTCGCTTCTCTTTCCTCCTTGGAGGAATTGGATCTGAGTCTGAATTCGGGGATTAAGGCG  2546449

Query  481      CTCCCTTTTAATTACGAAAAACTTGTAAATCTCATTAATCTAAAAAGATTGAATATTaaa  540
                ||||||||||||||||||||||||||||||||||||||||||||||||||||||||||||
Sbjct  2546450  CTCCCTTTTAATTACGAAAAACTTGTAAATCTCATTAATCTAAAAAGATTGAATATTAAA  2546509

Query  541      aaaaCTTCATTAAAGGGAGAGGATGCAGATAAATTGCAGGCGATTCTTCCAAATACCAAA  600
                |||||||||||||||||||||||||||||||||||||||||||||||||||| ||||||
Sbjct  2546510  AAAACTTCATTAAAGGGAGAGGATGCAGATAAATTGCAGGCGATTCTTCCAAACACCAAA  2546569

Query  601      ATCGACTACTGA  612
                ||||||||||||
Sbjct  2546570  ATCGACTACTGA  2546581
```

>Leptospira borgpetersenii strain R6 chromosome 1
Sequence ID: CP047372.1 Length: 3513910
Range 1: 2460661 to 2461272

Score:1125 bits(609), Expect:0.0,
Identities:611/612(99%), Gaps:0/612(0%), Strand: Plus/Plus

```
Query  1        GGCTTCGTTTGTTTTACTGCGAGTTTCGACTGTAAAAAGAATGCGGAAGAGATTTTGGGA  60
                ||||||||||||||||||||||||||||||||||||||||||||||||||||||||||||
Sbjct  2460661  GGCTTCGTTTGTTTTACTGCGAGTTTCGACTGTAAAAAGAATGCGGAAGAGATTTTGGGA  2460720

Query  61       GAGGCAAAGGCAAAACCGGAATTGGTTCAAACTTTGGATTTTGGAATGCAGAAGTTGTCC  120
                ||||||||||||||||||||||||||||||||||||||||||||||||||||||||||||
Sbjct  2460721  GAGGCAAAGGCAAAACCGGAATTGGTTCAAACTTTGGATTTTGGAATGCAGAAGTTGTCC  2460780

Query  121      ACGGTTCCGGAAGAAGTTTGCGGTTTTCCGAATTTAACCAAGTTGGATCTTCGTTTAAAC  180
                ||||||||||||||||||||||||||||||||||||||||||||||||||||||||||||
Sbjct  2460781  ACGGTTCCGGAAGAAGTTTGCGGTTTTCCGAATTTAACCAAGTTGGATCTTCGTTTAAAC  2460840

Query  181      AGTTTGACTTTTCTTCCGGAATTTATCGGAGAATGTAAACGCCTTGAACAACTTAATCTT  240
                ||||||||||||||||||||||||||||||||||||||||||||||||||||||||||||
Sbjct  2460841  AGTTTGACTTTTCTTCCGGAATTTATCGGAGAATGTAAACGCCTTGAACAACTTAATCTT  2460900

Query  241      TTCGGGAACGACCTTACAACATTTCCGTCTACGTTTTCTAAATTAAAAAATCTGAAAGTG  300
                ||||||||||||||||||||||||||||||||||||||||||||||||||||||||||||
Sbjct  2460901  TTCGGGAACGACCTTACAACATTTCCGTCTACGTTTTCTAAATTAAAAAATCTGAAAGTG  2460960

Query  301      TTACTGGCGGGAAATAACGATTTTACGATTCTACCCTCCGAACTTTTGTTTCTTCCGTTG  360
                ||||||||||||||||||||||||||||||||||||||||||||||||||||||||||||
Sbjct  2460961  TTACTGGCGGGAAATAACGATTTTACGATTCTACCCTCCGAACTTTTGTTTCTTCCGTTG  2461020

Query  361      ATCAAAATTCTATACGTAGATCGGAATAAATTAACTCTAACGGAAACGGATGTGGAAATT  420
                ||||||||||||||||||||||||||||||||||||||||||||||||||||||||||||
```

```
Sbjct  2461021  ATCAAAATTCTATACGTAGATCGGAATAAATTAACTCTAACGGAAACGGATGTGGAAATT  2461080

Query  421      CTCGCTTCTCTTTCCTCCTTGGAGGAATTGGATCTGAGTCTGAATTCGGGGATTAAGGCG  480
                ||||||||||||||||||||||||||||||||||||||||||||||||||||||||||||
Sbjct  2461081  CTCGCTTCTCTTTCCTCCTTGGAGGAATTGGATCTGAGTCTGAATTCGGGGATTAAGGCG  2461140

Query  481      CTCCCTTTTAATTACGAAAAACTTGTAAATCTCATTAATCTAAAAAGATTGAATATTaaa  540
                ||||||||||||||||||||||||||||||||||||||||||||||||||||||||||||
Sbjct  2461141  CTCCCTTTTAATTACGAAAAACTTGTAAATCTCATTAATCTAAAAAGATTGAATATTAAA  2461200

Query  541      aaaaCTTCATTAAAGGGAGAGGATGCAGATAAATTGCAGGCGATTCTTCCAAATACCAAA  600
                ||||||||||||||||||||||||||||||||||||||||||||||||||||| ||||||
Sbjct  2461201  AAAACTTCATTAAAGGGAGAGGATGCAGATAAATTGCAGGCGATTCTTCCAAACACCAAA  2461260

Query  601      ATCGACTACTGA  612
                ||||||||||||
Sbjct  2461261  ATCGACTACTGA  2461272




>Leptospira borgpetersenii strain Mo4 chromosome 1
Sequence ID: CP047334.1 Length: 3581767
Range 1: 2585809 to 2586420

Score:1125 bits(609), Expect:0.0,
Identities:611/612(99%),  Gaps:0/612(0%), Strand: Plus/Plus

Query  1        GGCTTCGTTTGTTTTACTGCGAGTTTCGACTGTAAAAAGAATGCGGAAGAGATTTTGGGA  60
                ||||||||||||||||||||||||||||||||||||||||||||||||||||||||||||
Sbjct  2585809  GGCTTCGTTTGTTTTACTGCGAGTTTCGACTGTAAAAAGAATGCGGAAGAGATTTTGGGA  2585868

Query  61       GAGGCAAAGGCAAAACCGGAATTGGTTCAAACTTTGGATTTTGGAATGCAGAAGTTGTCC  120
                ||||||||||||||||||||||||||||||||||||||||||||||||||||||||||||
Sbjct  2585869  GAGGCAAAGGCAAAACCGGAATTGGTTCAAACTTTGGATTTTGGAATGCAGAAGTTGTCC  2585928

Query  121      ACGGTTCCGGAAGAAGTTTGCGGTTTTCCGAATTTAACCAAGTTGGATCTTCGTTTAAAC  180
                ||||||||||||||||||||||||||||||||||||||||||||||||||||||||||||
Sbjct  2585929  ACGGTTCCGGAAGAAGTTTGCGGTTTTCCGAATTTAACCAAGTTGGATCTTCGTTTAAAC  2585988

Query  181      AGTTTGACTTTTCTTCCGGAATTTATCGGAGAATGTAAACGCCTTGAACAACTTAATCTT  240
                ||||||||||||||||||||||||||||||||||||||||||||||||||||||||||||
Sbjct  2585989  AGTTTGACTTTTCTTCCGGAATTTATCGGAGAATGTAAACGCCTTGAACAACTTAATCTT  2586048

Query  241      TTCGGGAACGACCTTACAACATTTCCGTCTACGTTTTCTAAATTAAAAAATCTGAAAGTG  300
                ||||||||||||||||||||||||||||||||||||||||||||||||||||||||||||
Sbjct  2586049  TTCGGGAACGACCTTACAACATTTCCGTCTACGTTTTCTAAATTAAAAAATCTGAAAGTG  2586108

Query  301      TTACTGGCGGGAAATAACGATTTTACGATTCTACCCTCCGAACTTTTGTTTCTTCCGTTG  360
                ||||||||||||||||||||||||||||||||||||||||||||||||||||||||||||
Sbjct  2586109  TTACTGGCGGGAAATAACGATTTTACGATTCTACCCTCCGAACTTTTGTTTCTTCCGTTG  2586168

Query  361      ATCAAAATTCTATACGTAGATCGGAATAAATTAACTCTAACGGAAACGGATGTGGAAATT  420
                ||||||||||||||||||||||||||||||||||||||||||||||||||||||||||||
Sbjct  2586169  ATCAAAATTCTATACGTAGATCGGAATAAATTAACTCTAACGGAAACGGATGTGGAAATT  2586228

Query  421      CTCGCTTCTCTTTCCTCCTTGGAGGAATTGGATCTGAGTCTGAATTCGGGGATTAAGGCG  480
                ||||||||||||||||||||||||||||||||||||||||||||||||||||||||||||
Sbjct  2586229  CTCGCTTCTCTTTCCTCCTTGGAGGAATTGGATCTGAGTCTGAATTCGGGGATTAAGGCG  2586288

Query  481      CTCCCTTTTAATTACGAAAAACTTGTAAATCTCATTAATCTAAAAAGATTGAATATTaaa  540
                ||||||||||||||||||||||||||||||||||||||||||||||||||||||||||||
Sbjct  2586289  CTCCCTTTTAATTACGAAAAACTTGTAAATCTCATTAATCTAAAAAGATTGAATATTAAA  2586348
```

```
Query  541      aaaaCTTCATTAAAGGGAGAGGATGCAGATAAATTGCAGGCGATTCTTCCAAATACCAAA  600
                |||||||||||||||||||||||||||||||||||||||||||||||||||| ||||||
Sbjct  2586349  AAAACTTCATTAAAGGGAGAGGATGCAGATAAATTGCAGGCGATTCTTCCAAACACCAAA  2586408

Query  601      ATCGACTACTGA  612
                ||||||||||||
Sbjct  2586409  ATCGACTACTGA  2586420
```

>Leptospira borgpetersenii strain R28 chromosome 1
Sequence ID: CP047332.1 Length: 3580481
Range 1: 2527184 to 2527795

Score:1125 bits(609), Expect:0.0,
Identities:611/612(99%), Gaps:0/612(0%), Strand: Plus/Plus

```
Query  1        GGCTTCGTTTGTTTTACTGCGAGTTTCGACTGTAAAAAGAATGCGGAAGAGATTTTGGGA  60
                ||||||||||||||||||||||||||||||||||||||||||||||||||||||||||||
Sbjct  2527184  GGCTTCGTTTGTTTTACTGCGAGTTTCGACTGTAAAAAGAATGCGGAAGAGATTTTGGGA  2527243

Query  61       GAGGCAAAGGCAAAACCGGAATTGGTTCAAACTTTGGATTTTGGAATGCAGAAGTTGTCC  120
                ||||||||||||||||||||||||||||||||||||||||||||||||||||||||||||
Sbjct  2527244  GAGGCAAAGGCAAAACCGGAATTGGTTCAAACTTTGGATTTTGGAATGCAGAAGTTGTCC  2527303

Query  121      ACGGTTCCGGAAGAAGTTTGCGGTTTTCCGAATTTAACCAAGTTGGATCTTCGTTTAAAC  180
                ||||||||||||||||||||||||||||||||||||||||||||||||||||||||||||
Sbjct  2527304  ACGGTTCCGGAAGAAGTTTGCGGTTTTCCGAATTTAACCAAGTTGGATCTTCGTTTAAAC  2527363

Query  181      AGTTTGACTTTTCTTCCGGAATTTATCGGAGAATGTAAACGCCTTGAACAACTTAATCTT  240
                ||||||||||||||||||||||||||||||||||||||||||||||||||||||||||||
Sbjct  2527364  AGTTTGACTTTTCTTCCGGAATTTATCGGAGAATGTAAACGCCTTGAACAACTTAATCTT  2527423

Query  241      TTCGGGAACGACCTTACAACATTTCCGTCTACGTTTTCTAAATTAAAAAATCTGAAAGTG  300
                ||||||||||||||||||||||||||||||||||||||||||||||||||||||||||||
Sbjct  2527424  TTCGGGAACGACCTTACAACATTTCCGTCTACGTTTTCTAAATTAAAAAATCTGAAAGTG  2527483

Query  301      TTACTGGCGGGAAATAACGATTTTACGATTCTACCCTCCGAACTTTTGTTTCTTCCGTTG  360
                ||||||||||||||||||||||||||||||||||||||||||||||||||||||||||||
Sbjct  2527484  TTACTGGCGGGAAATAACGATTTTACGATTCTACCCTCCGAACTTTTGTTTCTTCCGTTG  2527543

Query  361      ATCAAAATTCTATACGTAGATCGGAATAAATTAACTCTAACGGAAACGGATGTGGAAATT  420
                ||||||||||||||||||||||||||||||||||||||||||||||||||||||||||||
Sbjct  2527544  ATCAAAATTCTATACGTAGATCGGAATAAATTAACTCTAACGGAAACGGATGTGGAAATT  2527603

Query  421      CTCGCTTCTCTTTCCTCCTTGGAGGAATTGGATCTGAGTCTGAATTCGGGGATTAAGGCG  480
                ||||||||||||||||||||||||||||||||||||||||||||||||||||||||||||
Sbjct  2527604  CTCGCTTCTCTTTCCTCCTTGGAGGAATTGGATCTGAGTCTGAATTCGGGGATTAAGGCG  2527663

Query  481      CTCCCTTTTAATTACGAAAAACTTGTAAATCTCATTAATCTAAAAAGATTGAATATTaaa  540
                ||||||||||||||||||||||||||||||||||||||||||||||||||||||||||||
Sbjct  2527664  CTCCCTTTTAATTACGAAAAACTTGTAAATCTCATTAATCTAAAAAGATTGAATATTAAA  2527723

Query  541      aaaaCTTCATTAAAGGGAGAGGATGCAGATAAATTGCAGGCGATTCTTCCAAATACCAAA  600
                |||||||||||||||||||||||||||||||||||||||||||||||||||| ||||||
Sbjct  2527724  AAAACTTCATTAAAGGGAGAGGATGCAGATAAATTGCAGGCGATTCTTCCAAACACCAAA  2527783

Query  601      ATCGACTACTGA  612
                ||||||||||||
Sbjct  2527784  ATCGACTACTGA  2527795
```

```
>Leptospira borgpetersenii strain R23 chromosome 1
Sequence ID: CP047370.1 Length: 3579904
Range 1: 2527240 to 2527851

Score:1125 bits(609), Expect:0.0,
Identities:611/612(99%),  Gaps:0/612(0%), Strand: Plus/Plus

Query  1        GGCTTCGTTTGTTTTACTGCGAGTTTCGACTGTAAAAAGAATGCGGAAGAGATTTTGGGA  60
                ||||||||||||||||||||||||||||||||||||||||||||||||||||||||||||
Sbjct  2527240  GGCTTCGTTTGTTTTACTGCGAGTTTCGACTGTAAAAAGAATGCGGAAGAGATTTTGGGA  2527299

Query  61       GAGGCAAAGGCAAAACCGGAATTGGTTCAAACTTTGGATTTTGGAATGCAGAAGTTGTCC  120
                ||||||||||||||||||||||||||||||||||||||||||||||||||||||||||||
Sbjct  2527300  GAGGCAAAGGCAAAACCGGAATTGGTTCAAACTTTGGATTTTGGAATGCAGAAGTTGTCC  2527359

Query  121      ACGGTTCCGGAAGAAGTTTGCGGTTTTCCGAATTTAACCAAGTTGGATCTTCGTTTAAAC  180
                ||||||||||||||||||||||||||||||||||||||||||||||||||||||||||||
Sbjct  2527360  ACGGTTCCGGAAGAAGTTTGCGGTTTTCCGAATTTAACCAAGTTGGATCTTCGTTTAAAC  2527419

Query  181      AGTTTGACTTTTCTTCCGGAATTTATCGGAGAATGTAAACGCCTTGAACAACTTAATCTT  240
                ||||||||||||||||||||||||||||||||||||||||||||||||||||||||||||
Sbjct  2527420  AGTTTGACTTTTCTTCCGGAATTTATCGGAGAATGTAAACGCCTTGAACAACTTAATCTT  2527479

Query  241      TTCGGGAACGACCTTACAACATTTCCGTCTACGTTTTCTAAATTAAAAAATCTGAAAGTG  300
                ||||||||||||||||||||||||||||||||||||||||||||||||||||||||||||
Sbjct  2527480  TTCGGGAACGACCTTACAACATTTCCGTCTACGTTTTCTAAATTAAAAAATCTGAAAGTG  2527539

Query  301      TTACTGGCGGGAAATAACGATTTTACGATTCTACCCTCCGAACTTTTGTTTCTTCCGTTG  360
                ||||||||||||||||||||||||||||||||||||||||||||||||||||||||||||
Sbjct  2527540  TTACTGGCGGGAAATAACGATTTTACGATTCTACCCTCCGAACTTTTGTTTCTTCCGTTG  2527599

Query  361      ATCAAAATTCTATACGTAGATCGGAATAAATTAACTCTAACGGAAACGGATGTGGAAATT  420
                ||||||||||||||||||||||||||||||||||||||||||||||||||||||||||||
Sbjct  2527600  ATCAAAATTCTATACGTAGATCGGAATAAATTAACTCTAACGGAAACGGATGTGGAAATT  2527659

Query  421      CTCGCTTCTCTTTCCTCCTTGGAGGAATTGGATCTGAGTCTGAATTCGGGGATTAAGGCG  480
                ||||||||||||||||||||||||||||||||||||||||||||||||||||||||||||
Sbjct  2527660  CTCGCTTCTCTTTCCTCCTTGGAGGAATTGGATCTGAGTCTGAATTCGGGGATTAAGGCG  2527719

Query  481      CTCCCTTTTAATTACGAAAAACTTGTAAATCTCATTAATCTAAAAAGATTGAATATTaaa  540
                ||||||||||||||||||||||||||||||||||||||||||||||||||||||||||||
Sbjct  2527720  CTCCCTTTTAATTACGAAAAACTTGTAAATCTCATTAATCTAAAAAGATTGAATATTAAA  2527779

Query  541      aaaaCTTCATTAAAGGGAGAGGATGCAGATAAATTGCAGGCGATTCTTCCAAATACCAAA  600
                ||||||||||||||||||||||||||||||||||||||||||||||||||||| ||||||
Sbjct  2527780  AAAACTTCATTAAAGGGAGAGGATGCAGATAAATTGCAGGCGATTCTTCCAAACACCAAA  2527839

Query  601      ATCGACTACTGA  612
                ||||||||||||
Sbjct  2527840  ATCGACTACTGA  2527851




>Leptospira borgpetersenii strain R29 chromosome 1
Sequence ID: CP047330.1 Length: 3580756
Range 1: 2527211 to 2527822

Score:1125 bits(609), Expect:0.0,
Identities:611/612(99%),  Gaps:0/612(0%), Strand: Plus/Plus
```

```
Query  1        GGCTTCGTTTGTTTTACTGCGAGTTTCGACTGTAAAAAGAATGCGGAAGAGATTTTGGGA  60
                ||||||||||||||||||||||||||||||||||||||||||||||||||||||||||||
Sbjct  2527211  GGCTTCGTTTGTTTTACTGCGAGTTTCGACTGTAAAAAGAATGCGGAAGAGATTTTGGGA  2527270

Query  61       GAGGCAAAGGCAAAACCGGAATTGGTTCAAACTTTGGATTTTGGAATGCAGAAGTTGTCC  120
                ||||||||||||||||||||||||||||||||||||||||||||||||||||||||||||
Sbjct  2527271  GAGGCAAAGGCAAAACCGGAATTGGTTCAAACTTTGGATTTTGGAATGCAGAAGTTGTCC  2527330

Query  121      ACGGTTCCGGAAGAAGTTTGCGGTTTTCCGAATTTAACCAAGTTGGATCTTCGTTTAAAC  180
                ||||||||||||||||||||||||||||||||||||||||||||||||||||||||||||
Sbjct  2527331  ACGGTTCCGGAAGAAGTTTGCGGTTTTCCGAATTTAACCAAGTTGGATCTTCGTTTAAAC  2527390

Query  181      AGTTTGACTTTTCTTCCGGAATTTATCGGAGAATGTAAACGCCTTGAACAACTTAATCTT  240
                ||||||||||||||||||||||||||||||||||||||||||||||||||||||||||||
Sbjct  2527391  AGTTTGACTTTTCTTCCGGAATTTATCGGAGAATGTAAACGCCTTGAACAACTTAATCTT  2527450

Query  241      TTCGGGAACGACCTTACAACATTTCCGTCTACGTTTTCTAAATTAAAAAATCTGAAAGTG  300
                ||||||||||||||||||||||||||||||||||||||||||||||||||||||||||||
Sbjct  2527451  TTCGGGAACGACCTTACAACATTTCCGTCTACGTTTTCTAAATTAAAAAATCTGAAAGTG  2527510

Query  301      TTACTGGCGGGAAATAACGATTTTACGATTCTACCCTCCGAACTTTTGTTTCTTCCGTTG  360
                ||||||||||||||||||||||||||||||||||||||||||||||||||||||||||||
Sbjct  2527511  TTACTGGCGGGAAATAACGATTTTACGATTCTACCCTCCGAACTTTTGTTTCTTCCGTTG  2527570

Query  361      ATCAAAATTCTATACGTAGATCGGAATAAATTAACTCTAACGGAAACGGATGTGGAAATT  420
                ||||||||||||||||||||||||||||||||||||||||||||||||||||||||||||
Sbjct  2527571  ATCAAAATTCTATACGTAGATCGGAATAAATTAACTCTAACGGAAACGGATGTGGAAATT  2527630

Query  421      CTCGCTTCTCTTTCCTCCTTGGAGGAATTGGATCTGAGTCTGAATTCGGGGATTAAGGCG  480
                ||||||||||||||||||||||||||||||||||||||||||||||||||||||||||||
Sbjct  2527631  CTCGCTTCTCTTTCCTCCTTGGAGGAATTGGATCTGAGTCTGAATTCGGGGATTAAGGCG  2527690

Query  481      CTCCCTTTTAATTACGAAAAACTTGTAAATCTCATTAATCTAAAAAGATTGAATATTaaa  540
                ||||||||||||||||||||||||||||||||||||||||||||||||||||||||||||
Sbjct  2527691  CTCCCTTTTAATTACGAAAAACTTGTAAATCTCATTAATCTAAAAAGATTGAATATTAAA  2527750

Query  541      aaaaCTTCATTAAAGGGAGAGGATGCAGATAAATTGCAGGCGATTCTTCCAAATACCAAA  600
                ||||||||||||||||||||||||||||||||||||||||||||||||||||| ||||||
Sbjct  2527751  AAAACTTCATTAAAGGGAGAGGATGCAGATAAATTGCAGGCGATTCTTCCAAACACCAAA  2527810

Query  601      ATCGACTACTGA  612
                ||||||||||||
Sbjct  2527811  ATCGACTACTGA  2527822
```

>Leptospira borgpetersenii str. 4E chromosome 1
Sequence ID: CP015814.2 Length: 3550837
Range 1: 2151282 to 2151893

Score:1125 bits(609), Expect:0.0,
Identities:611/612(99%), Gaps:0/612(0%), Strand: Plus/Plus

```
Query  1        GGCTTCGTTTGTTTTACTGCGAGTTTCGACTGTAAAAAGAATGCGGAAGAGATTTTGGGA  60
                ||||||||||||||||||||||||||||||||||||||||||||||||||||||||||||
Sbjct  2151282  GGCTTCGTTTGTTTTACTGCGAGTTTCGACTGTAAAAAGAATGCGGAAGAGATTTTGGGA  2151341

Query  61       GAGGCAAAGGCAAAACCGGAATTGGTTCAAACTTTGGATTTTGGAATGCAGAAGTTGTCC  120
                ||||||||||||||||||||||||||||||||||||||||||||||||||||||||||||
Sbjct  2151342  GAGGCAAAGGCAAAACCGGAATTGGTTCAAACTTTGGATTTTGGAATGCAGAAGTTGTCC  2151401
```

```
Query  121      ACGGTTCCGGAAGAAGTTTGCGGTTTTCCGAATTTAACCAAGTTGGATCTTCGTTTAAAC  180
                ||||||||||||||||||||||||||||||||||||||||||||||||||||||||||||
Sbjct  2151402  ACGGTTCCGGAAGAAGTTTGCGGTTTTCCGAATTTAACCAAGTTGGATCTTCGTTTAAAC  2151461

Query  181      AGTTTGACTTTTCTTCCGGAATTTATCGGAGAATGTAAACGCCTTGAACAACTTAATCTT  240
                ||||||||||||||||||||||||||||||||||||||||||||||||||||||||||||
Sbjct  2151462  AGTTTGACTTTTCTTCCGGAATTTATCGGAGAATGTAAACGCCTTGAACAACTTAATCTT  2151521

Query  241      TTCGGGAACGACCTTACAACATTTCCGTCTACGTTTTCTAAATTAAAAAATCTGAAAGTG  300
                ||||||||||||||||||||||||||||||||||||||||||||||||||||||||||||
Sbjct  2151522  TTCGGGAACGACCTTACAACATTTCCGTCTACGTTTTCTAAATTAAAAAATCTGAAAGTG  2151581

Query  301      TTACTGGCGGGAAATAACGATTTTACGATTCTACCCTCCGAACTTTTGTTTCTTCCGTTG  360
                ||||||||||||||||||||||||||||||||||||||||||||||||||||||||||||
Sbjct  2151582  TTACTGGCGGGAAATAACGATTTTACGATTCTACCCTCCGAACTTTTGTTTCTTCCGTTG  2151641

Query  361      ATCAAAATTCTATACGTAGATCGGAATAAATTAACTCTAACGGAAACGGATGTGGAAATT  420
                ||||||||||||||||||||||||||||||||||||||||||||||||||||||||||||
Sbjct  2151642  ATCAAAATTCTATACGTAGATCGGAATAAATTAACTCTAACGGAAACGGATGTGGAAATT  2151701

Query  421      CTCGCTTCTCTTTCCTCCTTGGAGGAATTGGATCTGAGTCTGAATTCGGGGATTAAGGCG  480
                ||||||||||||||||||||||||||||||||||||||||||||||||||||||||||||
Sbjct  2151702  CTCGCTTCTCTTTCCTCCTTGGAGGAATTGGATCTGAGTCTGAATTCGGGGATTAAGGCG  2151761

Query  481      CTCCCTTTTAATTACGAAAAACTTGTAAATCTCATTAATCTAAAAAGATTGAATATTaaa  540
                ||||||||||||||||||||||||||||||||||||||||||||||||||||||||||||
Sbjct  2151762  CTCCCTTTTAATTACGAAAAACTTGTAAATCTCATTAATCTAAAAAGATTGAATATTAAA  2151821

Query  541      aaaaCTTCATTAAAGGGAGAGGATGCAGATAAATTGCAGGCGATTCTTCCAAATACCAAA  600
                ||||||||||||||||||||||||||||||||||||||||||||||||||||| ||||||
Sbjct  2151822  AAAACTTCATTAAAGGGAGAGGATGCAGATAAATTGCAGGCGATTCTTCCAAACACCAAA  2151881

Query  601      ATCGACTACTGA  612
                ||||||||||||
Sbjct  2151882  ATCGACTACTGA  2151893
```

```
>Leptospira borgpetersenii serovar Ballum strain 56604 chromosome 1, complete sequence
Sequence ID: CP012029.1 Length: 3550837
Range 1: 2151282 to 2151893

Score:1125 bits(609), Expect:0.0,
Identities:611/612(99%),  Gaps:0/612(0%), Strand: Plus/Plus

Query  1        GGCTTCGTTTGTTTTACTGCGAGTTTCGACTGTAAAAAGAATGCGGAAGAGATTTTGGGA  60
                ||||||||||||||||||||||||||||||||||||||||||||||||||||||||||||
Sbjct  2151282  GGCTTCGTTTGTTTTACTGCGAGTTTCGACTGTAAAAAGAATGCGGAAGAGATTTTGGGA  2151341

Query  61       GAGGCAAAGGCAAAACCGGAATTGGTTCAAACTTTGGATTTTGGAATGCAGAAGTTGTCC  120
                ||||||||||||||||||||||||||||||||||||||||||||||||||||||||||||
Sbjct  2151342  GAGGCAAAGGCAAAACCGGAATTGGTTCAAACTTTGGATTTTGGAATGCAGAAGTTGTCC  2151401

Query  121      ACGGTTCCGGAAGAAGTTTGCGGTTTTCCGAATTTAACCAAGTTGGATCTTCGTTTAAAC  180
                ||||||||||||||||||||||||||||||||||||||||||||||||||||||||||||
Sbjct  2151402  ACGGTTCCGGAAGAAGTTTGCGGTTTTCCGAATTTAACCAAGTTGGATCTTCGTTTAAAC  2151461

Query  181      AGTTTGACTTTTCTTCCGGAATTTATCGGAGAATGTAAACGCCTTGAACAACTTAATCTT  240
                ||||||||||||||||||||||||||||||||||||||||||||||||||||||||||||
Sbjct  2151462  AGTTTGACTTTTCTTCCGGAATTTATCGGAGAATGTAAACGCCTTGAACAACTTAATCTT  2151521

Query  241      TTCGGGAACGACCTTACAACATTTCCGTCTACGTTTTCTAAATTAAAAAATCTGAAAGTG  300
```

```
                   ||||||||||||||||||||||||||||||||||||||||||||||||||||||||||||
Sbjct  2151522  TTCGGGAACGACCTTACAACATTTCCGTCTACGTTTTCTAAATTAAAAAATCTGAAAGTG  2151581

Query  301      TTACTGGCGGGAAATAACGATTTTACGATTCTACCCTCCGAACTTTTGTTTCTTCCGTTG  360
                ||||||||||||||||||||||||||||||||||||||||||||||||||||||||||||
Sbjct  2151582  TTACTGGCGGGAAATAACGATTTTACGATTCTACCCTCCGAACTTTTGTTTCTTCCGTTG  2151641

Query  361      ATCAAAATTCTATACGTAGATCGGAATAAATTAACTCTAACGGAAACGGATGTGGAAATT  420
                ||||||||||||||||||||||||||||||||||||||||||||||||||||||||||||
Sbjct  2151642  ATCAAAATTCTATACGTAGATCGGAATAAATTAACTCTAACGGAAACGGATGTGGAAATT  2151701

Query  421      CTCGCTTCTCTTTCCTCCTTGGAGGAATTGGATCTGAGTCTGAATTCGGGGATTAAGGCG  480
                ||||||||||||||||||||||||||||||||||||||||||||||||||||||||||||
Sbjct  2151702  CTCGCTTCTCTTTCCTCCTTGGAGGAATTGGATCTGAGTCTGAATTCGGGGATTAAGGCG  2151761

Query  481      CTCCCTTTTAATTACGAAAAACTTGTAAATCTCATTAATCTAAAAAGATTGAATATTaaa  540
                ||||||||||||||||||||||||||||||||||||||||||||||||||||||||||||
Sbjct  2151762  CTCCCTTTTAATTACGAAAAACTTGTAAATCTCATTAATCTAAAAAGATTGAATATTAAA  2151821

Query  541      aaaaCTTCATTAAAGGGAGAGGATGCAGATAAATTGCAGGCGATTCTTCCAAATACCAAA  600
                ||||||||||||||||||||||||||||||||||||||||||||||||||||| ||||||
Sbjct  2151822  AAAACTTCATTAAAGGGAGAGGATGCAGATAAATTGCAGGCGATTCTTCCAAACACCAAA  2151881

Query  601      ATCGACTACTGA  612
                ||||||||||||
Sbjct  2151882  ATCGACTACTGA  2151893
```

>Leptospira borgpetersenii serovar Mini leucine-rich repeat protein (LRR) gene, partial cds
Sequence ID: JX522459.1 Length: 612
Range 1: 1 to 612

Score:1125 bits(609), Expect:0.0,
Identities:611/612(99%), Gaps:0/612(0%), Strand: Plus/Plus

```
Query  1    GGCTTCGTTTGTTTTACTGCGAGTTTCGACTGTAAAAAGAATGCGGAAGAGATTTTGGGA  60
            ||||||||||||||||||||||||||||||||||||||||||||||||||||||||||||
Sbjct  1    GGCTTCGTTTGTTTTACTGCGAGTTTCGACTGTAAAAAGAATGCGGAAGAGATTTTGGGA  60

Query  61   GAGGCAAAGGCAAAACCGGAATTGGTTCAAACTTTGGATTTTGGAATGCAGAAGTTGTCC  120
            ||||||||||||||||||||||||||||||||||||||||||||||||||||||||||||
Sbjct  61   GAGGCAAAGGCAAAACCGGAATTGGTTCAAACTTTGGATTTTGGAATGCAGAAGTTGTCC  120

Query  121  ACGGTTCCGGAAGAAGTTTGCGGTTTTCCGAATTTAACCAAGTTGGATCTTCGTTTAAAC  180
            ||||||||||||||||||||||||||||||||||||||||||||||||||||||||||||
Sbjct  121  ACGGTTCCGGAAGAAGTTTGCGGTTTTCCGAATTTAACCAAGTTGGATCTTCGTTTAAAC  180

Query  181  AGTTTGACTTTTCTTCCGGAATTTATCGGAGAATGTAAACGCCTTGAACAACTTAATCTT  240
            ||||||||||||||||||||||||||||||||||||||||||||||||||||||||||||
Sbjct  181  AGTTTGACTTTTCTTCCGGAATTTATCGGAGAATGTAAACGCCTTGAACAACTTAATCTT  240

Query  241  TTCGGGAACGACCTTACAACATTTCCGTCTACGTTTTCTAAATTAAAAAATCTGAAAGTG  300
            ||||||||||||||||||||||||||||||||||||||||||||||||||||||||||||
Sbjct  241  TTCGGGAACGACCTTACAACATTTCCGTCTACGTTTTCTAAATTAAAAAATCTGAAAGTG  300

Query  301  TTACTGGCGGGAAATAACGATTTTACGATTCTACCCTCCGAACTTTTGTTTCTTCCGTTG  360
            ||||||||||||||||||||||||||||||||||||||||||||||||||||||||||||
Sbjct  301  TTACTGGCGGGAAATAACGATTTTACGATTCTACCCTCCGAACTTTTGTTTCTTCCGTTG  360

Query  361  ATCAAAATTCTATACGTAGATCGGAATAAATTAACTCTAACGGAAACGGATGTGGAAATT  420
```

```
                ||||||||||||||||||||||||||||||||||||||||||||||||||||||||||||
Sbjct  361  ATCAAAATTCTATACGTAGATCGGAATAAATTAACTCTAACGGAAACGGATGTGGAAATT  420

Query  421  CTCGCTTCTCTTTCCTCCTTGGAGGAATTGGATCTGAGTCTGAATTCGGGGATTAAGGCG  480
            ||||||||||||||||||||||||||||||||||||||||||||||||||||||||||||
Sbjct  421  CTCGCTTCTCTTTCCTCCTTGGAGGAATTGGATCTGAGTCTGAATTCGGGGATTAAGGCG  480

Query  481  CTCCCTTTTAATTACGAAAAACTTGTAAATCTCATTAATCTAAAAAGATTGAATATTaaa  540
            ||||||||||||||||||||||||||||||||||||||| ||||||||||||||||||||
Sbjct  481  CTCCCTTTTAATTACGAAAAACTTGTAAATCTCATTAATTTAAAAAGATTGAATATTAAA  540

Query  541  aaaaCTTCATTAAAGGGAGAGGATGCAGATAAATTGCAGGCGATTCTTCCAAATACCAAA  600
            ||||||||||||||||||||||||||||||||||||||||||||||||||||||||||||
Sbjct  541  AAAACTTCATTAAAGGGAGAGGATGCAGATAAATTGCAGGCGATTCTTCCAAATACCAAA  600

Query  601  ATCGACTACTGA  612
            ||||||||||||
Sbjct  601  ATCGACTACTGA  612
```

>Leptospira borgpetersenii serovar Ballum leucine-rich repeat protein (LRR) gene, partial cds
Sequence ID: JX522458.1 Length: 612
Range 1: 1 to 612

Score:1125 bits(609), Expect:0.0,
Identities:611/612(99%), Gaps:0/612(0%), Strand: Plus/Plus

```
Query  1    GGCTTCGTTTGTTTTACTGCGAGTTTCGACTGTAAAAAGAATGCGGAAGAGATTTTGGGA  60
            ||||||||||||||||||||||||||||||||||||||||||||||||||||||||||||
Sbjct  1    GGCTTCGTTTGTTTTACTGCGAGTTTCGACTGTAAAAAGAATGCGGAAGAGATTTTGGGA  60

Query  61   GAGGCAAAGGCAAAACCGGAATTGGTTCAAACTTTGGATTTTGGAATGCAGAAGTTGTCC  120
            ||||||||||||||||||||||||||||||||||||||||||||||||||||||||||||
Sbjct  61   GAGGCAAAGGCAAAACCGGAATTGGTTCAAACTTTGGATTTTGGAATGCAGAAGTTGTCC  120

Query  121  ACGGTTCCGGAAGAAGTTTGCGGTTTTCCGAATTTAACCAAGTTGGATCTTCGTTTAAAC  180
            ||||||||||||||||||||||||||||||||||||||||||||||||||||||||||||
Sbjct  121  ACGGTTCCGGAAGAAGTTTGCGGTTTTCCGAATTTAACCAAGTTGGATCTTCGTTTAAAC  180

Query  181  AGTTTGACTTTTCTTCCGGAATTTATCGGAGAATGTAAACGCCTTGAACAACTTAATCTT  240
            ||||||||||||||||||||||||||||||||||||||||||||||||||||||||||||
Sbjct  181  AGTTTGACTTTTCTTCCGGAATTTATCGGAGAATGTAAACGCCTTGAACAACTTAATCTT  240

Query  241  TTCGGGAACGACCTTACAACATTTCCGTCTACGTTTTCTAAATTAAAAAATCTGAAAGTG  300
            ||||||||||||||||||||||||||||||||||||||||||||||||||||||||||||
Sbjct  241  TTCGGGAACGACCTTACAACATTTCCGTCTACGTTTTCTAAATTAAAAAATCTGAAAGTG  300

Query  301  TTACTGGCGGGAAATAACGATTTTACGATTCTACCCTCCGAACTTTTGTTTCTTCCGTTG  360
            ||||||||||||||||||||||||||||||||||||||||||||||||||||||||||||
Sbjct  301  TTACTGGCGGGAAATAACGATTTTACGATTCTACCCTCCGAACTTTTGTTTCTTCCGTTG  360

Query  361  ATCAAAATTCTATACGTAGATCGGAATAAATTAACTCTAACGGAAACGGATGTGGAAATT  420
            ||||||||||||||||||||||||||||||||||||||||||||||||||||||||||||
Sbjct  361  ATCAAAATTCTATACGTAGATCGGAATAAATTAACTCTAACGGAAACGGATGTGGAAATT  420

Query  421  CTCGCTTCTCTTTCCTCCTTGGAGGAATTGGATCTGAGTCTGAATTCGGGGATTAAGGCG  480
            ||||||||||||||||||||||||||||||||||||||||||||||||||||||||||||
Sbjct  421  CTCGCTTCTCTTTCCTCCTTGGAGGAATTGGATCTGAGTCTGAATTCGGGGATTAAGGCG  480

Query  481  CTCCCTTTTAATTACGAAAAACTTGTAAATCTCATTAATCTAAAAAGATTGAATATTaaa  540
```

```
          ||||||||||||||||||||||||||||||||||||||||||||||||||||||||||||
Sbjct  481  CTCCCTTTTAATTACGAAAAACTTGTAAATCTCATTAATCTAAAAAGATTGAATATTAAA  540

Query  541  aaaaCTTCATTAAAGGGAGAGGATGCAGATAAATTGCAGGCGATTCTTCCAAATACCAAA  600
            |||||||||||||||||||||||||||||||||||||||||||||||||||||||| |||
Sbjct  541  AAAACTTCATTAAAGGGAGAGGATGCAGATAAATTGCAGGCGATTCTTCCAAATACAAAA  600

Query  601  ATCGACTACTGA  612
            ||||||||||||
Sbjct  601  ATCGACTACTGA  612
```

>Leptospira borgpetersenii strain 34-PK chromosome 1, complete sequence
Sequence ID: CP097464.1 Length: 3603305
Range 1: 943987 to 944598

Score:1120 bits(606), Expect:0.0,
Identities:610/612(99%), Gaps:0/612(0%), Strand: Plus/Plus

```
Query  1       GGCTTCGTTTGTTTTACTGCGAGTTTCGACTGTAAAAAGAATGCGGAAGAGATTTTGGGA  60
               ||||||||||||||||||||||||||||||||||||||||||||||||||||||||||||
Sbjct  943987  GGCTTCGTTTGTTTTACTGCGAGTTTCGACTGTAAAAAGAATGCGGAAGAGATTTTGGGA  944046

Query  61      GAGGCAAAGGCAAAACCGGAATTGGTTCAAACTTTGGATTTTGGAATGCAGAAGTTGTCC  120
               ||||||||||||||||||||||||||||||||||||||||||||||||||||||||||||
Sbjct  944047  GAGGCAAAGGCAAAACCGGAATTGGTTCAAACTTTGGATTTTGGAATGCAGAAGTTGTCC  944106

Query  121     ACGGTTCCGGAAGAAGTTTGCGGTTTTCCGAATTTAACCAAGTTGGATCTTCGTTTAAAC  180
               ||||||||||||||||||||||||||||||||||||||||||||||||||||||||||||
Sbjct  944107  ACGGTTCCGGAAGAAGTTTGCGGTTTTCCGAATTTAACCAAGTTGGATCTTCGTTTAAAC  944166

Query  181     AGTTTGACTTTTCTTCCGGAATTTATCGGAGAATGTAAACGCCTTGAACAACTTAATCTT  240
               ||||||||||||||||||||||||||||||||||||||||||||||||||||||||||||
Sbjct  944167  AGTTTGACTTTTCTTCCGGAATTTATCGGAGAATGTAAACGCCTTGAACAACTTAATCTT  944226

Query  241     TTCGGGAACGACCTTACAACATTTCCGTCTACGTTTTCTAAATTAAAAAATCTGAAAGTG  300
               ||||||||||||||||||||||||||||||||||||||||||||||||||||||||||||
Sbjct  944227  TTCGGGAACGACCTTACAACATTTCCGTCTACGTTTTCTAAATTAAAAAATCTGAAAGTG  944286

Query  301     TTACTGGCGGGAAATAACGATTTTACGATTCTACCCTCCGAACTTTTGTTTCTTCCGTTG  360
               ||||||||||||||||||||||||||||||||||||||||||||||||||||||||||||
Sbjct  944287  TTACTGGCGGGAAATAACGATTTTACGATTCTACCCTCCGAACTTTTGTTTCTTCCGTTG  944346

Query  361     ATCAAAATTCTATACGTAGATCGGAATAAATTAACTCTAACGGAAACGGATGTGGAAATT  420
               ||||||||| ||||||||||||||||||||||||||||||||||||||||||||||||||
Sbjct  944347  ATCAAAATTTTATACGTAGATCGGAATAAATTAACTCTAACGGAAACGGATGTGGAAATT  944406

Query  421     CTCGCTTCTCTTTCCTCCTTGGAGGAATTGGATCTGAGTCTGAATTCGGGGATTAAGGCG  480
               ||||||||||||||||||||||||||||||||||||||||||||||||||||||||||||
Sbjct  944407  CTCGCTTCTCTTTCCTCCTTGGAGGAATTGGATCTGAGTCTGAATTCGGGGATTAAGGCG  944466

Query  481     CTCCCTTTTAATTACGAAAAACTTGTAAATCTCATTAATCTAAAAAGATTGAATATTaaa  540
               ||||||||||||||||||||||||||||||||||||||||||||||||||||||||||||
Sbjct  944467  CTCCCTTTTAATTACGAAAAACTTGTAAATCTCATTAATCTAAAAAGATTGAATATTAAA  944526

Query  541     aaaaCTTCATTAAAGGGAGAGGATGCAGATAAATTGCAGGCGATTCTTCCAAATACCAAA  600
               ||||||||||||||||||||||||||||||||||||||||||||||||||||| ||||||
Sbjct  944527  AAAACTTCATTAAAGGGAGAGGATGCAGATAAATTGCAGGCGATTCTTCCAAACACCAAA  944586

Query  601     ATCGACTACTGA  612
               ||||||||||||
```

```
Sbjct  944587  ATCGACTACTGA  944598
```

>Leptospira borgpetersenii serovar Ceylonica strain Piyasena chromosome 1, complete sequence
Sequence ID: CP026671.1 Length: 3670231
Range 1: 3098426 to 3099037

Score:1114 bits(603), Expect:0.0,
Identities:609/612(99%), Gaps:0/612(0%), Strand: Plus/Minus

```
Query  1        GGCTTCGTTTGTTTTACTGCGAGTTTCGACTGTAAAAAGAATGCGGAAGAGATTTTGGGA  60
                ||||||||||||||||||||||||||||||||||||||||||||||||||||||||||||
Sbjct  3099037  GGCTTCGTTTGTTTTACTGCGAGTTTCGACTGTAAAAAGAATGCGGAAGAGATTTTGGGA  3098978

Query  61       GAGGCAAAGGCAAAACCGGAATTGGTTCAAACTTTGGATTTTGGAATGCAGAAGTTGTCC  120
                ||||||||||||||||||||||||||||||||||||||||||||||||||||||||||||
Sbjct  3098977  GAGGCAAAGGCAAAACCGGAATTGGTTCAAACTTTGGATTTTGGAATGCAGAAGTTGTCC  3098918

Query  121      ACGGTTCCGGAAGAAGTTTGCGGTTTTCCGAATTTAACCAAGTTGGATCTTCGTTTAAAC  180
                ||||||||||||||||||||||||||||||||||||||||||||||||||||||||||||
Sbjct  3098917  ACGGTTCCGGAAGAAGTTTGCGGTTTTCCGAATTTAACCAAGTTGGATCTTCGTTTAAAC  3098858

Query  181      AGTTTGACTTTTCTTCCGGAATTTATCGGAGAATGTAAACGCCTTGAACAACTTAATCTT  240
                ||||||||||||||||||||||||||||||||||||||||||||||||||||||||||||
Sbjct  3098857  AGTTTGACTTTTCTTCCGGAATTTATCGGAGAATGTAAACGCCTTGAACAACTTAATCTT  3098798

Query  241      TTCGGGAACGACCTTACAACATTTCCGTCTACGTTTTCTAAATTAAAAAATCTGAAAGTG  300
                ||||||||||| ||||||||||||||||||||||||||||||||||||||||||||||||
Sbjct  3098797  TTCGGGAACGATCTTACAACATTTCCGTCTACGTTTTCTAAATTAAAAAATCTGAAAGTG  3098738

Query  301      TTACTGGCGGGAAATAACGATTTTACGATTCTACCCTCCGAACTTTTGTTTCTTCCGTTG  360
                ||||||||||||||||||||||||||||||||||||||||||||||||||||||||||||
Sbjct  3098737  TTACTGGCGGGAAATAACGATTTTACGATTCTACCCTCCGAACTTTTGTTTCTTCCGTTG  3098678

Query  361      ATCAAAATTCTATACGTAGATCGGAATAAATTAACTCTAACGGAAACGGATGTGGAAATT  420
                ||||||||| ||||||||||||||||||||||||||||||||||||||||||||||||||
Sbjct  3098677  ATCAAAATTTTATACGTAGATCGGAATAAATTAACTCTAACGGAAACGGATGTGGAAATT  3098618

Query  421      CTCGCTTCTCTTTCCTCCTTGGAGGAATTGGATCTGAGTCTGAATTCGGGGATTAAGGCG  480
                ||||||||||||||||||||||||||||||||||||||||||||||||||||||||||||
Sbjct  3098617  CTCGCTTCTCTTTCCTCCTTGGAGGAATTGGATCTGAGTCTGAATTCGGGGATTAAGGCG  3098558

Query  481      CTCCCTTTTAATTACGAAAAACTTGTAAATCTCATTAATCTAAAAAGATTGAATATTaaa  540
                ||||||||||||||||||||||||||||||||||||||||||||||||||||||||||||
Sbjct  3098557  CTCCCTTTTAATTACGAAAAACTTGTAAATCTCATTAATCTAAAAAGATTGAATATTAAA  3098498

Query  541      aaaaCTTCATTAAAGGGAGAGGATGCAGATAAATTGCAGGCGATTCTTCCAAATACCAAA  600
                ||||||||||||||||||||||||||||||||||||||||||||||||||||| ||||||
Sbjct  3098497  AAAACTTCATTAAAGGGAGAGGATGCAGATAAATTGCAGGCGATTCTTCCAAACACCAAA  3098438

Query  601      ATCGACTACTGA  612
                ||||||||||||
Sbjct  3098437  ATCGACTACTGA  3098426
```

>Leptospira borgpetersenii strain FMAS_AP2 chromosome 1, complete sequence
Sequence ID: CP072630.1 Length: 3595436
Range 1: 939788 to 940399

```
Score:1114 bits(603), Expect:0.0,
Identities:609/612(99%),  Gaps:0/612(0%), Strand: Plus/Plus

Query  1       GGCTTCGTTTGTTTTACTGCGAGTTTCGACTGTAAAAAGAATGCGGAAGAGATTTTGGGA  60
               ||||||||||||||||||||||||||||||||||||||||||||||||||||||||||||
Sbjct  939788  GGCTTCGTTTGTTTTACTGCGAGTTTCGACTGTAAAAAGAATGCGGAAGAGATTTTGGGA  939847

Query  61      GAGGCAAAGGCAAAACCGGAATTGGTTCAAACTTTGGATTTTGGAATGCAGAAGTTGTCC  120
               ||||||||||||||||||||||||||||||||||||||||||||||||||||||||||||
Sbjct  939848  GAGGCAAAGGCAAAACCGGAATTGGTTCAAACTTTGGATTTTGGAATGCAGAAGTTGTCC  939907

Query  121     ACGGTTCCGGAAGAAGTTTGCGGTTTTCCGAATTTAACCAAGTTGGATCTTCGTTTAAAC  180
               ||||||||||||||||||||||||||||||||||||||||||||||||||||||||||||
Sbjct  939908  ACGGTTCCGGAAGAAGTTTGCGGTTTTCCGAATTTAACCAAGTTGGATCTTCGTTTAAAC  939967

Query  181     AGTTTGACTTTTCTTCCGGAATTTATCGGAGAATGTAAACGCCTTGAACAACTTAATCTT  240
               ||||||||||||||||||||||||||||||||||||||||||||||||||||||||||||
Sbjct  939968  AGTTTGACTTTTCTTCCGGAATTTATCGGAGAATGTAAACGCCTTGAACAACTTAATCTT  940027

Query  241     TTCGGGAACGACCTTACAACATTTCCGTCTACGTTTTCTAAATTAAAAAATCTGAAAGTG  300
               ||||||||||| ||||||||||||||||||||||||||||||||||||||||||||||||
Sbjct  940028  TTCGGGAACGATCTTACAACATTTCCGTCTACGTTTTCTAAATTAAAAAATCTGAAAGTG  940087

Query  301     TTACTGGCGGGAAATAACGATTTTACGATTCTACCCTCCGAACTTTTGTTTCTTCCGTTG  360
               ||||||||||||||||||||||||||||||||||||||||||||||||||||||||||||
Sbjct  940088  TTACTGGCGGGAAATAACGATTTTACGATTCTACCCTCCGAACTTTTGTTTCTTCCGTTG  940147

Query  361     ATCAAAATTCTATACGTAGATCGGAATAAATTAACTCTAACGGAAACGGATGTGGAAATT  420
               ||||||||| ||||||||||||||||||||||||||||||||||||||||||||||||||
Sbjct  940148  ATCAAAATTTTATACGTAGATCGGAATAAATTAACTCTAACGGAAACGGATGTGGAAATT  940207

Query  421     CTCGCTTCTCTTTCCTCCTTGGAGGAATTGGATCTGAGTCTGAATTCGGGGATTAAGGCG  480
               ||||||||||||||||||||||||||||||||||||||||||||||||||||||||||||
Sbjct  940208  CTCGCTTCTCTTTCCTCCTTGGAGGAATTGGATCTGAGTCTGAATTCGGGGATTAAGGCG  940267

Query  481     CTCCCTTTTAATTACGAAAAACTTGTAAATCTCATTAATCTAAAAAGATTGAATATTaaa  540
               ||||||||||||||||||||||||||||||||||||||||||||||||||||||||||||
Sbjct  940268  CTCCCTTTTAATTACGAAAAACTTGTAAATCTCATTAATCTAAAAAGATTGAATATTAAA  940327

Query  541     aaaaCTTCATTAAAGGGAGAGGATGCAGATAAATTGCAGGCGATTCTTCCAAATACCAAA  600
               |||||||||||||||||||||||||||||||||||||||||||||||||||||| ||||||
Sbjct  940328  AAAACTTCATTAAAGGGAGAGGATGCAGATAAATTGCAGGCGATTCTTCCAAACACCAAA  940387

Query  601     ATCGACTACTGA  612
               ||||||||||||
Sbjct  940388  ATCGACTACTGA  940399



>Leptospira borgpetersenii strain FMAS_AP3 chromosome 1, complete sequence
Sequence ID: CP072628.1 Length: 3595898
Range 1: 939787 to 940398

Score:1114 bits(603), Expect:0.0,
Identities:609/612(99%),  Gaps:0/612(0%), Strand: Plus/Plus

Query  1       GGCTTCGTTTGTTTTACTGCGAGTTTCGACTGTAAAAAGAATGCGGAAGAGATTTTGGGA  60
               ||||||||||||||||||||||||||||||||||||||||||||||||||||||||||||
Sbjct  939787  GGCTTCGTTTGTTTTACTGCGAGTTTCGACTGTAAAAAGAATGCGGAAGAGATTTTGGGA  939846

Query  61      GAGGCAAAGGCAAAACCGGAATTGGTTCAAACTTTGGATTTTGGAATGCAGAAGTTGTCC  120
```

```
                  ||||||||||||||||||||||||||||||||||||||||||||||||||||||||||||
Sbjct  939847  GAGGCAAAGGCAAAACCGGAATTGGTTCAAACTTTGGATTTTGGAATGCAGAAGTTGTCC  939906

Query  121     ACGGTTCCGGAAGAAGTTTGCGGTTTTCCGAATTTAACCAAGTTGGATCTTCGTTTAAAC  180
               ||||||||||||||||||||||||||||||||||||||||||||||||||||||||||||
Sbjct  939907  ACGGTTCCGGAAGAAGTTTGCGGTTTTCCGAATTTAACCAAGTTGGATCTTCGTTTAAAC  939966

Query  181     AGTTTGACTTTTCTTCCGGAATTTATCGGAGAATGTAAACGCCTTGAACAACTTAATCTT  240
               ||||||||||||||||||||||||||||||||||||||||||||||||||||||||||||
Sbjct  939967  AGTTTGACTTTTCTTCCGGAATTTATCGGAGAATGTAAACGCCTTGAACAACTTAATCTT  940026

Query  241     TTCGGGAACGACCTTACAACATTTCCGTCTACGTTTTCTAAATTAAAAAATCTGAAAGTG  300
               ||||||||||| ||||||||||||||||||||||||||||||||||||||||||||||||
Sbjct  940027  TTCGGGAACGATCTTACAACATTTCCGTCTACGTTTTCTAAATTAAAAAATCTGAAAGTG  940086

Query  301     TTACTGGCGGGAAATAACGATTTTACGATTCTACCCTCCGAACTTTTGTTTCTTCCGTTG  360
               ||||||||||||||||||||||||||||||||||||||||||||||||||||||||||||
Sbjct  940087  TTACTGGCGGGAAATAACGATTTTACGATTCTACCCTCCGAACTTTTGTTTCTTCCGTTG  940146

Query  361     ATCAAAATTCTATACGTAGATCGGAATAAATTAACTCTAACGGAAACGGATGTGGAAATT  420
               ||||||||| ||||||||||||||||||||||||||||||||||||||||||||||||||
Sbjct  940147  ATCAAAATTTTATACGTAGATCGGAATAAATTAACTCTAACGGAAACGGATGTGGAAATT  940206

Query  421     CTCGCTTCTCTTTCCTCCTTGGAGGAATTGGATCTGAGTCTGAATTCGGGGATTAAGGCG  480
               ||||||||||||||||||||||||||||||||||||||||||||||||||||||||||||
Sbjct  940207  CTCGCTTCTCTTTCCTCCTTGGAGGAATTGGATCTGAGTCTGAATTCGGGGATTAAGGCG  940266

Query  481     CTCCCTTTTAATTACGAAAAACTTGTAAATCTCATTAATCTAAAAAGATTGAATATTaaa  540
               ||||||||||||||||||||||||||||||||||||||||||||||||||||||||||||
Sbjct  940267  CTCCCTTTTAATTACGAAAAACTTGTAAATCTCATTAATCTAAAAAGATTGAATATTAAA  940326

Query  541     aaaaCTTCATTAAAGGGAGAGGATGCAGATAAATTGCAGGCGATTCTTCCAAATACCAAA  600
               ||||||||||||||||||||||||||||||||||||||||||||||||||||| ||||||
Sbjct  940327  AAAACTTCATTAAAGGGAGAGGATGCAGATAAATTGCAGGCGATTCTTCCAAACACCAAA  940386

Query  601     ATCGACTACTGA  612
               ||||||||||||
Sbjct  940387  ATCGACTACTGA  940398
```

>Leptospira borgpetersenii strain FMAS_AP4 chromosome 1, complete sequence
Sequence ID: CP072626.1 Length: 3595848
Range 1: 939937 to 940548

Score:1114 bits(603), Expect:0.0,
Identities:609/612(99%), Gaps:0/612(0%), Strand: Plus/Plus

```
Query  1       GGCTTCGTTTGTTTTACTGCGAGTTTCGACTGTAAAAAGAATGCGGAAGAGATTTTGGGA  60
               ||||||||||||||||||||||||||||||||||||||||||||||||||||||||||||
Sbjct  939937  GGCTTCGTTTGTTTTACTGCGAGTTTCGACTGTAAAAAGAATGCGGAAGAGATTTTGGGA  939996

Query  61      GAGGCAAAGGCAAAACCGGAATTGGTTCAAACTTTGGATTTTGGAATGCAGAAGTTGTCC  120
               ||||||||||||||||||||||||||||||||||||||||||||||||||||||||||||
Sbjct  939997  GAGGCAAAGGCAAAACCGGAATTGGTTCAAACTTTGGATTTTGGAATGCAGAAGTTGTCC  940056

Query  121     ACGGTTCCGGAAGAAGTTTGCGGTTTTCCGAATTTAACCAAGTTGGATCTTCGTTTAAAC  180
               ||||||||||||||||||||||||||||||||||||||||||||||||||||||||||||
Sbjct  940057  ACGGTTCCGGAAGAAGTTTGCGGTTTTCCGAATTTAACCAAGTTGGATCTTCGTTTAAAC  940116

Query  181     AGTTTGACTTTTCTTCCGGAATTTATCGGAGAATGTAAACGCCTTGAACAACTTAATCTT  240
               ||||||||||||||||||||||||||||||||||||||||||||||||||||||||||||
```

```
Sbjct  940117  AGTTTGACTTTTCTTCCGGAATTTATCGGAGAATGTAAACGCCTTGAACAACTTAATCTT  940176

Query  241     TTCGGGAACGACCTTACAACATTTCCGTCTACGTTTTCTAAATTAAAAAATCTGAAAGTG  300
               ||||||||||| ||||||||||||||||||||||||||||||||||||||||||||||||
Sbjct  940177  TTCGGGAACGATCTTACAACATTTCCGTCTACGTTTTCTAAATTAAAAAATCTGAAAGTG  940236

Query  301     TTACTGGCGGGAAATAACGATTTTACGATTCTACCCTCCGAACTTTTGTTTCTTCCGTTG  360
               ||||||||||||||||||||||||||||||||||||||||||||||||||||||||||||
Sbjct  940237  TTACTGGCGGGAAATAACGATTTTACGATTCTACCCTCCGAACTTTTGTTTCTTCCGTTG  940296

Query  361     ATCAAAATTCTATACGTAGATCGGAATAAATTAACTCTAACGGAAACGGATGTGGAAATT  420
               ||||||||| ||||||||||||||||||||||||||||||||||||||||||||||||||
Sbjct  940297  ATCAAAATTTTATACGTAGATCGGAATAAATTAACTCTAACGGAAACGGATGTGGAAATT  940356

Query  421     CTCGCTTCTCTTTCCTCCTTGGAGGAATTGGATCTGAGTCTGAATTCGGGGATTAAGGCG  480
               ||||||||||||||||||||||||||||||||||||||||||||||||||||||||||||
Sbjct  940357  CTCGCTTCTCTTTCCTCCTTGGAGGAATTGGATCTGAGTCTGAATTCGGGGATTAAGGCG  940416

Query  481     CTCCCTTTTAATTACGAAAAACTTGTAAATCTCATTAATCTAAAAAGATTGAATATTaaa  540
               ||||||||||||||||||||||||||||||||||||||||||||||||||||||||||||
Sbjct  940417  CTCCCTTTTAATTACGAAAAACTTGTAAATCTCATTAATCTAAAAAGATTGAATATTAAA  940476

Query  541     aaaaCTTCATTAAAGGGAGAGGATGCAGATAAATTGCAGGCGATTCTTCCAAATACCAAA  600
               ||||||||||||||||||||||||||||||||||||||||||||||||||||| ||||||
Sbjct  940477  AAAACTTCATTAAAGGGAGAGGATGCAGATAAATTGCAGGCGATTCTTCCAAACACCAAA  940536

Query  601     ATCGACTACTGA  612
               ||||||||||||
Sbjct  940537  ATCGACTACTGA  940548
```

```
>Leptospira borgpetersenii strain FMAS_PN1 chromosome 1, complete sequence
Sequence ID: CP072624.1 Length: 3596207
Range 1: 940706 to 941317

Score:1114 bits(603), Expect:0.0,
Identities:609/612(99%),  Gaps:0/612(0%), Strand: Plus/Plus

Query  1       GGCTTCGTTTGTTTTACTGCGAGTTTCGACTGTAAAAAGAATGCGGAAGAGATTTTGGGA  60
               ||||||||||||||||||||||||||||||||||||||||||||||||||||||||||||
Sbjct  940706  GGCTTCGTTTGTTTTACTGCGAGTTTCGACTGTAAAAAGAATGCGGAAGAGATTTTGGGA  940765

Query  61      GAGGCAAAGGCAAAACCGGAATTGGTTCAAACTTTGGATTTTGGAATGCAGAAGTTGTCC  120
               ||||||||||||||||||||||||||||||||||||||||||||||||||||||||||||
Sbjct  940766  GAGGCAAAGGCAAAACCGGAATTGGTTCAAACTTTGGATTTTGGAATGCAGAAGTTGTCC  940825

Query  121     ACGGTTCCGGAAGAAGTTTGCGGTTTTCCGAATTTAACCAAGTTGGATCTTCGTTTAAAC  180
               ||||||||||||||||||||||||||||||||||||||||||||||||||||||||||||
Sbjct  940826  ACGGTTCCGGAAGAAGTTTGCGGTTTTCCGAATTTAACCAAGTTGGATCTTCGTTTAAAC  940885

Query  181     AGTTTGACTTTTCTTCCGGAATTTATCGGAGAATGTAAACGCCTTGAACAACTTAATCTT  240
               ||||||||||||||||||||||||||||||||||||||||||||||||||||||||||||
Sbjct  940886  AGTTTGACTTTTCTTCCGGAATTTATCGGAGAATGTAAACGCCTTGAACAACTTAATCTT  940945

Query  241     TTCGGGAACGACCTTACAACATTTCCGTCTACGTTTTCTAAATTAAAAAATCTGAAAGTG  300
               ||||||||||| ||||||||||||||||||||||||||||||||||||||||||||||||
Sbjct  940946  TTCGGGAACGATCTTACAACATTTCCGTCTACGTTTTCTAAATTAAAAAATCTGAAAGTG  941005

Query  301     TTACTGGCGGGAAATAACGATTTTACGATTCTACCCTCCGAACTTTTGTTTCTTCCGTTG  360
               ||||||||||||||||||||||||||||||||||||||||||||||||||||||||||||
Sbjct  941006  TTACTGGCGGGAAATAACGATTTTACGATTCTACCCTCCGAACTTTTGTTTCTTCCGTTG  941065
```

```
Query  361     ATCAAAATTCTATACGTAGATCGGAATAAATTAACTCTAACGGAAACGGATGTGGAAATT  420
               ||||||||| ||||||||||||||||||||||||||||||||||||||||||||||||||
Sbjct  941066  ATCAAAATTTTATACGTAGATCGGAATAAATTAACTCTAACGGAAACGGATGTGGAAATT  941125

Query  421     CTCGCTTCTCTTTCCTCCTTGGAGGAATTGGATCTGAGTCTGAATTCGGGGATTAAGGCG  480
               ||||||||||||||||||||||||||||||||||||||||||||||||||||||||||||
Sbjct  941126  CTCGCTTCTCTTTCCTCCTTGGAGGAATTGGATCTGAGTCTGAATTCGGGGATTAAGGCG  941185

Query  481     CTCCCTTTTAATTACGAAAAACTTGTAAATCTCATTAATCTAAAAAGATTGAATATTaaa  540
               ||||||||||||||||||||||||||||||||||||||||||||||||||||||||||||
Sbjct  941186  CTCCCTTTTAATTACGAAAAACTTGTAAATCTCATTAATCTAAAAAGATTGAATATTAAA  941245

Query  541     aaaaCTTCATTAAAGGGAGAGGATGCAGATAAATTGCAGGCGATTCTTCCAAATACCAAA  600
               ||||||||||||||||||||||||||||||||||||||||||||||||||||| ||||||
Sbjct  941246  AAAACTTCATTAAAGGGAGAGGATGCAGATAAATTGCAGGCGATTCTTCCAAACACCAAA  941305

Query  601     ATCGACTACTGA  612
               ||||||||||||
Sbjct  941306  ATCGACTACTGA  941317
```

```
>Leptospira borgpetersenii strain FMAS_PN4 chromosome 1, complete sequence
Sequence ID: CP072622.1 Length: 3595796
Range 1: 940221 to 940832

Score:1114 bits(603), Expect:0.0,
Identities:609/612(99%),  Gaps:0/612(0%), Strand: Plus/Plus

Query  1       GGCTTCGTTTGTTTTACTGCGAGTTTCGACTGTAAAAAGAATGCGGAAGAGATTTTGGGA  60
               ||||||||||||||||||||||||||||||||||||||||||||||||||||||||||||
Sbjct  940221  GGCTTCGTTTGTTTTACTGCGAGTTTCGACTGTAAAAAGAATGCGGAAGAGATTTTGGGA  940280

Query  61      GAGGCAAAGGCAAAACCGGAATTGGTTCAAACTTTGGATTTTGGAATGCAGAAGTTGTCC  120
               ||||||||||||||||||||||||||||||||||||||||||||||||||||||||||||
Sbjct  940281  GAGGCAAAGGCAAAACCGGAATTGGTTCAAACTTTGGATTTTGGAATGCAGAAGTTGTCC  940340

Query  121     ACGGTTCCGGAAGAAGTTTGCGGTTTTCCGAATTTAACCAAGTTGGATCTTCGTTTAAAC  180
               ||||||||||||||||||||||||||||||||||||||||||||||||||||||||||||
Sbjct  940341  ACGGTTCCGGAAGAAGTTTGCGGTTTTCCGAATTTAACCAAGTTGGATCTTCGTTTAAAC  940400

Query  181     AGTTTGACTTTTCTTCCGGAATTTATCGGAGAATGTAAACGCCTTGAACAACTTAATCTT  240
               ||||||||||||||||||||||||||||||||||||||||||||||||||||||||||||
Sbjct  940401  AGTTTGACTTTTCTTCCGGAATTTATCGGAGAATGTAAACGCCTTGAACAACTTAATCTT  940460

Query  241     TTCGGGAACGACCTTACAACATTTCCGTCTACGTTTTCTAAATTAAAAAATCTGAAAGTG  300
               ||||||||||| ||||||||||||||||||||||||||||||||||||||||||||||||
Sbjct  940461  TTCGGGAACGATCTTACAACATTTCCGTCTACGTTTTCTAAATTAAAAAATCTGAAAGTG  940520

Query  301     TTACTGGCGGGAAATAACGATTTTACGATTCTACCCTCCGAACTTTTGTTTCTTCCGTTG  360
               ||||||||||||||||||||||||||||||||||||||||||||||||||||||||||||
Sbjct  940521  TTACTGGCGGGAAATAACGATTTTACGATTCTACCCTCCGAACTTTTGTTTCTTCCGTTG  940580

Query  361     ATCAAAATTCTATACGTAGATCGGAATAAATTAACTCTAACGGAAACGGATGTGGAAATT  420
               ||||||||| ||||||||||||||||||||||||||||||||||||||||||||||||||
Sbjct  940581  ATCAAAATTTTATACGTAGATCGGAATAAATTAACTCTAACGGAAACGGATGTGGAAATT  940640

Query  421     CTCGCTTCTCTTTCCTCCTTGGAGGAATTGGATCTGAGTCTGAATTCGGGGATTAAGGCG  480
               ||||||||||||||||||||||||||||||||||||||||||||||||||||||||||||
Sbjct  940641  CTCGCTTCTCTTTCCTCCTTGGAGGAATTGGATCTGAGTCTGAATTCGGGGATTAAGGCG  940700
```

```
Query  481     CTCCCTTTTAATTACGAAAAACTTGTAAATCTCATTAATCTAAAAAGATTGAATATTaaa  540
               ||||||||||||||||||||||||||||||||||||||||||||||||||||||||||||
Sbjct  940701  CTCCCTTTTAATTACGAAAAACTTGTAAATCTCATTAATCTAAAAAGATTGAATATTAAA  940760

Query  541     aaaaCTTCATTAAAGGGAGAGGATGCAGATAAATTGCAGGCGATTCTTCCAAATACCAAA  600
               ||||||||||||||||||||||||||||||||||||||||||||||||||||| ||||||
Sbjct  940761  AAAACTTCATTAAAGGGAGAGGATGCAGATAAATTGCAGGCGATTCTTCCAAACACCAAA  940820

Query  601     ATCGACTACTGA  612
               ||||||||||||
Sbjct  940821  ATCGACTACTGA  940832
```

>Leptospira borgpetersenii strain FMAS_AP8 chromosome 1, complete sequence
Sequence ID: CP072620.1 Length: 3595870
Range 1: 939788 to 940399

Score:1114 bits(603), Expect:0.0,
Identities:609/612(99%), Gaps:0/612(0%), Strand: Plus/Plus

```
Query  1       GGCTTCGTTTGTTTTACTGCGAGTTTCGACTGTAAAAAGAATGCGGAAGAGATTTTGGGA  60
               ||||||||||||||||||||||||||||||||||||||||||||||||||||||||||||
Sbjct  939788  GGCTTCGTTTGTTTTACTGCGAGTTTCGACTGTAAAAAGAATGCGGAAGAGATTTTGGGA  939847

Query  61      GAGGCAAAGGCAAAACCGGAATTGGTTCAAACTTTGGATTTTGGAATGCAGAAGTTGTCC  120
               ||||||||||||||||||||||||||||||||||||||||||||||||||||||||||||
Sbjct  939848  GAGGCAAAGGCAAAACCGGAATTGGTTCAAACTTTGGATTTTGGAATGCAGAAGTTGTCC  939907

Query  121     ACGGTTCCGGAAGAAGTTTGCGGTTTTCCGAATTTAACCAAGTTGGATCTTCGTTTAAAC  180
               ||||||||||||||||||||||||||||||||||||||||||||||||||||||||||||
Sbjct  939908  ACGGTTCCGGAAGAAGTTTGCGGTTTTCCGAATTTAACCAAGTTGGATCTTCGTTTAAAC  939967

Query  181     AGTTTGACTTTTCTTCCGGAATTTATCGGAGAATGTAAACGCCTTGAACAACTTAATCTT  240
               ||||||||||||||||||||||||||||||||||||||||||||||||||||||||||||
Sbjct  939968  AGTTTGACTTTTCTTCCGGAATTTATCGGAGAATGTAAACGCCTTGAACAACTTAATCTT  940027

Query  241     TTCGGGAACGACCTTACAACATTTCCGTCTACGTTTTCTAAATTAAAAAATCTGAAAGTG  300
               ||||||||||| ||||||||||||||||||||||||||||||||||||||||||||||||
Sbjct  940028  TTCGGGAACGATCTTACAACATTTCCGTCTACGTTTTCTAAATTAAAAAATCTGAAAGTG  940087

Query  301     TTACTGGCGGGAAATAACGATTTTACGATTCTACCCTCCGAACTTTTGTTTCTTCCGTTG  360
               ||||||||||||||||||||||||||||||||||||||||||||||||||||||||||||
Sbjct  940088  TTACTGGCGGGAAATAACGATTTTACGATTCTACCCTCCGAACTTTTGTTTCTTCCGTTG  940147

Query  361     ATCAAAATTCTATACGTAGATCGGAATAAATTAACTCTAACGGAAACGGATGTGGAAATT  420
               ||||||||| ||||||||||||||||||||||||||||||||||||||||||||||||||
Sbjct  940148  ATCAAAATTTTATACGTAGATCGGAATAAATTAACTCTAACGGAAACGGATGTGGAAATT  940207

Query  421     CTCGCTTCTCTTTCCTCCTTGGAGGAATTGGATCTGAGTCTGAATTCGGGGATTAAGGCG  480
               ||||||||||||||||||||||||||||||||||||||||||||||||||||||||||||
Sbjct  940208  CTCGCTTCTCTTTCCTCCTTGGAGGAATTGGATCTGAGTCTGAATTCGGGGATTAAGGCG  940267

Query  481     CTCCCTTTTAATTACGAAAAACTTGTAAATCTCATTAATCTAAAAAGATTGAATATTaaa  540
               ||||||||||||||||||||||||||||||||||||||||||||||||||||||||||||
Sbjct  940268  CTCCCTTTTAATTACGAAAAACTTGTAAATCTCATTAATCTAAAAAGATTGAATATTAAA  940327

Query  541     aaaaCTTCATTAAAGGGAGAGGATGCAGATAAATTGCAGGCGATTCTTCCAAATACCAAA  600
               ||||||||||||||||||||||||||||||||||||||||||||||||||||| ||||||
Sbjct  940328  AAAACTTCATTAAAGGGAGAGGATGCAGATAAATTGCAGGCGATTCTTCCAAACACCAAA  940387

Query  601     ATCGACTACTGA  612
```

```
               ||||||||||||
Sbjct  940388  ATCGACTACTGA  940399
```

>Leptospira borgpetersenii strain FMAS_AP9 chromosome 1, complete sequence
Sequence ID: CP072618.1 Length: 3595582
Range 1: 2369403 to 2370014

Score:1114 bits(603), Expect:0.0,
Identities:609/612(99%), Gaps:0/612(0%), Strand: Plus/Minus

```
Query  1        GGCTTCGTTTGTTTTACTGCGAGTTTCGACTGTAAAAAGAATGCGGAAGAGATTTTGGGA  60
                ||||||||||||||||||||||||||||||||||||||||||||||||||||||||||||
Sbjct  2370014  GGCTTCGTTTGTTTTACTGCGAGTTTCGACTGTAAAAAGAATGCGGAAGAGATTTTGGGA  2369955

Query  61       GAGGCAAAGGCAAAACCGGAATTGGTTCAAACTTTGGATTTTGGAATGCAGAAGTTGTCC  120
                ||||||||||||||||||||||||||||||||||||||||||||||||||||||||||||
Sbjct  2369954  GAGGCAAAGGCAAAACCGGAATTGGTTCAAACTTTGGATTTTGGAATGCAGAAGTTGTCC  2369895

Query  121      ACGGTTCCGGAAGAAGTTTGCGGTTTTCCGAATTTAACCAAGTTGGATCTTCGTTTAAAC  180
                ||||||||||||||||||||||||||||||||||||||||||||||||||||||||||||
Sbjct  2369894  ACGGTTCCGGAAGAAGTTTGCGGTTTTCCGAATTTAACCAAGTTGGATCTTCGTTTAAAC  2369835

Query  181      AGTTTGACTTTTCTTCCGGAATTTATCGGAGAATGTAAACGCCTTGAACAACTTAATCTT  240
                ||||||||||||||||||||||||||||||||||||||||||||||||||||||||||||
Sbjct  2369834  AGTTTGACTTTTCTTCCGGAATTTATCGGAGAATGTAAACGCCTTGAACAACTTAATCTT  2369775

Query  241      TTCGGGAACGACCTTACAACATTTCCGTCTACGTTTTCTAAATTAAAAAATCTGAAAGTG  300
                ||||||||||| ||||||||||||||||||||||||||||||||||||||||||||||||
Sbjct  2369774  TTCGGGAACGATCTTACAACATTTCCGTCTACGTTTTCTAAATTAAAAAATCTGAAAGTG  2369715

Query  301      TTACTGGCGGGAAATAACGATTTTACGATTCTACCCTCCGAACTTTTGTTTCTTCCGTTG  360
                ||||||||||||||||||||||||||||||||||||||||||||||||||||||||||||
Sbjct  2369714  TTACTGGCGGGAAATAACGATTTTACGATTCTACCCTCCGAACTTTTGTTTCTTCCGTTG  2369655

Query  361      ATCAAAATTCTATACGTAGATCGGAATAAATTAACTCTAACGGAAACGGATGTGGAAATT  420
                ||||||||| ||||||||||||||||||||||||||||||||||||||||||||||||||
Sbjct  2369654  ATCAAAATTTTATACGTAGATCGGAATAAATTAACTCTAACGGAAACGGATGTGGAAATT  2369595

Query  421      CTCGCTTCTCTTTCCTCCTTGGAGGAATTGGATCTGAGTCTGAATTCGGGGATTAAGGCG  480
                ||||||||||||||||||||||||||||||||||||||||||||||||||||||||||||
Sbjct  2369594  CTCGCTTCTCTTTCCTCCTTGGAGGAATTGGATCTGAGTCTGAATTCGGGGATTAAGGCG  2369535

Query  481      CTCCCTTTTAATTACGAAAAACTTGTAAATCTCATTAATCTAAAAAGATTGAATATTaaa  540
                ||||||||||||||||||||||||||||||||||||||||||||||||||||||||||||
Sbjct  2369534  CTCCCTTTTAATTACGAAAAACTTGTAAATCTCATTAATCTAAAAAGATTGAATATTAAA  2369475

Query  541      aaaaCTTCATTAAAGGGAGAGGATGCAGATAAATTGCAGGCGATTCTTCCAAATACCAAA  600
                ||||||||||||||||||||||||||||||||||||||||||||||||||||| ||||||
Sbjct  2369474  AAAACTTCATTAAAGGGAGAGGATGCAGATAAATTGCAGGCGATTCTTCCAAACACCAAA  2369415

Query  601      ATCGACTACTGA  612
                ||||||||||||
Sbjct  2369414  ATCGACTACTGA  2369403
```

>Leptospira borgpetersenii serovar Javanica leucine-rich repeat protein (LRR) gene,
partial cds
Sequence ID: JX522457.1 Length: 612

```
Range 1: 1 to 612

Score:1114 bits(603), Expect:0.0,
Identities:609/612(99%),  Gaps:0/612(0%), Strand: Plus/Plus

Query  1    GGCTTCGTTTGTTTTACTGCGAGTTTCGACTGTAAAAAGAATGCGGAAGAGATTTTGGGA  60
            |||||| |||||||||||||||||||||||||||||||||||||||||||||||||||||
Sbjct  1    GGCTTCCTTTGTTTTACTGCGAGTTTCGACTGTAAAAAGAATGCGGAAGAGATTTTGGGA  60

Query  61   GAGGCAAAGGCAAAACCGGAATTGGTTCAAACTTTGGATTTTGGAATGCAGAAGTTGTCC  120
            ||||||||||||||||||||||||||||||||||||||||||||||||||||||||||||
Sbjct  61   GAGGCAAAGGCAAAACCGGAATTGGTTCAAACTTTGGATTTTGGAATGCAGAAGTTGTCC  120

Query  121  ACGGTTCCGGAAGAAGTTTGCGGTTTTCCGAATTTAACCAAGTTGGATCTTCGTTTAAAC  180
            ||||||||||||||||||||||||||||||||||||||||||||||||||||||||||||
Sbjct  121  ACGGTTCCGGAAGAAGTTTGCGGTTTTCCGAATTTAACCAAGTTGGATCTTCGTTTAAAC  180

Query  181  AGTTTGACTTTTCTTCCGGAATTTATCGGAGAATGTAAACGCCTTGAACAACTTAATCTT  240
            ||||||||||||||||||||||||||||||||||||||||||||||||||||||||||||
Sbjct  181  AGTTTGACTTTTCTTCCGGAATTTATCGGAGAATGTAAACGCCTTGAACAACTTAATCTT  240

Query  241  TTCGGGAACGACCTTACAACATTTCCGTCTACGTTTTCTAAATTAAAAAATCTGAAAGTG  300
            ||||||||||||||||||||||||||||||||||||||||||||||||||||||||||||
Sbjct  241  TTCGGGAACGACCTTACAACATTTCCGTCTACGTTTTCTAAATTAAAAAATCTGAAAGTG  300

Query  301  TTACTGGCGGGAAATAACGATTTTACGATTCTACCCTCCGAACTTTTGTTTCTTCCGTTG  360
            ||||||||||||||||||||||||||||||||||||||||||||||||||||||||||||
Sbjct  301  TTACTGGCGGGAAATAACGATTTTACGATTCTACCCTCCGAACTTTTGTTTCTTCCGTTG  360

Query  361  ATCAAAATTCTATACGTAGATCGGAATAAATTAACTCTAACGGAAACGGATGTGGAAATT  420
            ||||||||||||||||||||||||||||||||||||||||||||||||||||||||||||
Sbjct  361  ATCAAAATTCTATACGTAGATCGGAATAAATTAACTCTAACGGAAACGGATGTGGAAATT  420

Query  421  CTCGCTTCTCTTTCCTCCTTGGAGGAATTGGATCTGAGTCTGAATTCGGGGATTAAGGCG  480
            ||||||||||||||||||||||||||||||||||||||||||||||||||||||||||||
Sbjct  421  CTCGCTTCTCTTTCCTCCTTGGAGGAATTGGATCTGAGTCTGAATTCGGGGATTAAGGCG  480

Query  481  CTCCCTTTTAATTACGAAAAACTTGTAAATCTCATTAATCTAAAAAGATTGAATATTaaa  540
            ||||||||||||||||||||||||||||||||||||||| ||||||||||||||||||||
Sbjct  481  CTCCCTTTTAATTACGAAAAACTTGTAAATCTCATTAATTTAAAAAGATTGAATATTAAA  540

Query  541  aaaaCTTCATTAAAGGGAGAGGATGCAGATAAATTGCAGGCGATTCTTCCAAATACCAAA  600
            ||||||||||||||||||||||||||||||||||||||||||||||||| ||||||||||
Sbjct  541  AAAACTTCATTAAAGGGAGAGGATGCAGATAAATTGCAGGCGATTCTTCGAAATACCAAA  600

Query  601  ATCGACTACTGA  612
            ||||||||||||
Sbjct  601  ATCGACTACTGA  612



>Leptospira borgpetersenii serovar Hardjo strain NVSL S 818 chromosome 1
Sequence ID: CP015052.1 Length: 3584464
Range 1: 2714429 to 2715040

Score:1070 bits(579), Expect:0.0,
Identities:601/612(98%),  Gaps:0/612(0%), Strand: Plus/Minus

Query  1        GGCTTCGTTTGTTTTACTGCGAGTTTCGACTGTAAAAAGAATGCGGAAGAGATTTTGGGA  60
                |||||||||||||||||||||||||| |||||||||||||||||||||||||||||||||
Sbjct  2715040  GGCTTCGTTTGTTTTACTGCGAGTTTCAACTGTAAAAAGAATGCGGAAGAGATTTTGGGA  2714981
```

```
Query  61       GAGGCAAAGGCAAAACCGGAATTGGTTCAAACTTTGGATTTTGGAATGCAGAAGTTGTCC  120
                ||||||||||||||||||||||||||||||||||||||||||||||||||||||||||||
Sbjct  2714980  GAGGCAAAGGCAAAACCGGAATTGGTTCAAACTTTGGATTTTGGAATGCAGAAGTTGTCC  2714921

Query  121      ACGGTTCCGGAAGAAGTTTGCGGTTTTCCGAATTTAACCAAGTTGGATCTTCGTTTAAAC  180
                ||||||||||||| ||||||||||||||||||||||||||||||||||||||||||||||
Sbjct  2714920  ACGGTTCCGGAAGGAGTTTGCGGTTTTCCGAATTTAACCAAGTTGGATCTTCGTTTAAAC  2714861

Query  181      AGTTTGACTTTTCTTCCGGAATTTATCGGAGAATGTAAACGCCTTGAACAACTTAATCTT  240
                ||||||||||||||||||||||||||||||||||||||||||||||||||||||||||||
Sbjct  2714860  AGTTTGACTTTTCTTCCGGAATTTATCGGAGAATGTAAACGCCTTGAACAACTTAATCTT  2714801

Query  241      TTCGGGAACGACCTTACAACATTTCCGTCTACGTTTTCTAAATTAAAAAATCTGAAAGTG  300
                ||||||||||||||||| ||||||||||||||||||||||||||||||||||||||||||
Sbjct  2714800  TTCGGGAACGACCTTACGACATTTCCGTCTACGTTTTCTAAATTAAAAAATCTGAAAGTG  2714741

Query  301      TTACTGGCGGGAAATAACGATTTTACGATTCTACCCTCCGAACTTTTGTTTCTTCCGTTG  360
                ||||||||||||||||||||||||||||||||||| |||||||||||||||||||| |||
Sbjct  2714740  TTACTGGCGGGAAATAACGATTTTACGATTCTACCTTCCGAACTTTTGTTTCTTCCATTG  2714681

Query  361      ATCAAAATTCTATACGTAGATCGGAATAAATTAACTCTAACGGAAACGGATGTGGAAATT  420
                ||||||||| |||||||||||||||||||||||| |||||||||||||||||||||||||
Sbjct  2714680  ATCAAAATTTTATACGTAGATCGGAATAAATTAATTCTAACGGAAACGGATGTGGAAATT  2714621

Query  421      CTCGCTTCTCTTTCCTCCTTGGAGGAATTGGATCTGAGTCTGAATTCGGGGATTAAGGCG  480
                ||||||||||||||||||||||||||||||||||||||||||||||||||||||||||||
Sbjct  2714620  CTCGCTTCTCTTTCCTCCTTGGAGGAATTGGATCTGAGTCTGAATTCGGGGATTAAGGCG  2714561

Query  481      CTCCCTTTTAATTACGAAAAACTTGTAAATCTCATTAATCTAAAAAGATTGAATATTaaa  540
                || ||||||||||||||||||||||| |||||||||||| ||||||||||||||||||||
Sbjct  2714560  CTTCCTTTTAATTACGAAAAACTTGTGAATCTCATTAATTTAAAAAGATTGAATATTAAA  2714501

Query  541      aaaaCTTCATTAAAGGGAGAGGATGCAGATAAATTGCAGGCGATTCTTCCAAATACCAAA  600
                ||||| ||||||||||||||||||||||||||||||||||||||||||||||||||||||
Sbjct  2714500  AAAACATCATTAAAGGGAGAGGATGCAGATAAATTGCAGGCGATTCTTCCAAATACCAAA  2714441

Query  601      ATCGACTACTGA  612
                ||||||||||||
Sbjct  2714440  ATCGACTACTGA  2714429
```

>Leptospira borgpetersenii serovar Hardjo-bovis JB197 chromosome 1, complete sequence
Sequence ID: CP000350.1 Length: 3576473
Range 1: 2702099 to 2702710

Score:1070 bits(579), Expect:0.0,
Identities:601/612(98%), Gaps:0/612(0%), Strand: Plus/Minus

```
Query  1        GGCTTCGTTTGTTTTACTGCGAGTTTCGACTGTAAAAAGAATGCGGAAGAGATTTTGGGA  60
                ||||||||||||||||||||||||||| ||||||||||||||||||||||||||||||||
Sbjct  2702710  GGCTTCGTTTGTTTTACTGCGAGTTTCAACTGTAAAAAGAATGCGGAAGAGATTTTGGGA  2702651

Query  61       GAGGCAAAGGCAAAACCGGAATTGGTTCAAACTTTGGATTTTGGAATGCAGAAGTTGTCC  120
                ||||||||||||||||||||||||||||||||||||||||||||||||||||||||||||
Sbjct  2702650  GAGGCAAAGGCAAAACCGGAATTGGTTCAAACTTTGGATTTTGGAATGCAGAAGTTGTCC  2702591

Query  121      ACGGTTCCGGAAGAAGTTTGCGGTTTTCCGAATTTAACCAAGTTGGATCTTCGTTTAAAC  180
                ||||||||||||| ||||||||||||||||||||||||||||||||||||||||||||||
Sbjct  2702590  ACGGTTCCGGAAGGAGTTTGCGGTTTTCCGAATTTAACCAAGTTGGATCTTCGTTTAAAC  2702531

Query  181      AGTTTGACTTTTCTTCCGGAATTTATCGGAGAATGTAAACGCCTTGAACAACTTAATCTT  240
```

```
                ||||||||||||||||||||||||||||||||||||||||||||||||||||||||||||
Sbjct  2702530  AGTTTGACTTTTCTTCCGGAATTTATCGGAGAATGTAAACGCCTTGAACAACTTAATCTT  2702471

Query  241      TTCGGGAACGACCTTACAACATTTCCGTCTACGTTTTCTAAATTAAAAAATCTGAAAGTG  300
                ||||||||||||||||| ||||||||||||||||||||||||||||||||||||||||||
Sbjct  2702470  TTCGGGAACGACCTTACGACATTTCCGTCTACGTTTTCTAAATTAAAAAATCTGAAAGTG  2702411

Query  301      TTACTGGCGGGAAATAACGATTTTACGATTCTACCCTCCGAACTTTTGTTTCTTCCGTTG  360
                ||||||||||||||||||||||||||||||||||| |||||||||||||||||||| |||
Sbjct  2702410  TTACTGGCGGGAAATAACGATTTTACGATTCTACCTTCCGAACTTTTGTTTCTTCCATTG  2702351

Query  361      ATCAAAATTCTATACGTAGATCGGAATAAATTAACTCTAACGGAAACGGATGTGGAAATT  420
                ||||||||| |||||||||||||||||||||||||| |||||||||||||||||||||||||
Sbjct  2702350  ATCAAAATTTTATACGTAGATCGGAATAAATTAATTCTAACGGAAACGGATGTGGAAATT  2702291

Query  421      CTCGCTTCTCTTTCCTCCTTGGAGGAATTGGATCTGAGTCTGAATTCGGGGATTAAGGCG  480
                ||||||||||||||||||||||||||||||||||||||||||||||||||||||||||||
Sbjct  2702290  CTCGCTTCTCTTTCCTCCTTGGAGGAATTGGATCTGAGTCTGAATTCGGGGATTAAGGCG  2702231

Query  481      CTCCCTTTTAATTACGAAAAACTTGTAAATCTCATTAATCTAAAAAGATTGAATATTaaa  540
                || ||||||||||||||||||||||| |||||||||||| ||||||||||||||||||||
Sbjct  2702230  CTTCCTTTTAATTACGAAAAACTTGTGAATCTCATTAATTTAAAAAGATTGAATATTAAA  2702171

Query  541      aaaaCTTCATTAAAGGGAGAGGATGCAGATAAATTGCAGGCGATTCTTCCAAATACCAAA  600
                ||||| ||||||||||||||||||||||||||||||||||||||||||||||||||||||
Sbjct  2702170  AAAACATCATTAAAGGGAGAGGATGCAGATAAATTGCAGGCGATTCTTCCAAATACCAAA  2702111

Query  601      ATCGACTACTGA  612
                ||||||||||||
Sbjct  2702110  ATCGACTACTGA  2702099
```

>Leptospira borgpetersenii serovar Hardjo-bovis strain L49 chromosome 1, complete sequence
Sequence ID: CP033440.1 Length: 3620710
Range 1: 946028 to 946639

Score:1064 bits(576), Expect:0.0,
Identities:600/612(98%), Gaps:0/612(0%), Strand: Plus/Plus

```
Query  1       GGCTTCGTTTGTTTTACTGCGAGTTTCGACTGTAAAAAGAATGCGGAAGAGATTTTGGGA  60
               ||||||||||||||||||||||||||| | ||||||||||||||||||||||||||||||
Sbjct  946028  GGCTTCGTTTGTTTTACTGCGAGTTTCAATTGTAAAAAGAATGCGGAAGAGATTTTGGGA  946087

Query  61      GAGGCAAAGGCAAAACCGGAATTGGTTCAAACTTTGGATTTTGGAATGCAGAAGTTGTCC  120
               ||||||||||||||||||||||||||||||||||||||||||||||||||||||||||||
Sbjct  946088  GAGGCAAAGGCAAAACCGGAATTGGTTCAAACTTTGGATTTTGGAATGCAGAAGTTGTCC  946147

Query  121     ACGGTTCCGGAAGAAGTTTGCGGTTTTCCGAATTTAACCAAGTTGGATCTTCGTTTAAAC  180
               ||||||||||||| ||||||||||||||||||||||||||||||||||||||||||||||
Sbjct  946148  ACGGTTCCGGAAGGAGTTTGCGGTTTTCCGAATTTAACCAAGTTGGATCTTCGTTTAAAC  946207

Query  181     AGTTTGACTTTTCTTCCGGAATTTATCGGAGAATGTAAACGCCTTGAACAACTTAATCTT  240
               ||||||||||||||||||||||||||||||||||||||||||||||||||||||||||||
Sbjct  946208  AGTTTGACTTTTCTTCCGGAATTTATCGGAGAATGTAAACGCCTTGAACAACTTAATCTT  946267

Query  241     TTCGGGAACGACCTTACAACATTTCCGTCTACGTTTTCTAAATTAAAAAATCTGAAAGTG  300
               ||||||||||||||||| ||||||||||||||||||||||||||||||||||||||||||
Sbjct  946268  TTCGGGAACGACCTTACGACATTTCCGTCTACGTTTTCTAAATTAAAAAATCTGAAAGTG  946327

Query  301     TTACTGGCGGGAAATAACGATTTTACGATTCTACCCTCCGAACTTTTGTTTCTTCCGTTG  360
```

```
                  ||||||||||||||||||||||||||||||||||| |||||||||||||||||||| |||
Sbjct  946328  TTACTGGCGGGAAATAACGATTTTACGATTCTACCTTCCGAACTTTTGTTTCTTCCATTG  946387

Query  361     ATCAAAATTCTATACGTAGATCGGAATAAATTAACTCTAACGGAAACGGATGTGGAAATT  420
               ||||||||| |||||||||||||||||||||||| |||||||||||||||||||||||||
Sbjct  946388  ATCAAAATTTTATACGTAGATCGGAATAAATTAATTCTAACGGAAACGGATGTGGAAATT  946447

Query  421     CTCGCTTCTCTTTCCTCCTTGGAGGAATTGGATCTGAGTCTGAATTCGGGGATTAAGGCG  480
               ||||||||||||||||||||||||||||||||||||||||||||||||||||||||||||
Sbjct  946448  CTCGCTTCTCTTTCCTCCTTGGAGGAATTGGATCTGAGTCTGAATTCGGGGATTAAGGCG  946507

Query  481     CTCCCTTTTAATTACGAAAAACTTGTAAATCTCATTAATCTAAAAAGATTGAATATTaaa  540
               || ||||||||||||||||||||||| |||||||||||| ||||||||||||||||||||
Sbjct  946508  CTTCCTTTTAATTACGAAAAACTTGTGAATCTCATTAATTTAAAAAGATTGAATATTAAA  946567

Query  541     aaaaCTTCATTAAAGGGAGAGGATGCAGATAAATTGCAGGCGATTCTTCCAAATACCAAA  600
               ||||| ||||||||||||||||||||||||||||||||||||||||||||||||||||||
Sbjct  946568  AAAACATCATTAAAGGGAGAGGATGCAGATAAATTGCAGGCGATTCTTCCAAATACCAAA  946627

Query  601     ATCGACTACTGA  612
               ||||||||||||
Sbjct  946628  ATCGACTACTGA  946639
```

>Leptospira borgpetersenii serovar Hardjo-bovis strain 203 chromosome I, complete sequence
Sequence ID: CP021412.1 Length: 3589981
Range 1: 940162 to 940773

Score:1064 bits(576), Expect:0.0,
Identities:600/612(98%), Gaps:0/612(0%), Strand: Plus/Plus

```
Query  1       GGCTTCGTTTGTTTTACTGCGAGTTTCGACTGTAAAAAGAATGCGGAAGAGATTTTGGGA  60
               ||||||||||||||||||||||||||| | ||||||||||||||||||||||||||||||
Sbjct  940162  GGCTTCGTTTGTTTTACTGCGAGTTTCAATTGTAAAAAGAATGCGGAAGAGATTTTGGGA  940221

Query  61      GAGGCAAAGGCAAAACCGGAATTGGTTCAAACTTTGGATTTTGGAATGCAGAAGTTGTCC  120
               ||||||||||||||||||||||||||||||||||||||||||||||||||||||||||||
Sbjct  940222  GAGGCAAAGGCAAAACCGGAATTGGTTCAAACTTTGGATTTTGGAATGCAGAAGTTGTCC  940281

Query  121     ACGGTTCCGGAAGAAGTTTGCGGTTTTCCGAATTTAACCAAGTTGGATCTTCGTTTAAAC  180
               ||||||||||||| ||||||||||||||||||||||||||||||||||||||||||||||
Sbjct  940282  ACGGTTCCGGAAGGAGTTTGCGGTTTTCCGAATTTAACCAAGTTGGATCTTCGTTTAAAC  940341

Query  181     AGTTTGACTTTTCTTCCGGAATTTATCGGAGAATGTAAACGCCTTGAACAACTTAATCTT  240
               ||||||||||||||||||||||||||||||||||||||||||||||||||||||||||||
Sbjct  940342  AGTTTGACTTTTCTTCCGGAATTTATCGGAGAATGTAAACGCCTTGAACAACTTAATCTT  940401

Query  241     TTCGGGAACGACCTTACAACATTTCCGTCTACGTTTTCTAAATTAAAAAATCTGAAAGTG  300
               ||||||||||||||||| ||||||||||||||||||||||||||||||||||||||||||
Sbjct  940402  TTCGGGAACGACCTTACGACATTTCCGTCTACGTTTTCTAAATTAAAAAATCTGAAAGTG  940461

Query  301     TTACTGGCGGGAAATAACGATTTTACGATTCTACCCTCCGAACTTTTGTTTCTTCCGTTG  360
               ||||||||||||||||||||||||||||||||||| |||||||||||||||||||| |||
Sbjct  940462  TTACTGGCGGGAAATAACGATTTTACGATTCTACCTTCCGAACTTTTGTTTCTTCCATTG  940521

Query  361     ATCAAAATTCTATACGTAGATCGGAATAAATTAACTCTAACGGAAACGGATGTGGAAATT  420
               ||||||||| |||||||||||||||||||||||| |||||||||||||||||||||||||
Sbjct  940522  ATCAAAATTTTATACGTAGATCGGAATAAATTAATTCTAACGGAAACGGATGTGGAAATT  940581

Query  421     CTCGCTTCTCTTTCCTCCTTGGAGGAATTGGATCTGAGTCTGAATTCGGGGATTAAGGCG  480
```

```
                ||||||||||||||||||||||||||||||||||||||||||||||||||||||||||||
Sbjct  940582  CTCGCTTCTCTTTCCTCCTTGGAGGAATTGGATCTGAGTCTGAATTCGGGGATTAAGGCG  940641

Query  481     CTCCCTTTTAATTACGAAAAACTTGTAAATCTCATTAATCTAAAAAGATTGAATATTaaa  540
               || ||||||||||||||||||||||| |||||||||||| ||||||||||||||||||||
Sbjct  940642  CTTCCTTTTAATTACGAAAAACTTGTGAATCTCATTAATTTAAAAAGATTGAATATTAAA  940701

Query  541     aaaaCTTCATTAAAGGGAGAGGATGCAGATAAATTGCAGGCGATTCTTCCAAATACCAAA  600
               ||||| ||||||||||||||||||||||||||||||||||||||||||||||||||||||
Sbjct  940702  AAAACATCATTAAAGGGAGAGGATGCAGATAAATTGCAGGCGATTCTTCCAAATACCAAA  940761

Query  601     ATCGACTACTGA  612
               ||||||||||||
Sbjct  940762  ATCGACTACTGA  940773



>Leptospira borgpetersenii serovar Hardjo strain NVSL S 1343 chromosome 1
Sequence ID: CP015050.1 Length: 3613654
Range 1: 978860 to 979471

Score:1064 bits(576), Expect:0.0,
Identities:600/612(98%),  Gaps:0/612(0%), Strand: Plus/Plus

Query  1       GGCTTCGTTTGTTTTACTGCGAGTTTCGACTGTAAAAAGAATGCGGAAGAGATTTTGGGA  60
               ||||||||||||||||||||||||||| | ||||||||||||||||||||||||||||||
Sbjct  978860  GGCTTCGTTTGTTTTACTGCGAGTTTCAATTGTAAAAAGAATGCGGAAGAGATTTTGGGA  978919

Query  61      GAGGCAAAGGCAAAACCGGAATTGGTTCAAACTTTGGATTTTGGAATGCAGAAGTTGTCC  120
               ||||||||||||||||||||||||||||||||||||||||||||||||||||||||||||
Sbjct  978920  GAGGCAAAGGCAAAACCGGAATTGGTTCAAACTTTGGATTTTGGAATGCAGAAGTTGTCC  978979

Query  121     ACGGTTCCGGAAGAAGTTTGCGGTTTTCCGAATTTAACCAAGTTGGATCTTCGTTTAAAC  180
               ||||||||||||| ||||||||||||||||||||||||||||||||||||||||||||||
Sbjct  978980  ACGGTTCCGGAAGGAGTTTGCGGTTTTCCGAATTTAACCAAGTTGGATCTTCGTTTAAAC  979039

Query  181     AGTTTGACTTTTCTTCCGGAATTTATCGGAGAATGTAAACGCCTTGAACAACTTAATCTT  240
               ||||||||||||||||||||||||||||||||||||||||||||||||||||||||||||
Sbjct  979040  AGTTTGACTTTTCTTCCGGAATTTATCGGAGAATGTAAACGCCTTGAACAACTTAATCTT  979099

Query  241     TTCGGGAACGACCTTACAACATTTCCGTCTACGTTTTCTAAATTAAAAAATCTGAAAGTG  300
               ||||||||||||||||| ||||||||||||||||||||||||||||||||||||||||||
Sbjct  979100  TTCGGGAACGACCTTACGACATTTCCGTCTACGTTTTCTAAATTAAAAAATCTGAAAGTG  979159

Query  301     TTACTGGCGGGAAATAACGATTTTACGATTCTACCCTCCGAACTTTTGTTTCTTCCGTTG  360
               ||||||||||||||||||||||||||||||||||| |||||||||||||||||||| |||
Sbjct  979160  TTACTGGCGGGAAATAACGATTTTACGATTCTACCTTCCGAACTTTTGTTTCTTCCATTG  979219

Query  361     ATCAAAATTCTATACGTAGATCGGAATAAATTAACTCTAACGGAAACGGATGTGGAAATT  420
               ||||||||| |||||||||||||||||||||||| |||||||||||||||||||||||||
Sbjct  979220  ATCAAAATTTTATACGTAGATCGGAATAAATTAATTCTAACGGAAACGGATGTGGAAATT  979279

Query  421     CTCGCTTCTCTTTCCTCCTTGGAGGAATTGGATCTGAGTCTGAATTCGGGGATTAAGGCG  480
               ||||||||||||||||||||||||||||||||||||||||||||||||||||||||||||
Sbjct  979280  CTCGCTTCTCTTTCCTCCTTGGAGGAATTGGATCTGAGTCTGAATTCGGGGATTAAGGCG  979339

Query  481     CTCCCTTTTAATTACGAAAAACTTGTAAATCTCATTAATCTAAAAAGATTGAATATTaaa  540
               || ||||||||||||||||||||||| |||||||||||| ||||||||||||||||||||
Sbjct  979340  CTTCCTTTTAATTACGAAAAACTTGTGAATCTCATTAATTTAAAAAGATTGAATATTAAA  979399

Query  541     aaaaCTTCATTAAAGGGAGAGGATGCAGATAAATTGCAGGCGATTCTTCCAAATACCAAA  600
               ||||| ||||||||||||||||||||||||||||||||||||||||||||||||||||||
```

```
Sbjct  979400  AAAACATCATTAAAGGGAGAGGATGCAGATAAATTGCAGGCGATTCTTCCAAATACCAAA  979459

Query  601     ATCGACTACTGA  612
               ||||||||||||
Sbjct  979460  ATCGACTACTGA  979471
```

>Leptospira borgpetersenii serovar Hardjo strain BK-30 chromosome 1
Sequence ID: CP015048.1 Length: 3629289
Range 1: 979247 to 979858

Score:1064 bits(576), Expect:0.0,
Identities:600/612(98%), Gaps:0/612(0%), Strand: Plus/Plus

```
Query  1       GGCTTCGTTTGTTTTACTGCGAGTTTCGACTGTAAAAAGAATGCGGAAGAGATTTTGGGA  60
               ||||||||||||||||||||||||||| | ||||||||||||||||||||||||||||||
Sbjct  979247  GGCTTCGTTTGTTTTACTGCGAGTTTCAATTGTAAAAAGAATGCGGAAGAGATTTTGGGA  979306

Query  61      GAGGCAAAGGCAAAACCGGAATTGGTTCAAACTTTGGATTTTGGAATGCAGAAGTTGTCC  120
               ||||||||||||||||||||||||||||||||||||||||||||||||||||||||||||
Sbjct  979307  GAGGCAAAGGCAAAACCGGAATTGGTTCAAACTTTGGATTTTGGAATGCAGAAGTTGTCC  979366

Query  121     ACGGTTCCGGAAGAAGTTTGCGGTTTTCCGAATTTAACCAAGTTGGATCTTCGTTTAAAC  180
               ||||||||||||| ||||||||||||||||||||||||||||||||||||||||||||||
Sbjct  979367  ACGGTTCCGGAAGGAGTTTGCGGTTTTCCGAATTTAACCAAGTTGGATCTTCGTTTAAAC  979426

Query  181     AGTTTGACTTTTCTTCCGGAATTTATCGGAGAATGTAAACGCCTTGAACAACTTAATCTT  240
               ||||||||||||||||||||||||||||||||||||||||||||||||||||||||||||
Sbjct  979427  AGTTTGACTTTTCTTCCGGAATTTATCGGAGAATGTAAACGCCTTGAACAACTTAATCTT  979486

Query  241     TTCGGGAACGACCTTACAACATTTCCGTCTACGTTTTCTAAATTAAAAAATCTGAAAGTG  300
               ||||||||||||||||| ||||||||||||||||||||||||||||||||||||||||||
Sbjct  979487  TTCGGGAACGACCTTACGACATTTCCGTCTACGTTTTCTAAATTAAAAAATCTGAAAGTG  979546

Query  301     TTACTGGCGGGAAATAACGATTTTACGATTCTACCCTCCGAACTTTTGTTTCTTCCGTTG  360
               ||||||||||||||||||||||||||||||||||| |||||||||||||||||||| |||
Sbjct  979547  TTACTGGCGGGAAATAACGATTTTACGATTCTACCTTCCGAACTTTTGTTTCTTCCATTG  979606

Query  361     ATCAAAATTCTATACGTAGATCGGAATAAATTAACTCTAACGGAAACGGATGTGGAAATT  420
               ||||||||| |||||||||||||||||||||||| |||||||||||||||||||||||||
Sbjct  979607  ATCAAAATTTTATACGTAGATCGGAATAAATTAATTCTAACGGAAACGGATGTGGAAATT  979666

Query  421     CTCGCTTCTCTTTCCTCCTTGGAGGAATTGGATCTGAGTCTGAATTCGGGGATTAAGGCG  480
               ||||||||||||||||||||||||||||||||||||||||||||||||||||||||||||
Sbjct  979667  CTCGCTTCTCTTTCCTCCTTGGAGGAATTGGATCTGAGTCTGAATTCGGGGATTAAGGCG  979726

Query  481     CTCCCTTTTAATTACGAAAAACTTGTAAATCTCATTAATCTAAAAAGATTGAATATTaaa  540
               || ||||||||||||||||||||||| |||||||||||| ||||||||||||||||||||
Sbjct  979727  CTTCCTTTTAATTACGAAAAACTTGTGAATCTCATTAATTTAAAAAGATTGAATATTAAA  979786

Query  541     aaaaCTTCATTAAAGGGAGAGGATGCAGATAAATTGCAGGCGATTCTTCCAAATACCAAA  600
               ||||| ||||||||||||||||||||||||||||||||||||||||||||||||||||||
Sbjct  979787  AAAACATCATTAAAGGGAGAGGATGCAGATAAATTGCAGGCGATTCTTCCAAATACCAAA  979846

Query  601     ATCGACTACTGA  612
               ||||||||||||
Sbjct  979847  ATCGACTACTGA  979858
```

```
>Leptospira borgpetersenii serovar Hardjo strain BK-9 chromosome 1
Sequence ID: CP015046.1 Length: 3631634
Range 1: 979077 to 979688

Score:1064 bits(576), Expect:0.0,
Identities:600/612(98%),  Gaps:0/612(0%), Strand: Plus/Plus

Query  1       GGCTTCGTTTGTTTTACTGCGAGTTTCGACTGTAAAAAGAATGCGGAAGAGATTTTGGGA  60
               ||||||||||||||||||||||||||| | ||||||||||||||||||||||||||||||
Sbjct  979077  GGCTTCGTTTGTTTTACTGCGAGTTTCAATTGTAAAAAGAATGCGGAAGAGATTTTGGGA  979136

Query  61      GAGGCAAAGGCAAAACCGGAATTGGTTCAAACTTTGGATTTTGGAATGCAGAAGTTGTCC  120
               ||||||||||||||||||||||||||||||||||||||||||||||||||||||||||||
Sbjct  979137  GAGGCAAAGGCAAAACCGGAATTGGTTCAAACTTTGGATTTTGGAATGCAGAAGTTGTCC  979196

Query  121     ACGGTTCCGGAAGAAGTTTGCGGTTTTCCGAATTTAACCAAGTTGGATCTTCGTTTAAAC  180
               ||||||||||||| ||||||||||||||||||||||||||||||||||||||||||||||
Sbjct  979197  ACGGTTCCGGAAGGAGTTTGCGGTTTTCCGAATTTAACCAAGTTGGATCTTCGTTTAAAC  979256

Query  181     AGTTTGACTTTTCTTCCGGAATTTATCGGAGAATGTAAACGCCTTGAACAACTTAATCTT  240
               ||||||||||||||||||||||||||||||||||||||||||||||||||||||||||||
Sbjct  979257  AGTTTGACTTTTCTTCCGGAATTTATCGGAGAATGTAAACGCCTTGAACAACTTAATCTT  979316

Query  241     TTCGGGAACGACCTTACAACATTTCCGTCTACGTTTTCTAAATTAAAAAATCTGAAAGTG  300
               ||||||||||||||||| ||||||||||||||||||||||||||||||||||||||||||
Sbjct  979317  TTCGGGAACGACCTTACGACATTTCCGTCTACGTTTTCTAAATTAAAAAATCTGAAAGTG  979376

Query  301     TTACTGGCGGGAAATAACGATTTTACGATTCTACCCTCCGAACTTTTGTTTCTTCCGTTG  360
               ||||||||||||||||||||||||||||||||||| |||||||||||||||||||| |||
Sbjct  979377  TTACTGGCGGGAAATAACGATTTTACGATTCTACCTTCCGAACTTTTGTTTCTTCCATTG  979436

Query  361     ATCAAAATTCTATACGTAGATCGGAATAAATTAACTCTAACGGAAACGGATGTGGAAATT  420
               ||||||||| |||||||||||||||||||||||| |||||||||||||||||||||||||
Sbjct  979437  ATCAAAATTTTATACGTAGATCGGAATAAATTAATTCTAACGGAAACGGATGTGGAAATT  979496

Query  421     CTCGCTTCTCTTTCCTCCTTGGAGGAATTGGATCTGAGTCTGAATTCGGGGATTAAGGCG  480
               ||||||||||||||||||||||||||||||||||||||||||||||||||||||||||||
Sbjct  979497  CTCGCTTCTCTTTCCTCCTTGGAGGAATTGGATCTGAGTCTGAATTCGGGGATTAAGGCG  979556

Query  481     CTCCCTTTTAATTACGAAAAACTTGTAAATCTCATTAATCTAAAAAGATTGAATATTaaa  540
               || ||||||||||||||||||||||| |||||||||||| ||||||||||||||||||||
Sbjct  979557  CTTCCTTTTAATTACGAAAAACTTGTGAATCTCATTAATTTAAAAAGATTGAATATTAAA  979616

Query  541     aaaaCTTCATTAAAGGGAGAGGATGCAGATAAATTGCAGGCGATTCTTCCAAATACCAAA  600
               ||||| ||||||||||||||||||||||||||||||||||||||||||||||||||||||
Sbjct  979617  AAAACATCATTAAAGGGAGAGGATGCAGATAAATTGCAGGCGATTCTTCCAAATACCAAA  979676

Query  601     ATCGACTACTGA  612
               ||||||||||||
Sbjct  979677  ATCGACTACTGA  979688




>Leptospira borgpetersenii serovar Hardjo strain BK-6 chromosome 1
Sequence ID: CP015044.1 Length: 3649879
Range 1: 977825 to 978436

Score:1064 bits(576), Expect:0.0,
Identities:600/612(98%),  Gaps:0/612(0%), Strand: Plus/Plus

Query  1       GGCTTCGTTTGTTTTACTGCGAGTTTCGACTGTAAAAAGAATGCGGAAGAGATTTTGGGA  60
               ||||||||||||||||||||||||||| | ||||||||||||||||||||||||||||||
```

```
Sbjct  977825  GGCTTCGTTTGTTTTACTGCGAGTTTCAATTGTAAAAAGAATGCGGAAGAGATTTTGGGA  977884

Query  61      GAGGCAAAGGCAAAACCGGAATTGGTTCAAACTTTGGATTTTGGAATGCAGAAGTTGTCC  120
               ||||||||||||||||||||||||||||||||||||||||||||||||||||||||||||
Sbjct  977885  GAGGCAAAGGCAAAACCGGAATTGGTTCAAACTTTGGATTTTGGAATGCAGAAGTTGTCC  977944

Query  121     ACGGTTCCGGAAGAAGTTTGCGGTTTTCCGAATTTAACCAAGTTGGATCTTCGTTTAAAC  180
               ||||||||||||| ||||||||||||||||||||||||||||||||||||||||||||||
Sbjct  977945  ACGGTTCCGGAAGGAGTTTGCGGTTTTCCGAATTTAACCAAGTTGGATCTTCGTTTAAAC  978004

Query  181     AGTTTGACTTTTCTTCCGGAATTTATCGGAGAATGTAAACGCCTTGAACAACTTAATCTT  240
               ||||||||||||||||||||||||||||||||||||||||||||||||||||||||||||
Sbjct  978005  AGTTTGACTTTTCTTCCGGAATTTATCGGAGAATGTAAACGCCTTGAACAACTTAATCTT  978064

Query  241     TTCGGGAACGACCTTACAACATTTCCGTCTACGTTTTCTAAATTAAAAAATCTGAAAGTG  300
               ||||||||||||||||| ||||||||||||||||||||||||||||||||||||||||||
Sbjct  978065  TTCGGGAACGACCTTACGACATTTCCGTCTACGTTTTCTAAATTAAAAAATCTGAAAGTG  978124

Query  301     TTACTGGCGGGAAATAACGATTTTACGATTCTACCCTCCGAACTTTTGTTTCTTCCGTTG  360
               ||||||||||||||||||||||||||||||||||| |||||||||||||||||||| |||
Sbjct  978125  TTACTGGCGGGAAATAACGATTTTACGATTCTACCTTCCGAACTTTTGTTTCTTCCATTG  978184

Query  361     ATCAAAATTCTATACGTAGATCGGAATAAATTAACTCTAACGGAAACGGATGTGGAAATT  420
               ||||||||| |||||||||||||||||||||||||| |||||||||||||||||||||||
Sbjct  978185  ATCAAAATTTTATACGTAGATCGGAATAAATTAATTCTAACGGAAACGGATGTGGAAATT  978244

Query  421     CTCGCTTCTCTTTCCTCCTTGGAGGAATTGGATCTGAGTCTGAATTCGGGGATTAAGGCG  480
               ||||||||||||||||||||||||||||||||||||||||||||||||||||||||||||
Sbjct  978245  CTCGCTTCTCTTTCCTCCTTGGAGGAATTGGATCTGAGTCTGAATTCGGGGATTAAGGCG  978304

Query  481     CTCCCTTTTAATTACGAAAAACTTGTAAATCTCATTAATCTAAAAAGATTGAATATTaaa  540
               || ||||||||||||||||||||||| |||||||||||| ||||||||||||||||||||
Sbjct  978305  CTTCCTTTTAATTACGAAAAACTTGTGAATCTCATTAATTTAAAAAGATTGAATATTAAA  978364

Query  541     aaaaCTTCATTAAAGGGAGAGGATGCAGATAAATTGCAGGCGATTCTTCCAAATACCAAA  600
               ||||| ||||||||||||||||||||||||||||||||||||||||||||||||||||||
Sbjct  978365  AAAACATCATTAAAGGGAGAGGATGCAGATAAATTGCAGGCGATTCTTCCAAATACCAAA  978424

Query  601     ATCGACTACTGA  612
               ||||||||||||
Sbjct  978425  ATCGACTACTGA  978436
```

>Leptospira borgpetersenii strain KR39 chromosome 1, complete sequence
Sequence ID: CP096127.1 Length: 3603773
Range 1: 936053 to 936664

Score:1064 bits(576), Expect:0.0,
Identities:600/612(98%), Gaps:0/612(0%), Strand: Plus/Plus

```
Query  1       GGCTTCGTTTGTTTTACTGCGAGTTTCGACTGTAAAAAGAATGCGGAAGAGATTTTGGGA  60
               ||||||||||||||||||||||||||| | ||||||||||||||||||||||||||||||
Sbjct  936053  GGCTTCGTTTGTTTTACTGCGAGTTTCAATTGTAAAAAGAATGCGGAAGAGATTTTGGGA  936112

Query  61      GAGGCAAAGGCAAAACCGGAATTGGTTCAAACTTTGGATTTTGGAATGCAGAAGTTGTCC  120
               ||||||||||||||||||||||||||||||||||||||||||||||||||||||||||||
Sbjct  936113  GAGGCAAAGGCAAAACCGGAATTGGTTCAAACTTTGGATTTTGGAATGCAGAAGTTGTCC  936172

Query  121     ACGGTTCCGGAAGAAGTTTGCGGTTTTCCGAATTTAACCAAGTTGGATCTTCGTTTAAAC  180
               ||||||||||||| ||||||||||||||||||||||||||||||||||||||||||||||
Sbjct  936173  ACGGTTCCGGAAGGAGTTTGCGGTTTTCCGAATTTAACCAAGTTGGATCTTCGTTTAAAC  936232
```

```
Query  181     AGTTTGACTTTTCTTCCGGAATTTATCGGAGAATGTAAACGCCTTGAACAACTTAATCTT  240
               ||||||||||||||||||||||||||||||||||||||||||||||||||||||||||||
Sbjct  936233  AGTTTGACTTTTCTTCCGGAATTTATCGGAGAATGTAAACGCCTTGAACAACTTAATCTT  936292

Query  241     TTCGGGAACGACCTTACAACATTTCCGTCTACGTTTTCTAAATTAAAAAATCTGAAAGTG  300
               ||||||||||||||||| ||||||||||||||||||||||||||||||||||||||||||
Sbjct  936293  TTCGGGAACGACCTTACGACATTTCCGTCTACGTTTTCTAAATTAAAAAATCTGAAAGTG  936352

Query  301     TTACTGGCGGGAAATAACGATTTTACGATTCTACCCTCCGAACTTTTGTTTCTTCCGTTG  360
               ||||||||||||||||||||||||||||||||||| |||||||||||||||||||| |||
Sbjct  936353  TTACTGGCGGGAAATAACGATTTTACGATTCTACCTTCCGAACTTTTGTTTCTTCCATTG  936412

Query  361     ATCAAAATTCTATACGTAGATCGGAATAAATTAACTCTAACGGAAACGGATGTGGAAATT  420
               ||||||||| |||||||||||||||||||||||| |||||||||||||||||||||||||
Sbjct  936413  ATCAAAATTTTATACGTAGATCGGAATAAATTAATTCTAACGGAAACGGATGTGGAAATT  936472

Query  421     CTCGCTTCTCTTTCCTCCTTGGAGGAATTGGATCTGAGTCTGAATTCGGGGATTAAGGCG  480
               ||||||||||||||||||||||||||||||||||||||||||||||||||||||||||||
Sbjct  936473  CTCGCTTCTCTTTCCTCCTTGGAGGAATTGGATCTGAGTCTGAATTCGGGGATTAAGGCG  936532

Query  481     CTCCCTTTTAATTACGAAAAACTTGTAAATCTCATTAATCTAAAAAGATTGAATATTaaa  540
               || ||||||||||||||||||||||| |||||||||||| ||||||||||||||||||||
Sbjct  936533  CTTCCTTTTAATTACGAAAAACTTGTGAATCTCATTAATTTAAAAAGATTGAATATTAAA  936592

Query  541     aaaaCTTCATTAAAGGGAGAGGATGCAGATAAATTGCAGGCGATTCTTCCAAATACCAAA  600
               ||||| ||||||||||||||||||||||||||||||||||||||||||||||||||||||
Sbjct  936593  AAAACATCATTAAAGGGAGAGGATGCAGATAAATTGCAGGCGATTCTTCCAAATACCAAA  936652

Query  601     ATCGACTACTGA  612
               ||||||||||||
Sbjct  936653  ATCGACTACTGA  936664
```

>Leptospira borgpetersenii serovar Hardjo strain TC129 chromosome I, complete sequence
Sequence ID: CP084038.1 Length: 3585597
Range 1: 935940 to 936551

Score:1064 bits(576), Expect:0.0,
Identities:600/612(98%), Gaps:0/612(0%), Strand: Plus/Plus

```
Query  1       GGCTTCGTTTGTTTTACTGCGAGTTTCGACTGTAAAAAGAATGCGGAAGAGATTTTGGGA  60
               ||||||||||||||||||||||||||| | ||||||||||||||||||||||||||||||
Sbjct  935940  GGCTTCGTTTGTTTTACTGCGAGTTTCAATTGTAAAAAGAATGCGGAAGAGATTTTGGGA  935999

Query  61      GAGGCAAAGGCAAAACCGGAATTGGTTCAAACTTTGGATTTTGGAATGCAGAAGTTGTCC  120
               ||||||||||||||||||||||||||||||||||||||||||||||||||||||||||||
Sbjct  936000  GAGGCAAAGGCAAAACCGGAATTGGTTCAAACTTTGGATTTTGGAATGCAGAAGTTGTCC  936059

Query  121     ACGGTTCCGGAAGAAGTTTGCGGTTTTCCGAATTTAACCAAGTTGGATCTTCGTTTAAAC  180
               ||||||||||||| ||||||||||||||||||||||||||||||||||||||||||||||
Sbjct  936060  ACGGTTCCGGAAGGAGTTTGCGGTTTTCCGAATTTAACCAAGTTGGATCTTCGTTTAAAC  936119

Query  181     AGTTTGACTTTTCTTCCGGAATTTATCGGAGAATGTAAACGCCTTGAACAACTTAATCTT  240
               ||||||||||||||||||||||||||||||||||||||||||||||||||||||||||||
Sbjct  936120  AGTTTGACTTTTCTTCCGGAATTTATCGGAGAATGTAAACGCCTTGAACAACTTAATCTT  936179

Query  241     TTCGGGAACGACCTTACAACATTTCCGTCTACGTTTTCTAAATTAAAAAATCTGAAAGTG  300
               ||||||||||||||||| ||||||||||||||||||||||||||||||||||||||||||
Sbjct  936180  TTCGGGAACGACCTTACGACATTTCCGTCTACGTTTTCTAAATTAAAAAATCTGAAAGTG  936239
```

```
Query  301     TTACTGGCGGGAAATAACGATTTTACGATTCTACCCTCCGAACTTTTGTTTCTTCCGTTG  360
               ||||||||||||||||||||||||||||||||||| |||||||||||||||||||| |||
Sbjct  936240  TTACTGGCGGGAAATAACGATTTTACGATTCTACCTTCCGAACTTTTGTTTCTTCCATTG  936299

Query  361     ATCAAAATTCTATACGTAGATCGGAATAAATTAACTCTAACGGAAACGGATGTGGAAATT  420
               ||||||||| |||||||||||||||||||||||| |||||||||||||||||||||||||
Sbjct  936300  ATCAAAATTTTATACGTAGATCGGAATAAATTAATTCTAACGGAAACGGATGTGGAAATT  936359

Query  421     CTCGCTTCTCTTTCCTCCTTGGAGGAATTGGATCTGAGTCTGAATTCGGGGATTAAGGCG  480
               ||||||||||||||||||||||||||||||||||||||||||||||||||||||||||||
Sbjct  936360  CTCGCTTCTCTTTCCTCCTTGGAGGAATTGGATCTGAGTCTGAATTCGGGGATTAAGGCG  936419

Query  481     CTCCCTTTTAATTACGAAAAACTTGTAAATCTCATTAATCTAAAAAGATTGAATATTaaa  540
               || ||||||||||||||||||||||| |||||||||||| ||||||||||||||||||||
Sbjct  936420  CTTCCTTTTAATTACGAAAAACTTGTGAATCTCATTAATTTAAAAAGATTGAATATTAAA  936479

Query  541     aaaaCTTCATTAAAGGGAGAGGATGCAGATAAATTGCAGGCGATTCTTCCAAATACCAAA  600
               ||||| ||||||||||||||||||||||||||||||||||||||||||||||||||||||
Sbjct  936480  AAAACATCATTAAAGGGAGAGGATGCAGATAAATTGCAGGCGATTCTTCCAAATACCAAA  936539

Query  601     ATCGACTACTGA  612
               ||||||||||||
Sbjct  936540  ATCGACTACTGA  936551
```

```
>Leptospira borgpetersenii strain TC147 chromosome I, complete sequence
Sequence ID: CP084040.1 Length: 3585599
Range 1: 935940 to 936551

Score:1064 bits(576), Expect:0.0,
Identities:600/612(98%),  Gaps:0/612(0%), Strand: Plus/Plus

Query  1       GGCTTCGTTTGTTTTACTGCGAGTTTCGACTGTAAAAGAATGCGGAAGAGATTTTGGGA  60
               |||||||||||||||||||||||||| | |||||||||||||||||||||||||||||||
Sbjct  935940  GGCTTCGTTTGTTTTACTGCGAGTTTCAATTGTAAAAGAATGCGGAAGAGATTTTGGGA  935999

Query  61      GAGGCAAAGGCAAAACCGGAATTGGTTCAAACTTTGGATTTTGGAATGCAGAAGTTGTCC  120
               ||||||||||||||||||||||||||||||||||||||||||||||||||||||||||||
Sbjct  936000  GAGGCAAAGGCAAAACCGGAATTGGTTCAAACTTTGGATTTTGGAATGCAGAAGTTGTCC  936059

Query  121     ACGGTTCCGGAAGAAGTTTGCGGTTTTCCGAATTTAACCAAGTTGGATCTTCGTTTAAAC  180
               ||||||||||||| ||||||||||||||||||||||||||||||||||||||||||||||
Sbjct  936060  ACGGTTCCGGAAGGAGTTTGCGGTTTTCCGAATTTAACCAAGTTGGATCTTCGTTTAAAC  936119

Query  181     AGTTTGACTTTTCTTCCGGAATTTATCGGAGAATGTAAACGCCTTGAACAACTTAATCTT  240
               ||||||||||||||||||||||||||||||||||||||||||||||||||||||||||||
Sbjct  936120  AGTTTGACTTTTCTTCCGGAATTTATCGGAGAATGTAAACGCCTTGAACAACTTAATCTT  936179

Query  241     TTCGGGAACGACCTTACAACATTTCCGTCTACGTTTTCTAAATTAAAAAATCTGAAAGTG  300
               ||||||||||||||||| ||||||||||||||||||||||||||||||||||||||||||
Sbjct  936180  TTCGGGAACGACCTTACGACATTTCCGTCTACGTTTTCTAAATTAAAAAATCTGAAAGTG  936239

Query  301     TTACTGGCGGGAAATAACGATTTTACGATTCTACCCTCCGAACTTTTGTTTCTTCCGTTG  360
               ||||||||||||||||||||||||||||||||||| |||||||||||||||||||| |||
Sbjct  936240  TTACTGGCGGGAAATAACGATTTTACGATTCTACCTTCCGAACTTTTGTTTCTTCCATTG  936299

Query  361     ATCAAAATTCTATACGTAGATCGGAATAAATTAACTCTAACGGAAACGGATGTGGAAATT  420
               ||||||||| |||||||||||||||||||||||| |||||||||||||||||||||||||
Sbjct  936300  ATCAAAATTTTATACGTAGATCGGAATAAATTAATTCTAACGGAAACGGATGTGGAAATT  936359

Query  421     CTCGCTTCTCTTTCCTCCTTGGAGGAATTGGATCTGAGTCTGAATTCGGGGATTAAGGCG  480
```

```
                 ||||||||||||||||||||||||||||||||||||||||||||||||||||||||||||
Sbjct  936360  CTCGCTTCTCTTTCCTCCTTGGAGGAATTGGATCTGAGTCTGAATTCGGGGATTAAGGCG  936419

Query  481     CTCCCTTTTAATTACGAAAAACTTGTAAATCTCATTAATCTAAAAAGATTGAATATTaaa  540
               || ||||||||||||||||||||||| |||||||||||| ||||||||||||||||||||
Sbjct  936420  CTTCCTTTTAATTACGAAAAACTTGTGAATCTCATTAATTTAAAAAGATTGAATATTAAA  936479

Query  541     aaaaCTTCATTAAAGGGAGAGGATGCAGATAAATTGCAGGCGATTCTTCCAAATACCAAA  600
               ||||| ||||||||||||||||||||||||||||||||||||||||||||||||||||||
Sbjct  936480  AAAACATCATTAAAGGGAGAGGATGCAGATAAATTGCAGGCGATTCTTCCAAATACCAAA  936539

Query  601     ATCGACTACTGA  612
               ||||||||||||
Sbjct  936540  ATCGACTACTGA  936551
```

>Leptospira borgpetersenii strain TC273 chromosome I, complete sequence
Sequence ID: CP084042.1 Length: 3585577
Range 1: 935929 to 936540

Score:1064 bits(576), Expect:0.0,
Identities:600/612(98%), Gaps:0/612(0%), Strand: Plus/Plus

```
Query  1       GGCTTCGTTTGTTTTACTGCGAGTTTCGACTGTAAAAAGAATGCGGAAGAGATTTTGGGA  60
               ||||||||||||||||||||||||||| | ||||||||||||||||||||||||||||||
Sbjct  935929  GGCTTCGTTTGTTTTACTGCGAGTTTCAATTGTAAAAAGAATGCGGAAGAGATTTTGGGA  935988

Query  61      GAGGCAAAGGCAAAACCGGAATTGGTTCAAACTTTGGATTTTGGAATGCAGAAGTTGTCC  120
               ||||||||||||||||||||||||||||||||||||||||||||||||||||||||||||
Sbjct  935989  GAGGCAAAGGCAAAACCGGAATTGGTTCAAACTTTGGATTTTGGAATGCAGAAGTTGTCC  936048

Query  121     ACGGTTCCGGAAGAAGTTTGCGGTTTTCCGAATTTAACCAAGTTGGATCTTCGTTTAAAC  180
               ||||||||||||| ||||||||||||||||||||||||||||||||||||||||||||||
Sbjct  936049  ACGGTTCCGGAAGGAGTTTGCGGTTTTCCGAATTTAACCAAGTTGGATCTTCGTTTAAAC  936108

Query  181     AGTTTGACTTTTCTTCCGGAATTTATCGGAGAATGTAAACGCCTTGAACAACTTAATCTT  240
               ||||||||||||||||||||||||||||||||||||||||||||||||||||||||||||
Sbjct  936109  AGTTTGACTTTTCTTCCGGAATTTATCGGAGAATGTAAACGCCTTGAACAACTTAATCTT  936168

Query  241     TTCGGGAACGACCTTACAACATTTCCGTCTACGTTTTCTAAATTAAAAAATCTGAAAGTG  300
               ||||||||||||||||| ||||||||||||||||||||||||||||||||||||||||||
Sbjct  936169  TTCGGGAACGACCTTACGACATTTCCGTCTACGTTTTCTAAATTAAAAAATCTGAAAGTG  936228

Query  301     TTACTGGCGGGAAATAACGATTTTACGATTCTACCCTCCGAACTTTTGTTTCTTCCGTTG  360
               ||||||||||||||||||||||||||||||||||| |||||||||||||||||||| |||
Sbjct  936229  TTACTGGCGGGAAATAACGATTTTACGATTCTACCTTCCGAACTTTTGTTTCTTCCATTG  936288

Query  361     ATCAAAATTCTATACGTAGATCGGAATAAATTAACTCTAACGGAAACGGATGTGGAAATT  420
               ||||||||| |||||||||||||||||||||||| |||||||||||||||||||||||||
Sbjct  936289  ATCAAAATTTTATACGTAGATCGGAATAAATTAATTCTAACGGAAACGGATGTGGAAATT  936348

Query  421     CTCGCTTCTCTTTCCTCCTTGGAGGAATTGGATCTGAGTCTGAATTCGGGGATTAAGGCG  480
               ||||||||||||||||||||||||||||||||||||||||||||||||||||||||||||
Sbjct  936349  CTCGCTTCTCTTTCCTCCTTGGAGGAATTGGATCTGAGTCTGAATTCGGGGATTAAGGCG  936408

Query  481     CTCCCTTTTAATTACGAAAAACTTGTAAATCTCATTAATCTAAAAAGATTGAATATTaaa  540
               || ||||||||||||||||||||||| |||||||||||| ||||||||||||||||||||
Sbjct  936409  CTTCCTTTTAATTACGAAAAACTTGTGAATCTCATTAATTTAAAAAGATTGAATATTAAA  936468

Query  541     aaaaCTTCATTAAAGGGAGAGGATGCAGATAAATTGCAGGCGATTCTTCCAAATACCAAA  600
               ||||| ||||||||||||||||||||||||||||||||||||||||||||||||||||||
```

```
Sbjct  936469  AAAACATCATTAAAGGGAGAGGATGCAGATAAATTGCAGGCGATTCTTCCAAATACCAAA  936528

Query  601     ATCGACTACTGA  612
               ||||||||||||
Sbjct  936529  ATCGACTACTGA  936540
```

```
>Leptospira borgpetersenii strain TC112 chromosome I, complete sequence
Sequence ID: CP084036.1 Length: 3585599
Range 1: 935940 to 936551

Score:1064 bits(576), Expect:0.0,
Identities:600/612(98%),  Gaps:0/612(0%), Strand: Plus/Plus

Query  1       GGCTTCGTTTGTTTTACTGCGAGTTTCGACTGTAAAAAGAATGCGGAAGAGATTTTGGGA  60
               ||||||||||||||||||||||||||| | ||||||||||||||||||||||||||||||
Sbjct  935940  GGCTTCGTTTGTTTTACTGCGAGTTTCAATTGTAAAAAGAATGCGGAAGAGATTTTGGGA  935999

Query  61      GAGGCAAAGGCAAAACCGGAATTGGTTCAAACTTTGGATTTTGGAATGCAGAAGTTGTCC  120
               ||||||||||||||||||||||||||||||||||||||||||||||||||||||||||||
Sbjct  936000  GAGGCAAAGGCAAAACCGGAATTGGTTCAAACTTTGGATTTTGGAATGCAGAAGTTGTCC  936059

Query  121     ACGGTTCCGGAAGAAGTTTGCGGTTTTCCGAATTTAACCAAGTTGGATCTTCGTTTAAAC  180
               ||||||||||||| ||||||||||||||||||||||||||||||||||||||||||||||
Sbjct  936060  ACGGTTCCGGAAGGAGTTTGCGGTTTTCCGAATTTAACCAAGTTGGATCTTCGTTTAAAC  936119

Query  181     AGTTTGACTTTTCTTCCGGAATTTATCGGAGAATGTAAACGCCTTGAACAACTTAATCTT  240
               ||||||||||||||||||||||||||||||||||||||||||||||||||||||||||||
Sbjct  936120  AGTTTGACTTTTCTTCCGGAATTTATCGGAGAATGTAAACGCCTTGAACAACTTAATCTT  936179

Query  241     TTCGGGAACGACCTTACAACATTTCCGTCTACGTTTTCTAAATTAAAAAATCTGAAAGTG  300
               ||||||||||||||||| ||||||||||||||||||||||||||||||||||||||||||
Sbjct  936180  TTCGGGAACGACCTTACGACATTTCCGTCTACGTTTTCTAAATTAAAAAATCTGAAAGTG  936239

Query  301     TTACTGGCGGGAAATAACGATTTTACGATTCTACCCTCCGAACTTTTGTTTCTTCCGTTG  360
               ||||||||||||||||||||||||||||||||||| |||||||||||||||||||| |||
Sbjct  936240  TTACTGGCGGGAAATAACGATTTTACGATTCTACCTTCCGAACTTTTGTTTCTTCCATTG  936299

Query  361     ATCAAAATTCTATACGTAGATCGGAATAAATTAACTCTAACGGAAACGGATGTGGAAATT  420
               ||||||||| |||||||||||||||||||||||| |||||||||||||||||||||||||
Sbjct  936300  ATCAAAATTTTATACGTAGATCGGAATAAATTAATTCTAACGGAAACGGATGTGGAAATT  936359

Query  421     CTCGCTTCTCTTTCCTCCTTGGAGGAATTGGATCTGAGTCTGAATTCGGGGATTAAGGCG  480
               ||||||||||||||||||||||||||||||||||||||||||||||||||||||||||||
Sbjct  936360  CTCGCTTCTCTTTCCTCCTTGGAGGAATTGGATCTGAGTCTGAATTCGGGGATTAAGGCG  936419

Query  481     CTCCCTTTTAATTACGAAAAACTTGTAAATCTCATTAATCTAAAAAGATTGAATATTaaa  540
               || ||||||||||||||||||||||| |||||||||||| ||||||||||||||||||||
Sbjct  936420  CTTCCTTTTAATTACGAAAAACTTGTGAATCTCATTAATTTAAAAAGATTGAATATTAAA  936479

Query  541     aaaaCTTCATTAAAGGGAGAGGATGCAGATAAATTGCAGGCGATTCTTCCAAATACCAAA  600
               ||||| ||||||||||||||||||||||||||||||||||||||||||||||||||||||
Sbjct  936480  AAAACATCATTAAAGGGAGAGGATGCAGATAAATTGCAGGCGATTCTTCCAAATACCAAA  936539

Query  601     ATCGACTACTGA  612
               ||||||||||||
Sbjct  936540  ATCGACTACTGA  936551
```

```
>Leptospira borgpetersenii serovar Hardjo-bovis L550 chromosome 1, complete sequence
Sequence ID: CP000348.1 Length: 3614446
Range 1: 969260 to 969871

Score:1064 bits(576), Expect:0.0,
Identities:600/612(98%),  Gaps:0/612(0%), Strand: Plus/Plus

Query  1       GGCTTCGTTTGTTTTACTGCGAGTTTCGACTGTAAAAAGAATGCGGAAGAGATTTTGGGA  60
               ||||||||||||||||||||||||||| | ||||||||||||||||||||||||||||||
Sbjct  969260  GGCTTCGTTTGTTTTACTGCGAGTTTCAATTGTAAAAAGAATGCGGAAGAGATTTTGGGA  969319

Query  61      GAGGCAAAGGCAAAACCGGAATTGGTTCAAACTTTGGATTTTGGAATGCAGAAGTTGTCC  120
               ||||||||||||||||||||||||||||||||||||||||||||||||||||||||||||
Sbjct  969320  GAGGCAAAGGCAAAACCGGAATTGGTTCAAACTTTGGATTTTGGAATGCAGAAGTTGTCC  969379

Query  121     ACGGTTCCGGAAGAAGTTTGCGGTTTTCCGAATTTAACCAAGTTGGATCTTCGTTTAAAC  180
               ||||||||||||| ||||||||||||||||||||||||||||||||||||||||||||||
Sbjct  969380  ACGGTTCCGGAAGGAGTTTGCGGTTTTCCGAATTTAACCAAGTTGGATCTTCGTTTAAAC  969439

Query  181     AGTTTGACTTTTCTTCCGGAATTTATCGGAGAATGTAAACGCCTTGAACAACTTAATCTT  240
               ||||||||||||||||||||||||||||||||||||||||||||||||||||||||||||
Sbjct  969440  AGTTTGACTTTTCTTCCGGAATTTATCGGAGAATGTAAACGCCTTGAACAACTTAATCTT  969499

Query  241     TTCGGGAACGACCTTACAACATTTCCGTCTACGTTTTCTAAATTAAAAAATCTGAAAGTG  300
               ||||||||||||||||| ||||||||||||||||||||||||||||||||||||||||||
Sbjct  969500  TTCGGGAACGACCTTACGACATTTCCGTCTACGTTTTCTAAATTAAAAAATCTGAAAGTG  969559

Query  301     TTACTGGCGGGAAATAACGATTTTACGATTCTACCCTCCGAACTTTTGTTTCTTCCGTTG  360
               ||||||||||||||||||||||||||||||||||| |||||||||||||||||||| |||
Sbjct  969560  TTACTGGCGGGAAATAACGATTTTACGATTCTACCTTCCGAACTTTTGTTTCTTCCATTG  969619

Query  361     ATCAAAATTCTATACGTAGATCGGAATAAATTAACTCTAACGGAAACGGATGTGGAAATT  420
               ||||||||| |||||||||||||||||||||||| |||||||||||||||||||||||||
Sbjct  969620  ATCAAAATTTTATACGTAGATCGGAATAAATTAATTCTAACGGAAACGGATGTGGAAATT  969679

Query  421     CTCGCTTCTCTTTCCTCCTTGGAGGAATTGGATCTGAGTCTGAATTCGGGGATTAAGGCG  480
               ||||||||||||||||||||||||||||||||||||||||||||||||||||||||||||
Sbjct  969680  CTCGCTTCTCTTTCCTCCTTGGAGGAATTGGATCTGAGTCTGAATTCGGGGATTAAGGCG  969739

Query  481     CTCCCTTTTAATTACGAAAAACTTGTAAATCTCATTAATCTAAAAAGATTGAATATTaaa  540
               || ||||||||||||||||||||||| |||||||||||| ||||||||||||||||||||
Sbjct  969740  CTTCCTTTTAATTACGAAAAACTTGTGAATCTCATTAATTTAAAAAGATTGAATATTAAA  969799

Query  541     aaaaCTTCATTAAAGGGAGAGGATGCAGATAAATTGCAGGCGATTCTTCCAAATACCAAA  600
               ||||| ||||||||||||||||||||||||||||||||||||||||||||||||||||||
Sbjct  969800  AAAACATCATTAAAGGGAGAGGATGCAGATAAATTGCAGGCGATTCTTCCAAATACCAAA  969859

Query  601     ATCGACTACTGA  612
               ||||||||||||
Sbjct  969860  ATCGACTACTGA  969871
```
