# Supplementary material for: Leptospira borgpetersenii Leucine-Rich Repeat Proteins Provide Strong Protective Efficacy as Novel Leptospiral Vaccine Candidates
Source: Trop Med Infect Dis. 2022 Dec 22;8(1):6. doi: 10.3390/tropicalmed8010006 (PMC9863753; doi:10.3390/tropicalmed8010006)
Supplement: Supplementary file 1 [file tropicalmed-08-00006-s001.zip › Supplementary Figure S1.pdf]

A

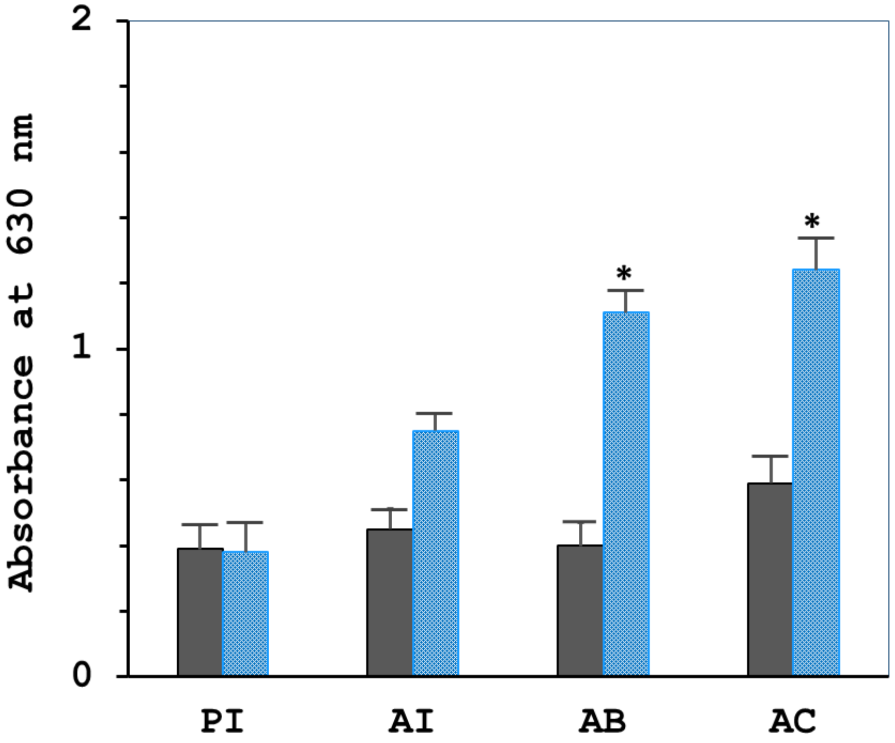

2
1
0
Absorbance at 630 nm
*
*
PI
AI
AB
AC

B

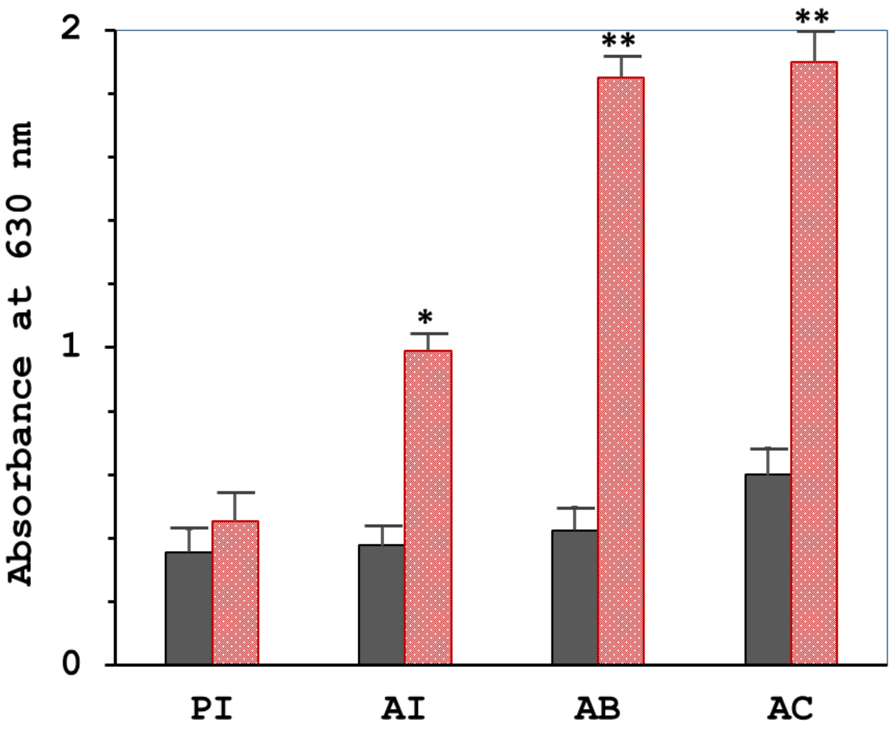

2
1
0
Absorbance at 630 nm
*
**
**
PI
AI
AB
AC

Supplementary Figure S1. Induction of specific humoral immune responses in hamsters immunized with LRR proteins, either the rhKU_Sej_LRR_2012 or the rhKU_Sej_LRR_2271. The **(A)** rhKU_Sej_LRR_2012-specific IgG and **(B)** the rhKU_Sej_LRR_2271-specific IgG were evaluated in experiment 1 hamster sera at dilution 1:1,000 by ELISA. The level of specific IgG production shown on Y-axis were compared between the control (Gray bar) and immunized animals with each protein (Green: the rhKU_Sej_LRR_2012 or Orange: the rhKU_Sej_LRR_2271 bars). Sera from the control and immunized animals were collected on day 0 (pre-immunization, PI), day 21 (3 weeks after the 1st immunization, AI), day 42 (3 weeks after the 2nd immunization or after 1st boost, AB), and day 49 – 63 (7 – 21 days after the challenge, AC) as shown on X-axis. The data represent the mean of eight hamsters per treatment group ± S.E.M. The absorbance values that higher than 2.0 were calculated from 2 to 3 folds dilution of the original diluted samples. The *, ** indicate significant differences of *p value* <0.05, <0.01 by using the Kruskal-Wallis one-way ANOVA analysis, respectively.
